# Inhibition of Cancer Stem-like Cells by Curcumin and Other Polyphenol Derivatives in MDA-MB-231 TNBC Cells

**DOI:** 10.3390/ijms25137446

**Published:** 2024-07-06

**Authors:** Maria Ros, Gerard Riesco-Llach, Emma Polonio-Alcalá, Pere Miquel Morla-Barcelo, Santiago Ruiz-Martínez, Lidia Feliu, Marta Planas, Teresa Puig

**Affiliations:** 1 New Therapeutic Targets Laboratory (TargetsLab)-Oncology Unit, Department of Medical Sciences, Faculty of Medicine, University of Girona, 17003 Girona, Spain; 2 Laboratori d’Innovació en Processos i Productes de Síntesi Orgànica (LIPPSO), Department of Chemistry, University of Girona, 17003 Girona, Spainmarta.planas@udg.edu (M.P.); 3 Grupo Multidisciplinar de Oncología Traslacional, Institut Universitari d’Investigació en Ciències de la Salut (IUNICS), Universitat de les Illes Balears, 07122 Palma, Spain; 4 Department of Laboratory Medicine, Institute of Biomedicine, Sahlgrenska Academy, Sahlgrenska Center for Cancer Research, University of Gothenburg, 405 30 Gothenburg, Sweden

**Keywords:** polyphenols, curcumin, triple-negative breast cancer, cancer stem cells

## Abstract

Triple-negative breast cancer (TNBC) accounts for 15% of all breast cancers and is highly aggressive. Despite an initial positive response to chemotherapy, most patients experience rapid disease progression leading to relapse and metastasis. This is attributed to the presence of breast cancer stem cells (BCSCs) within the tumor, which are characterized by self-renewal, pluripotency, and resistance mechanisms. Targeting BCSCs has become critical as conventional therapies fail to eradicate them due to a lack of specific targets. Curcumin, a polyphenol derived from turmeric (*Curcuma longa*), exhibits anticancer effects against breast cancer cells and BCSCs. The use of curcumin derivatives has been suggested as an approach to overcome the bioavailability and solubility problems of curcumin in humans, thereby increasing its anticancer effects. The aim of this study was to evaluate the cellular and molecular effects of six synthetic compounds derived from the natural polyphenol epigallocatechin gallate (EGCG) (TL1, TL2) and curcumin derivatives (TL3, TL4, TL5, and TL6) on a TNBC mesenchymal stem-like cell line. The activity of the compounds against BCSCs was also determined by a mammosphere inhibition assay and studying different BCSC markers by Western blotting. Finally, a drug combination assay was performed with the most promising compounds to evaluate their potential synergistic effects with the chemotherapeutic agents doxorubicin, cisplatin, and paclitaxel. The results showed that compounds exhibited specific cytotoxicity against the TNBC cell line and BCSCs. Interestingly, the combination of the curcumin derivative TL3 with doxorubicin and cisplatin displayed a synergistic effect in TNBC cells.

## 1. Introduction

Breast cancer (BCa) is the most common and second most fatal cancer among Western women [1]. Within the different molecular subtypes of BCa, triple-negative breast cancer (TNBC) refers to tumors that lack expression of estrogen and progesterone receptors, as well as overexpression and/or amplification of the human epidermal growth factor receptor-2 (HER2). TNBC constitutes the most lethal subtype of BCa, accounting for 15–20% of all BCas [2], and showing a higher incidence among young women. Despite an initial positive response to chemotherapy, the lack of specific targets, poor cell differentiation, high molecular heterogeneity, and metastatic potential of the tumor result in an only 30% survival rate for TNBC patients beyond 5 years from diagnosis [3]. Unfortunately, chemotherapy constitutes the main treatment, since targeted therapies remain infeasible due to the absence of specific markers [4]. Consequently, scientific efforts are currently focused on searching for new targets and pharmacological treatments for these patients.

The aggressiveness of TNBC is potentially driven by a subpopulation of stem-like cells within breast tumors, the so-called breast cancer stem cells (BCSCs) [4]. These cells represent 1–3% of tumor cells and exhibit unique properties, such as self-renewal and pluripotency or the ability to invade and metastasize to other parts of the body [5]. Through asymmetric division, they are able to maintain their own population and to differentiate into any of the tumorigenic cells, allowing the reconstitution of the heterogeneous tumor cell population [6]. Current therapies lack specific targets to attack this cell population. Hence, it is important to identify novel therapies that selectively target the BCSC population. BCSCs are characterized by CD44^+^/CD24^-^ cell surface marker expression, increased expression of the enzyme aldehyde dehydrogenase 1 (ALDH1), and the ability to grow as spheres in suspension [7,8]. Furthermore, several signaling pathways are altered in BCSCs, including the embryonic signaling pathways Notch, Wnt/β-catenin, and Hedgehog. During the process of carcinogenesis, alterations in these pathways allow cancer cells to acquire stemness properties, thereby increasing proliferation, tolerance, and dedifferentiation [9,10,11].

**Figure 1:**
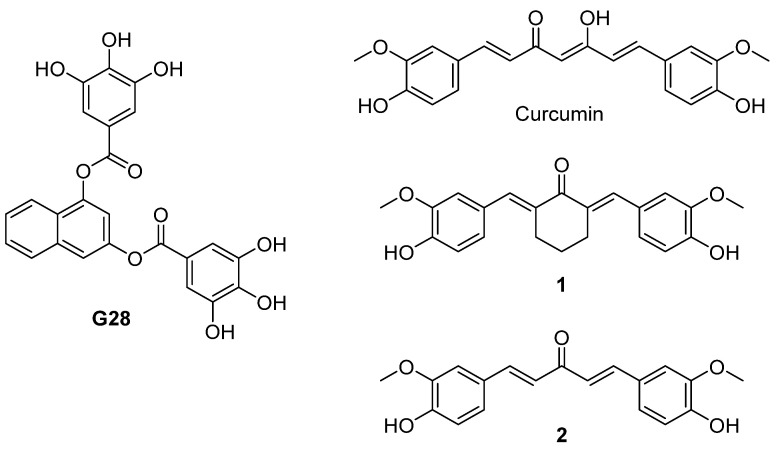
Structure of G28, curcumin, and curcumin analogues **1** and **2**.

A large number of plant secondary metabolites, especially polyphenols, have historically been employed as antitumor agents. The effects of these dietary compounds are better tolerated by patients than conventional anticancer therapies. Some examples include epigallocatechin-3-gallate (EGCG), curcumin, resveratrol, and emodin [12]. EGCG, in particular, is a polyphenol present in green tea (*Camelia sinensis*) that exhibits antiproliferative, antimetastatic, and pro-apoptotic activities. This catechin has also demonstrated antiproliferative effects in TNBC cells through the inhibition of the lipogenic enzyme fatty acid synthase [13,14]. Since EGCG has limited bioavailability and poor stability in serum, new analogues have been developed. In this context, Turrado et al. designed and synthesized a collection of compounds incorporating two galloyl esters linked to a cyclic subunit. Three of these compounds, namely G28, G37, and G56, resulted to be the most active against HER2+ BCa cells. G28 also exhibited good pharmacokinetic parameters and demonstrated potent tumor volume reduction in vivo (Figure 1) [15]. Furthermore, G28 showed significant antiproliferative activity in TNBC and the capacity to act against the BCSC population with a notable inhibition of mammosphere formation [13,16,17,18].

On the other hand, curcumin is a polyphenol extracted from the rhizome of turmeric (*Curcuma longa*), widely used in Eastern medicine for its antioxidant, anti-inflammatory, antimicrobial, and antiviral properties (Figure 1). It also has potential anticancer properties, which are still under investigation, and can regulate growth factors, enzymes, transcription factors, kinases, as well as inflammatory cytokines and pro- and antiapoptotic proteins [19,20]. In BCa cells, curcumin shows antiproliferative and anti-invasive effects. Alone or in combination with other drugs, it has been shown to be an effective approach to treat BCa [21,22,23]. However, curcumin has very low bioavailability and low absorption in humans, caused by its poor water solubility and rapid systemic elimination. To overcome these limitations and enhance the specific cytotoxicity of curcumin towards cancer cells, researchers have designed and synthesized analogues of this natural polyphenol [24,25,26]. For instance, replacement of the diketone moiety of curcumin with a cyclohexanone led to 2,6-bis(4-hydroxy-3-methoxybenzylidene)cyclohexan-1-one (**1**), which displayed a lower IC_50_ on murine 4T1 breast cancer cells and better in vivo antitumor properties than curcumin [27] (Figure 1). The 1,3-diketone was also substituted by a ketone group, the resulting (1*E*,4*E*)-1,5-bis(4-hydroxy-3-methoxyphenyl)penta-1,4-dien-3-one (**2**) also being more potent than curcumin [28].

**Figure 2:**
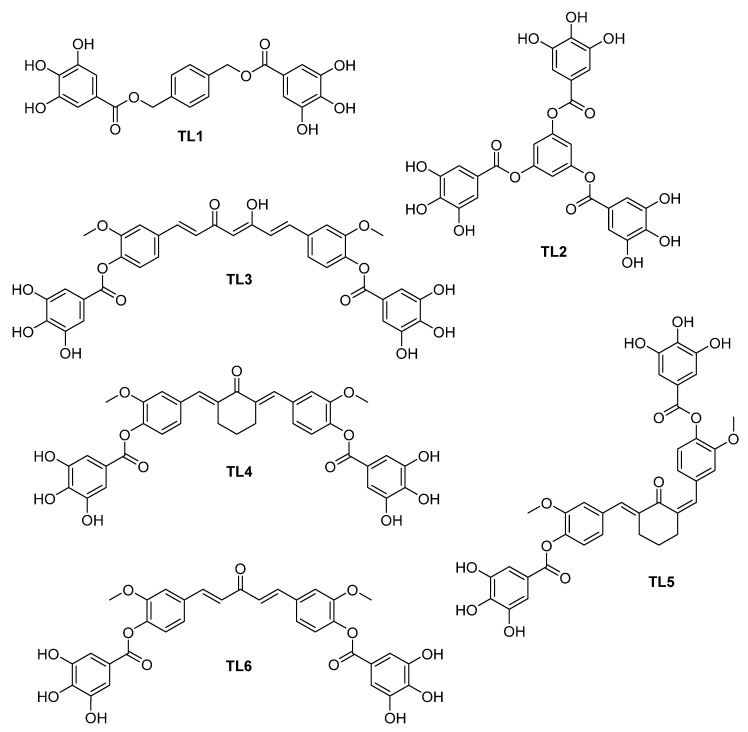
Structures of polyphenols TL1–TL6.

Taking advantage of the antitumor properties of the polyphenol G28, and of curcumin and its derivatives **1** and **2**, herein we planned to design and study the new polyphenolic compounds TL1-TL6 (Figure 2). The cytotoxic potential of these compounds against an MDA-MB-231 TNBC cell model and its BCSC subpopulation were evaluated. Alterations in common signaling pathways and cell death markers were also analyzed. Finally, drug combination assays with chemotherapeutic agents were performed using the most feasible derivatives.

## 2. Results

### 2.1. Design of Polyphenols TL1–TL6

Polyphenols TL1–TL6 were designed based on the structure of G28, curcumin, or curcumin analogues **1** and **2** (Figure 1 and Figure 2). TL1 and TL2 are G28 analogues resulting from the substitution of the naphthalene-1,3-diyl central core of G28 with a 1,4-phenylenebis(methylene) or a benzene-1,3,5-triyl, respectively. In TL1, the sp^3^ carbon between the cyclic subunit and the galloyl esters provides a more flexible structure. TL2 was designed to assess the influence in the activity of a third galloyl ester. Polyphenols TL3-TL6 are curcumin derivatives bearing two galloyl esters linked to the curcumin scaffold (TL3), to the curcumin analogue **1** (TL4 and TL5) or to the curcumin analogue **2** (TL6). TL4 and its isomer TL5 contain a cyclohexanone instead of the 1,3-diketone moiety present in curcumin, whereas TL6 incorporates a single ketone group.

### 2.2. Synthesis of Polyphenols TL1–TL6

**Scheme 1:**
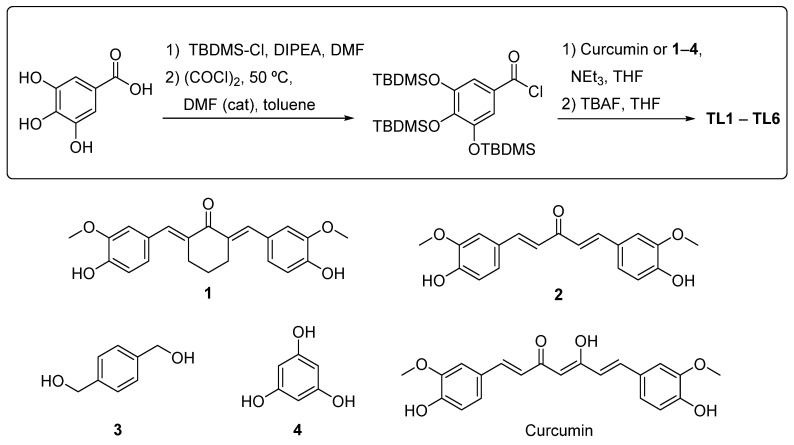
Synthetic strategy for the preparation of polyphenols TL1–TL6.

Polyphenols TL1–TL6 were synthesized following a previously reported strategy [17] (Scheme 1). The protocol involved the following: (i) protection of gallic acid with the *tert*-butyldimethylsilyl group (TBDMS); (ii) activation of the carboxylic acid group of the TBDMS-protected gallic acid; (iii) esterification with curcumin or the corresponding alcohol **1**–**4**; and (iv) removal of the TBDMS group. Curcumin and alcohols **3** and **4** are commercially available, whereas curcumin analogues **1** and **2** were prepared as previously described [29,30]. Finally, TL1–TL6 compounds were fully characterized by ^1^H and ^13^C-NMR.

### 2.3. Analysis of Cytotoxicity and Selectivity of Curcumin and Polyphenols

The cytotoxic effect of TL1–TL6 on the TNBC MDA-MB-231 cell line was evaluated using an MTT assay (Table 1 and Supplementary Figure S2). Curcumin was included for comparison purposes, whereas the cytotoxicity of G28 had already been studied [18]. Prior to the cytotoxicity assays of the compounds, the toxicity of the vehicle (DMSO) on the MDA-MB-231 cell line was also evaluated (See Supplementary Figure S1A). This assay revealed that DMSO was toxic to the cells at concentrations higher than 0.8%. Therefore, the DMSO concentration used in the treatments with the compounds did not exceed 0.8%.

All compounds exhibited cytotoxicity against MDA-MB-231 cells. TL3, TL4, TL5, and TL6 displayed half-maximal inhibitory concentration (IC_50_) values significantly lower than that of curcumin (*p* < 0.001) (Table 1 and Figure 2), being, therefore, significantly more cytotoxic. Notably, the IC_50_ of TL4 (0.86 μM) was 20 times lower than that of curcumin and was significantly lower than that of TL5 (1.31 μM; *p* = 0.0073) and TL6 (3.58 μM; *p* = 0.0002). Due to the high IC_50_ values of G28, TL1, and TL2, these compounds were no longer used in the following experiments.

Additionally, the cytotoxicity of TL3 and TL4, with the lowest IC_50_ values, was assayed against the non-tumor 184B5 cell line obtained from mammary epithelial cells in order to assess the selectivity of these compounds for cancer cells in comparison to non-tumoral cells (Supplementary Figure S1B). The results indicated that these compounds did not show a specific cytotoxicity or adverse effects against mammary epithelial cells.

### 2.4. Effect of Curcumin and Its Derivatives TL3–TL6 on BCSCs

**Figure 3:**
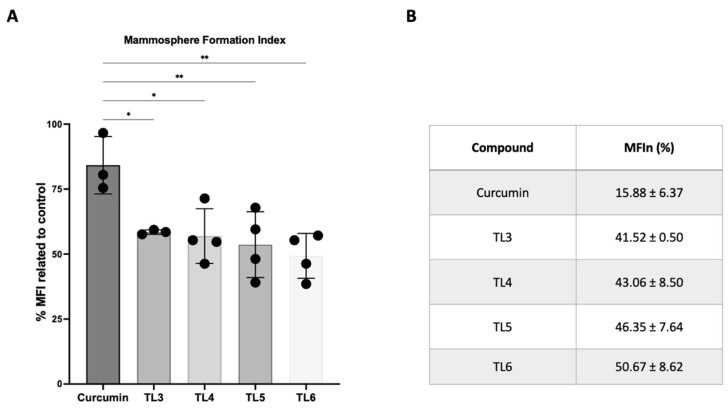
Effect of curcumin and TL3–TL6 on breast cancer stem cells (BCSCs) in MDA-MB-231 cells evaluated using a mammosphere formation assay after treatment with concentrations equivalent to their IC_30_ for 7 days. (**A**) Mammosphere Formation Index (MFI) for each compound. Significant differences in MFI compared to curcumin treatment are indicated as * (*p* < 0.05) and ** (*p* < 0.01). (**B**) Mammosphere Inhibition Index (MFIn) for each compound.

Firstly, the efficacy of curcumin and the polyphenolic derivatives TL3–TL6 against BCSCs was assessed using the mammosphere formation assay. A general reduction in mammosphere size and a decrease in the number of mammospheres were observed in all the treatments with TL3–TL6 at thirty percent inhibitory concentration (IC_30_), compared to the control, demonstrating their noteworthy efficacy in inhibiting mammosphere formation. Furthermore, TL3–TL6 showed a significantly lower MFI compared to the parent compound, indicating that curcumin has a lower efficacy than its derivatives in targeting the BCSC subpopulation (Figure 3A). Regarding MFIn (Figure 3B), the MFIn value of TL3, TL4, TL5, and TL6 indicated higher potency compared to curcumin; what is more, the concentration used by these compounds was at least 13 times lower. Interestingly, TL4, even at a lower concentration than TL3, TL5, and TL6, exhibited similar results in BCSC inhibition.

**Figure 4:**
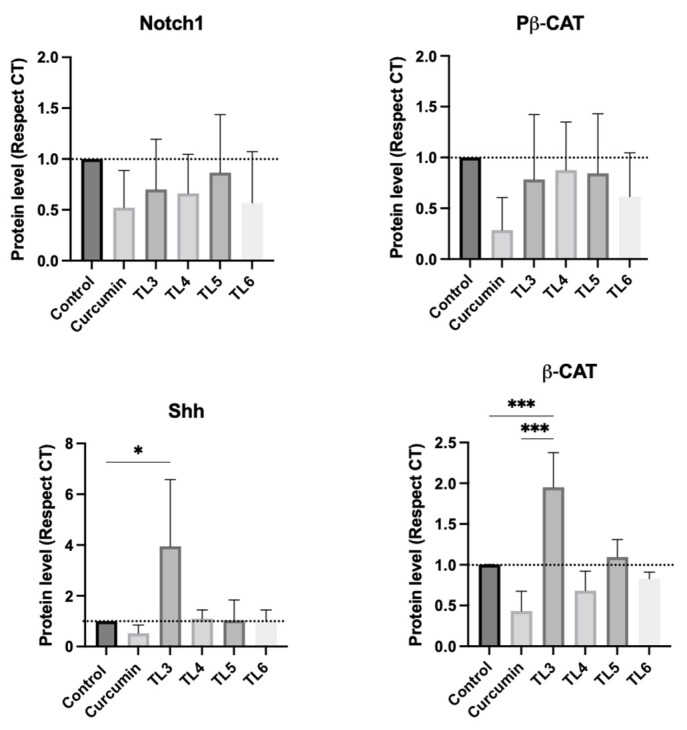
Notch1, Shh, and β-catenin protein expression analysis of MDA-MB-231 cells treated with curcumin and polyphenols TL3–TL6 for 72 h. GAPDH was used as a loading control. Quantified Western blotting results obtained using ImageLab software (Image Lab Software for Mac Version 6.1). Expression ratios are relativized with GAPDH and control group (untreated cells). Significant differences are indicated as * (*p* < 0.05) and *** (*p* < 0.001). See Western blots in Supplementary Figure S3A.

To validate the mammosphere assay findings for curcumin and its derivatives (TL3–TL6), the most relevant embryonic signaling pathways and stemness markers were characterized by Western blotting (Figure 4). The results revealed that curcumin reduced the expression of Notch1, β-catenin, and Shh proteins, resulting in the inhibition of these embryonic signaling pathways. In contrast, polyphenols TL3–TL6 showed distinct effects on the pathways. Notably, these compounds reduced the activation of the Notch signaling pathway, especially TL4 and TL6. Surprisingly, TL3 appeared to promote the activation of the Shh signaling pathway, while TL4, TL5, and TL6 did not induce such overactivation. Specifically, TL3 significantly increased Shh expression compared to curcumin. Regarding the Wnt/β-catenin pathway, TL4 and TL6 reduced the expression of β-catenin protein, whereas TL3 and TL5 enhanced its activation. Furthermore, TL3 significantly increased β-catenin compared to curcumin and untreated cells.

Therefore, although curcumin has been shown to be a useful compound for the inhibition of key embryonic pathways, the effects of its derivatives were not uniform. TL4 and TL6 inhibited β-catenin expression while TL4, TL5, and TL6 did not induce overactivation of Shh expression.

**Figure 5:**
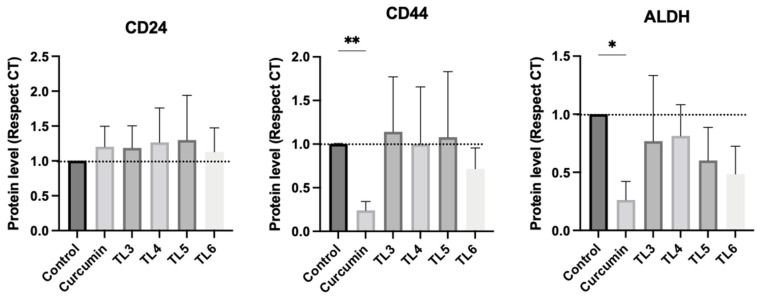
CD24, CD44, and ALDH1 protein expression analysis of MDA-MB-231 cells treated with curcumin and polyphenols TL3–TL6 for 72 h. GAPDH was used as a loading control. Quantified Western blotting results obtained using ImageLab software (Image Lab Software for Mac Version 6.1). Expression ratios are relativized with GAPDH and control group (untreated cells). Significant differences are indicated as * (*p* < 0.05) and ** (*p* < 0.01). See Western blots in Supplementary Figure S3B.

Protein expression analysis of key stemness markers in BCSCs, specifically CD24, CD44, and ALDH1, were also characterized (Figure 5). The results demonstrated that curcumin significantly decreased the expression of CD44 and ALDH1 compared to the control. These findings indicated an inhibitory effect of curcumin on BCSCs. Regarding the polyphenols, TL5–TL6 treatment also decreased ALDH1 expression. In addition, TL6 caused a reduction in CD44 expression, although this tendency was not statistically significant. With regard to CD24, no changes were observed when treated with TL3–TL6.

### 2.5. TL3-doxorubicin/TL3-cisplatin: A Synergetic Combination for TNBC

Combination therapies involving curcumin with other chemotherapeutic agents have allowed for the sensitization of BCSCs and the reduction in drug doses, thereby minimizing side effects and avoiding the emerge of resistance [31]. Considering that chemotherapy is the main treatment option for TNBC in the clinical setting, drug combination assays were performed to study the pharmacological interactions of curcumin-derived compounds in combination with doxorubicin, cisplatin, or paclitaxel, which are commonly used chemotherapeutic agents in medical practice. Specifically, compounds TL3 and TL4 were evaluated in combination with these chemotherapeutics due to their low IC_50_ values and the promising MFI found.

**Figure 6:**
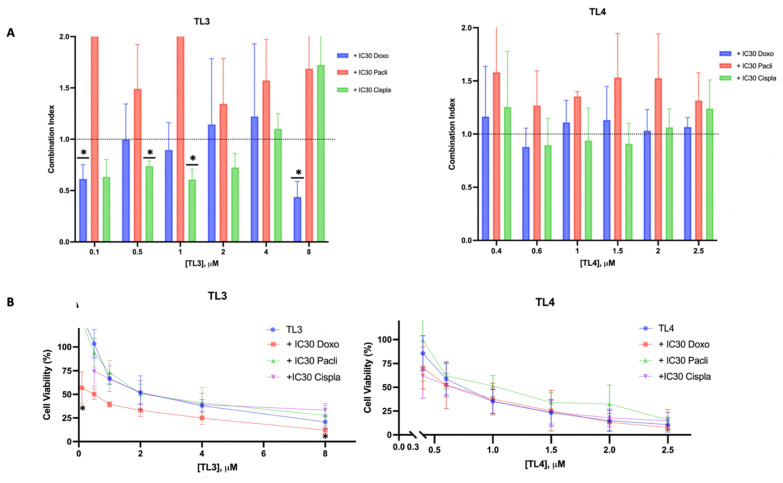
Combinatorial treatment between TL3 or TL4 and chemotherapeutic agents at concentrations equivalent to their IC_30_ (Doxo: doxorubicin; Pacli: paclitaxel; and Cispla: cisplatin) in MDA-MB-231 cells. (**A**) Combination index (CI) from treatments with TL3 or TL4 and chemotherapeutic agents. Data are expressed as mean ± SD from three independent experiments and are based on the Chou and Talalay method. The dotted line indicates additive effect (CI approximately equal to 1). CI > 1 is indicative of an antagonistic effect and CI < 1 a synergistic effect. Significant differences are indicated as * (*p* < 0.05). (**B**) Dose–response curves of TL3 (from 0.1 to 8 µM) or TL4 (from 0.4 to 2.5 µM) alone and in combination with chemotherapeutic agents at concentrations equivalent to their IC_30_ for 72 h. Results shown are mean ± SE from at least three independent experiments.

The results showed that in the case of compound TL4, the combination index (CI) values at each concentration were higher than one, indicating an antagonistic effect between the chemotherapeutic agent and TL4 (Figure 6A). Remarkably, when the compound TL3 was added at 0.1 and 8 μM in combination with doxorubicin or at 0.5 and 1 μM combined with cisplatin, a synergistic effect was evident (CI < 1). Therefore, in these cases, the curcumin-derived compound enhanced the effects of doxorubicin and cisplatin, leading to greater toxicity in tumor cells. This synergistic effect of TL3 and doxorubicin is supported by the cell viability curves in Figure 6B, which demonstrated significant cell cytotoxicity at 0.1 and 8 μM (*p* = 0.0400 and 0.0234, respectively) compared to TL3 alone. Although this effect was not clear in the cell viability curve for cisplatin, the CI values were lower than one for all TL3 concentrations, except for 4 and 8 μM, being significant at 0.5 and 1 μM (*p* = 0.0111 and *p* = 0.0215, respectively) (Figure 6B). Notably, some of these synergistic effects of TL3 with doxorubicin and cisplatin were achieved at very low doses of TL3 and using an IC_30_ of the chemotherapeutic agents. However, these results were not achieved with paclitaxel, and in most cases, an antagonistic effect was observed.

## 3. Discussion

The aggressiveness of TNBC is primarily due to the presence of a subpopulation of cells with stem cell properties within the tumor, known as BCSCs [6]. Current therapies are unable to eradicate them because no specific target for this type of cells has been identified, although curcumin has been shown to be effective against them. The use of derivatives of curcumin has been suggested as a strategy to overcome the bioavailability and solubility problems of curcumin in humans, allowing for increased cytotoxicity against cancer cells. The use of curcumin delivery systems, such as encapsulation and nanoparticles or liposomes, are other pharmacological strategies that have been proposed [32,33]. This study aimed to evaluate the effect of novel polyphenolic compounds on the TNBC MDA-MB-231 cell line and its BCSC population. In addition, the pharmacological interaction between these derivatives and commonly used chemotherapeutic agents was analyzed to assess their potential application in the treatment of TNBC patients.

The cytotoxic activity of the polyphenols derived from G28, curcumin, or curcumin derivatives **1** and **2** was evaluated on TNBC MDA-MB-231 cells. Regarding the G28 analogues, it was observed that the replacement of the naphthalene core with a 1,4-phenylenebis(methylene) resulted in a considerably more active derivative, TL1. This result could be attributed to the increase in the distance between the two galloyl ester groups caused by the presence of the 1,4-phenylenebis(methylene). This effect was previously reported by Turrado et al. for other G28 analogues [16]. In contrast, the incorporation of an additional galloyl group did not lead to an improvement of the cytotoxicity, TL2 being as active as G28. Concerning the polyphenols derived from curcumin or curcumin analogues **1** and **2**, TL3-TL6 were significantly more active than the parent compound. The two galloyl groups present in TL3–TL6 could be responsible for this higher activity. Among them, TL3, TL4, and TL5 were similarly active, pointing out that the replacement of the 1,3-diketone moiety by a cyclohexanone ring does not influence the activity. TL6, which contains a single ketone group, was slightly less active than TL3–TL5. It is noteworthy that the isomeric polyphenols TL4 and TL5 showed similar activity, although it has been observed that small variations in the configuration of compounds can lead to significant differences in their cytotoxicity. Furthermore, it has been described that curcumin analogues **1** and **2**, designed to circumvent the limitations of curcumin, are more active than this natural compound. In contrast, the results obtained in this study revealed that the activity of the curcumin diester TL3 did not differ significantly to that of TL4–TL6, diesters of **1** and **2**. Interestingly, the two polyphenols TL3 and TL4, with the lowest IC_50_ values against TNBC MDA-MB-231 cells, were not cytotoxic against non-tumorigenic 184B5 breast epithelial cells. These two compounds have selective cytotoxicity against breast cancer cells and are therefore promising agents for targeted therapy of both cancer cells and BCSCs.

Regarding the activity of compounds against BCSCs, the results obtained in the mammosphere formation assays showed that curcumin has a lower efficacy than its derivatives. These results are consistent with those obtained by Zhou et al., who found that curcumin had little ability to reduce the spread of BCSCs [21]. Also, the MFIn results had a high standard deviation. This could be a consequence of the cell line used, which has a low efficiency of mammosphere formation compared to other cell lines, such as the estrogen and progesterone receptor expressing MCF-7 [34]. However, TL4 stood out among all the derivatives as it produced a similar MFIn effect with the lowest concentration.

As previously discussed, the BCSC population exhibits alterations in several cell signaling pathways, including the Notch, Wnt/β-catenin, and Hedgehog embryonic pathways. These alterations allow these cells to acquire stemness properties [35]. Consequently, the inhibition of these pathways was explored as compounds targeted this malignant population. Regarding the Notch pathway, previous studies have shown that curcumin can inhibit the pathway receptor and some components of the pathway [26]. In this study, curcumin and TL3–TL6 treatments were shown to inhibit this embryonic pathway. As aberrant activation of the Notch pathway has been associated with BCa development, it plays a key role in the differentiation and maintenance of stem cell subpopulations [36]. Therefore, by inhibiting this embryonic pathway, curcumin and TL3–TL6 may target the BCSC subpopulation.

With regard to Shh and Wnt/β-catenin, these embryonic pathways are involved in maintenance and proliferation of BCSCs, further contributing to tumorigenesis. Researchers reported the potential of curcumin to reduce this malignant subpopulation by inhibiting these pathways [36], which is in agreement with our results. Although TL4 and TL6 reduced the Wnt/β-catenin activation, the derivatives TL3 and TL5 triggered the activation of Hedgehog signaling, and also TL3 activated the Wnt/β-catenin pathway (Figure 4). This response could potentially be attributed to a compensatory resistance mechanism triggered by these treatments, given the observed reduction in the Notch pathway and the MFI. In fact, it has been reported that Notch inhibitors, such as psoralidin, inhibit cell viability and mammosphere formation and induce apoptosis in both BCa cells and BCSCs. At the molecular level, psoralidin inhibits Notch signaling, resulting in inhibition of EMT markers (β-catenin and vimentin) and upregulation of E-cadherin expression, leading to reduced migration and invasion [37]. This suggests that Notch may play a key role in the maintenance of cell viability by allowing cells to remain suspended in space.

In the evaluation of the stemness markers—CD24, CD44, and ALDH1—it was expected that CD44 and ALDH1 expressions would diminish following treatments, thereby leading to a reduction in the BCSC population. Curcumin treatment decreased CD44 and ALDH1 expression, and CD24 expression did not show an increase. The results for the CD24 marker can be explained by the fact that CD24 is not expressed or is expressed at very low levels in BCSCs, and therefore no significant change in its expression was expected. This result finds support in the work of Calaf et al., who reported no decrease in this surface marker using the MDA-MB-231 cell line when treated with curcumin [38]. Interestingly, the treatment with TL3–TL6 also diminished ALDH expression, and TL6 also reduced CD44 expression. The CD44^+^/CD24^low/−^ phenotype predominantly characterizes the mesenchymal subtype, typically localized in the tumor periphery, contributing to tumor metastasis [39]. In contrast, the overexpression of ALDH1 is linked to the proliferation and self-renewal of the BCSC population [35,40]. Hence, a plausible hypothesis arises: TL3, TL4, TL5, and TL6 might have inhibited the proliferation of this malignant population, while specifically TL6 reduced the mesenchymal phenotype. This implies that the compound TL6 might possess a dual impact on BCSCs. It should be noted that the inhibitory effects of the curcumin derivatives could occur through other pathways that were not characterized in this study, considering the multiple pathways altered in this malignant subpopulation [9,10,11]. These include, for instance, the JAK/STAT3 pathway, implicated in BCSC maintenance [41], or NF-Kβ, which is related to the invasive potential of BCa cells [23], among others.

Several studies have demonstrated that curcumin in combination with other chemotherapeutic agents allows for the sensitization of BCSCs and the reduction in the dosage of these chemotherapeutics [31,42]. Therefore, TL3 and TL4 were considered as potential compounds to be used in combination with other chemotherapeutic agents. Our findings revealed a synergic effect when TL3 was added in combination with doxorubicin and cisplatin. Curcumin derivatives are known to reduce the side effects of cisplatin because curcumin can inhibit inflammation by downregulating the NF-κB pathway [33]. Curcumin derivatives can also enhance the cytotoxicity of cisplatin against certain cancers, including breast cancer [42]. Moreover, several studies have shown that the co-administration of curcumin and doxorubicin sensitized MDA-MB-231 cells to chemotherapy, including treatment-resistant cells [43]. Thus, most of the results obtained with cisplatin and doxorubicin were consistent with those described in the literature. However, most of the findings obtained with the combination of paclitaxel exhibited an antagonistic effect. Other researchers observed that the combined administration of curcumin and paclitaxel potentiates the apoptotic effect in the breast cancer cell line MDA-MB-231 [44]. Hence, several parameters must be considered when evaluating the results obtained with paclitaxel. First, researchers that achieved synergism needed a concentration of curcumin higher than the IC_50_ determined in this study [45]. Hence, the administration of higher doses of paclitaxel, or even other taxanes such as docetaxel, should be studied to determine a possible synergistic effect. Finally, it is possible that these antagonistic results were caused by the low solubility of paclitaxel, which could decrease the solubility of TL3 and TL4 when administered together. So, as the results of this study, we should highlight the positive combination of TL3 and TL4 with doxorubicin and cisplatin and further study these drug combinations.

## 4. Materials and Methods

### 4.1. Chemistry

The analysis by thin layer chromatography (TLC) was performed on TLC plates precoated with silica gel 60 F254 (Merck, Darmstadt, Germany) and compounds were detected with UV light (at 254 nm). Purification by flash chromatography was carried out on silica gel 60 (0.040–0.063 mm, Merck). HPLC analyses were performed with a 1260 Infinity II (Agilent Technologies, Santa Clara, CA, USA) constituted by a 1260 Vial sampler, a Pump VL quaternary pump and a Diode Array HS detector using a reverse phase Kromasil 100 C_18_ column (3 μm, 4.6 mm × 40 mm), and a mobile phase constituted by H_2_O with 0.1% of TFA (solvent A) and CH_3_CN with 0.1% TFA (solvent B) and a flow rate of 1 mL/min. For the elution, a linear gradient from 2 to 100% was applied over 12 min, and the OpenLab CDS ChemStation software (Rev. C.01.07 SR4) was used for the control and analysis of the chromatograms. NMR experiments were acquired in the Serveis Tècnics de Recerca de la Universitat de Girona (STR-UdG) with an Ultrashield Avance III 400 (9.4T) spectrometer from Bruker (Billerica, MA, USA) (^1^H, 400 MHz; ^13^C, 100 MHz), equipped with an RT BBI probe and a temperature control unit (BCU Xtreme) or with an Ultrashield ASCEND Nanobay 400 (9.4T) from Bruker (^1^H, 400 MHz; ^13^C, 100 MHz). Structural assignments were made with gCOSY, gHSQC, and gHMBC experiments. NMR spectra were processed and analyzed using TopSpin 3.6.2. Chemical shifts were calibrated with the solvent signal and reported as δ (ppm). IR spectra were acquired with a Cary 630 FT-IR spectrophotometer (Agilent Technologies) equipped with a Golden Gate Single Reflection, ATR MK-II system, controlled by the MicroLabPC software (4.0), and spectra were analyzed with ResolutionsPro 5.2.0. The analysis by ESI-MS (STR-UdG) was carried out with an Esquire 6000 ESI Bruker ion Trap LC/MS equipped with an electrospray ion source operating in both positive and negative ion modes. Samples (5 μL) were introduced into the spectrometer through a 1200 Series Agilent HPLC autosampler. The mobile phase, CH_3_CN/H_2_O (80:20), was delivered by an Agilent 1200 Series HPLC pump at a flow rate of 0.1 mL/min. Nitrogen was employed as both a drying and nebulizing gas. HRMS (STR-UdG) were recorded with a Bruker MicroTOF-Q II instrument under conditions of ESI using a hybrid quadrupole time-of-flight mass spectrometer. Samples were introduced into the mass spectrometer ion source by direct infusion through a syringe pump and were externally calibrated using sodium formate. The melting point (mp) of the compounds was determined using a Melting Point SMP10 (Merck).

### 4.2. Synthesis of the Polyphenols TL1-TL6

#### 4.2.1. Synthesis of 3,4,5-tris(*tert*-butyldimethylsilyloxy)benzoic Acid [17]

Gallic acid (2.0 g, 11.75 mmol, 1 equiv) and *tert*-butyldimethylsilyl chloride (10.6 g, 70.5 mmol, 6 equiv) were dissolved in anhydrous *N*,*N*-dimethylformamide (DMF) (20 mL) under nitrogen. *N,N’*-Diisopropylethylamine (DIPEA) (14 mL, 81.8 mmol, 7 equiv) was added to the solution and the mixture was stirred overnight at room temperature under nitrogen. The crude reaction mixture turned from cloudy white to pale pink. The reaction was controlled by thin layer chromatography (TLC) (hexane/EtOAc 4:1). When the reaction was completed, cold 1 M H_3_PO_4_ (18 mL) was added to the crude, and extractions were carried out with hexane (3 × 60 mL). After washings of the organic layers with NaHCO_3_ (3 × 60 mL) and brine (3 × 60 mL), they were dried over anhydrous MgSO_4_ and filtered. The removal of the solvent under reduced pressured afforded a white solid, which was dissolved in tetrahydrofuran (THF)/H_2_O/acetic acid (1:1:3, 70 mL) and stirred at room temperature overnight. Then, cold H_2_O was added, and THF was evaporated under reduced pressure. The residue was extracted with hexane/diethyl ether (1:1, 2 × 140 mL) and the combined organic layers were washed with water (2 × 140 mL) and brine (2 × 140 mL), dried over anhydrous MgSO_4_, and then filtered. Removal of the solvent under reduced pressure yielded 3,4,5-tris(*tert*-butyldimethylsilyloxy)benzoic acid as a white solid (4.56 g, 76% yield), which was immediately used in the next step without purification. MW (C_19_H_18_O_5_): 512.9 g/mol. TLC (hexane/EtOAc 4:1): R*_f_* = 0.30. ^1^H-NMR (400 MHz, CDCl_3_) δ (ppm): 0.19 (s, 6H, (C*H*_3_)_2_SiO *para*), 0.28 (s, 12H, (C*H*_3_)_2_SiO *meta*), 0.98 (s, 18H, (C*H*_3_)_3_CSiO *met*a), 1.03 (s, 9H, (C*H*_3_)_3_CSiO *para*), 7.30 (s, 2H, H-2). ^13^C-NMR (100 MHz, CDCl_3_) δ (ppm): –3.9 (2CH_3_, (*C*H_3_)_2_SiO *para*), –3.6 (4CH_3_, (*C*H_3_)_2_SiO *meta*), 18.5 (1C, (CH_3_)_3_*C*SiO *para*), 18.8 (2C, (CH_3_)_3_*C*SiO *meta*), 26.1 (3CH_3_, (*C*H_3_)_3_CSiO *para*), 26.2 (6CH_3_, (*C*H_3_)_3_CSiO *meta*), 116.1 (2CH, C-2), 121.0 (C-1), 144.1 (C-4), 148.5 (2C, C-3), 171.8 (C=O). (–)-ESI-MS (*m*/*z*): 511.1 [M–H]^–^; (+)-ESI-MS (*m*/*z*): 513.4 [M+H]^+^.

#### 4.2.2. Synthesis of Alcohols **1** and **2**

##### Synthesis of 2,6-bis((*E*)-4-hydroxy-3-methoxybenzylidene)cyclohexan-1-one (**1**) [31]

Vanillin (1.0 g, 6.57 mmol, 2 equiv) was dissolved in EtOH (1.5 mL) at room temperature. Next, cyclohexanone (0.34 mL, 3.28 mmol, 1 equiv) and 37% HCl (66 μL, 0.79 mmol, 12 mol%) were added. The mixture was stirred for 72 h at room temperature. The reaction was monitored by TLC, and, once it was completed, EtOH was evaporated under reduced pressure. After the addition of cold H_2_O (30 mL), the crude was neutralized with aqueous 1% KOH, and a dark precipitate was obtained. After filtration, this solid was purified by *flash* chromatography using hexane/EtOAc (6:4), providing **1** as an orange solid (741 mg, 62% yield). MW (C_22_H_22_O_5_): 366.4 g/mol; TLC (hexane/EtOAc 1:2): R*_f_* = 0.72; HPLC (λ = 360 nm): *t*_R_ = 7.89 min (>99% purity); mp: 171–173 °C. FT-IR (ATR) ν (cm^–1^): 3350 (O–H), 1572 (Ar), 1122 (C–O). ^1^H-NMR (400 MHz, (CD_3_)_2_SO) δ (ppm): 1.72 (quint, *J* = 5.4 Hz, 2H, H-1), 2.88 (t, *J* = 5.4 Hz, 4H, H-2), 3.80 (s, 6H, OCH_3_), 6.85 (d, *J* = 8.4 Hz, 2H, H-9), 7.02 (dd, *J* = 8.4, *J’* = 1.4 Hz, 2H, H-10), 7.09 (d, *J* = 1.4 Hz, 2H, H-6), 7.55 (br, 2H, H-4). ^13^C-NMR (100 MHz, (CD_3_)_2_SO) δ (ppm): 22.6 (CH_2_, C-1), 28.0 (2CH_2_, C-2), 55.7 (2 OCH_3_), 114.8 (2CH, C-6), 115.6 (2CH, C-9), 124.3 (2CH, C-10), 127.0 (2C, C-8), 133.6 (2C, C-3), 136.2 (2CH, C-4), 147.5 (2C, C-7), 147.9 (2C, C-5), 188.7 (C=O); (–)-ESI-MS (*m*/*z*): 365.0 [M–H]^–^; (+)-ESI-MS (*m*/*z*): 367.2 [M+H]^+^.

##### Synthesis of (1*E*,4*E*)-1,5-bis(4-hydroxy-3-methoxyphenyl)penta-1,4-dien-3-one (**2**) [32]

Vanillin (1.0 g, 6.57 mmol, 2 equiv) was dissolved in EtOH (3 mL) at room temperature. To the resulting solution, acetone (0.244 mL, 3.28 mmol, 1 equiv) and 37% HCl (66 μL, 0.79 mmol, 12 mol%) were added. The mixture was heated to reflux for 5 h, and the reaction was monitored by TLC. Once the reaction was completed, EtOH was evaporated under reduced pressure. After the addition of cold H_2_O (30 mL), the crude was neutralized with aqueous 1% KOH, followed by extractions with CH_2_Cl_2_ (3 × 40 mL). The combined organic layers were washed with brine (2 × 60 mL), dried over anhydrous MgSO_4_, and then filtered. CH_2_Cl_2_ was evaporated under reduced pressure to provide a viscous black oil which was purified by *flash* chromatography (hexane/EtOAc 6:4), affording **2** as a dark green solid (397 mg, 37% yield). MW (C_19_H_18_O_5_): 326.1 g/mol; TLC (hexane/EtOAc 1:2): R*_f_* = 0.68; HPLC (λ = 360 nm): *t_R_* = 6.98 min (>99% purity); mp: 78–80 °C. FT-IR (ATR) ν (cm^–1^): 3312 (O–H), 1719 (C=O), 1581 (Ar), 1263 (C–O), 1155 (C–O). ^1^H-NMR (400 MHz, (CD_3_)_2_CO) δ (ppm): 3.93 (s, 6H, OCH_3_), 6.89 (d, *J* = 8.0 Hz, 2H, H-7), 7.11 (d, *J* = 16.0 Hz, 2H, H-1), 7.23 (dd, *J* = 8.0 Hz, *J’* = 2.0 Hz, 2H, H-8), 7.38 (d, *J* = 2.0 Hz, 2H, H-4), 7.67 (d, *J* = 16.0 Hz, 2H, H-2), 8.13 (s, 2H, OH). ^13^C-NMR (100 MHz, (CD_3_)_2_CO) δ (ppm): 56.4 (2 OCH_3_), 111.6 (2CH, C-4), 116.2 (2CH, C-7), 124.1 (2CH, C-8), 124.2 (2CH, C-1), 128.2 (2C, C-3), 143.3 (2CH, C-2), 148.8 (2C, C-5), 150.1 (2C, C_6_), 188.6 (C=O). (–)-ESI-MS (*m*/*z*): 324.9 [M–H]^–^; (+)-ESI-MS (*m*/*z*): 327.2 [M+H]^+^.

#### 4.2.3. General Procedure for the Esterification

3,4,5-Tris(*tert*-butyldimethylsilyloxy)benzoic acid (1 equiv) was dissolved in anhydrous toluene (10 mL) at room temperature under nitrogen, followed by the addition of anhydrous DMF (20 mol%). The mixture was heated at 50 °C and, afterwards, a solution of (COCl)_2_ (3 equiv) in anhydrous toluene (2 mL) was added dropwise. The mixture was stirred at 50 °C for 1 h, and then the reaction was cooled to room temperature and the solvent removed under reduced pressure to afford 3,4,5-tris(*tert*-butyldimethylsilyloxy)benzoyl chloride as a crystalline yellow solid, which was immediately used without further purification.

3,4,5-Tris(*tert*-butyldimethylsilyloxy)benzoyl chloride (4 or 6 equiv) was dissolved in anhydrous THF (8 mL) under nitrogen at room temperature, and a solution of the corresponding alcohol (**1**–**4**, curcumin) (1 equiv) and triethylamine (8 equiv) in anhydrous THF (2 mL) was added dropwise. The mixture was stirred under nitrogen overnight at room temperature. Once the reaction was completed, as checked by TLC monitoring, THF was removed under reduced pressure and the residue was dissolved in CH_2_Cl_2_ (30 mL), and then washed with saturated NaHCO_3_ (3 × 30 mL) and H_2_O (3 × 30 mL). Then, the combined organic layers were dried over anhydrous MgSO_4_, and the solvent was removed under reduced pressure. The resulting residue was purified by *flash* chromatography using mixtures of hexane/EtOAc of increasing polarity. The corresponding *tert*-butyldimethylsilyl (TBDMS)-protected TL1-TL6 derivatives were obtained in yields ranging from 51 to 84%.

##### ((1*E*,1′*E*)-(2-Oxocyclohexane-1,3-diylidene)bis(methanylylidene))bis(2-methoxy-4,1-phenylene) bis[3,4,5-tris((tert-butyldimethylsilyl)oxy)benzoate]

This compound was prepared following the general procedure for the esterification step described above, using 2,6-bis((*E*)-4-hydroxy-3-methoxybenzylidene)cyclohexan-1-one (**1**) (178 mg, 0.48 mmol) and 3,4,5-tris(*tert*-butyldimethylsilyloxy)benzoyl chloride (4 equiv). Final purification by *flash* chromatography using hexane/EtOAc (92:8), afforded the expected TBDMS-protected diester as a yellow solid (368 mg, 56% yield). MW (C_72_H_114_O_13_Si_6_): 1356.2 g/mol; TLC (hexane/EtOAc 4:1): R*_f_* = 0.85; mp: 103–106 °C. FT-IR (ATR) ν (cm^–1^): 2928 (CH_2_), 2855 (CH_3_), 1742 (C=O), 1572 (Ar), 1190 (C–O). ^1^H-NMR (400 MHz, (CD_3_)_2_CO) δ (ppm): 0.23 (s, 12H, (C*H*_3_)_2_SiO *para*), 0.32 (s, 24H, (C*H*_3_)_2_SiO *meta*), 1.00 (s, 36H, (C*H*_3_)_3_CSiO *meta*), 1.06 (s, 18H, (C*H*_3_)_3_CSiO *para*), 1.85 (quint, *J* = 6.0 Hz, 2H, H-1), 3.03 (t, *J* = 6.0 Hz, 4H, H-2), 3.90 (s, 6H, OCH_3_), 7.21 (dd, *J* = 8.2, *J’* = 1.4 Hz, 2H, H-10), 7.30 (d, *J* = 1.4 Hz, 2H, H-6), 7.31 (d, *J* = 8.2 Hz, 2H, H-9), 7.44 (s, 4H, H-2′), 7.72 (br, 2H, H-4). ^13^C-NMR (100 MHz, (CD_3_)_2_CO) δ (ppm): –3.5 (4CH_3_, (*C*H_3_)_2_SiO *para*), –3.3 (8CH_3_, (*C*H_3_)_2_SiO *meta*), 19.2 (2C, (CH_3_)_3_*C*SiO *para*), 19.5 (4C, (CH_3_)_3_*C*SiO *meta*), 23.7 (CH_2_, C-1), 26.6 (12CH_3_, (CH_3_)_3_CSiO *meta*), 26.6 (6CH_3_, (CH_3_)_3_CSi *para*), 29.2 (2CH_2_, C-2), 56.5 (2 OCH_3_), 115.6 (2CH, C-6), 116.9 (4CH, C-2′), 122.4 (2C, C-1′), 123.5 (2CH, C-10), 124.0 (2CH, C-9), 135.7 (2C, C-5), 136.3 (2CH, C-4), 137.5 (2C, C-3), 141.4 (2C, C-8), 144.5 (2C, C-4′), 149.6 (4C, C-3′), 152.3 (2C, C-7), 164.4 (2 O=C–O), 189.5 (C=O). ESI-MS (*m*/*z*): 1373.8 [M+NH_4_]^+^; ESI-HRMS (*m*/*z*): calcd. for C_72_H_114_O_13_Si_6_Na [M+Na]^+^ 1377.6767, found 1377.6846.

##### ((1*E*,4*E*)-3-Oxopenta-1,4-diene-1,5-diyl)bis(2-methoxy-4,1-phenylene) bis[3,4,5-tris((*tert*-butyldimethylsilyl)oxy)benzoate]

This compound was prepared following the general procedure for the esterification step described above using (1*E*,4*E*)-1,5-bis(4-hydroxy-3-methoxyphenyl)penta-1,4-dien-3-one (**2**) (88 mg, 0.27 mmol) and 3,4,5-tris(*tert*-butyldimethylsilyloxy)benzoyl chloride (4 equiv). Final purification by *flash* chromatography using hexane/EtOAc (92:8), afforded the expected TBDMS-protected diester as an orange solid (201 mg, 57% yield). MW (C_69_H_110_O_13_Si_6_): 1316.4 g/mol; TLC (hexane/EtOAc 4:1): R*_f_* = 0.35. mp: 219–221 °C. FT-IR (ATR) ν (cm^–1^): 2854 (CH_3_), 1731 (C=O), 1577 (Ar), 1188 (C–O). ^1^H-NMR(400 MHz, CDCl_3_) δ (ppm): 0.17 (s, 12H, (C*H*_3_)_2_SiO *para*), 0.26 (s, 24H, (C*H*_3_)_2_SiO *meta*), 0.96 (s, 36H, (C*H*_3_)_3_CSiO *meta*), 1.01 (s, 18H, (C*H*_3_)_3_CSiO *para*), 3.89 (s, 6H, OCH_3_), 7.05 (d, *J* = 16.0 Hz, 2H, H-1), 7.23–7.27 (m, 6H, H-4, H-7 and H-8), 7.38 (s, 4H, H-2′), 7.73 (d, *J* = 16.0 Hz, 2H, H-2). ^13^C-NMR (100 MHz, CDCl_3_) δ (ppm): –3.7 (8CH_3_, (*C*H_3_)_2_SiO *meta*), –3.5 (4CH_3_, (*C*H_3_)_2_SiO *para*), 18.7 (2C, (CH_3_)_3_*C*SiO *para*), 19.0 (4C, (CH_3_)_3_*C*SiO *meta*), 26.3 (18CH_3_, (*C*H_3_)_3_CSiO *para* and (*C*H_3_)_3_CSiO *meta*)), 56.2 (2 OCH_3_), 111.9 (2CH, C-4), 116.4 (4CH, C-2′), 120.9 (2C, C-1′), 121.6 (2CH, C-8), 123.7 (2CH, C-7),125.6 (2CH, C-1), 133.6 (2C, C-3), 142.4 (2C, C-6), 143.0 (2CH, C-2), 144.1 (2C, C-4′), 148.7 (4C, C-3′), 152.0 (2C, C-5), 164.4 (2 O=C–O), 188.8 (C=O). ESI-MS (*m*/*z*): 1338.7 [M+Na]^+^; ESI-HRMS (*m*/*z*): calcd. for C_69_H_110_O_13_Si_6_Na [M+Na]^+^ 1337.6454, found 1337.6486.

##### 1,4-Phenylenebis(methylene) bis[3,4,5-tris((*tert*-butyldimethylsilyl)oxy)benzoate]

This compound was prepared following the general procedure for the esterification step described above using 1,4-benzenedimethanol (**3**) (81 mg, 0.58 mmol) and 3,4,5-tris(*tert*-butyldimethylsilyloxy)benzoyl chloride (4 equiv). Final purification by *flash* chromatography using (hexane/EtOAc 98:2) afforded the expected TBDMS-protected diester as a white solid (558 mg, 84% yield). MW (C_58_H_102_O_10_Si_6_): 1127.9 g/mol; TLC (Hexane/EtOAc 4:1): R*_f_* = 0.85. FT-IR (ATR) ν (cm^–1^): 2928 (CH_2_), 2855 (CH_3_), 1714 (C=O), 1572 (Ar), 1077 (C–O). ^1^H-NMR (400 MHz, CDCl_3_) δ (ppm): 0.13 (s, 12H, (C*H*_3_)_2_SiO *para*), 0.23 (s, 24H, (C*H*_3_)_2_SiO *meta*), 0.94 (s, 36H, (C*H*_3_)_3_CSiO *meta*), 0.98 (s, 18H, (C*H*_3_)_3_CSiO *para*), 5.31 (s, 4H, H-3), 7.26 (s, 4H, H-2′), 7.43 (s, 4H, H-1). ^13^C-NMR (100 MHz, CDCl_3_) δ (ppm): –3.9 (4CH_3_, (*C*H_3_)_2_SiO *para*), –3.6 (8CH_3_, (*C*H_3_)_2_SiO *meta*), 18.5 (2C, (CH_3_)_3_*C*SiO *para*), 18.8 (4C, (CH_3_)_3_*C*SiO *meta*), 26.1 (6CH_3_, (*C*H_3_)_3_CSiO *para*), 26.2 (12CH_3_, (*C*H_3_)_3_CSiO *meta*), 65.9 (2CH_2_, C-3), 115.6 (4CH, C-2′), 121.8 (2C, C-1′), 127.8 (4CH, C-1), 136.2 (2C, C-2), 143.4 (2C, C-4′), 148.5 (4C, C-3′), 166.0 (2 O=C–O). ESI-MS (*m*/*z*): 1127.6 [M+Na]^+^; ESI-HRMS (*m*/*z*): calcd. for C_58_H_102_O_10_Si_6_Na [M+Na]^+^ 1149.5981, found 1149.5988.

##### Benzene-1,3,5-triyl tris[3,4,5-tris((*tert*-butyldimethylsilyl)oxy)benzoate]

This compound was prepared following the general procedure described above for the esterification step using 1,3,5-trihydroxybenzene (**4**) (49 mg, 0.39 mmol) and 3,4,5-tris(*tert*-butyldimethylsilyloxy)benzoyl chloride (6 equiv). Final purification by *flash* chromatography using (hexane/EtOAc 98:2) afforded the expected TBDMS-protected triester as a white solid (473 mg, 75% yield). MW (C_81_H_144_O_15_Si_9_): 1608.8 g/mol; TLC (hexane/EtOAc 4:1): R*_f_* = 0.85. FT-IR (ATR) ν (cm^–1^): 2928 (CH_2_), 2856 (CH_3_), 1738 (C=O), 1573 (Ar), 1183 (C–O). ^1^H-NMR (400 MHz, CDCl_3_) δ (ppm): 0.16 (s, 18H, (C*H*_3_)_2_SiO *para*), 0.25 (s, 36H, (C*H*_3_)_2_SiO *meta*), 0.96 (s, 54H, (C*H*_3_)_3_CSiO *meta*), 1.00 (s, 27H, (C*H*_3_)_3_CSiO *para*), 7.06 (s, 3H, H-1), 7.35 (s, 6H, H-2′). ^13^C-NMR (100 MHz, CDCl_3_) δ (ppm): –3.9 (6CH_3_, (*C*H_3_)_2_SiO *para*), –3.6 (12CH_3_, (*C*H_3_)_2_SiO *meta*), 18.5 (3C, (CH_3_)_3_*C*SiO *para*), 18.8 (6C, (CH_3_)_3_*C*SiO *meta*), 26.1 (9CH_3_, (*C*H_3_)_3_CSiO *para*), 26.2 (18CH_3_, (*C*H_3_)_3_CSiO *meta*), 113.4 (3CH, C-1), 116.1 (6CH, C-2′), 120.7 (3C, C-1′), 144.2 (3C, C-4′), 148.7 (6C, C-3′), 151.7 (3C, C-2), 164.2 (3 O=C–O). ESI-MS (*m*/*z*): 1609.8 [M+H]^+^; ESI-HRMS (*m*/*z*): calcd. for C_81_H_144_O_15_Si_9_Na [M+Na]^+^ 1631.8321, found 1631.8330.

##### ((1*E*,3*Z*,6*E*)-3-Hydroxy-5-oxohepta-1,3,6-triene-1,7-diyliden)bis(2-methoxy-4,1-phenylene) bis[3,4,5-tris((*tert*-butyldimethylsilyl)oxy)benzoate]

This compound was prepared following the general procedure described above for the esterification step using curcumin (215 mg, 0.59 mmol) and 3,4,5-tris(*tert*-butyldimethylsilyloxy)benzoyl chloride (4 equiv). Final purification by dry column vacuum chromatography using hexane/EtOAc (92:8) afforded the expected TBDMS-protected diester as a brown solid (407 mg, 51% yield). MW (C_71_H_112_O_14_Si_6_): 1356.7 g/mol; TLC (hexane/EtOAc 4:1) R*_f_* = 0.60. FT-IR (ATR) ν (cm^–1^): 2913 (CH_2_), 2847 (CH_3_), 1732 (C=O), 1574 (Ar), 1192 (C–O). ^1^H-NMR (400 MHz, CDCl_3_) δ (ppm): 0.17 (s, 12H, (C*H*_3_)_2_SiO *para*), 0.26 (s, 24H, (C*H*_3_)_2_SiO *meta*), 0.96 (s, 36H, (C*H*_3_)_3_CSiO *meta*), 1.01 (s, 18H, (C*H*_3_)_3_CSiO *para*), 3.88 (s, 6H, CH_3_O), 5.89 (s, 1H, H-1), 6.60 (d, *J* = 16.0 Hz, 2H, H-3), 7.16-7.26 (m, 6H, H-6, H-9 and H-10), 7.48 (s, 4H, H-2′), 7.66 (d, *J* = 16.0 Hz, 2H, H-4). ^13^C-NMR (100 MHz, CDCl_3_) δ (ppm): –3.9 (4CH_3_, (*C*H_3_)_2_SiO *para*), –3.6 (8CH_3_, (*C*H_3_)_2_SiO *meta*), 18.5 (2C, (CH_3_)_3_*C*SiO *para*), 18.8 (4C, (CH_3_)_3_*C*SiO *meta*), 26.1 (6CH_3_, (*C*H_3_)_3_CSiO *para*), 26.2 (12CH_3_, (*C*H_3_)_3_*C*SiO *meta*), 56.0 (2 OCH_3_), 101.8 (CH, C-1), 111.6 (2CH, C-6), 116.3 (4CH, C_2-gal_), 120.8 (2C, C-1′), 121.1 (2CH, C-10), 123.5 (2CH, C-9), 124.1 (2CH, C-3), 133.7 (2C, C_5_), 140.1 (2CH, C_4_), 141.9 (2C, C_8_), 144.0 (2C, C-4′), 148.6 (4C, C-3′), 151.8 (2C, C-7), 164.3 (2 O=C–O), 183.2 (2C, C-2). ESI-MS (*m*/*z*): 1357.6 [M+H]^+^; ESI-HRMS (*m*/*z*): calcd. for C_71_H_112_O_14_Si_6_Na [M+Na]^+^ = 1379.6560, found 1379.6569.

#### 4.2.4. General Procedure for the Removal of the *tert*-Butyldimethylsilyl (TBDMS) Group

The corresponding TBDMS-protected TL1–TL6 derivative was dissolved in anhydrous THF under nitrogen. A solution of 1 M TBAF (7 or 10 equiv) in anhydrous THF was added and the reaction mixture was stirred at room temperature under nitrogen for 15 min. The progress of the reaction was monitored by TLC and, upon completion, the crude was concentrated under reduced pressure to approximately half its volume. Then, MeOH was added and the resulting solution was treated with a *Dowex 50WX8-200* ionic exchange resin in the presence of CaCO_3_. The mixture was stirred at room temperature for 2 h. Subsequently, the resin was filtered and the solvent was evaporated under reduced pressure. The resulting residue was purified by reversed-phase column chromatography using a CombiFlash instrument. TL1–TL6 were obtained in yields ranging from 37 to 83%.

##### 1,4-Phenylenebis(methylene) bis(3,4,5-trihydroxybenzoate) (TL1)

This compound was prepared following the general procedure described above, starting from the corresponding TBDMS-protected diester (558 mg, 0.50 mmol) using 7 equiv. of 1 M TBAF. Final purification eluting with H_2_O/CH_3_CN (67:33) afforded the diester TL1 as a white solid (112 mg, 51% yield). MW (C_22_H_18_O_10_): 442.4 g/mol; TLC (CH_2_Cl_2_/MeOH/AcOH 8:2:0.5): R*_f_* = 0.54; UV-Vis (CH_3_CN) $\lambda_{max}$= 225 and 273 nm; HPLC (λ = 220 nm) t_R_ = 6.02 min (>99% purity); mp: 213–216 °C. FT-IR (ATR) ν (cm^–1^): 3332 (O–H), 1668 (C=O), 1611 (Ar), 1255 (C–O). ^1^H-NMR (400 MHz, CD_3_OD) δ (ppm): 5.29 (s, 4H, H-3), 7.08 (s, 4H, H-2′), 7.45 (s, 4H, H-1). ^13^C-NMR (100 MHz, CD_3_OD) δ (ppm): 67.0 (2CH_2_, C-3), 110.1 (4CH, C-2′), 121.4 (2C, C-1′), 129.1 (4CH, C-1), 137.9 (2C, C-2), 139.9 (2C, C-4′), 146.5 (4C, C-3′), 168.2 (2 O=C–O). (–)-ESI-MS (*m*/*z*): 440.8 [M–H]^–^. (–)-ESI-HRMS (*m*/*z*): calcd. for C_22_H_17_O_10_ [M–H]^–^ 441.0827, found 441.0816; calcd. for C_22_H_16_O_10_ [M–2H]^2–^ 220.0377, found 220.0362.

##### Benzene-1,3,5-triyl tris(3,4,5-trihydroxybenzoate) (TL2)

This compound was prepared following the general procedure described above, starting from the corresponding TBDMS-protected triester (377 mg, 0.23 mmol) and using 10 equiv of 1 M TBAF. Final purification eluting with H_2_O/CH_3_CN (75:25) afforded the diester TL2 as a white solid (95.2 mg, 70% yield). MW (C_27_H_18_O_15_): 582.4 g/mol; TLC (CH_2_Cl_2_/MeOH/AcOH 8:2:0.5): R*_f_* = 0.25; HPLC (λ = 220 nm) *t_R_* = 5.25 min (99% purity); mp: 296–298 °C. FT-IR (ATR) ν (cm^–1^): 3402 (O–H), 1719 (C=O), 1614 (Ar), 1198 (C–O). ^1^H-NMR (400 MHz, CD_3_OD) δ (ppm): 7.03 (s, 3H, H-1), 7.21 (s, 6H, H-2′). ^13^C-NMR (100 MHz, CD_3_OD) δ (ppm): 110.7 (6CH, C-2′), 114.5 (3CH, C-1), 120.1 (3C, C-1′), 140.8 (3C, C-4′), 146.7 (6C, C-3′), 153.3 (3C, C-2), 166.3 (3 O=C–O). (–)-ESI-MS (*m*/*z*): 580.8 [M–H]^–^. (–)-ESI-HRMS (*m*/*z*): calcd. for C_27_H_17_O_15_ [M–H]^–^ 581.0573, found 581.0549; calcd. for C_27_H_16_O_15_ [M–2H]^2–^ 290.0250, found 290.0235.

##### 1,4-Phenylenebis(methylene) bis(3,4,5-trihydroxybenzoate) (TL3)

This compound was prepared following the general procedure described above, starting from the corresponding TBDMS-protected diester (381 mg, 0.28 mmol) and using 7 equiv of 1 M TBAF. Final purification eluting with H_2_O/CH_3_CN (50:50) afforded the diester TL3 as a yellow solid (156 mg, 83% yield). MW (C_35_H_28_O_14_): 672.2 g/mol; TLC (CH_2_Cl_2_/MeOH/AcOH 8:2:0.5): R*_f_* = 0.73; UV-Vis (CH_3_CN) $\lambda_{max\;}$= 226, 278 and 402 nm; HPLC (λ = 220 nm) *t_R_* = 8.14 min (98% purity); mp: 205–206 °C; FT-IR (ATR) ν (cm^–1^): 3360 (O–H), 1683 (C=O), 1505 (Ar), 1189 (C–O). ^1^H-NMR (400 MHz, (CD_3_)_2_CO) δ (ppm): 3.89 (s, 6H, CH_3_O), 6.12 (s, 1H, H-1), 6.91 (d, *J* = 16.0 Hz, 2H, H-3), 7.24 (d, *J* = 8.0 Hz, 2H, H-9), 7.28 (s, 4H, H-2′), 7.34 (dd, *J* = 8.0 Hz, *J’* = 1.6 Hz, 2H, H-10), 7.50 (d, *J* = 1.6 Hz, 2H, H-6), 7.70 (d, *J* = 16.0 Hz, 2H, H-4). ^13^C-NMR (100 MHz, (CD_3_)_2_CO) δ (ppm): 56.4 (2 OCH_3_), 102.5 (CH, C-1), 110.5 (4CH, C-2′), 112.6 (2C, C-6), 120.6 (2C, C-1′), 122.1 (2CH, C-10), 124.5 (2CH, C-9), 125.3 (2CH, C-3), 134.8 (2C, C-5), 139.5 (2C, C-4′), 140.7 (2CH, C-4), 142.9 (2C, C-8), 146.2 (4C, C-3′), 153.0 (2C, C-7), 164.7 (2 O=C–O), 184.5 (2C, C-2). (–)-ESI-MS (*m*/*z*): 670.9 [M–H]^–^, (–)-ESI-HRMS (*m*/*z*): calcd. for C_35_H_27_O_14_ [M–H]^–^ 671.1406, found 671.1407; calcd. for C_35_H_26_O_14_ [M–2H]^2–^ 335.0667, found 335.0666.

##### ((1*E*,1′*E*)-(2-Oxocyclohexane-1,3-diylidene)bis(methanylylidene))bis(2-methoxy-4,1-phenylene) bis[3,4,5-tris((*tert*-butyldimethylsilyl)oxy)benzoate] (TL4) and ((1*E*,1′*Z*)-(2-oxocyclohexane-1,3-diylidene)bis(methanylylidene))bis(2-methoxy-4,1-phenylene) bis[3,4,5-tris((*tert*-butyldimethylsilyl)oxy)benzoate] (TL5)

This compound was prepared following the general procedure described above; starting from the corresponding TBDMS-protected diester (233 mg, 0.17 mmol) and using 7 equiv of 1 M TBAF afforded a mixture of isomers TL4 and TL5. Final purification eluting with H_2_O/CH_3_CN (52:48) allowed the isolation of these two isomers: TL4 as a yellow solid (52 mg, 45% yield) and diester TL5 as a yellow-orange solid (28 mg, 24% yield).

*Characterization of TL4*: MW (C_36_H_30_O_13_): 670.6 g/mol; TLC (CH_2_Cl_2_/MeOH/AcOH 8:2:0.5): R*_f_* = 0.50; UV-Vis (CH_3_CN) $\lambda_{max\;}$ = 217 and 283 nm; HPLC (λ = 220 nm) *t_R_* = 7.63 min (92% purity); mp: 204–206 °C. FT-IR (ATR) ν (cm^–1^): 3324 (O–H), 1724 (C=O), 1595 (Ar), 1187 (C–O). ^1^H-NMR (400 MHz, (CD_3_)_2_CO) δ (ppm): 1.85 (quint, *J* = 4.6 Hz, 2H, H-1), 3.03 (t, *J* = 4.6 Hz, 4H, H-2), 3.88 (s, 6H, OCH_3_), 7.20 (dd, *J* = 8.2, *J’* = 1.4 Hz, 2H, H-10), 7.25 (d, *J* = 8.2 Hz, 2H, H-9), 7.27 (d, *J* = 1.4 Hz, 2H, H-6), 7.28 (s, 4H, H-2′), 7.72 (br, 2H, H-4). ^13^C-NMR (100 MHz, (CD_3_)_2_CO) δ (ppm): 23.7 (CH_2_, C-1), 29.1 (2CH_2_, C-2), 56.4 (2 OCH_3_), 110.6 (4CH, C-2′), 115.6 (2CH, C-6), 120.7 (2C, C-1′), 123.6 (2CH, C-10), 124.0 (2CH, C-9), 135.6 (2C, C-5), 136.4 (2CH, C-4), 137.4 (2C, C-3), 139.5 (2C, C-4′), 141.6 (2C, C-8), 146.3 (4C, C-3′), 152.5 (2C, C-7), 164.8 (2 O=C-O), 189.5 (C=O). (–)-ESI-MS (*m*/*z*): 669.0 [M–H]^–^. (–)-ESI-HRMS (*m*/*z*): calcd. for C_36_H_29_O_13_ [M–H]^–^ 669.1603, found 669.1638; calcd. for C_36_H_28_O_13_ [M–2H]^2–^ 334.0759, found 334.0763. 

*Characterization of TL5*: MW (C_36_H_30_O_13_): 670.6 g/mol; TLC (CH_2_Cl_2_/MeOH/AcOH 8:2:0.5): R*_f_* = 0.53; UV-Vis (CH_3_CN) $\lambda_{max\;}$ = 216 and 280 nm; HPLC (λ = 220 nm) *t_R_* = 7.93 min (93% purity); mp: 127–129 °C. FT-IR (ATR) ν (cm^–1^): 3294 (O–H), 1717 (C=O), 1595 (Ar), 1186 (C–O). ^1^H-NMR (400 MHz, (CD_3_)_2_CO) δ (ppm): 1.96 (quint, *J* = 5.8 Hz, 2H, H-1), 2.80 (t, *J* = 5.8 Hz, 2H, H-2′), 3.01 (t, *J* = 5.8 Hz, 2H, H-2), 3.83 (s, 3H, 7′-OCH_3_), 3.86 (s, 3H, 7-OCH_3_), 6.75 (br, 1H, H-4′), 7.10 (d, *J* = 8.2 Hz, 1H, H-9′), 7.15 (dd, *J* = 7.0 Hz, *J’* = 1.6 Hz, 1H, H-10), 7.18 (dd, *J* = 8.6 Hz, *J’* = 1.6 Hz, 1H, H-10′), 7.22 (d, *J* = 7.0 Hz, 1H, H-9), 7.25 (d, *J* = 1.8 Hz, 1H, H-6), 7.27 (s, 4H, H_2-gal_ and H_2′-gal_), 7.48 (br, 1H, H-4), 7.51 (d, *J* = 1.6 Hz, 1H, H-6′). ^13^C-NMR (100 MHz, (CD_3_)_2_CO) δ (ppm): 25.3 (CH_2_, C-1), 29.4 (CH_2_, C-2), 36.2 (CH_2_, C-2′), 56.2 (7′-OCH_3_), 56.3 (7-OCH_3_), 110.5 (4CH, C_2-gal_ and C_2′-gal_), 114.9 (CH, C-6′), 115.5 (CH, C-6), 120.7 (C_1-gal_), 120.9 (C_1′-gal_), 123.2 (CH, C-9′), 123.4 (2CH, C-10 and C-10′), 124.0 (CH, C-9), 135.3 (CH, C-4), 135.6 (C-5), 135.8 (C-5′), 136.2 (CH, C-4′), 139.3 (3C, C_4-gal_, C_4′-gal_ and C-3′), 140.2 (C-3), 141.1 (C-8′), 141.5 (C-8), 146.2 (4C, C_3-gal_ and C_3′-gal_), 151.7 (C-7′), 152.4 (C-7), 164.8 (2 O=C-O), 192.9 (C=O). (–)-ESI-MS (*m*/*z*): 669.0 [M–H]^–^. (–)-ESI-HRMS (*m*/*z*): calcd. for C_36_H_29_O_13_ [M–H]^–^ 669.1603, found 669.1635; calcd. for C_36_H_28_O_13_ [M–2H]^2–^ 334.0759, found 334.0775.

##### ((1*E*,4*E*)-3-Oxopenta-1,4-diene-1,5-diyl)bis(2-methoxy-4,1-phenylene) Bis[3,4,5-tris((*tert*-butyldimethylsilyl)oxy)benzoate] (TL6)

This compound was prepared following the general procedure described above, starting from the corresponding TBDMS-protected diester (120 mg, 0.09 mmol) and using 7 equiv. of 1 M TBAF. Final purification eluting with H_2_O/CH_3_CN (58:42) afforded the diester TL6 as a yellow solid (21 mg, 37% yield). MW (C_33_H_26_O_13_): 630.6 g/mol; TLC (CH_2_Cl_2_/MeOH/AcOH 8:2:0.5): R*_f_*= 0.51; UV-Vis (CH_3_CN) $\lambda_{max\;}$ = 218, 295 and 351 nm; HPLC (λ = 220 nm) *t_R_* = 20.21 min (96% purity); mp: 198–201 °C. FT-IR (ATR) ν (cm^–1^): 3265 (O–H), 1700 (C=O), 1594 (Ar), 1179 (C–O). ^1^H-NMR (400 MHz, CD_3_OD) δ (ppm): 3.89 (s, 6H, OCH_3_), 7.18 (d, *J* = 8.4 Hz, 2H, H-7), 7.20 (s, 4H, H-2′), 7.29 (d, *J* = 16.0 Hz, 2H, H-1), 7.36 (dd, *J* = 8.4, *J’* = 1.2 Hz, 2H, H-8), 7.47 (d, *J* = 1.2 Hz, 2H, H-4), 7.64 (d, *J* = 16.0 Hz, 2H, H-2). ^13^C-NMR (100 MHz, CD_3_OD) δ (ppm): 56.6 (2 OCH_3_), 110.7 (4CH, C-2′), 113.1 (2CH, C-4), 120.3 (2C, C-1′), 122.9 (2CH, C-8), 124.6 (2CH, C-7), 126.6 (2CH, C-1), 135.2 (2C, C-3), 140.6 (2C, C-4′), 143.7 (2C, C-6), 144.6 (2CH, C-2), 146.7 (4C, C-3′), 153.4 (2C, C-5), 166.4 (2 O=C–O), 191.3 (C=O). (–)-ESI-MS (*m*/*z*): 628.9 [M–H]^–^. (–)-ESI-HRMS (*m*/*z*): calcd. for C_33_H_25_O_13_ [M–H]^–^ 629.1290, found 629.1326; calcd. for C_33_H_24_O_13_ [M–2H]^2–^ 314.0612, found 314.0603.

### 4.3. Biological Evaluation

#### 4.3.1. Cell Culture

The MDA-MB-231 TNBC cell line, a mesenchymal stem-like subtype, was obtained from the American Type Culture Collection (ATCC, Manassas, VA, USA) and maintained in Dulbecco’s modified Eagle’s medium (DMEM) (Corning^®^, Corning, NY, USA), supplemented with 10% heat-inactivated fetal bovine serum (FBS) (HyClone, Logan, UT, USA) and 50 U/mL Pen/Strep (Lonza^®^, Basel, Switzerland). The 184B5 breast epithelial cell line was obtained from ATCC and maintained in mammary epithelial cell growth basal medium (MEBM) (Lonza), supplemented with 1 ng/mL cholera toxin (Sigma-Aldrich Co., St. Louis, MA, USA). MEBM-supplemented medium was maintained in the dark. Cells were incubated at 37 °C in a humidified environment with 5% CO_2_ and were maintained mycoplasma-free.

#### 4.3.2. Dilution of Polyphenolic Compounds

The polyphenolic compounds were dissolved in dimethyl sulfoxide (DMSO, Sigma-Aldrich) to prepare a master stock solution at a concentration of 100 ng/mL that was stored at −20 °C prior to use. The working solution was freshly prepared in complete culture medium.

#### 4.3.3. Cytotoxicity and Drug Combination Assay

MDA-MB-231 cells were seeded at a density of 4 × 10^3^ cells/well in their respective growth media in a 96-well microplate. Following overnight attachment, the culture medium was removed, and 200 µL of fresh medium containing increasing doses of the polyphenolic compound was added to each well. After 72 h of treatment, a colorimetric MTT assay (Sigma-Aldrich Co.) was used to measure cell viability as described elsewhere [46]. The percentage of cell viability was calculated based on the absorbance ratio between the cell culture treated with the compound and the untreated control multiplied by 100. Graph plots were used to determine the inhibitory concentrations.

For drug combination experiments, cells were treated with doxorubicin, cisplatin, or paclitaxel (provided by pharmacy service from Hospital Josep Trueta, Girona), at the IC_30_ in combination with a series of increasing concentrations of TL3 or TL4 for 72 h. After treatment, cell viability was measured using the MTT assay. Combinatorial effects were evaluated using the Combination Index (CI), which was calculated using CompuSyn^TM^ software V1.0 (Biosoft, Orlando, FL, USA) based on the Chou and Talalay method [47]. CompuSyn^TM^ calculates the CI values; if the value equals 1, the effect is considered additive; if it is above 1, it is considered antagonistic; and if it is below 1, it is considered synergistic.

#### 4.3.4. Mammosphere-Forming Assay

MDA-MB-231 cells were seeded into a non-adherent 6-well cell culture plate (Sarstedt, Nümbrecht, Germany) and incubated for 7 days at 37 °C in 5% CO_2_. Previously, the plates were coated with 1 mL/well pHEMA (1.02 g/L, Sigma-Aldrich Co.). Cells were cultured in DMEM/F12 medium (HyClone) supplemented with 2% B27 (Gibco, New York, NY, USA), 0.02% hEGF, 0.02% hFGF (20 ng/mL; Miltenyi Biotech, Bergich Gladbach, Germany), 1% L-glutamine, and 1% sodium pyruvate (Gibco, NY, USA). For the treatment conditions, compounds were added at an IC_30_ concentration at the time of seeding. After incubation, mammospheres with a minimum diameter of 50 μm were counted under an inverted optical microscope. The following parameters were calculated using the formulas described below: (A) Mammosphere-Forming Index (MFI) and (B) Mammosphere-Forming inhibition (MFIn).

$$MFI=\left(\frac{Nº\;mammosphere}{Nº\;cells\;plated}\right)\cdot 100\;\;(A)\;\;\;MFIn=\left(\frac{{Nº\;mammosphere}_{treatment}\cdot 100}{{Nº\;mammosphere}_{control}\cdot 10}\right)\;(B)\;$$

#### 4.3.5. Western Blot

For the analysis of TL1–TL6, cells were treated with IC_50_ for 72 h, with a previous 24 h untreated incubation period. Afterward, attached and floating cells were harvested and lysed in ice-cold lysis buffer (Cell Signaling Technology, Inc., Danvers, MA, USA) containing 100 µg/mL phenylmethylsulfonylfluoride (PMSF) by vortexing every 5 min for 30 min. Equal amounts of protein from cell lysates were heated in lithium dodecyl sulfate (LDS) Sample Buffer with Sample Reducing Agent (Invitrogen, Waltham, MA, USA) at 70 °C for 10 min, resolved by SDS-PAGE, and transferred to nitrocellulose membranes (Thermo Scientific Inc., New York, NY, USA). Membranes were incubated at room temperature in blocking buffer (5% skim milk powder in TBS 0.05% Tween [TBS-T]) for 1 h or in blocking buffer (5% bovine serum albumin (BSA) TBS 0.05% Tween [TBS-T]) for 3 h and overnight at 4 °C with the following primary antibodies diluted in the blocking buffer (see Supplementary Table S1). Specific horseradish peroxidase (HRP)-conjugated secondary antibodies were added and incubated for 1 h at room temperature. Immune complexes were detected using the chemiluminescent HRP substrate Clarity^TM^ Western ECL Substrate (Bio-Rad Laboratories, Hercules, CA, USA) or SuperSignal™ West Femto (Thermo Fisher Scientific) and a ChemiDoc™ Imaging System (Bio-Rad Laboratories, Inc.). Results were quantified using ImageLab software for Mac Version 6.1 (Bio-Rad Laboratories, Inc.). Western blot analyses were repeated at least three times.

#### 4.3.6. Statistical Analysis

All data are expressed as mean ± standard deviation of the mean (SD) of at least three independent experiments. Data analysis was performed using GraphPad Prism version 9.5.1 for Mac, GraphPad Software Prism 8.0.2 (La Jolla, CA, USA). Student’s *t*-test was employed to compare two groups and ANOVA for more than two groups with a Tukey post-test. Non-normally distributed data were analyzed by a non-parametric test (Kurskal–Wallis), while non-heteroscedasticity data were analyzed by ANOVA followed by a Thamane post-test. Significance levels are denoted as follows: *: *p* < 0.05; **: *p* < 0.01; and ***: *p* < 0.001.

## 5. Conclusions

This study demonstrated that the use of curcumin-derivative compounds is a valuable strategy for targeting BCSCs. Specifically, TL3, TL4, TL5, and TL6 exhibited a significantly enhanced cytotoxic capacity in in vitro assays using the MDA-MB-231 cell line, compared to curcumin. Notably, compounds TL3 and TL4 showed particularly promising results. In the latter case, the IC_50_ value was 20 times lower than that of the parent compound and significantly lower than that of TL5 and TL6. Moreover, neither of the two compounds demonstrated specific cytotoxicity in the non-tumorigenic cells. Furthermore, novel compounds TL3–TL6 significantly inhibited mammosphere formation, the Notch signaling pathway, and the stemness marker ALDH1, indicating their potential to target BCSC subpopulations. Lastly, the combination of TL3 with doxorubicin or cisplatin exhibited a synergistic effect, suggesting its potential for sensitizing BCSCs to chemotherapeutics. Nevertheless, further studies are required to determine the extent of these synergistic effects for potential clinical applications. Therefore, our findings provide a basis for further research using these curcumin derivatives, either alone or in combination with chemotherapeutic agents, to improve the treatment for TNBC patients.

## Figures and Tables

**Table 1:** IC_50_ and IC_30_ obtained for G28, curcumin, and TL1–TL6 in MDA-MB-231 cells. Significant differences with regard to curcumin treatment are indicated as *** (*p* < 0.001).

Compound	IC_50_ (μM)	IC_30_ (μM)
G28	34.77 ± 0.70	19.48 ± 2.02
Curcumin	17.36 ± 0.33	14.45 ± 0.01
TL1	18.02 ± 0.74	8.02 ± 1.23
TL2	32.03 ± 2.26	20.87 ± 1.12
TL3	0.93 ± 0.03 (***)	0.76 ± 0.03
TL4	0.86 ± 0.08 (***)	0.65 ± 0.09
TL5	1.31 ± 0.17 (***)	0.80 ± 0.07
TL5	3.58 ± 0.29 (***)	1.86 ± 0.36

## Data Availability

The data used to support the findings of this study are available from the corresponding authors upon request.

## Supplementary Materials

The following supporting information can be downloaded at: https://www.mdpi.com/article/10.3390/ijms25137446/s1.

## References

[1] Siegel R.L., Miller K.D., Fuchs H.E., Jemal A. (2022). Cancer Statistics, 2022. CA Cancer J. Clin..

[2] Harbeck N., Penault-Llorca F., Cortes J., Gnant M., Houssami N., Poortmans P., Ruddy K., Tsang J., Cardoso F. (2019). Breast Cancer. Nat. Rev. Dis. Prim..

[3] Vagia E., Mahalingam D., Cristofanilli M. (2020). The Landscape of Targeted Therapies in TNBC. Cancers.

[4] Nedeljković M., Damjanović A. (2019). Mechanisms of Chemotherapy Resistance in Triple-Negative Breast Cancer-How We Can Rise to the Challenge. Cells.

[5] O’Conor C.J., Chen T., González I., Cao D., Peng Y. (2018). Cancer Stem Cells in Triple-Negative Breast Cancer: A Potential Target and Prognostic Marker. Biomark. Med..

[6] Park C. (2019). Nam Targeting Cancer Stem Cells in Triple-Negative Breast Cancer. Cancers..

[7] Singleton C.S., Chan L.L., McCulley K.J., Kessel S.L., Del Valle L., Crabtree J.S. (2022). ER+ Breast Cancer Mammosphere Formation and Analysis. Methods Mol. Biol..

[8] Wei Y., Li Y., Chen Y., Liu P., Huang S., Zhang Y., Sun Y., Wu Z., Hu M., Wu Q. (2022). ALDH1: A Potential Therapeutic Target for Cancer Stem Cells in Solid Tumors. Front. Oncol..

[9] Pires B.R.B., DE Amorim Í.S.S., Souza L.D.E., Rodrigues J.A., Mencalha A.L. (2016). Targeting Cellular Signaling Pathways in Breast Cancer Stem Cells and Its Implication for Cancer Treatment. Anticancer Res..

[10] Zeng X., Liu C., Yao J., Wan H., Wan G., Li Y., Chen N. (2021). Breast Cancer Stem Cells, Heterogeneity, Targeting Therapies and Therapeutic Implications. Pharmacol. Res..

[11] Song K., Farzaneh M. (2021). Signaling Pathways Governing Breast Cancer Stem Cells Behavior. Stem Cell Res. Ther..

[12] Talib W.H., Alsalahat I., Daoud S., Abutayeh R.F., Mahmod A.I. (2020). Plant-Derived Natural Products in Cancer Research: Extraction, Mechanism of Action, and Drug Formulation. Molecules.

[13] Puig T., Turrado C., Benhamú B., Aguilar H., Relat J., Ortega-Gutiérrez S., Casals G., Marrero P.F., Urruticoechea A., Haro D. (2009). Novel Inhibitors of Fatty Acid Synthase with Anticancer Activity. Clin. Cancer Res..

[14] Bimonte S., Cascella M., Barbieri A., Arra C., Cuomo A. (2020). Current Shreds of Evidence on the Anticancer Role of EGCG in Triple Negative Breast Cancer: An Update of the Current State of Knowledge. Infect Agent. Cancer.

[15] Oliveras G., Blancafort A., Urruticoechea A., Campuzano O., Gómez-Cabello D., Brugada R., López-Rodríguez M.L., Colomer R., Puig T. (2010). Novel Anti-Fatty Acid Synthase Compounds with Anti-Cancer Activity in HER2+ Breast Cancer. Ann. N. Y. Acad. Sci..

[16] Turrado C., Puig T., García-Cárceles J., Artola M., Benhamú B., Ortega-Gutiérrez S., Relat J., Oliveras G., Blancafort A., Haro D. (2012). New Synthetic Inhibitors of Fatty Acid Synthase with Anticancer Activity. J. Med. Chem..

[17] Crous-Masó J., Palomeras S., Relat J., Camó C., Martínez-Garza Ú., Planas M., Feliu L., Puig T. (2018). (−)-Epigallocatechin 3-Gallate Synthetic Analogues Inhibit Fatty Acid Synthase and Show Anticancer Activity in Triple Negative Breast Cancer. Molecules.

[18] Giró-Perafita A., Rabionet M., Planas M., Feliu L., Ciurana J., Ruiz-Martínez S., Puig T. (2019). EGCG-Derivative G28 Shows High Efficacy Inhibiting the Mammosphere-Forming Capacity of Sensitive and Resistant TNBC Models. Molecules.

[19] Liu C., Rokavec M., Huang Z., Hermeking H. (2023). Curcumin Activates a ROS/KEAP1/NRF2/MiR-34a/b/c Cascade to Suppress Colorectal Cancer Metastasis. Cell Death Differ..

[20] Giordano A., Tommonaro G. (2019). Tommonaro Curcumin and Cancer. Nutrients.

[21] Liu H.T., Ho Y.S. (2018). Anticancer Effect of Curcumin on Breast Cancer and Stem Cells. Food Sci. Hum. Wellness.

[22] Cridge B.J., Larsen L., Rosengren R.J. (2013). Curcumin and Its Derivatives in Breast Cancer: Current Developments and Potential for the Treatment of Drug-Resistant Cancers. Oncol. Discov..

[23] Dandawate P.R., Subramaniam D., Jensen R.A., Anant S. (2016). Targeting Cancer Stem Cells and Signaling Pathways by Phytochemicals: Novel Approach for Breast Cancer Therapy. Semin. Cancer Biol..

[24] Pal K., Roy S., Parida P.K., Dutta A., Bardhan S., Das S., Jana K., Karmakar P. (2019). Folic Acid Conjugated Curcumin Loaded Biopolymeric Gum Acacia Microsphere for Triple Negative Breast Cancer Therapy in Invitro and Invivo Model. Mater. Sci. Eng. C.

[25] Mbese Z., Khwaza V., Aderibigbe B.A. (2019). Curcumin and Its Derivatives as Potential Therapeutic Agents in Prostate, Colon and Breast Cancers. Molecules.

[26] Ramasamy T.S., Ayob A.Z., Myint H.H.L., Thiagarajah S., Amini F. (2015). Targeting Colorectal Cancer Stem Cells Using Curcumin and Curcumin Analogues: Insights into the Mechanism of the Therapeutic Efficacy. Cancer Cell Int..

[27] Razak N.A., Akhtar M.N., Abu N., Ho W.Y., Tan S.W., Zareen S., Taj-Ud-Din S.N.B., Long K., Alitheen N.B., Yeap S.K. (2017). The: In Vivo Anti-Tumor Effect of Curcumin Derivative (2 E,6 E)-2,6-Bis(4-Hydroxy-3-Methoxybenzylidene)Cyclohexanone (BHMC) on 4T1 Breast Cancer Cells. RSC Adv..

[28] Lin L., Hutzen B., Ball S., Foust E., Sobo M., Deangelis S., Pandit B., Friedman L., Li C., Li P.K. (2009). New Curcumin Analogues Exhibit Enhanced Growth-Suppressive Activity and Inhibit AKT and Signal Transducer and Activator of Transcription 3 Phosphorylation in Breast and Prostate Cancer Cells. Cancer Sci..

[29] Sardjiman S.S., Reksohadiprodjo M.S., Hakim L., van der Goot H., Timmerman H. (1997). 1,5-Diphenyl-1,4-Pentadiene-3-Ones and Cyclic Analogues as Antioxidative Agents. Synthesis and Structure-Activity Relationship. Eur. J. Med. Chem..

[30] Quincoces Suarez J.A., Rando D.G., Santos R.P., Gonalves C.P., Ferreira E., De Carvalho J.E., Kohn L., Maria D.A., Faião-Flores F., Michalik D. (2010). New Antitumoral Agents I: In Vitro Anticancer Activity and in Vivo Acute Toxicity of Synthetic 1,5-Bis(4-Hydroxy-3-Methoxyphenyl)-1,4-Pentadien-3-One and Derivatives. Bioorganic Med. Chem..

[31] Yuan J.-D., ZhuGe D.-L., Tong M.-Q., Lin M.-T., Xu X.-F., Tang X., Zhao Y.-Z., Xu H.-L. (2018). PH-Sensitive Polymeric Nanoparticles of MPEG-PLGA-PGlu with Hybrid Core for Simultaneous Encapsulation of Curcumin and Doxorubicin to Kill the Heterogeneous Tumour Cells in Breast Cancer. Artif. Cells Nanomed. Biotechnol..

[32] Hu C., Li M., Guo T., Wang S., Huang W., Yang K., Liao Z., Wang J., Zhang F., Wang H. (2019). Anti-Metastasis Activity of Curcumin against Breast Cancer via the Inhibition of Stem Cell-like Properties and EMT. Phytomedicine.

[33] Abadi A.J., Mirzaei S., Mahabady M.K., Hashemi F., Zabolian A., Hashemi F., Raee P., Aghamiri S., Ashrafizadeh M., Aref A.R. (2022). Curcumin and Its Derivatives in Cancer Therapy: Potentiating Antitumor Activity of Cisplatin and Reducing Side Effects. Phyther. Res..

[34] Wang R., Lv Q., Meng W., Tan Q., Zhang S., Mo X., Yang X. (2014). Comparison of Mammosphere Formation from Breast Cancer Cell Lines and Primary Breast Tumors. J. Thorac. Dis..

[35] Fultang N., Chakraborty M., Peethambaran B. (2021). Regulation of Cancer Stem Cells in Triple Negative Breast Cancer. Cancer Drug Resist..

[36] Li X., Wang X., Xie C., Zhu J., Meng Y., Chen Y., Li Y., Jiang Y., Yang X., Wang S. (2018). Sonic Hedgehog and Wnt/β-Catenin Pathways Mediate Curcumin Inhibition of Breast Cancer Stem Cells. Anticancer Drugs.

[37] Suman S., Das T.P., Damodaran C. (2013). Silencing NOTCH Signaling Causes Growth Arrest in Both Breast Cancer Stem Cells and Breast Cancer Cells. Br. J. Cancer.

[38] Calaf G.M., Ponce-Cusi R., Abarca-Quinones J. (2018). Effect of Curcumin on the Cell Surface Markers CD44 and CD24 in Breast Cancer. Oncol. Rep..

[39] He L., Wick N., Germans S.K., Peng Y. (2021). The Role of Breast Cancer Stem Cells in Chemoresistance and Metastasis in Triple-Negative Breast Cancer. Cancers.

[40] Landeros N., Castillo I., Pérez-Castro R. (2023). Preclinical and Clinical Trials of New Treatment Strategies Targeting Cancer Stem Cells in Subtypes of Breast Cancer. Cells.

[41] Wang T., Fahrmann J.F., Lee H., Li Y.-J., Tripathi S.C., Yue C., Zhang C., Lifshitz V., Song J., Yuan Y. (2018). JAK/STAT3-Regulated Fatty Acid β-Oxidation Is Critical for Breast Cancer Stem Cell Self-Renewal and Chemoresistance. Cell Metab..

[42] Zhou Q., Ye M., Lu Y., Zhang H., Chen Q., Huang S., Su S. (2015). Curcumin Improves the Tumoricidal Effect of Mitomycin C by Suppressing ABCG2 Expression in Stem Cell-like Breast Cancer Cells. PLoS ONE.

[43] Wen C., Fu L., Huang J., Dai Y., Wang B., Xu G., Wu L., Zhou H. (2019). Curcumin Reverses Doxorubicin Resistance via Inhibition the Efflux Function of ABCB4 in Doxorubicin-resistant Breast Cancer Cells. Mol. Med. Rep..

[44] Calaf G.M., Ponce-Cusi R., Carrión F. (2018). Curcumin and Paclitaxel Induce Cell Death in Breast Cancer Cell Lines. Oncol. Rep..

[45] Bahramsoltani R., Rahimi R., Farzaei M.H. (2017). Pharmacokinetic Interactions of Curcuminoids with Conventional Drugs: A Review. J. Ethnopharmacol..

[46] Polonio-Alcalá E., Palomeras S., Torres-Oteros D., Relat J., Planas M., Feliu L., Ciurana J., Ruiz-Martínez S., Puig T. (2020). Fatty Acid Synthase Inhibitor G28 Shows Anticancer Activity in EGFR Tyrosine Kinase Inhibitor Resistant Lung Adenocarcinoma Models. Cancers.

[47] Chou T.-C. (2006). Theoretical Basis, Experimental Design, and Computerized Simulation of Synergism and Antagonism in Drug Combination Studies. Pharmacol. Rev..

