# Supplementary material for: Inhibition of Cancer Stem-like Cells by Curcumin and Other Polyphenol Derivatives in MDA-MB-231 TNBC Cells
Source: Int J Mol Sci. 2024 Jul 6;25(13):7446. doi: 10.3390/ijms25137446 (PMC11242520; doi:10.3390/ijms25137446)
Supplement: Supplementary file 1 [file ijms-25-07446-s001.zip › ijms-3029241-supplementary.pdf]

Supporting Information

Inhibition of cancer stem-like cells by curcumin and other polyphenol derivatives in TNBC cells

Maria Ros [1,†], Gerard Riesco-Llach [2,†], Emma Polonio-Alcalá [1], Pere Miquel Morla-Barcelo [3], Santiago Ruiz-Martínez [4], Lidia Feliu [2,*], Marta Planas [2,*], Teresa Puig [1,*]

[1] New Therapeutic Targets Laboratory (TargetsLab)-Oncology Unit, Department of Medical Sciences, Faculty of Medicine, University of Girona, Spain.

[2] Laboratori d'Innovació en Processos i Productes de Síntesi Orgànica (LIPPSO), Department of Chemistry, University of Girona, Spain.

[3] Grupo Multidisciplinar de Oncología Traslacional, Institut Universitari d'Investigació en Ciències de la Salut (IUNICS), Universitat de les Illes Balears, Spain.

[4] Department of Laboratory Medicine, Institute of Biomedicine, Sahlgrenska Academy, Sahlgrenska Center for Cancer Research, University of Gothenburg, Sweden.

[†] These authors contributed equally to this work

Table of contents

1. Supplementary materials of the biological assays S3

1.1. Western blot antibodies S3

1.2. Cytotoxicity of DMSO, TL3 and TL4 .... S4

1.3. Cell viability of MDA-MB-231 treated cells S5

1.4. Western blot results S6

2. Spectroscopic data (NMR, IR, UV-VIS, HPLC, ESI-MS and HRMS) of the compounds synthesized S7

2.1. 3,4,5-Tris(*tert*-butyldimethylsilyloxy)benzoic acid S7

2.2. 2,6-Bis((*E*)-4-hydroxy-3-methoxybenzylidene)cyclohexan-1-one (**1**) S9

2.3. (1*E*,4*E*)-1,5-Bis(4-hydroxy-3-methoxyphenyl)penta-1,4-dien-3-one (**2**) S14

2.4. ((1*E*,1'*E*)-(2-Oxocyclohexane-1,3-diylidene)bis(methanylylidene))bis(2-methoxy-4,1-phenylene) bis[3,4,5-tris((*tert*-butyldimethylsilyl)oxy)benzoate] S19

2.5. ((1*E*,4*E*)-3-Oxopenta-1,4-diene-1,5-diyl)bis(2-methoxy-4,1-phenylene) bis[3,4,5-tris((*tert*-butyldimethylsilyl)oxy)benzoate] S24

2.6. 1,4-Phenylenebis(methylene) bis[3,4,5-tris((*tert*-butyldimethylsilyl)oxy)benzoate] S30

2.7. Benzene-1,3,5-triyl tris[3,4,5-tris((*tert*-butyldimethylsilyl)oxy)benzoate] S33

2.8. ((1*E*,3*Z*,6*E*)-3-Hydroxy-5-oxohepta-1,3,6-triene-1,7-diyliden)bis(2-methoxy-4,1-phenylene) bis[3,4,5-tris((*tert*-butyldimethylsilyl)oxy)benzoate] S36

2.9. 1,4-Phenylenebis(methylene) bis(3,4,5-trihydroxybenzoate) (TL1) S39

2.10.Benzene-1,3,5-triyl tris(3,4,5-trihydroxybenzoate) (TL2) S46

2.11.1,4-Phenylenebis(methylene) bis(3,4,5-trihydroxybenzoate) (TL3) S52

2.12.. ((1*E*,1'*E*)-(2-Oxocyclohexane-1,3-diylidene)bis(methanylylidene))bis(2-methoxy-4,1-phenylene) bis[3,4,5-tris((*tert*-butyldimethylsilyl)oxy)benzoate] (TL4) S60

2.13.. ((1*E*,1'*Z*)-(2-Oxocyclohexane-1,3-diylidene)bis(methanylylidene))bis(2-methoxy-4,1-phenylene) bis[3,4,5-tris((*tert*-butyldimethylsilyl)oxy)benzoate] (TL5) S68

2.14.((1*E*,4*E*)-3-Oxopenta-1,4-diene-1,5-diyl)bis(2-methoxy-4,1-phenylene) bis[3,4,5-tris((*tert*-butyldimethylsilyl)oxy)benzoate] (TL6) S77

1. Supplementary materials of the biological assays

1.1. Western blot antibodies

Table S1. Western Blot Antibodies

Antibody	*Molecular Weight (kDa)*	*Blocking solution*	*Dilution*	*Source*	*Commercial*
CD24 (ab179821) (EPR3006(N)	35-50		1:1000		
CD44 (ab243894)(BLR038F)	81	5% of Milk in TBS-T	1:1000		Abcam ©
LC3B (ab48394)	13-15		1:15000		
ALDH1A1 (D9Q8E) XP®	55		1:1000		
Notch (Val11744) (D3B8)	110	5% of BSA in TBS-T	1:1000		
Shh (C9C5)	19		1:1000	Rabbit	
β-Catenin (6B3)	92		1:1000		Cell Signaling Technology®
GAPDH (D16H11)	37		1:2500		
PARP	89-116	% of Milk in TBS-T	1:1000		
Phospho-β-Catenin (Ser552) (D8E11)	92		1:1000		

1.2. Cytotoxicity of DMSO, TL3 and TL4

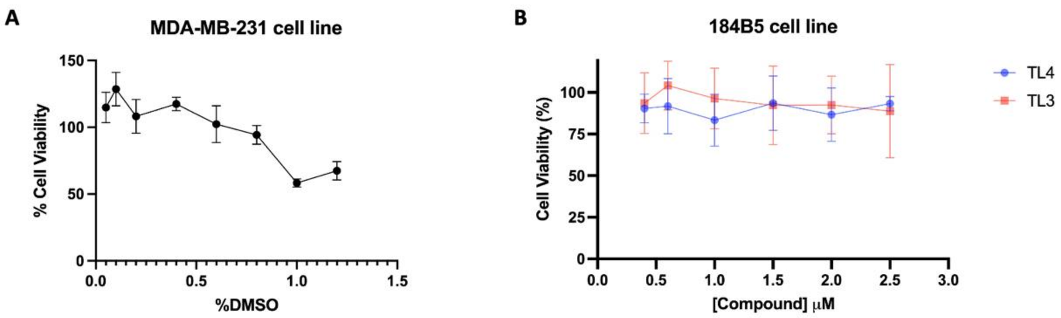

Figure S1. **(A)** Cell viability of MDA-MB-231 cells treated with 0.05 to 1.20% DMSO for 72 hours. **(B)** Cell viability of non-tumoral 184B5 breast epithelial cells treated with TL3 (from 0.4 to 2.5 μM) and TL4 (from 0.4 to 2.5 μM) for 72 hours.

1.3. Cell viability of MDA-MB-231 treated cells

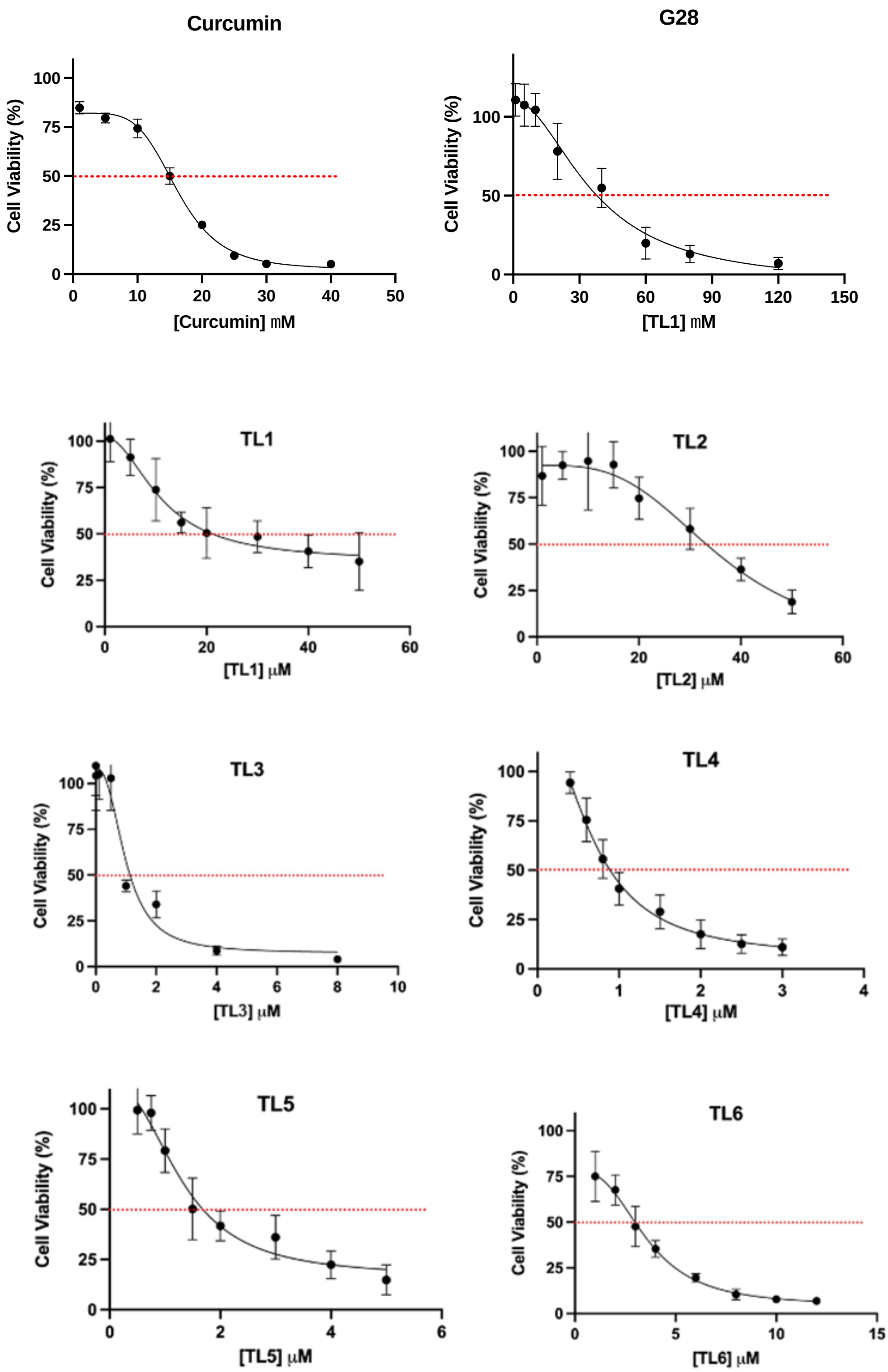

Figure S2. Cell viability of MDA-MB-231 cells treated with curcumin (from 1 to 40 µM), G28 (from 1 to 120 µM), TL1 (from 1 to 50 µM), TL2 (from 1 to 50 µM), TL3 (from 0.001 to 8 µM), TL4 (from 0.4 to 3 µM), TL5 (from 0.5 to 5 µM), and TL6 (from 1 to 12 µM) for 72 hours. Results are expressed as the percentage of surviving cells (mean ± SE) compared to control (untreated cells) from at least three independent experiments.

1.4. Western blot results

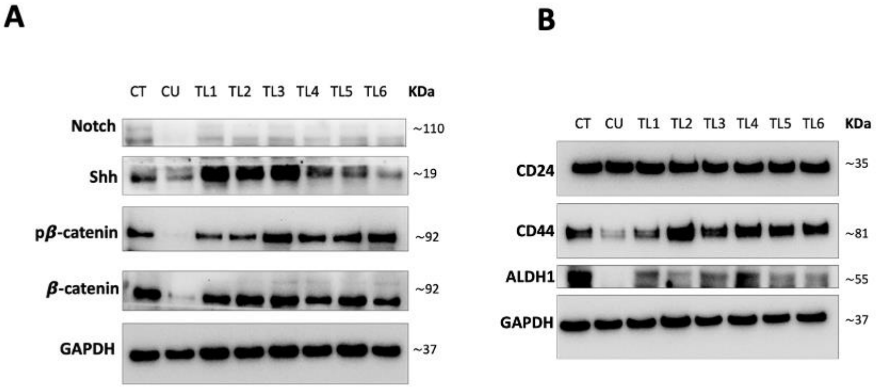

Figure S3. **(A)** Representative western blot images of proteins from embryonic signaling pathways are shown from a total of four replicates. (**B)** Representative western blot images of proteins from stemness markers are shown from a total of four replicates.

2. Spectroscopic data (NMR, IR, UV-VIS, HPLC, ESI-MS and HRMS) of the compounds synthesized

2.1. 3,4,5-Tris(*tert*-butyldimethylsilyloxy)benzoic acid

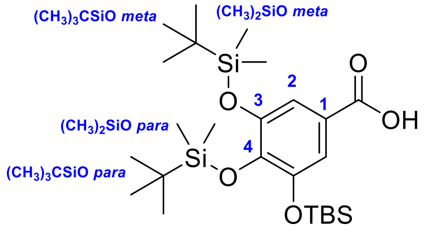

^{1}H-NMR

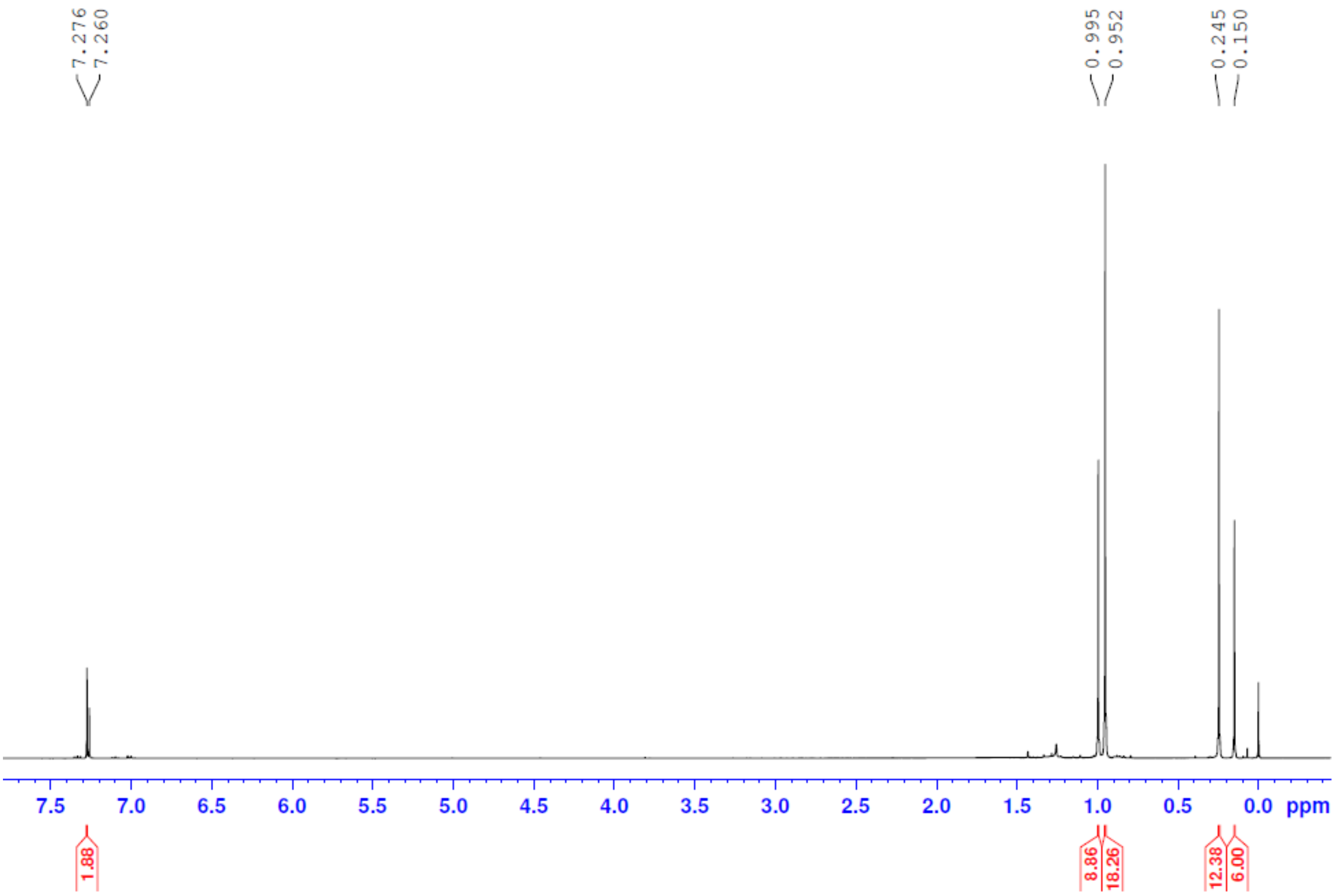

(–)-ESI-MS (*m/z*)

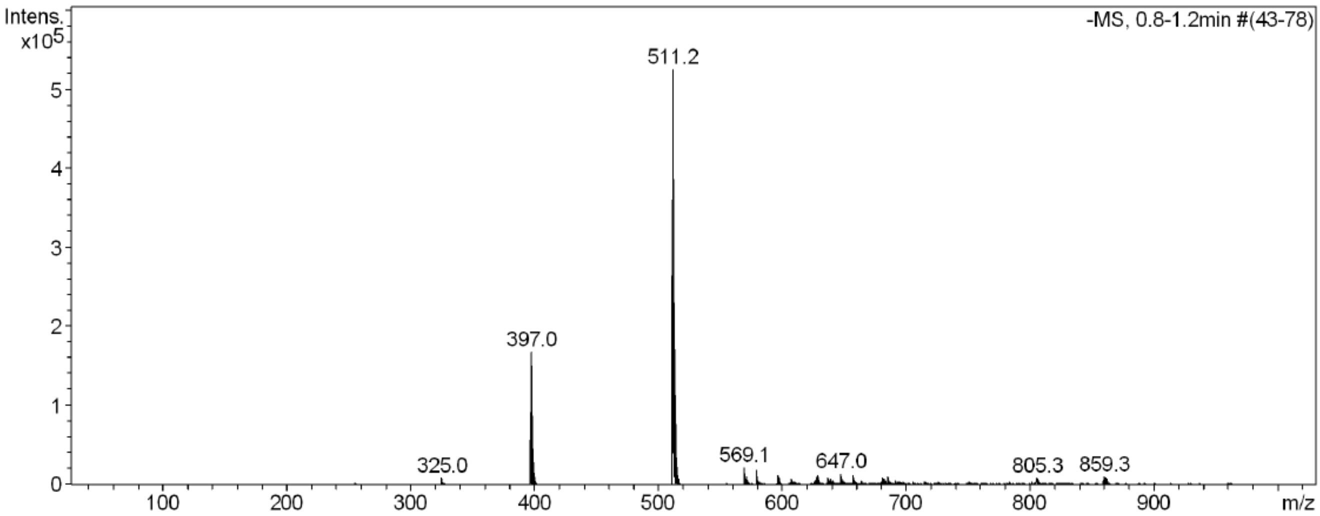
Intens.
x10^5
5
4
3
2
1
0
-MS, 0.8-1.2min #(43-78)
511.2
397.0
325.0
569.1
647.0
805.3
859.3
100
200
300
400
500
600
700
800
900
m/z

2.2. 2,6-Bis((*E*)-4-hydroxy-3-methoxybenzylidene)cyclohexan-1-one (1)

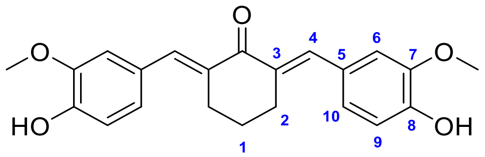

^{1}H-NMR (400 MHz, $(CD_3)_2SO$), δ (ppm)

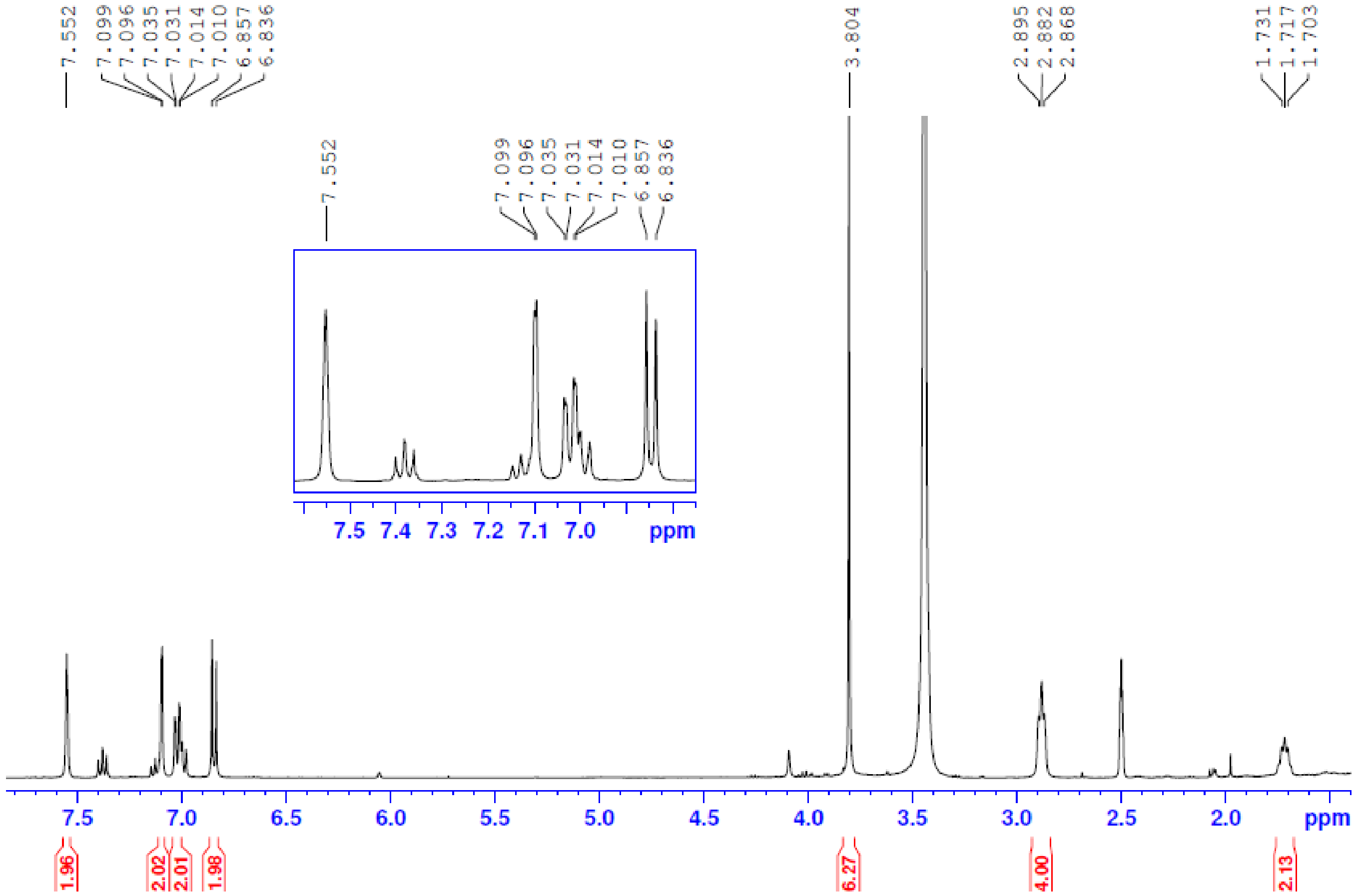

^{13}C-NMR (100 MHz, $(CD_3)_2SO$), δ (ppm)

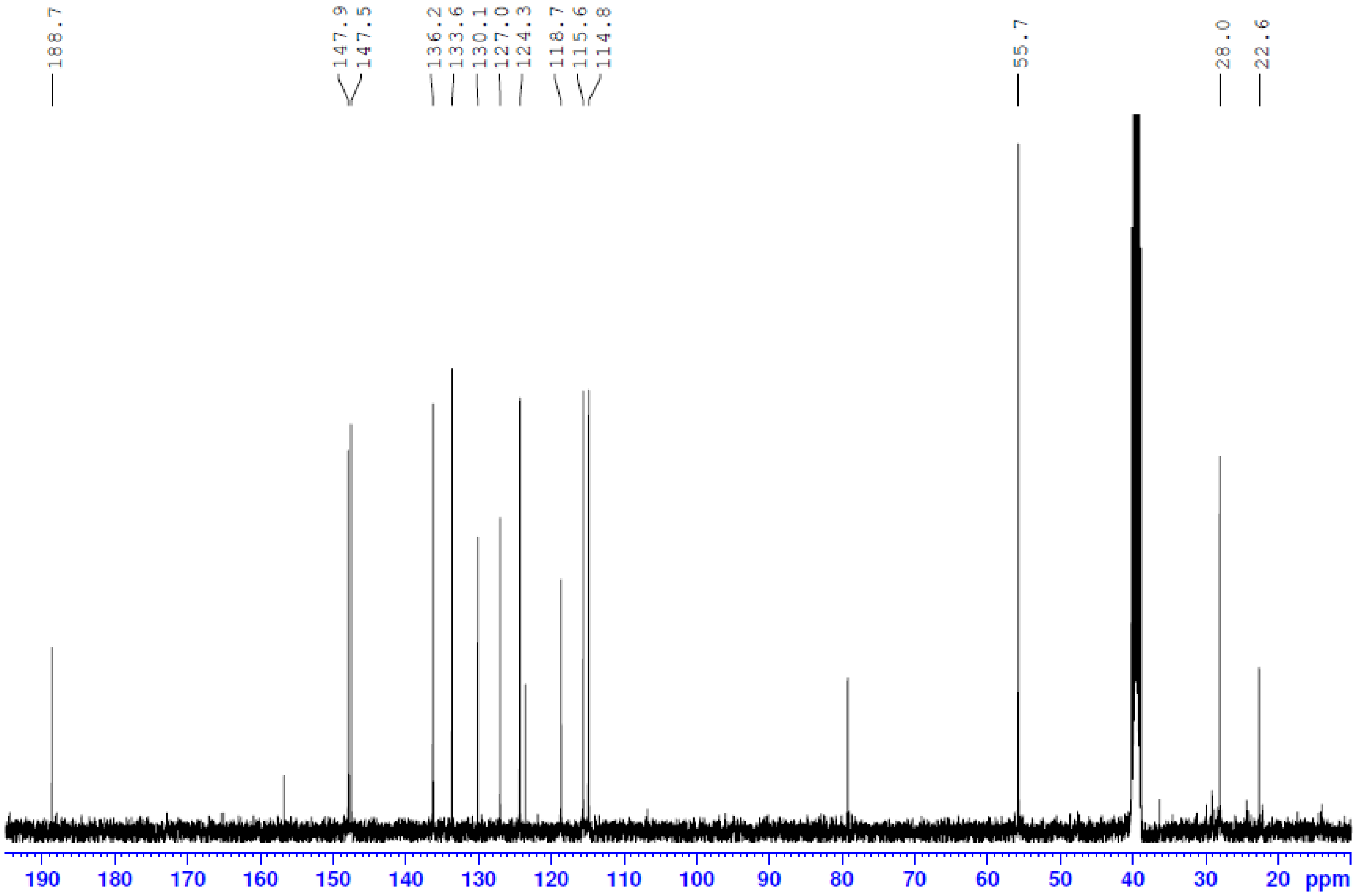

COSY ^{1}H-^{1}H ($(CD_3)_2SO$), δ (ppm)

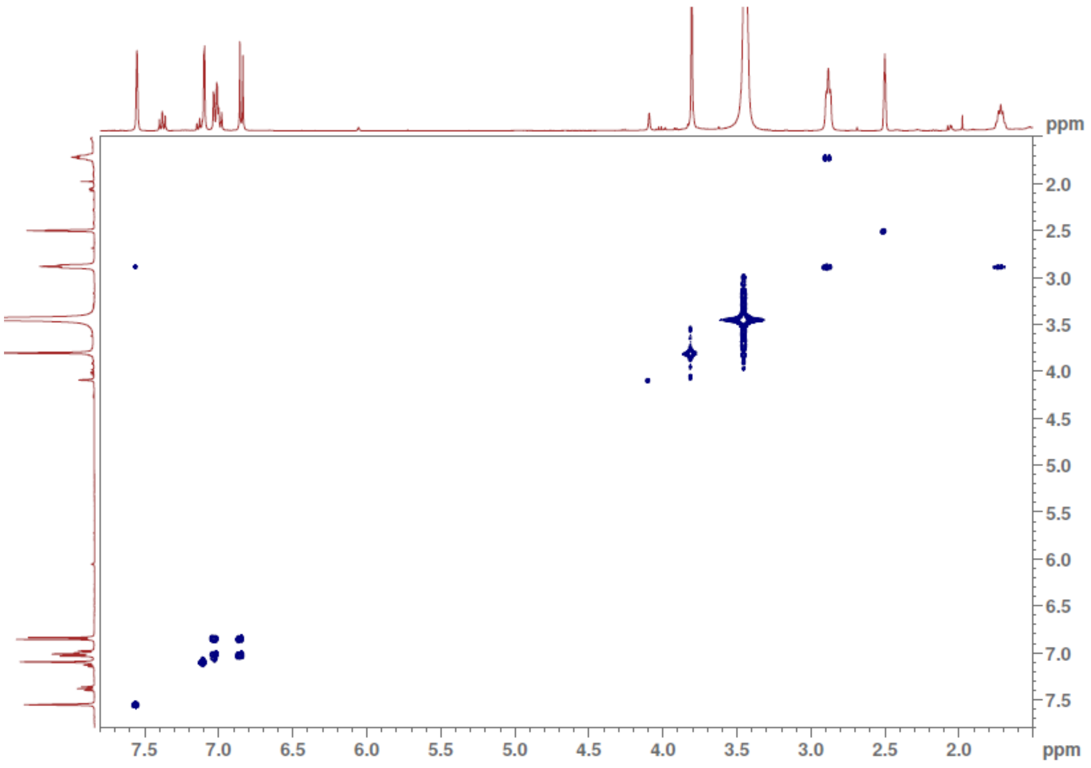

HSQC ^1H-^{13}C ($(CD_3)_2SO$), δ (ppm)

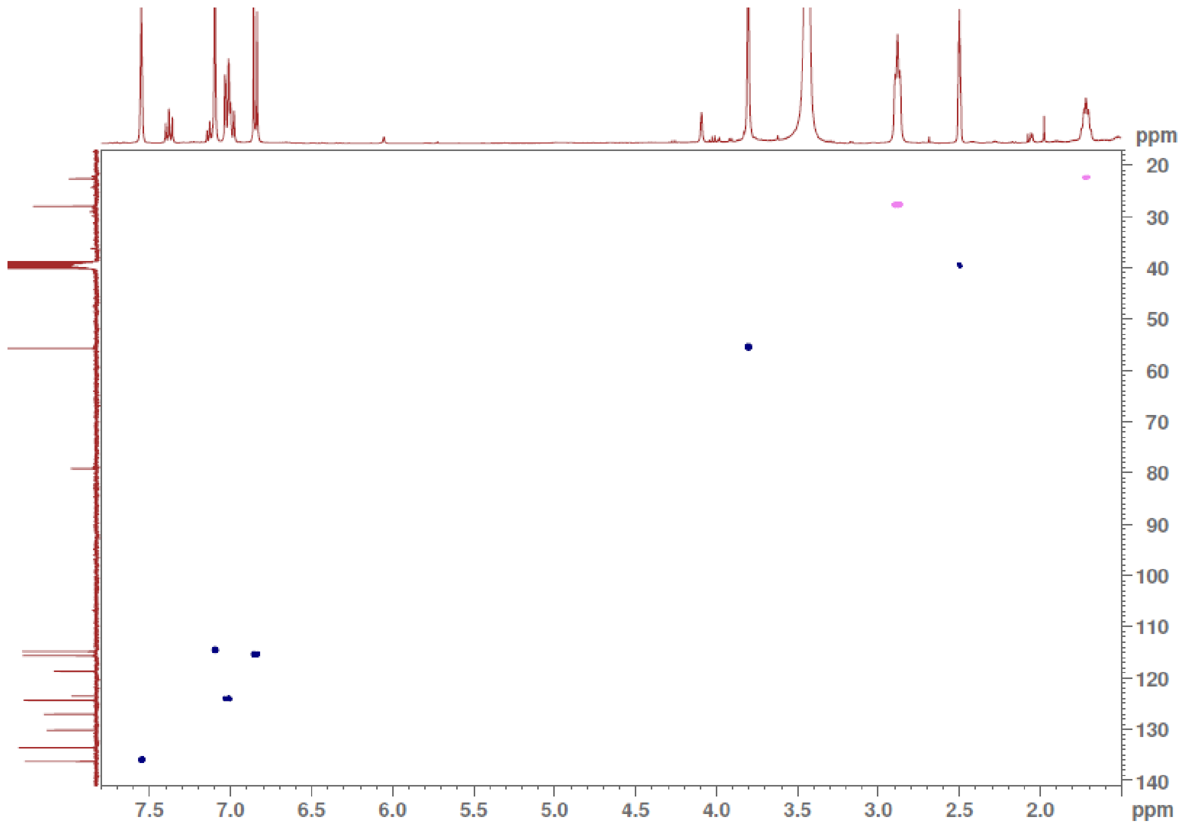

HMBC ^1H-^{13}C ($(CD_3)_2SO$), δ (ppm)

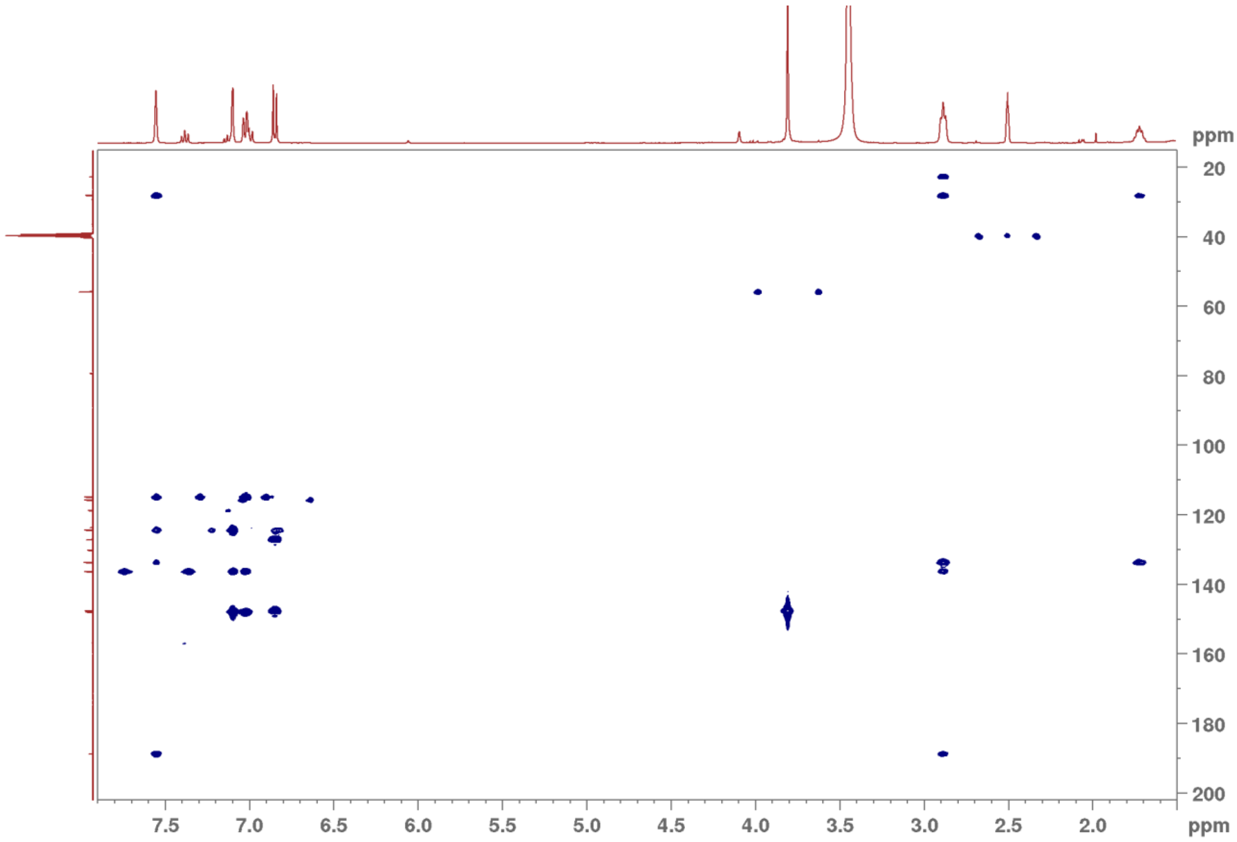

FT-IR (neat), ν (cm^{-1})

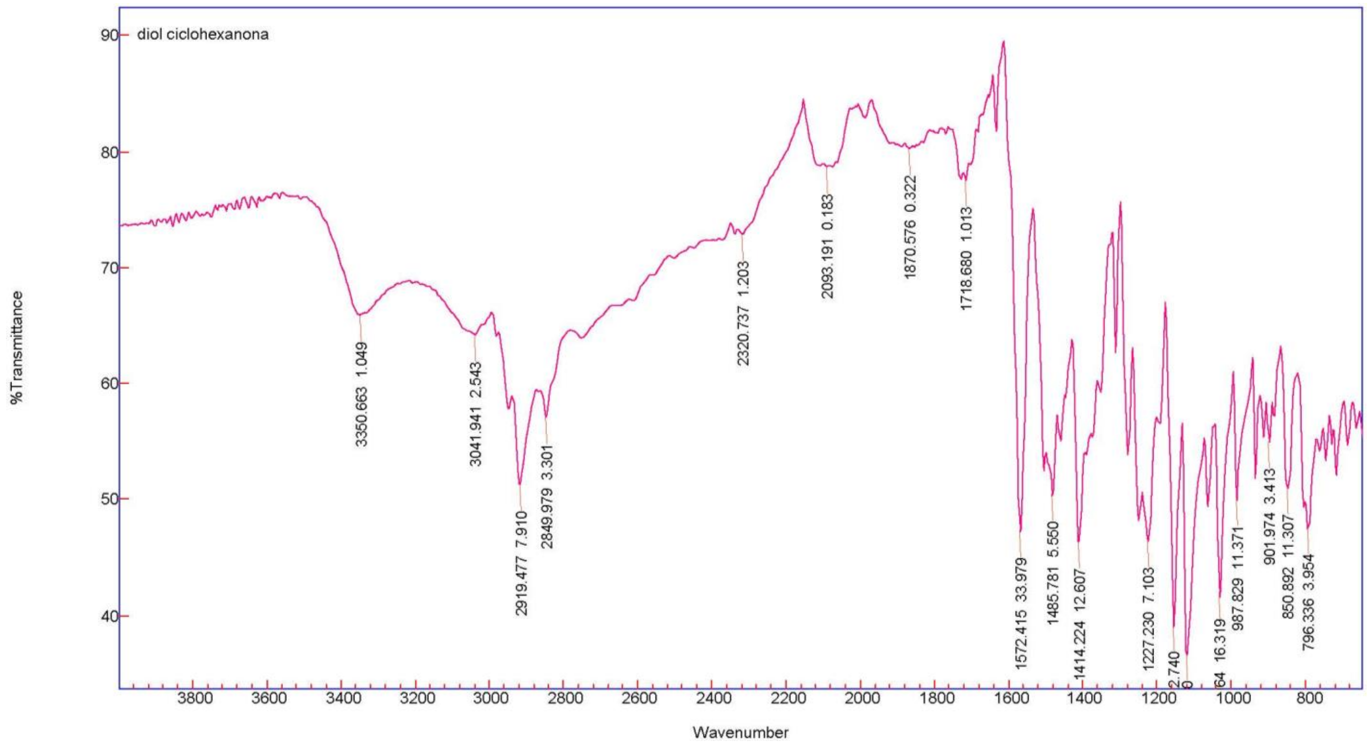

HPLC (λ = 360 nm)

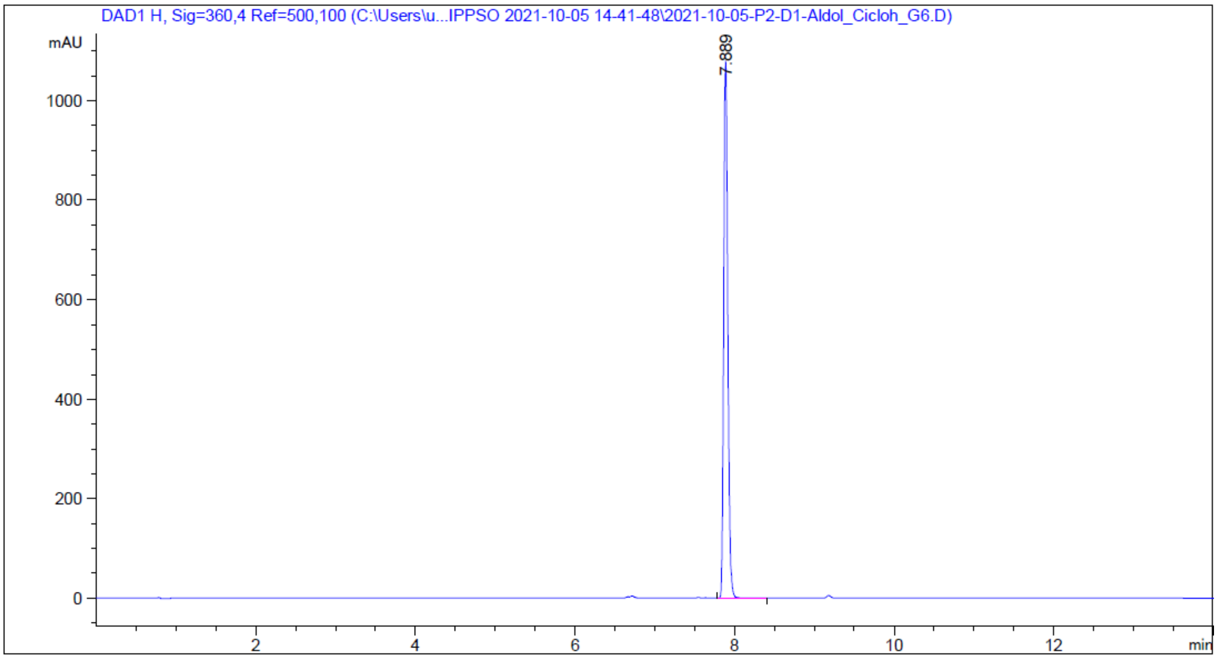

Peak #	RetTime [min]	Type	Width [min]	Area [mAU*s]	Height [mAU]	Area %
1	7.889	BB	0.0533	3834.10083	1082.30933	100.0000
Totals :				3834.10083	1082.30933	

(–)-ESI-MS (*m/z*)

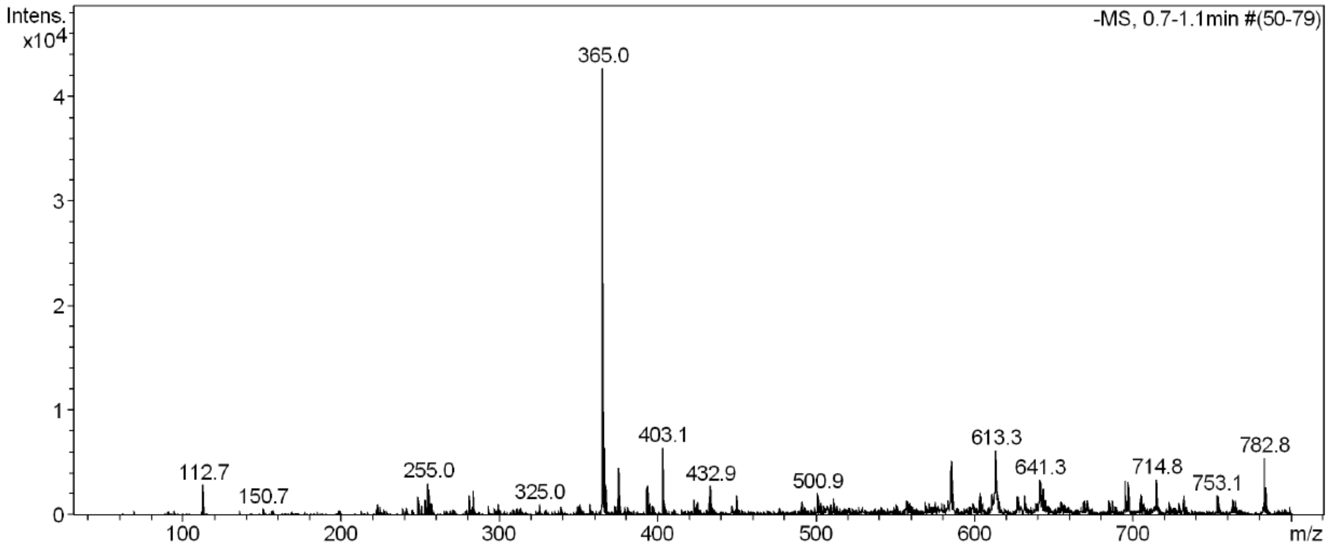
Intens.
x10^4
-MS, 0.7-1.1min #(50-79)
365.0
403.1
432.9
112.7
150.7
255.0
325.0
500.9
613.3
641.3
714.8
753.1
782.8
m/z

2.3. (1*E*,4*E*)-1,5-Bis(4-hydroxy-3-methoxyphenyl)penta-1,4-dien-3-one (2)

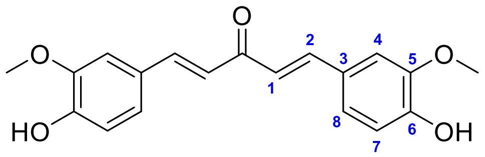

^{1}H-NMR (400 MHz, $(CD_3)_2CO$), δ (ppm)

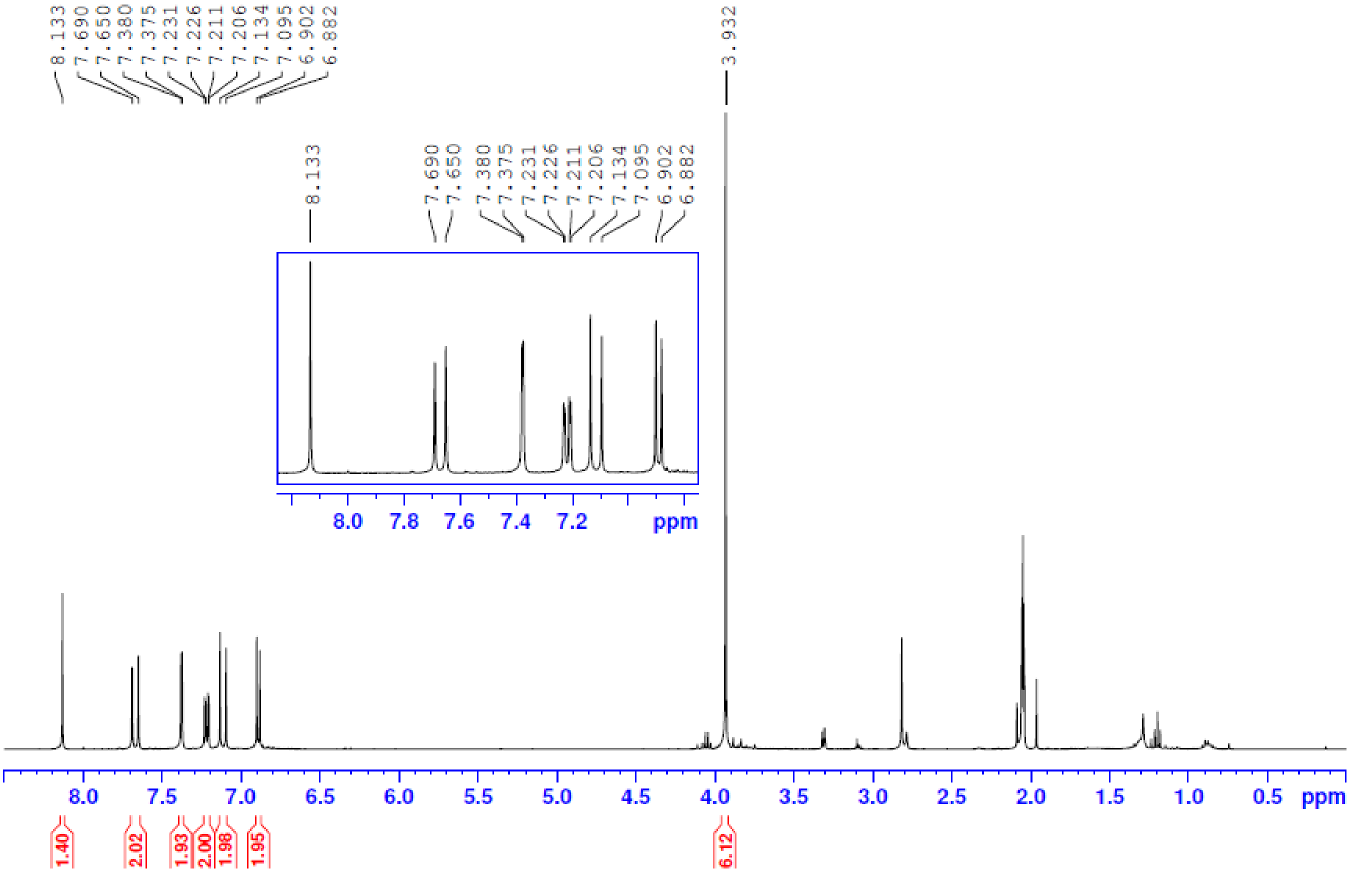

^{13}C-NMR (100 MHz, $(CD_3)_2SO$), δ (ppm)

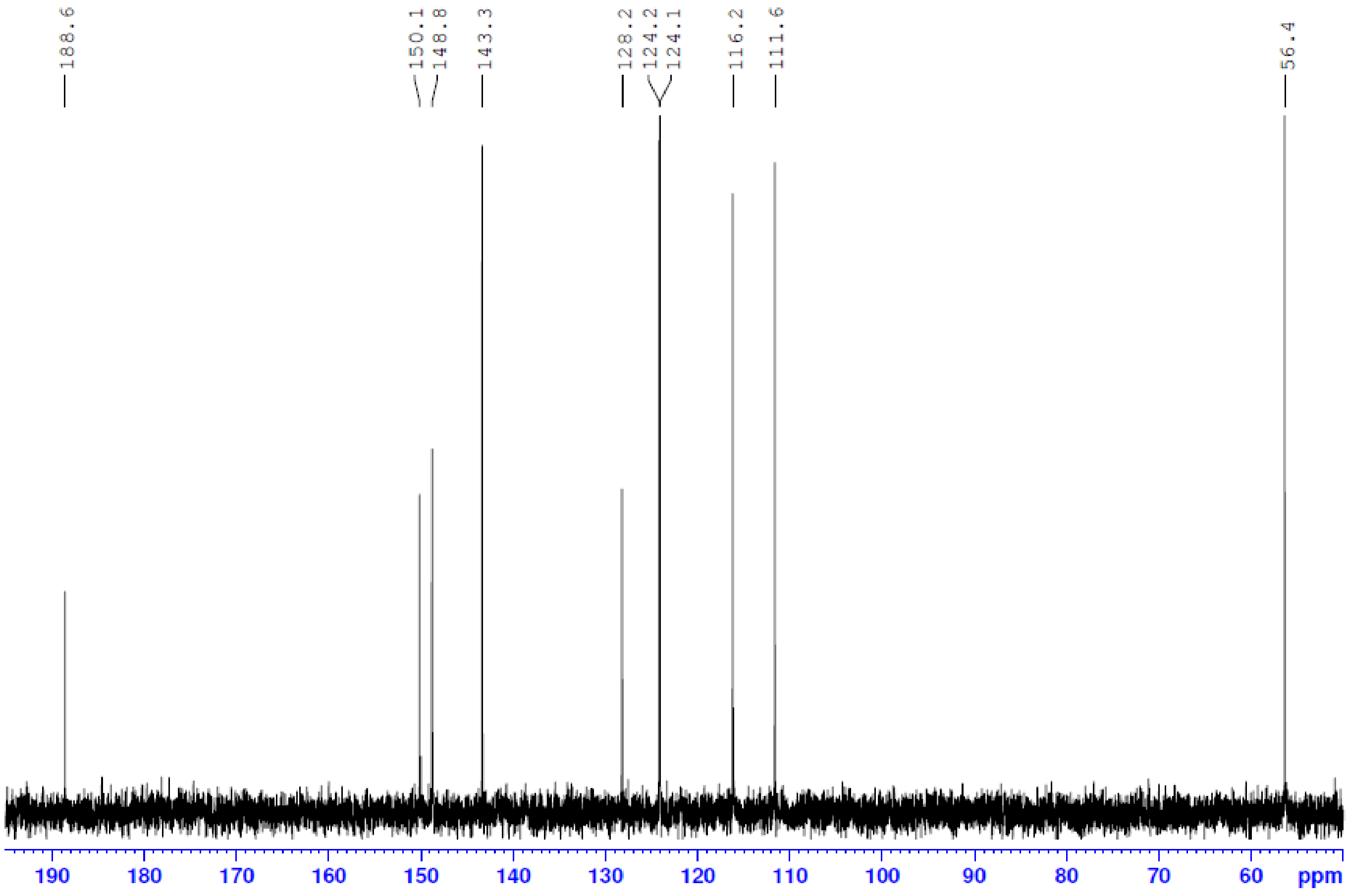

COSY ^{1}H-^{1}H ($(CD_3)_2SO$), δ (ppm)

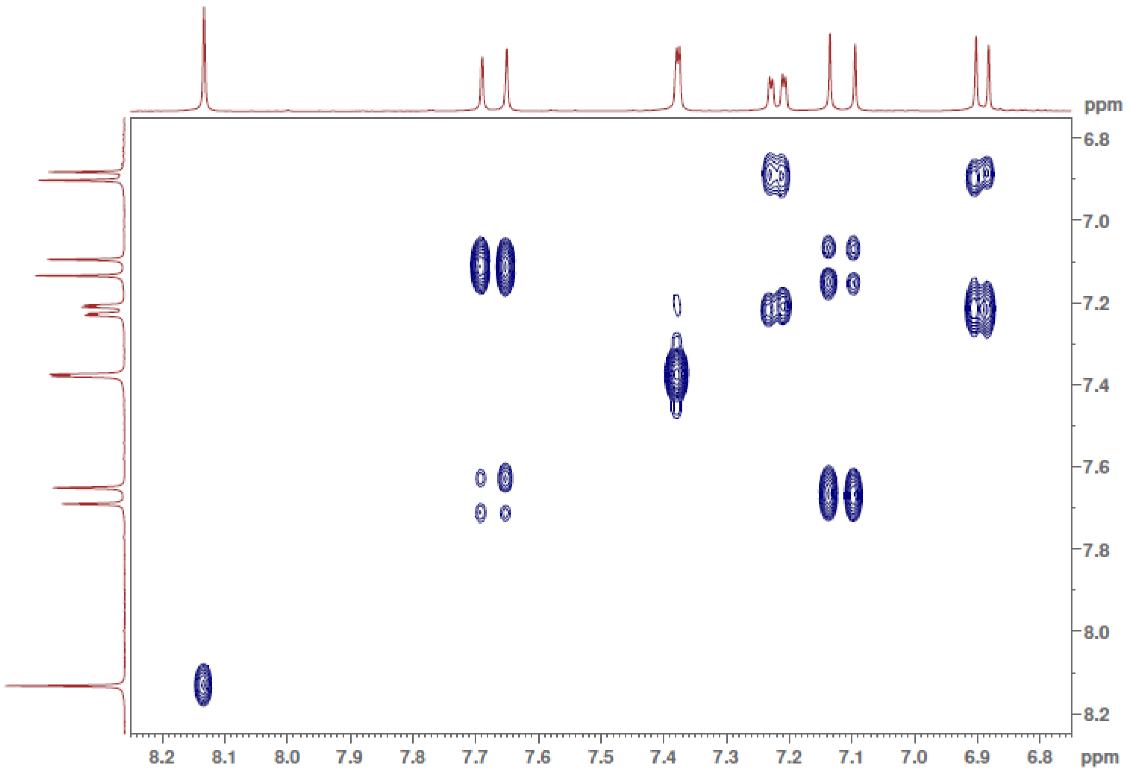

HSQC ^1H-^{13}C ($(CD_3)_2SO$), δ (ppm)

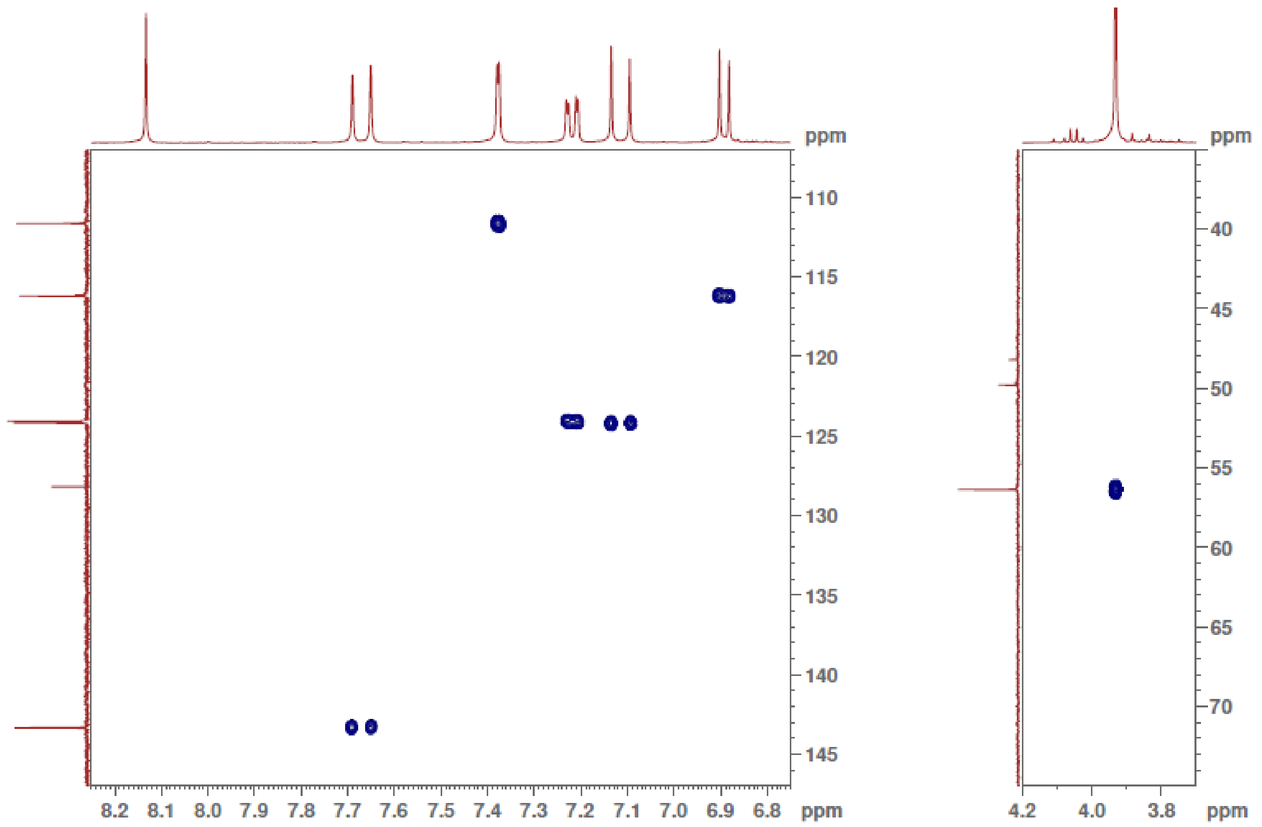

HMBC ^1H-^{13}C ($(CD_3)_2SO$), δ (ppm)

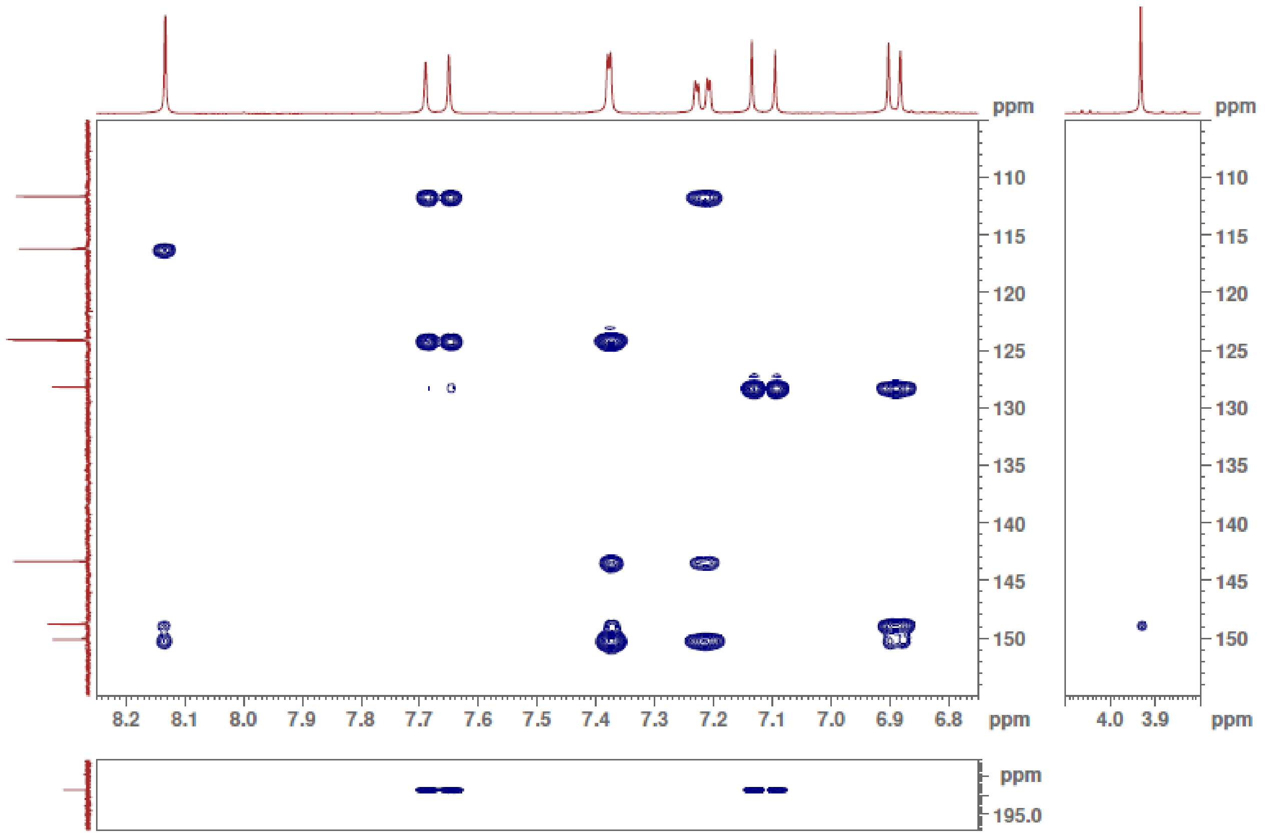

FT-IR (neat), ν (cm^{-1})

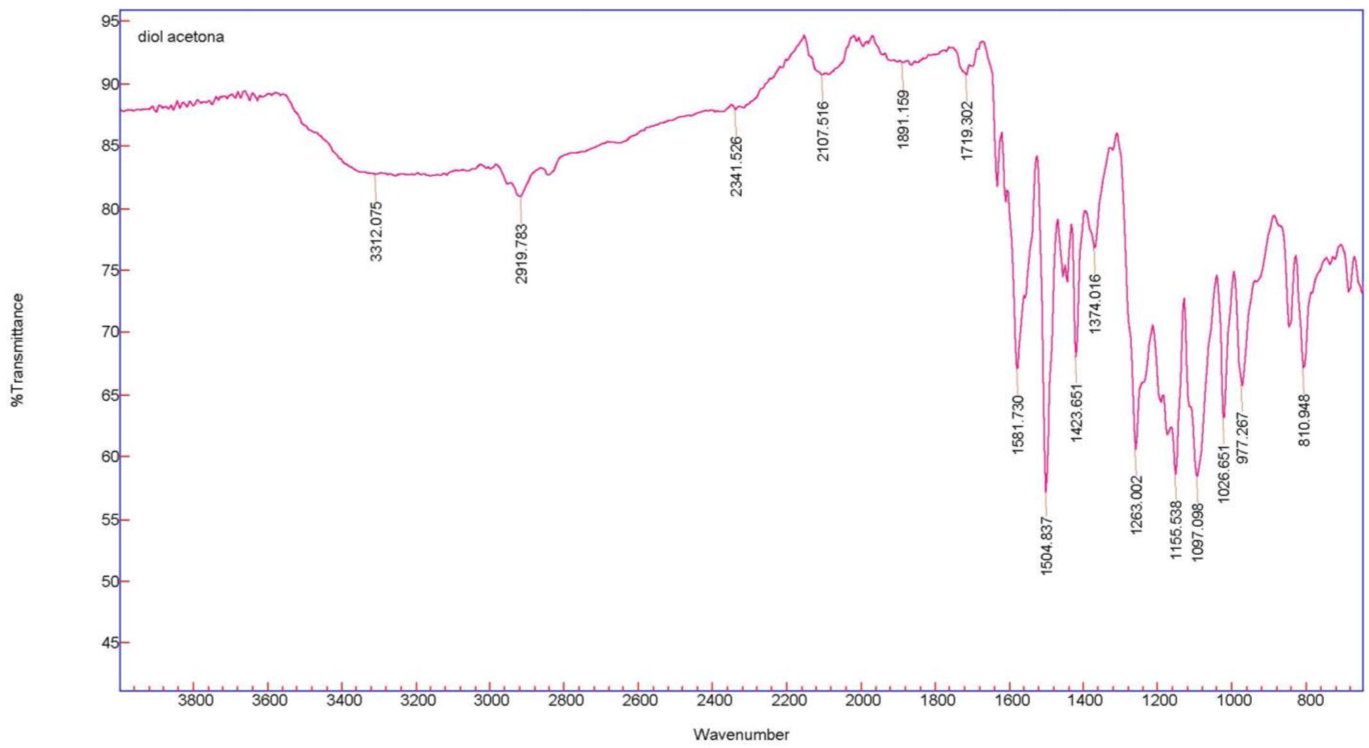

HPLC (λ = 360 nm)

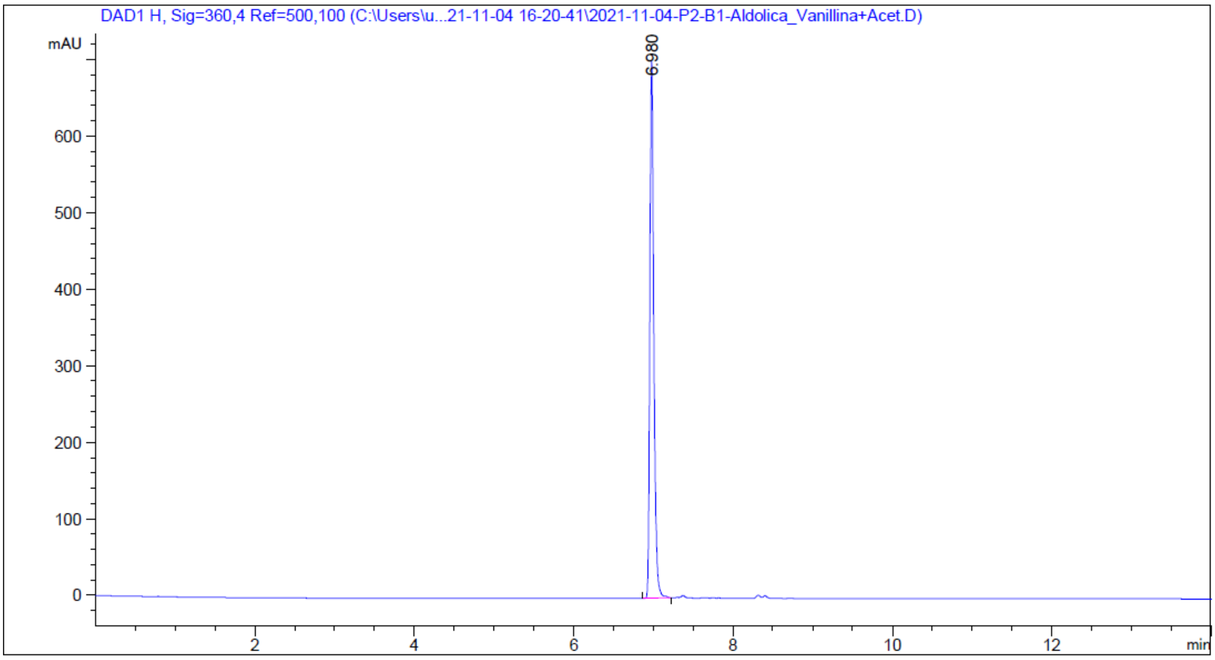

Peak #	RetTime [min]	Type	Width [min]	Area [mAU*s]	Height [mAU]	Area %
1	6.980	BB	0.0511	2411.89429	702.30542	100.0000

Totals : 2411.89429 702.30542

(–)-ESI-MS (*m/z*)

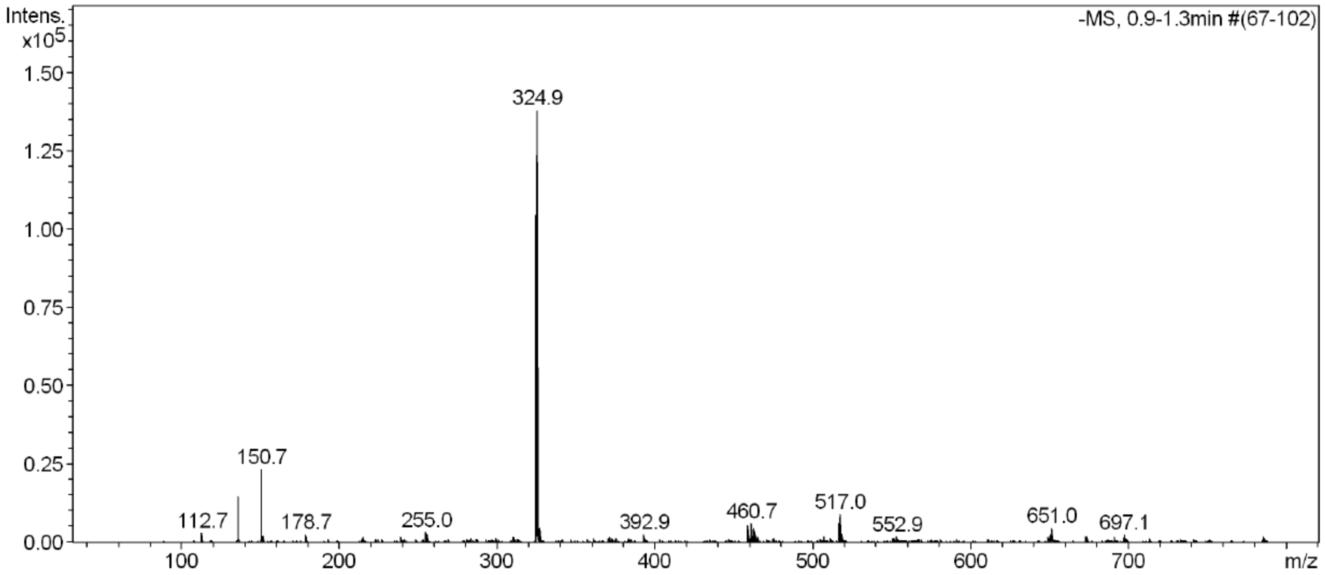

Intens.
x10^5
-MS, 0.9-1.3min #(67-102)
1.50
1.25
1.00
0.75
0.50
0.25
0.00
324.9
150.7
112.7
178.7
255.0
392.9
460.7
517.0
552.9
651.0
697.1
100
200
300
400
500
600
700
m/z

2.4. ((1*E*,1'*E*)-(2-Oxocyclohexane-1,3-diylidene)bis(methanylylidene))bis(2-methoxy-4,1-phenylene) bis[3,4,5-tris((*tert*-butyldimethylsilyl)oxy)benzoate]

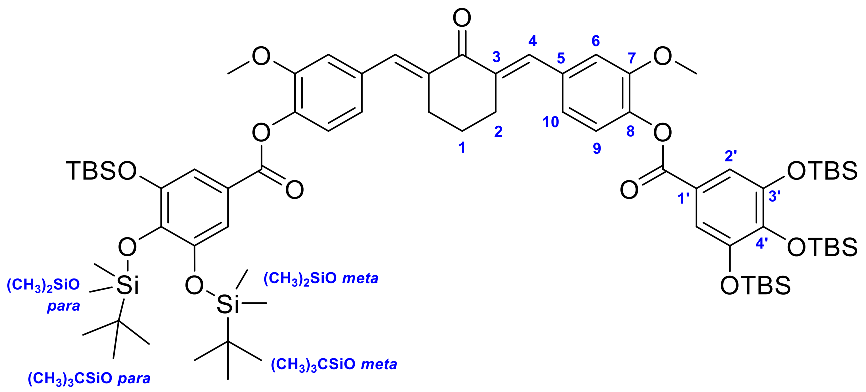

^{1}H-NMR (400 MHz, $(CD_3)_2CO$), δ (ppm)

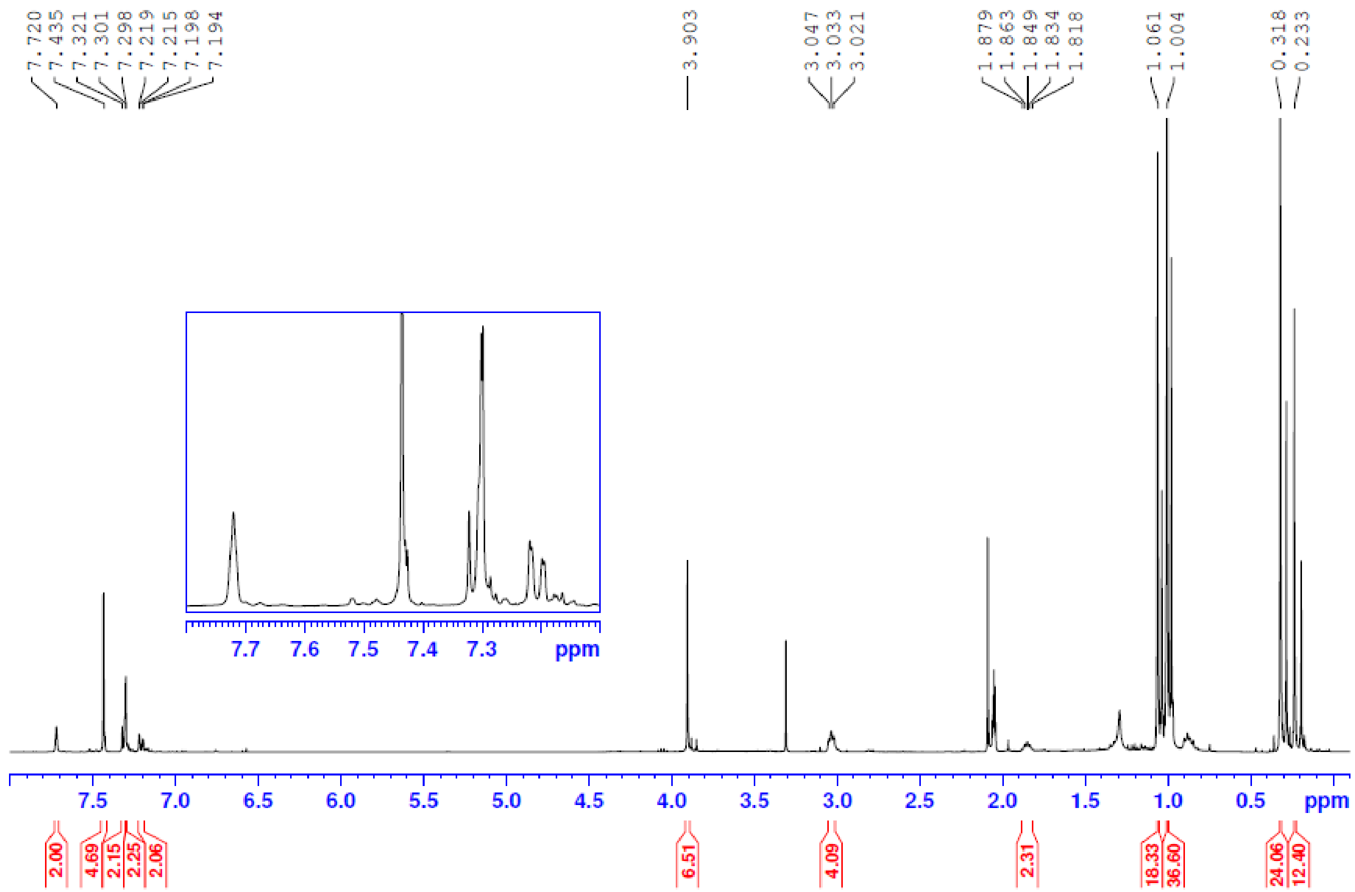

^{13}C-NMR (100 MHz, $(CD_3)_2CO$), δ (ppm)

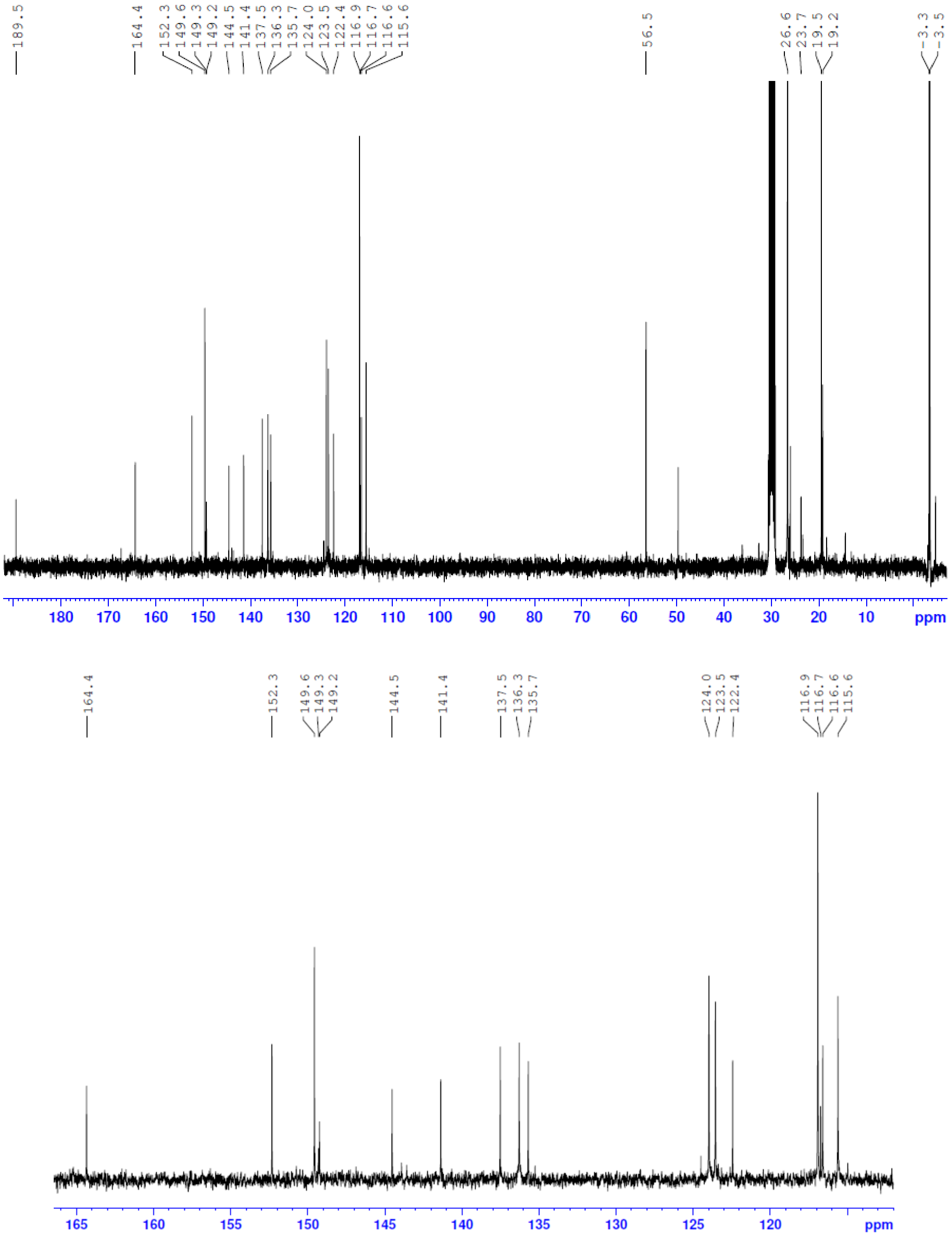

HSQC ^{1}H-^{13}C ($(CD_3)_2CO$), δ (ppm)

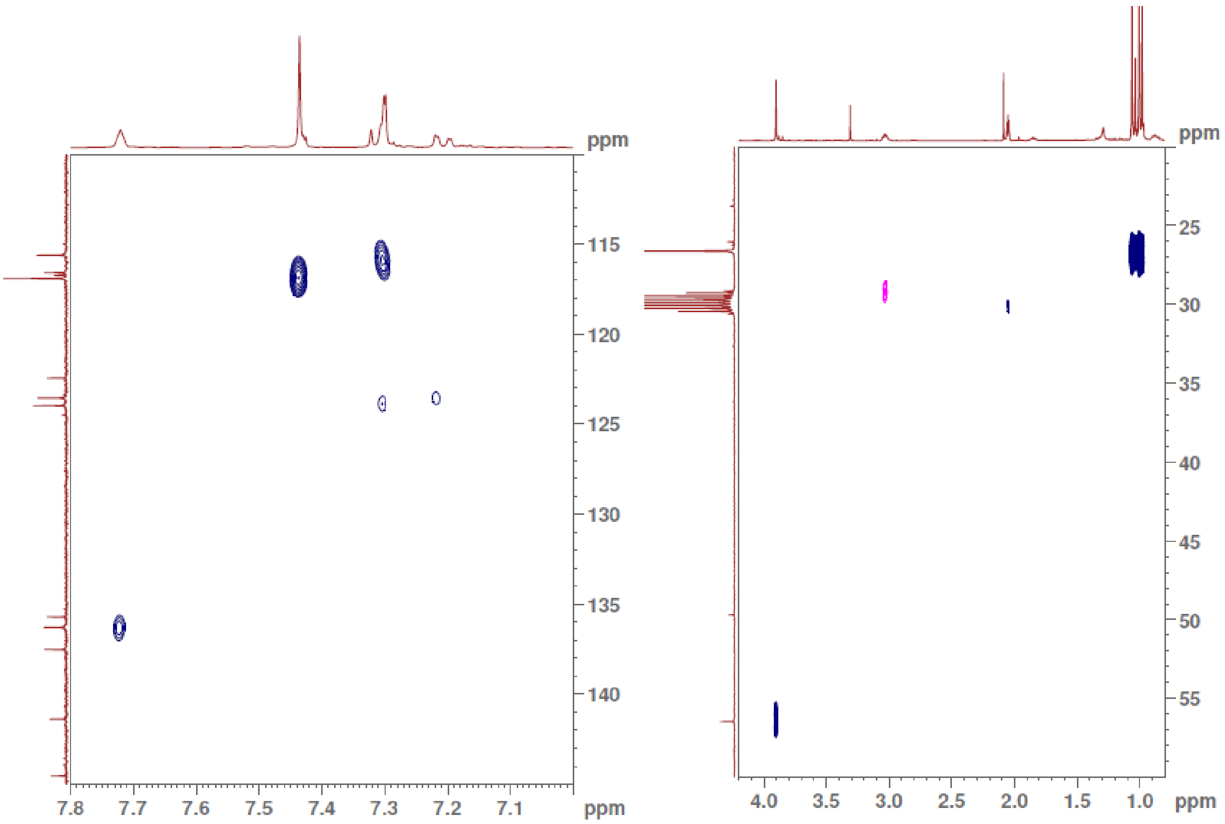

HMBC ^{1}H-^{13}C ($(CD_3)_2CO$), δ (ppm)

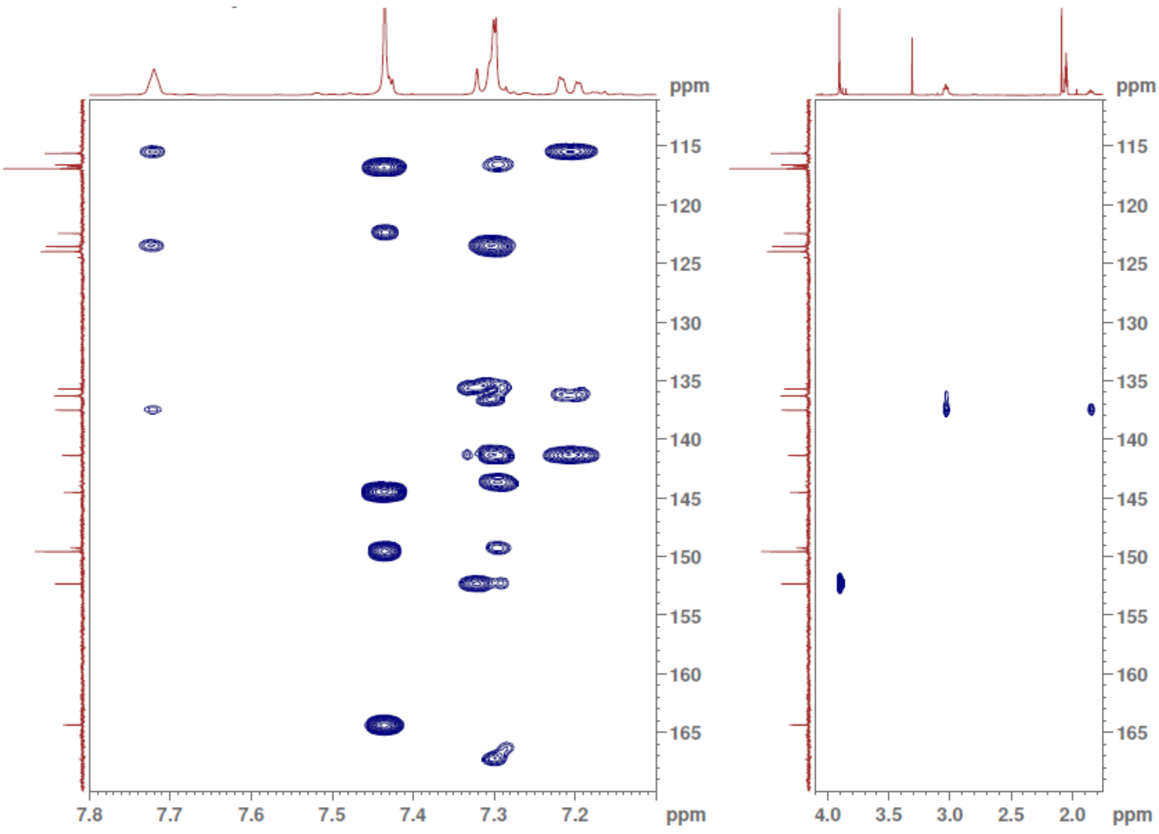

FT-IR (neat), ν (cm^{-1})

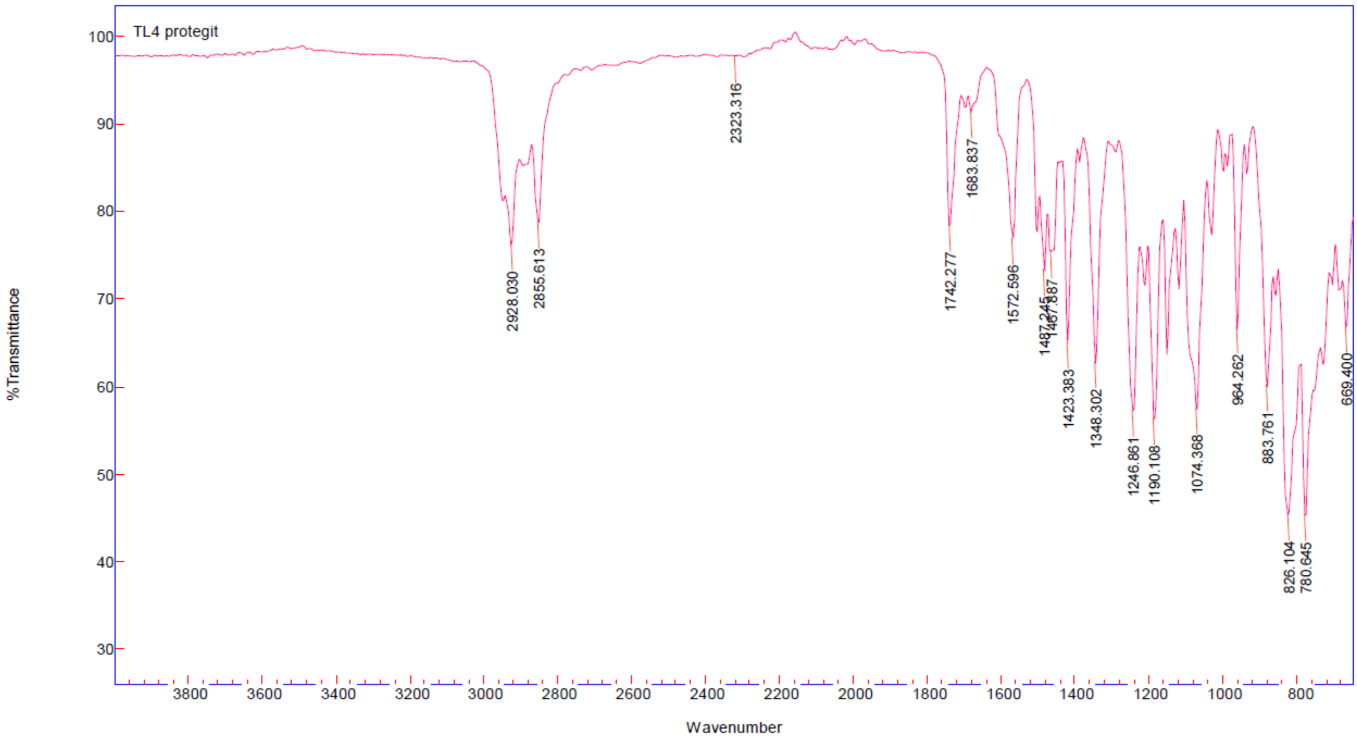

HRMS (m/z)

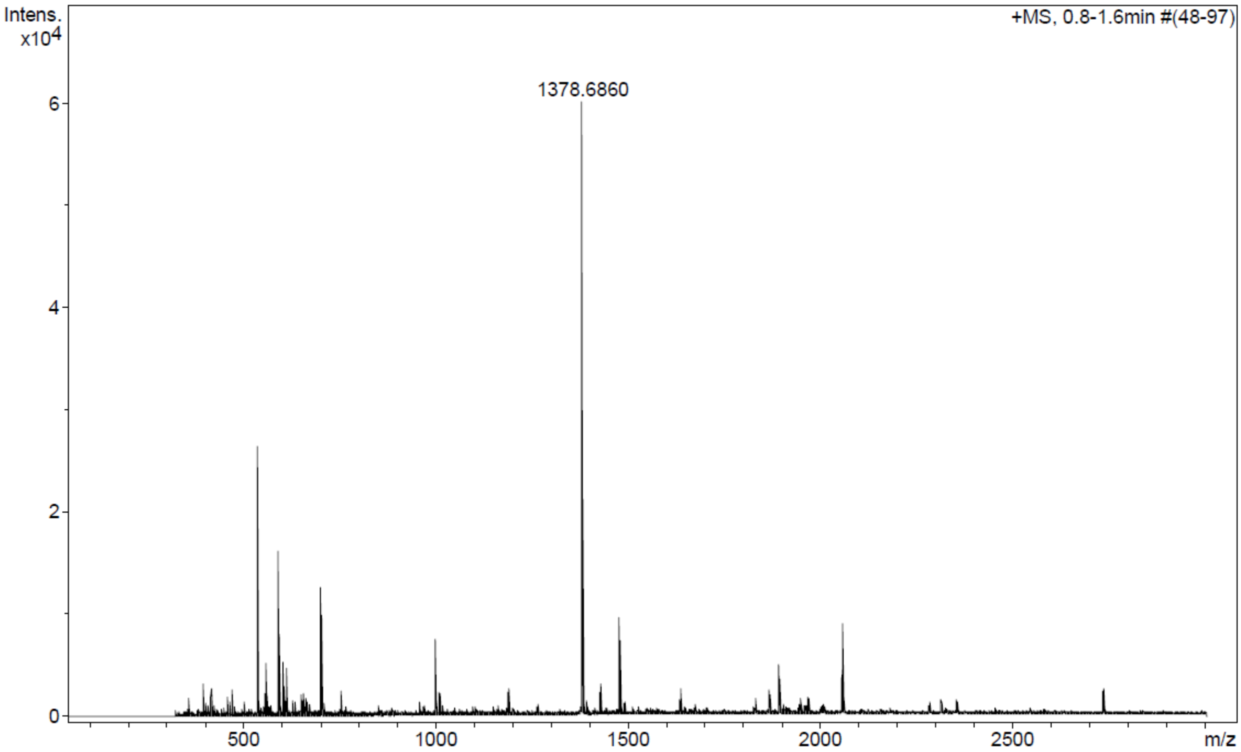

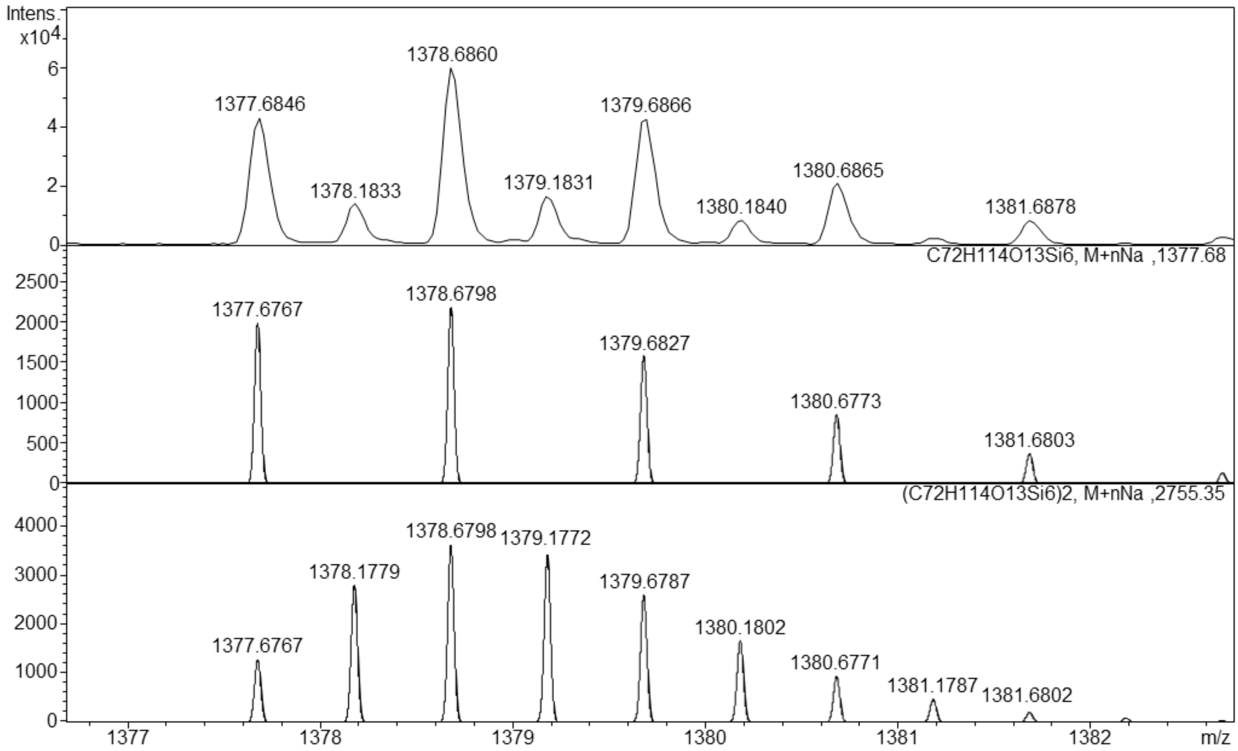

	Molecular formula	Calculated	Found
**$[M+Na]^+$**	$C_{72}H_{114}O_{13}Si_6Na$	1377.6767	1377.6846
**$[2M+2Na]^{2+}$**	$(C_{72}H_{114}O_{13}Si_6)_2Na_2$	1377.6767	1377.6846

2.5. ((1*E*,4*E*)-3-Oxopenta-1,4-diene-1,5-diyl)bis(2-methoxy-4,1-phenylene) bis[3,4,5-tris((*tert*-butyldimethylsilyl)oxy)benzoate]

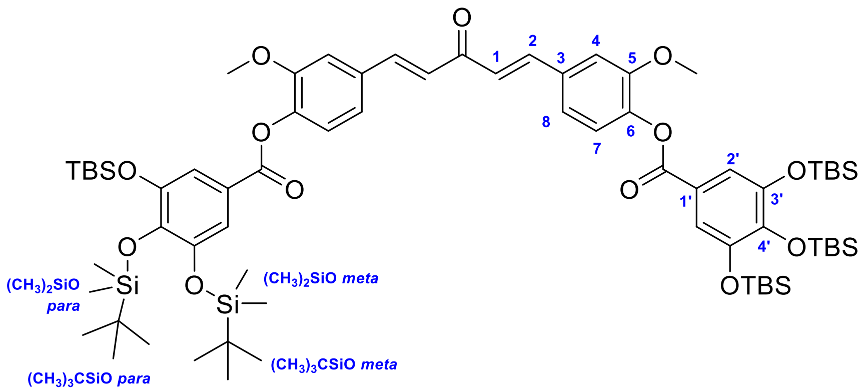

^{1}H-NMR (400 MHz, CDCl$_3$), δ (ppm)

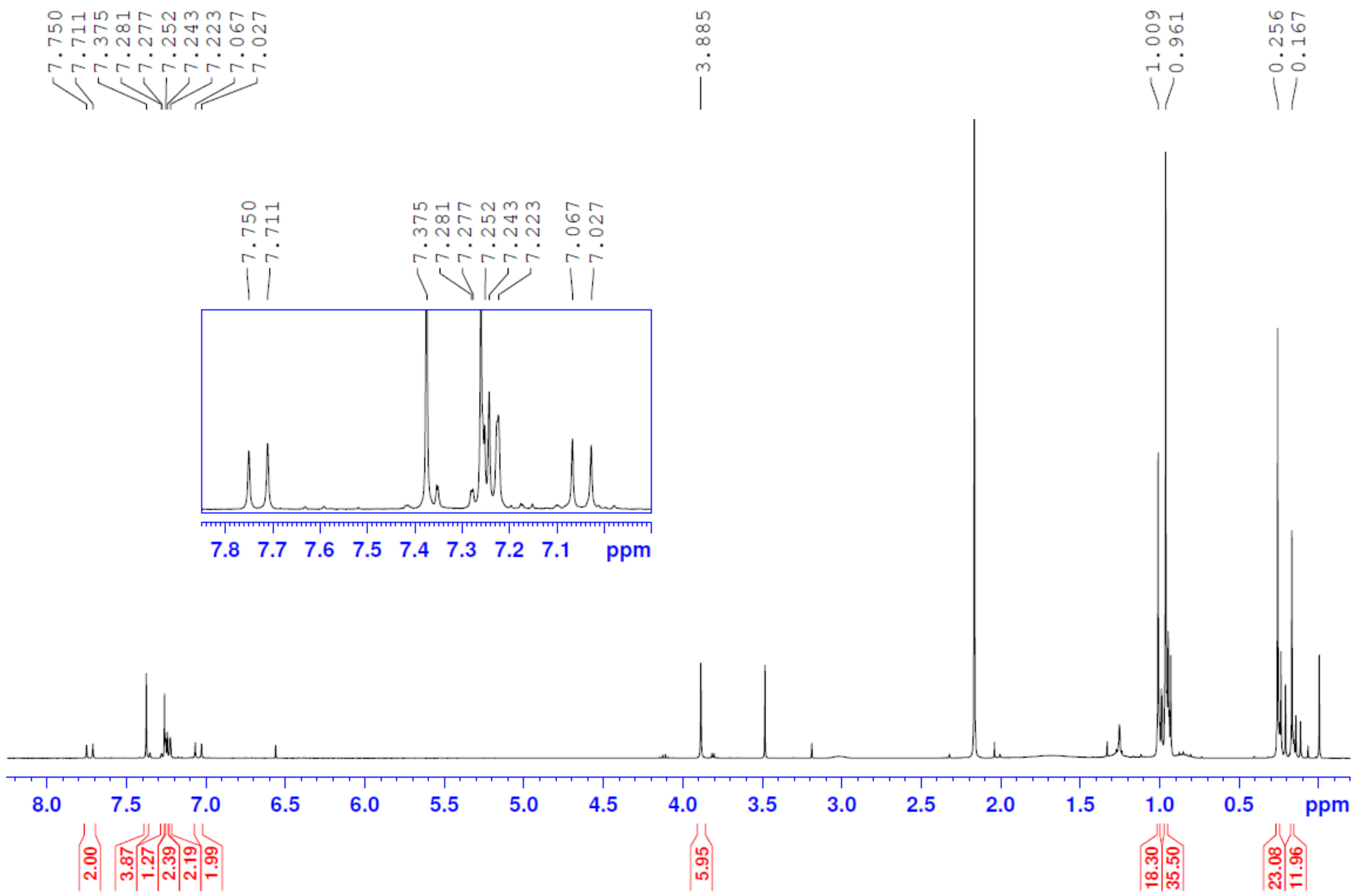

^{13}C-NMR (100 MHz, $CDCl_3$), δ (ppm)

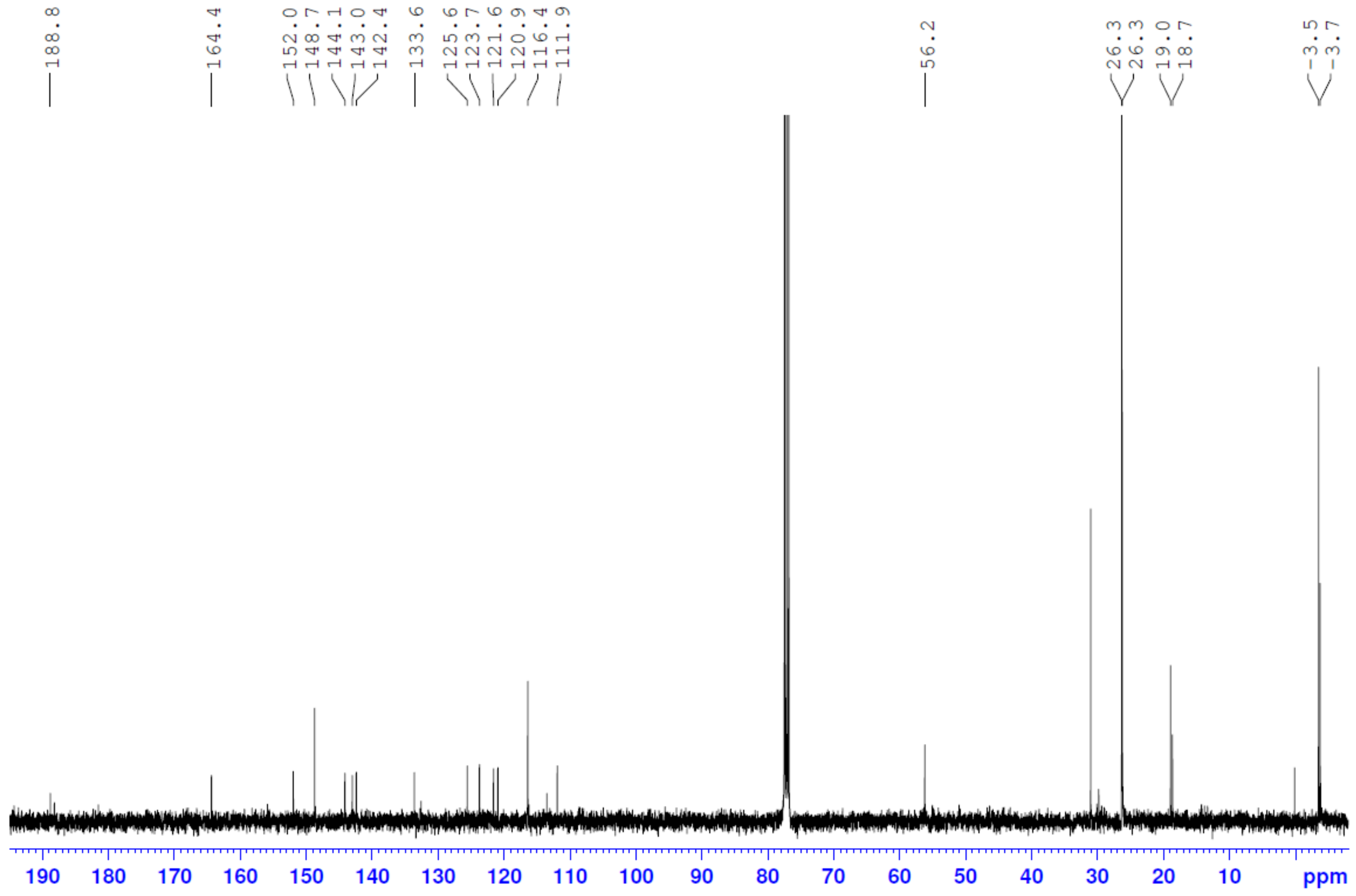

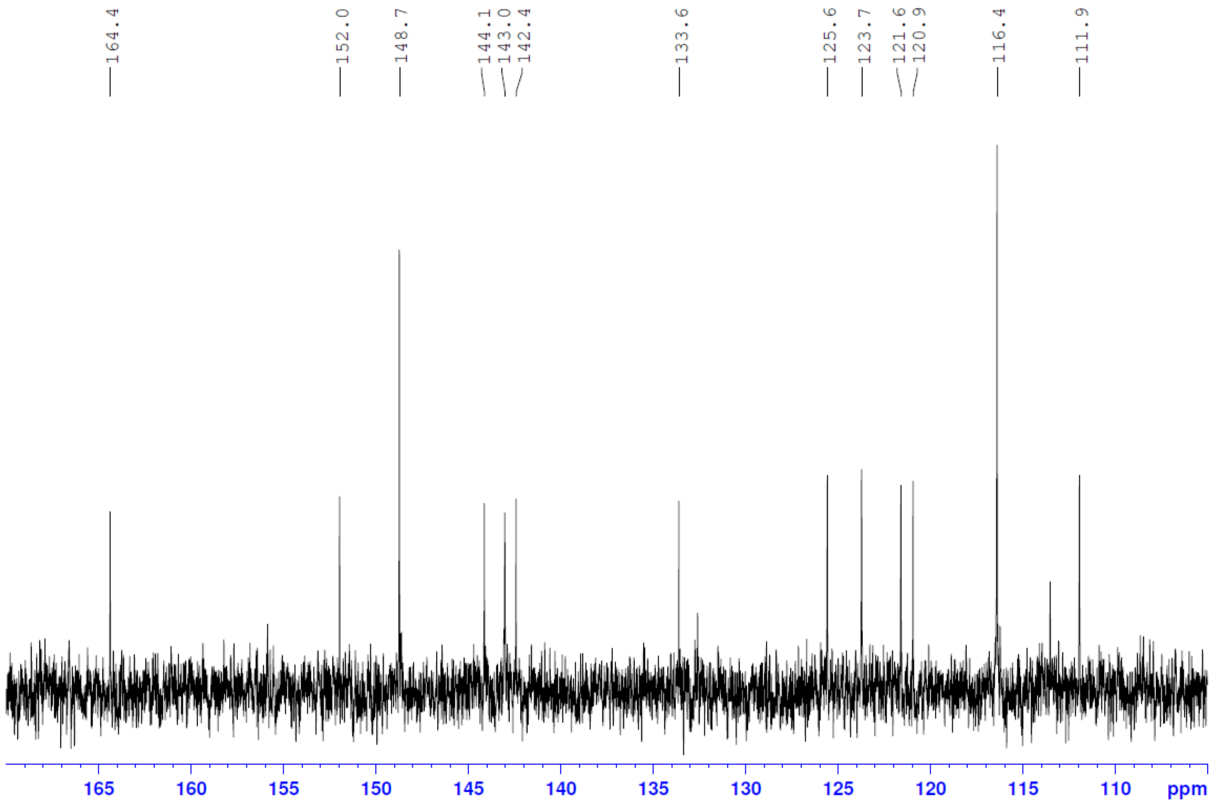

HSQC ¹H-¹³C ($CDCl_3$), δ (ppm)

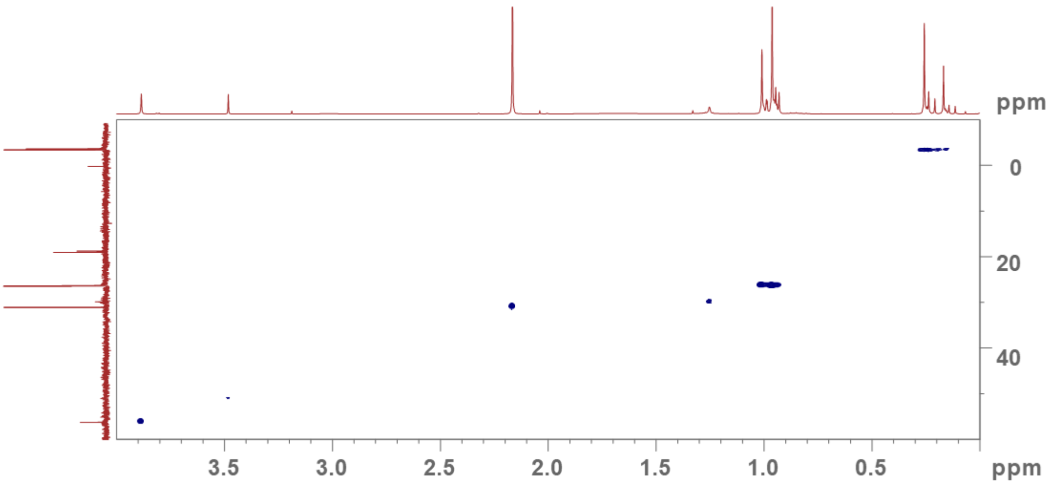

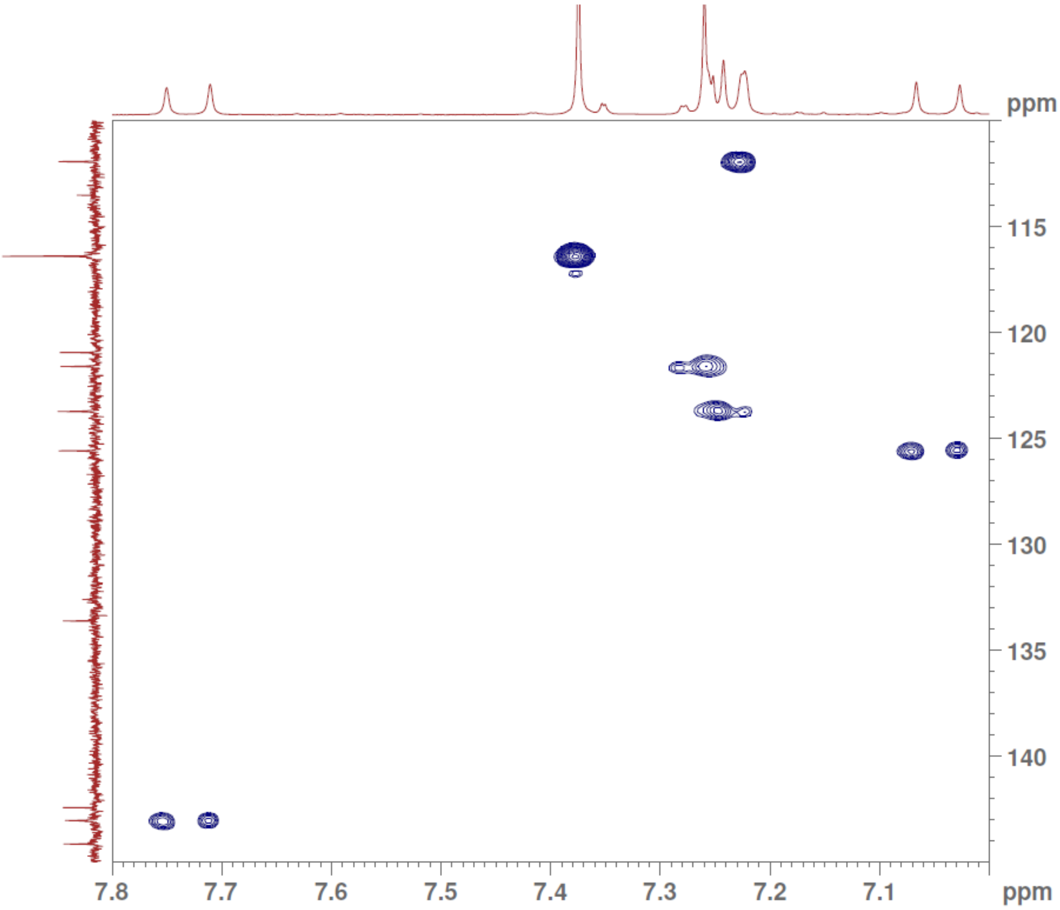

HMBC ^{1}H-^{13}C ($CDCl_3$), δ (ppm)

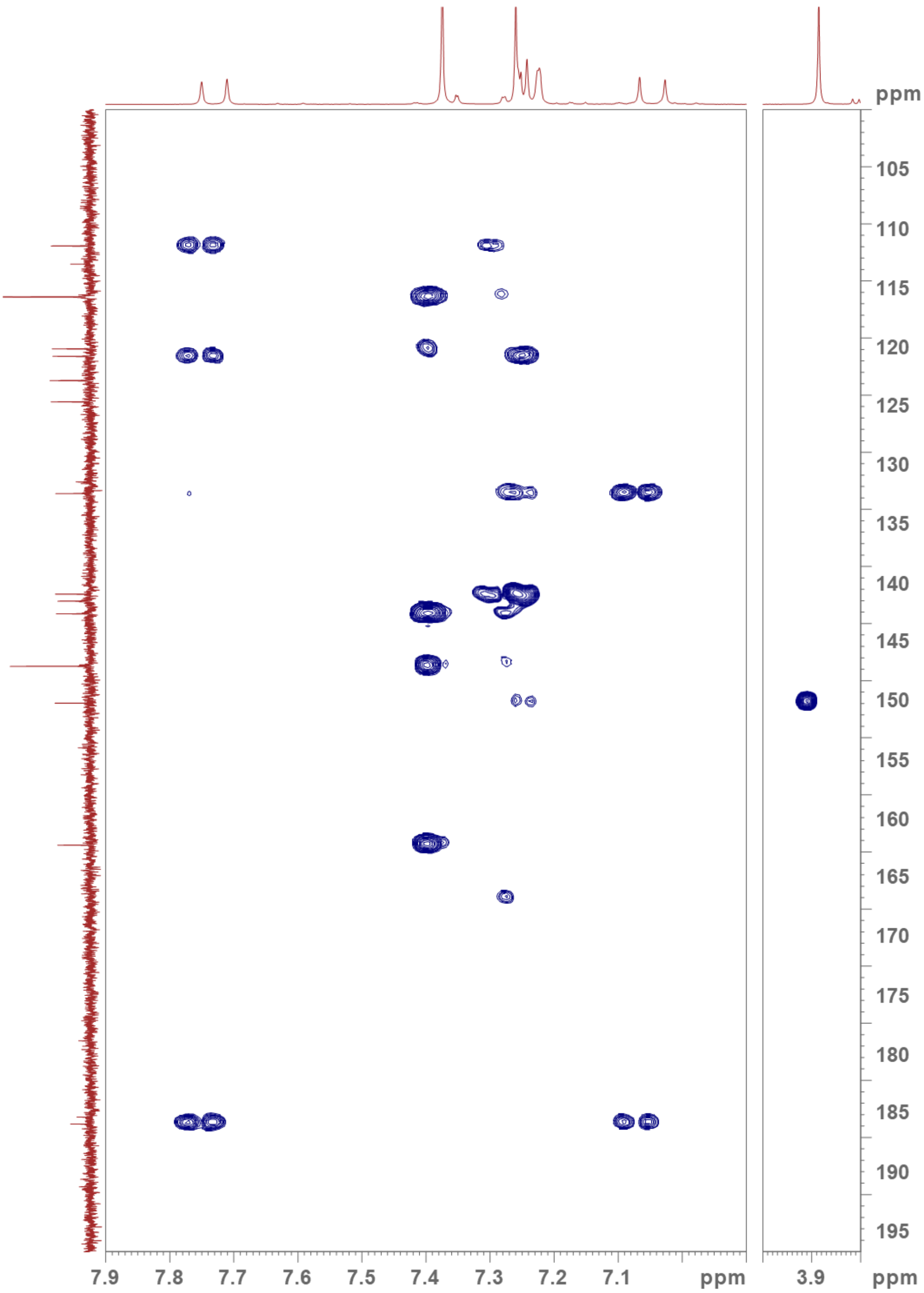

FT-IR (neat), ν (cm^{-1})

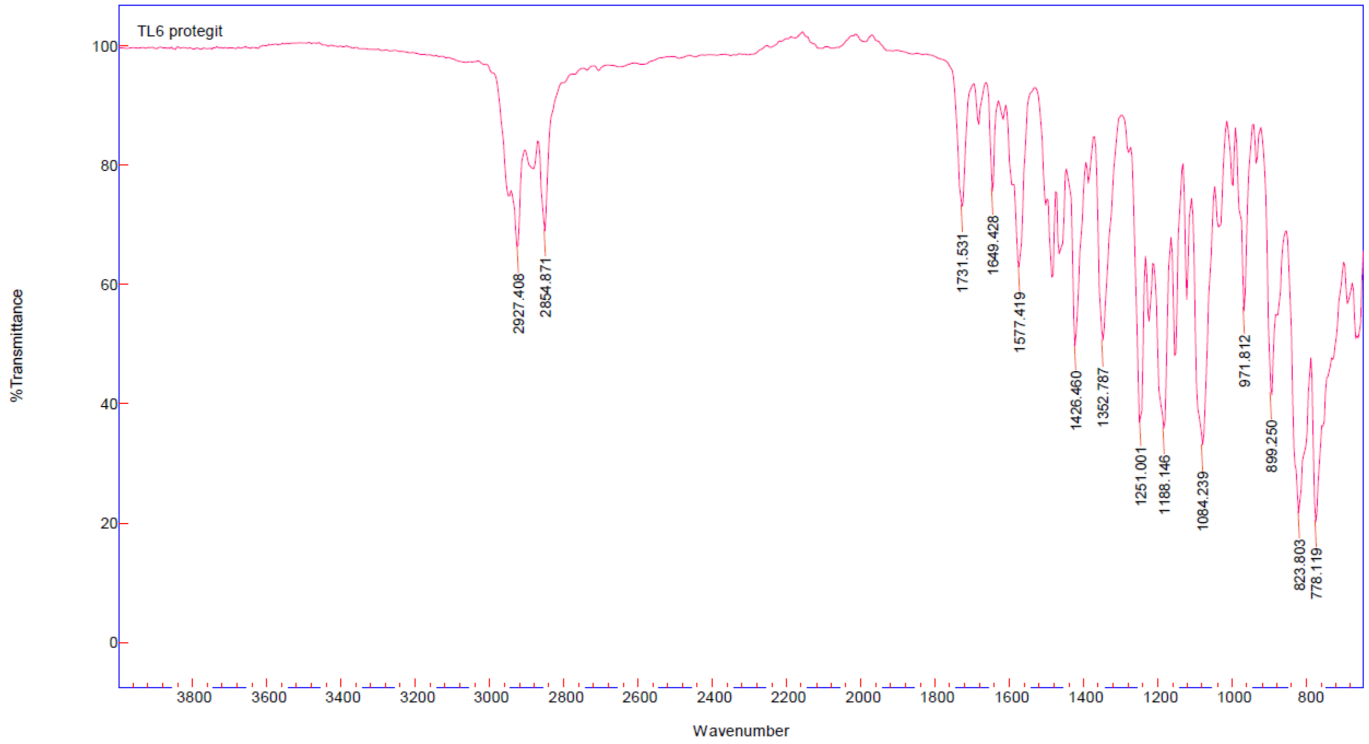

HRMS (*m/z*)

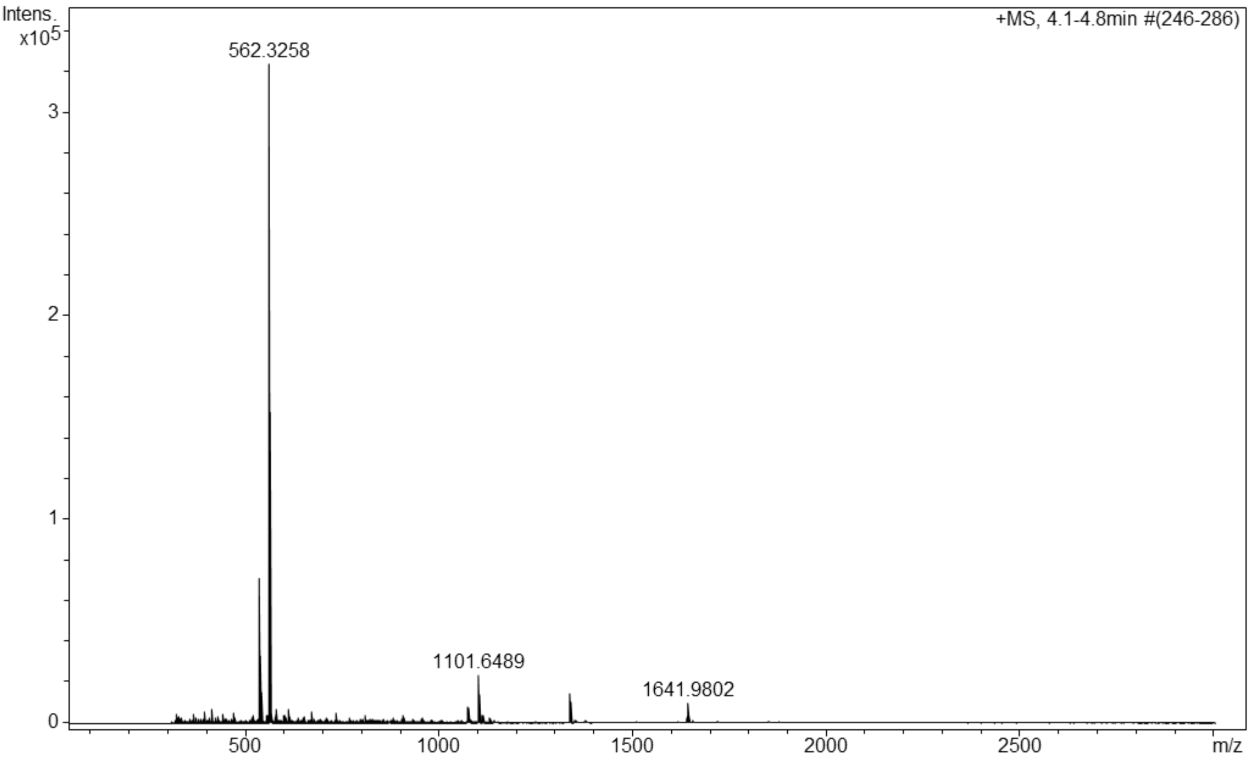

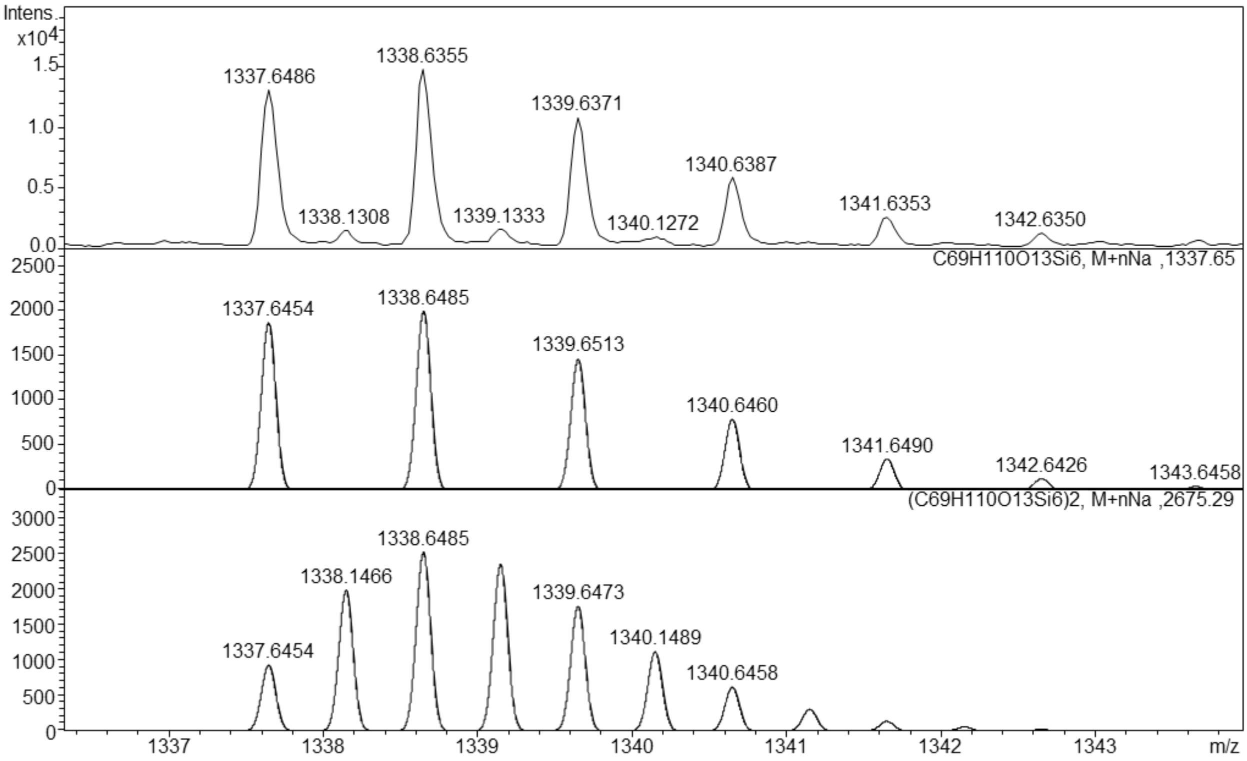

	Molecular formula	Calculated	Found
**$[M+Na]^+$**	$C_{69}H_{110}O_{13}Si_6Na$	1337.6454	1337.6486
**$[2M+2Na]^{2+}$**	$(C_{69}H_{110}O_{13}Si_6)_2Na_2$	1337.6454	1337.6486

2.6. 1,4-Phenylenebis(methylene) bis[3,4,5-tris((*tert*-butyldimethylsilyl)oxy)benzoate]

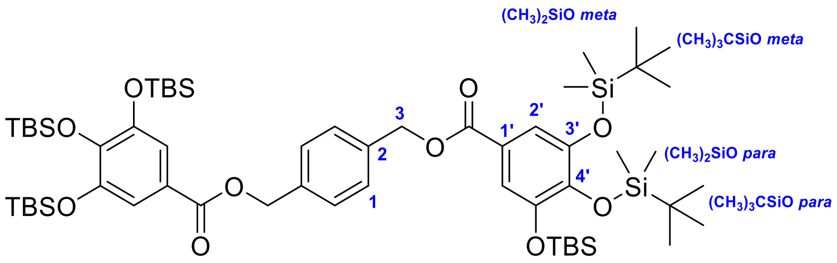

^{1}H-NMR (400 MHz, $CDCl_3$), δ (ppm)

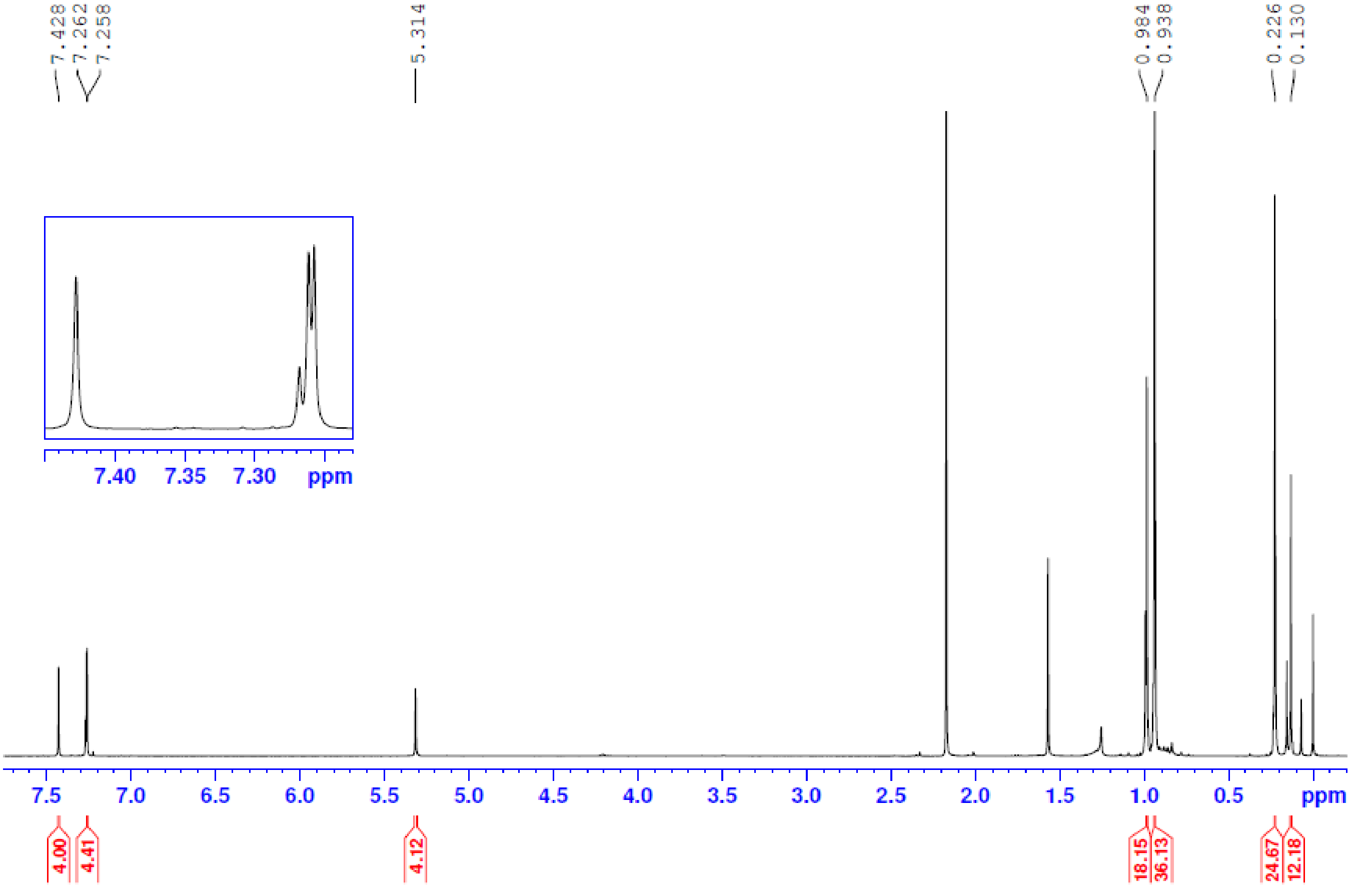

^{13}C-NMR (100 MHz, CDCl$_3$), δ (ppm)

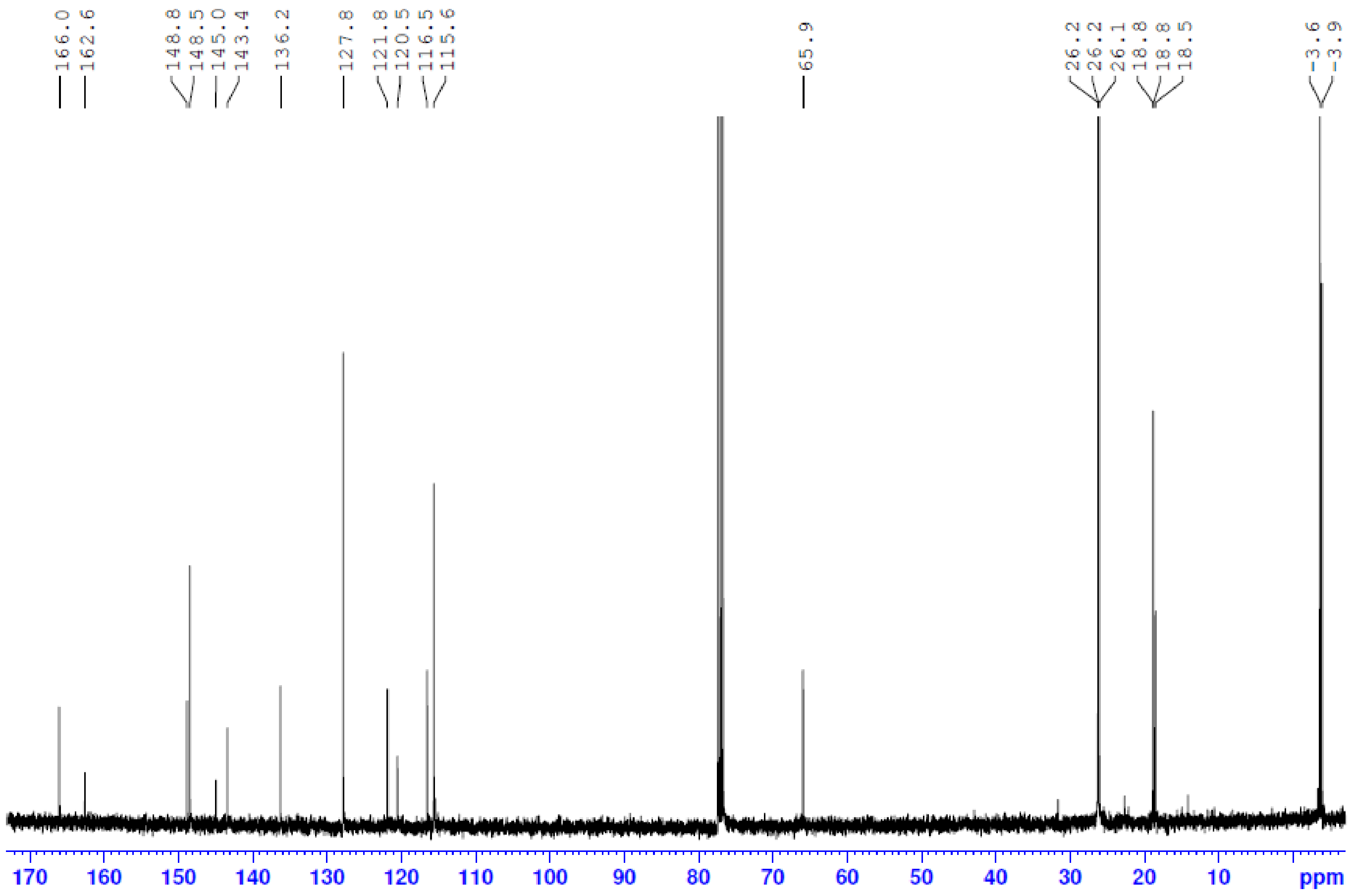

FT-IR (neat), ν (cm^{-1})

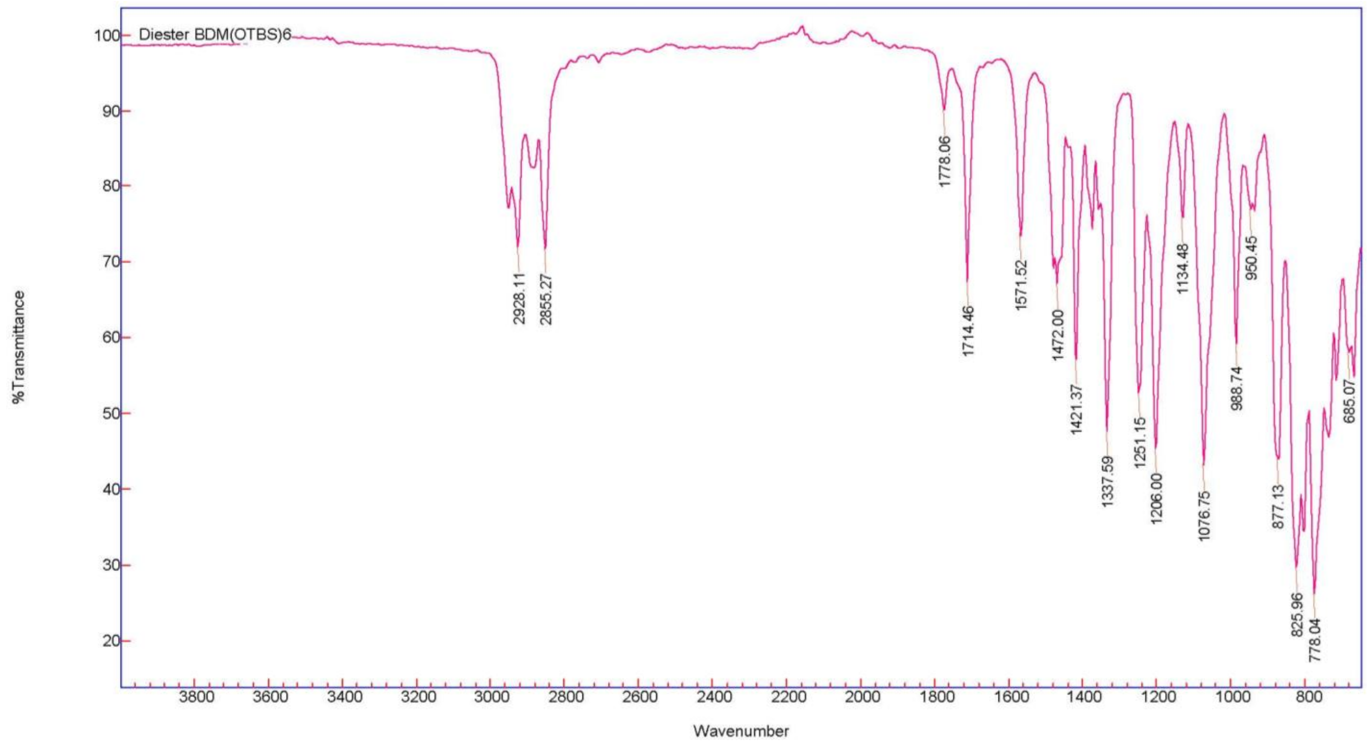

HRMS (*m/z*)

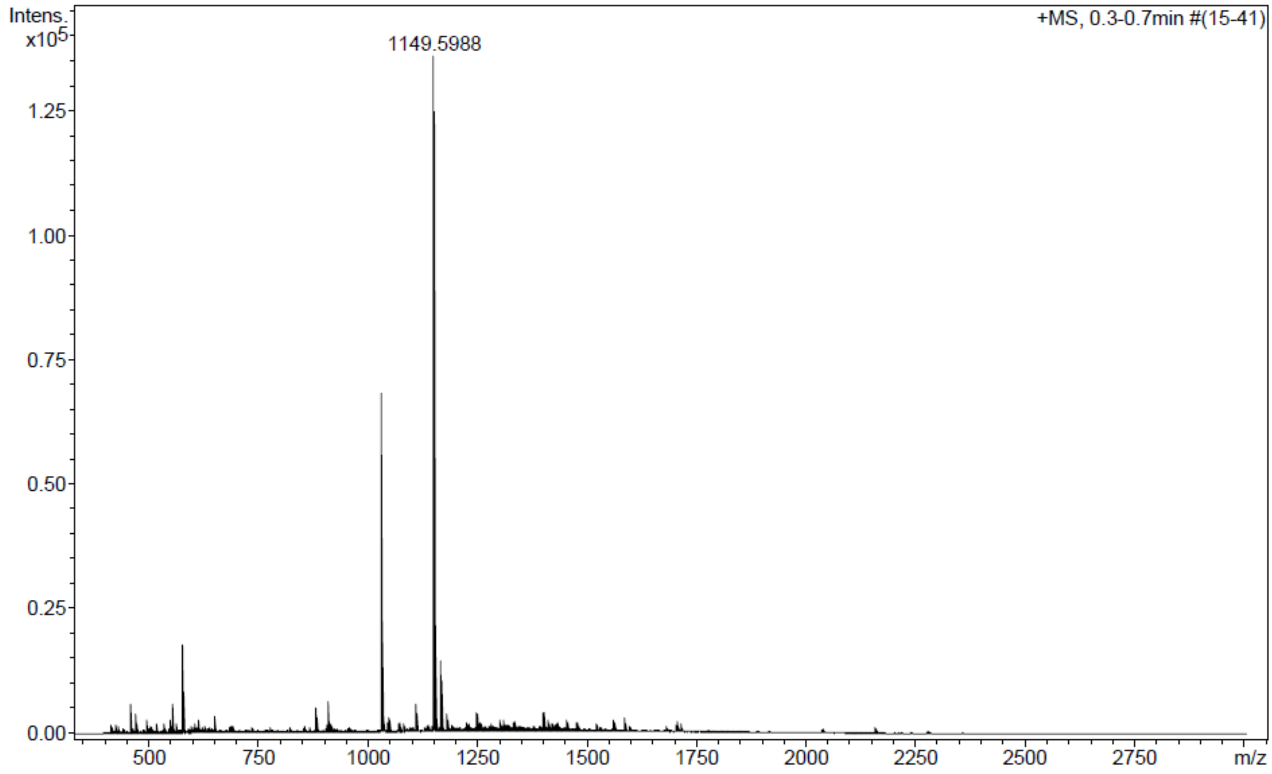

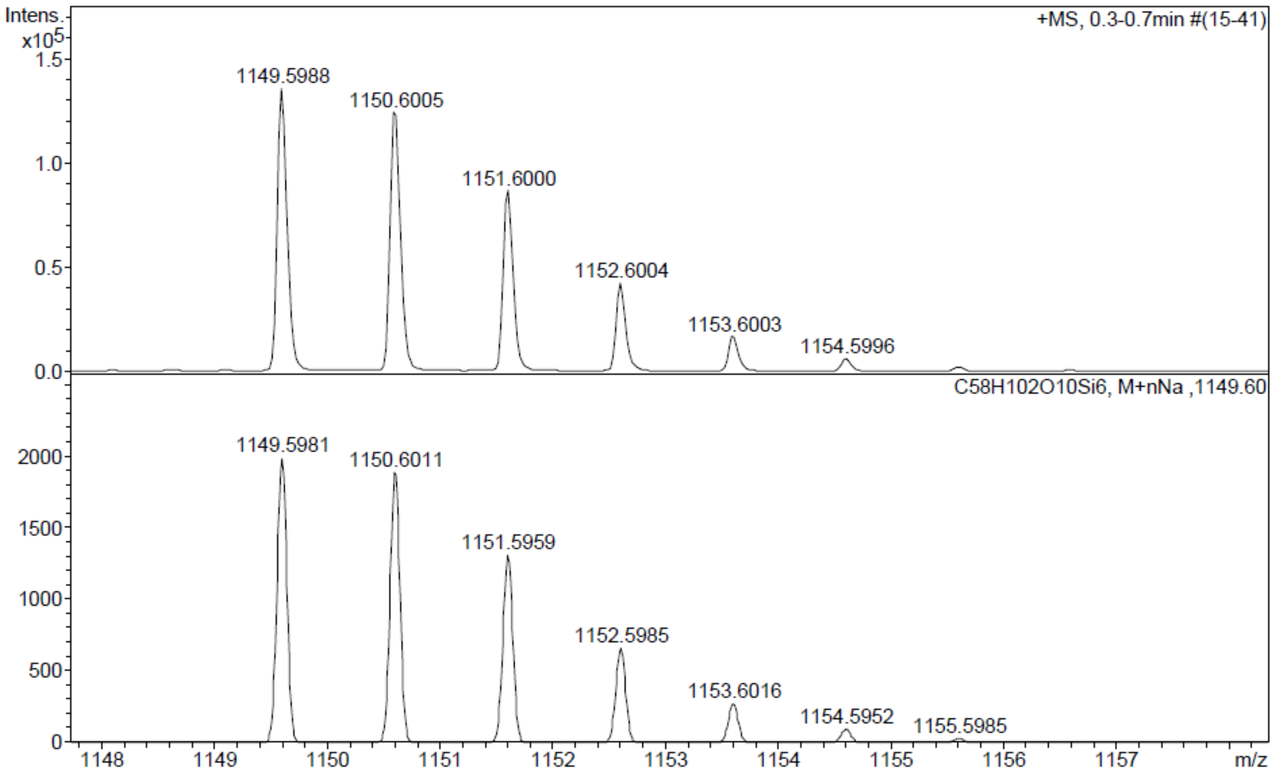

	Molecular formula	Calculated	Found
**[M+Na]$^+$**	$C_{58}H_{102}O_{10}Si_6Na$	1149.5981	1149.5988

2.7. Benzene-1,3,5-triyl tris[3,4,5-tris((*tert*-butyldimethylsilyl)oxy)benzoate]

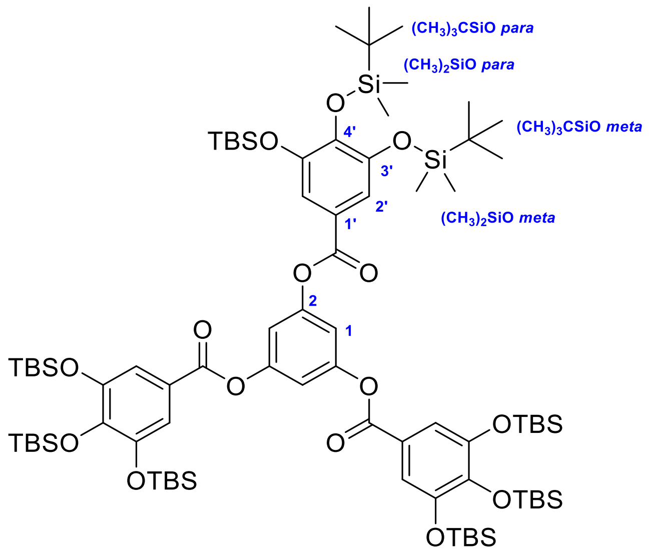

^{1}H-NMR (400 MHz, CDCl3), δ (ppm)

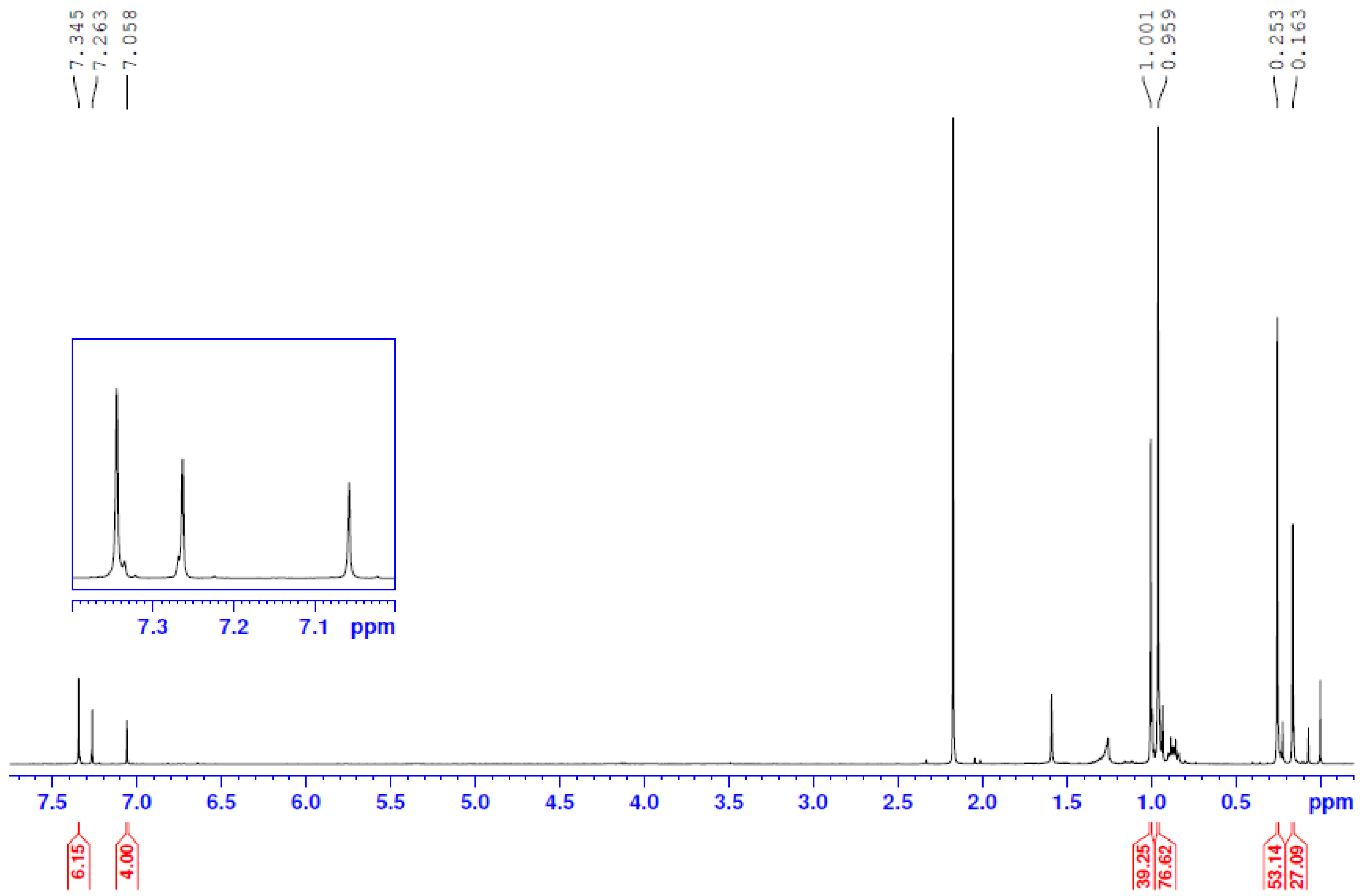

^{13}C-NMR (100 MHz, CDCl$_3$), δ (ppm)

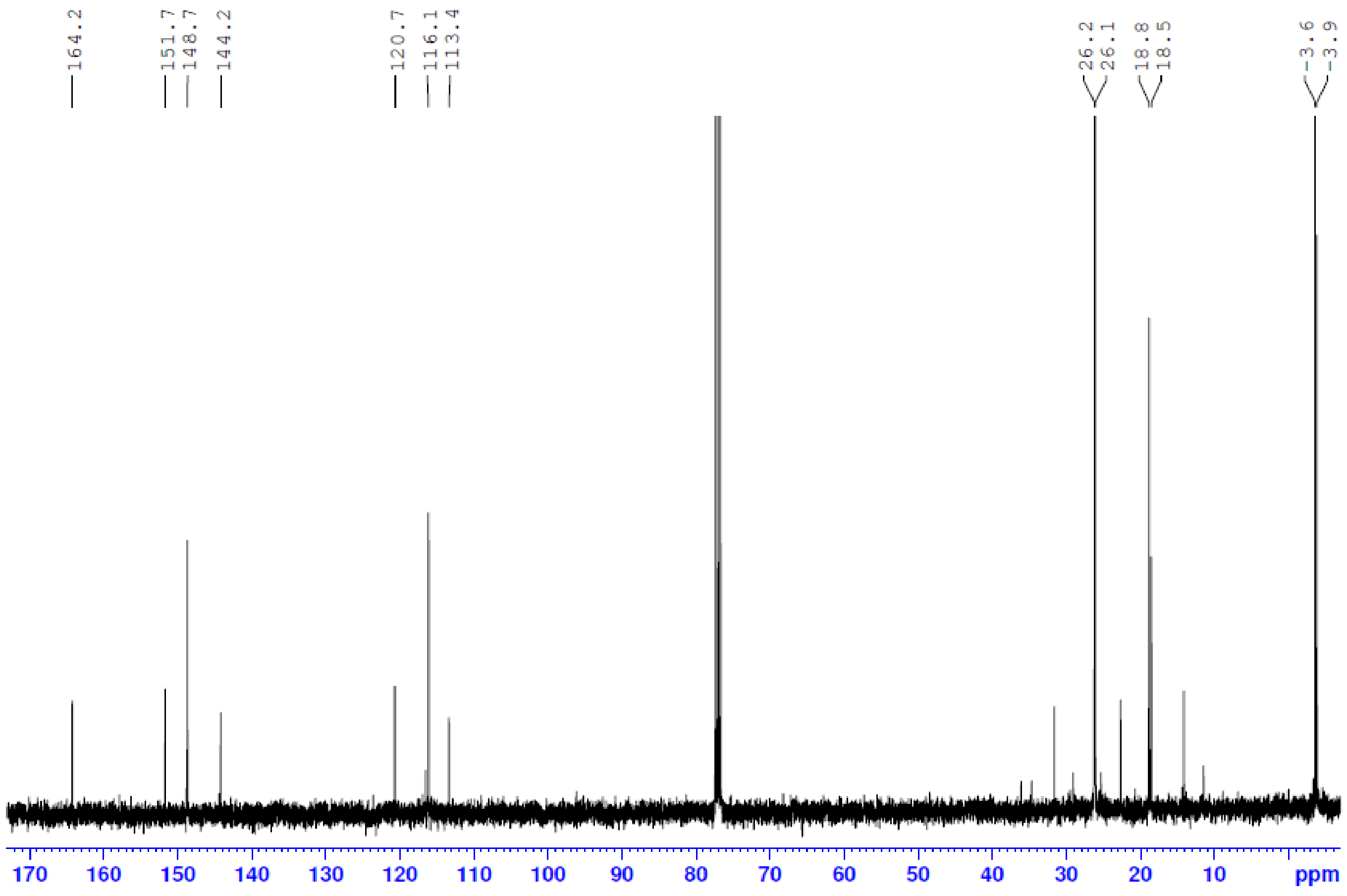

FT-IR (neat), ν (cm^{-1})

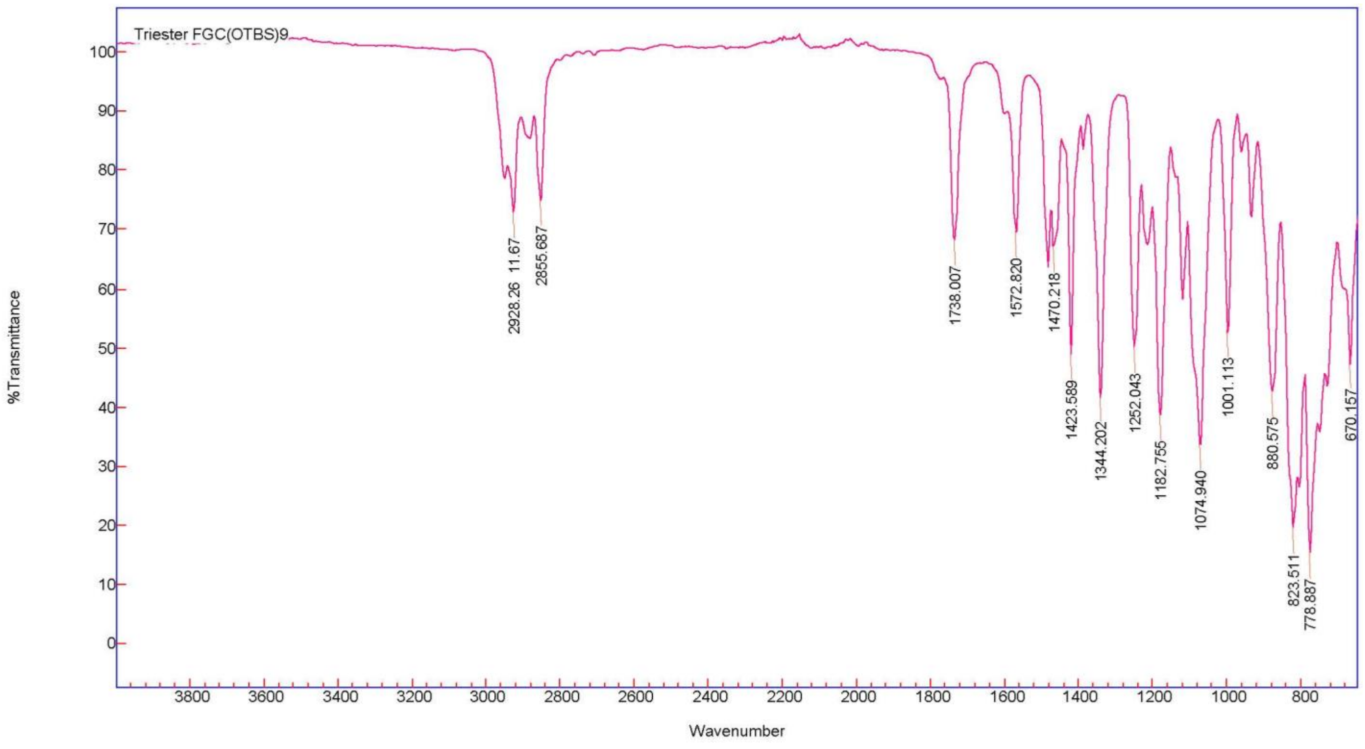

HRMS (*m/z*)

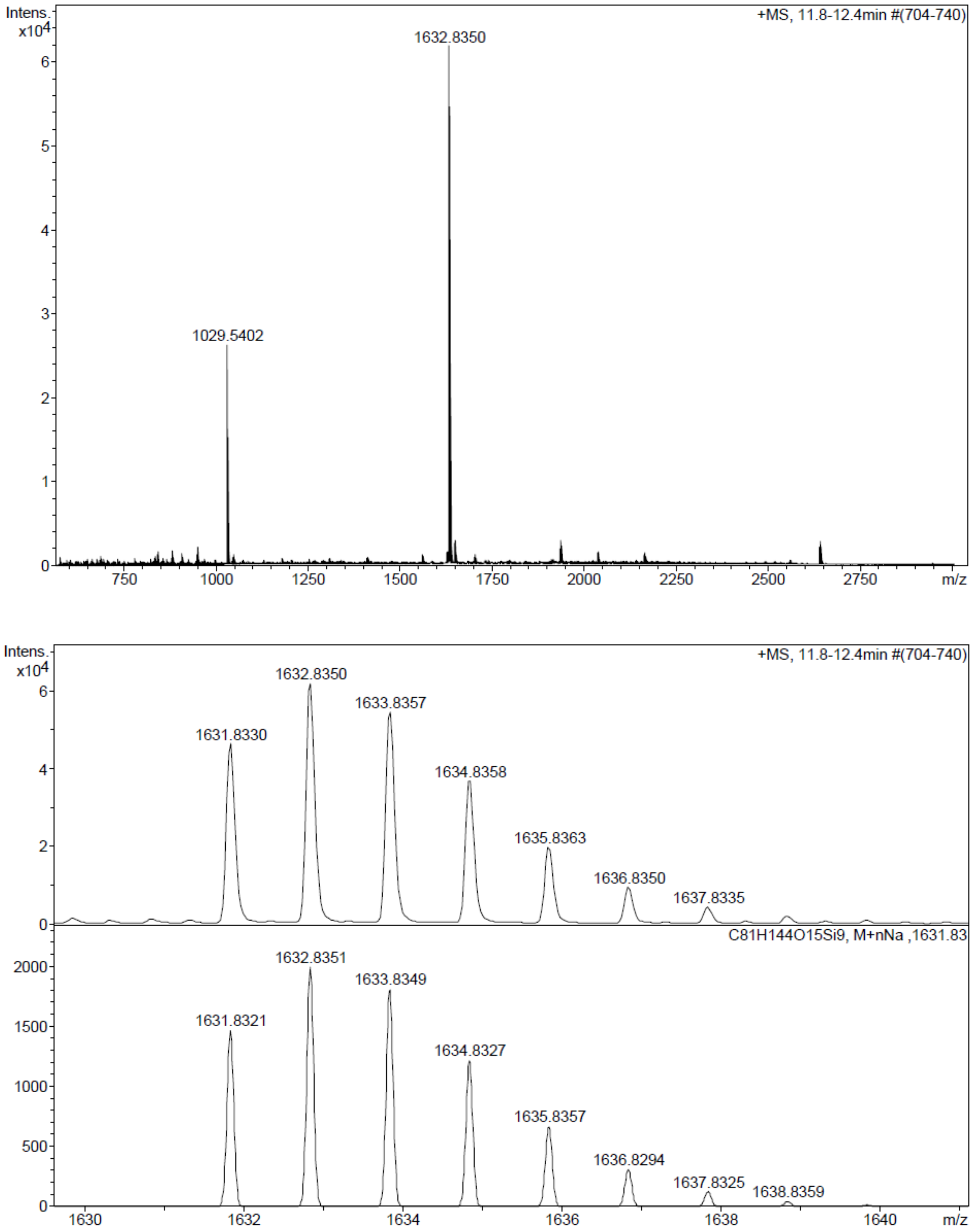

	Molecular formula	Calculated	Found
**$[M+Na]^+$**	$C_{81}H_{144}O_{15}Si_9Na$	1631.8321	1631.8330

2.8. ((1*E*,3*Z*,6*E*)-3-Hydroxy-5-oxohepta-1,3,6-triene-1,7-diyliden)bis(2-methoxy-4,1-phenylene) bis[3,4,5-tris((*tert*-butyldimethylsilyl)oxy)benzoate]

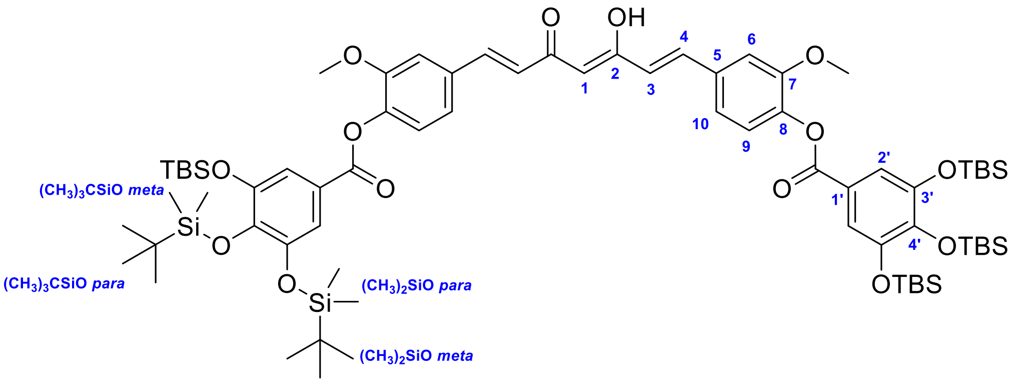

^{1}H-NMR (400 MHz, CDCl$_3$), δ (ppm)

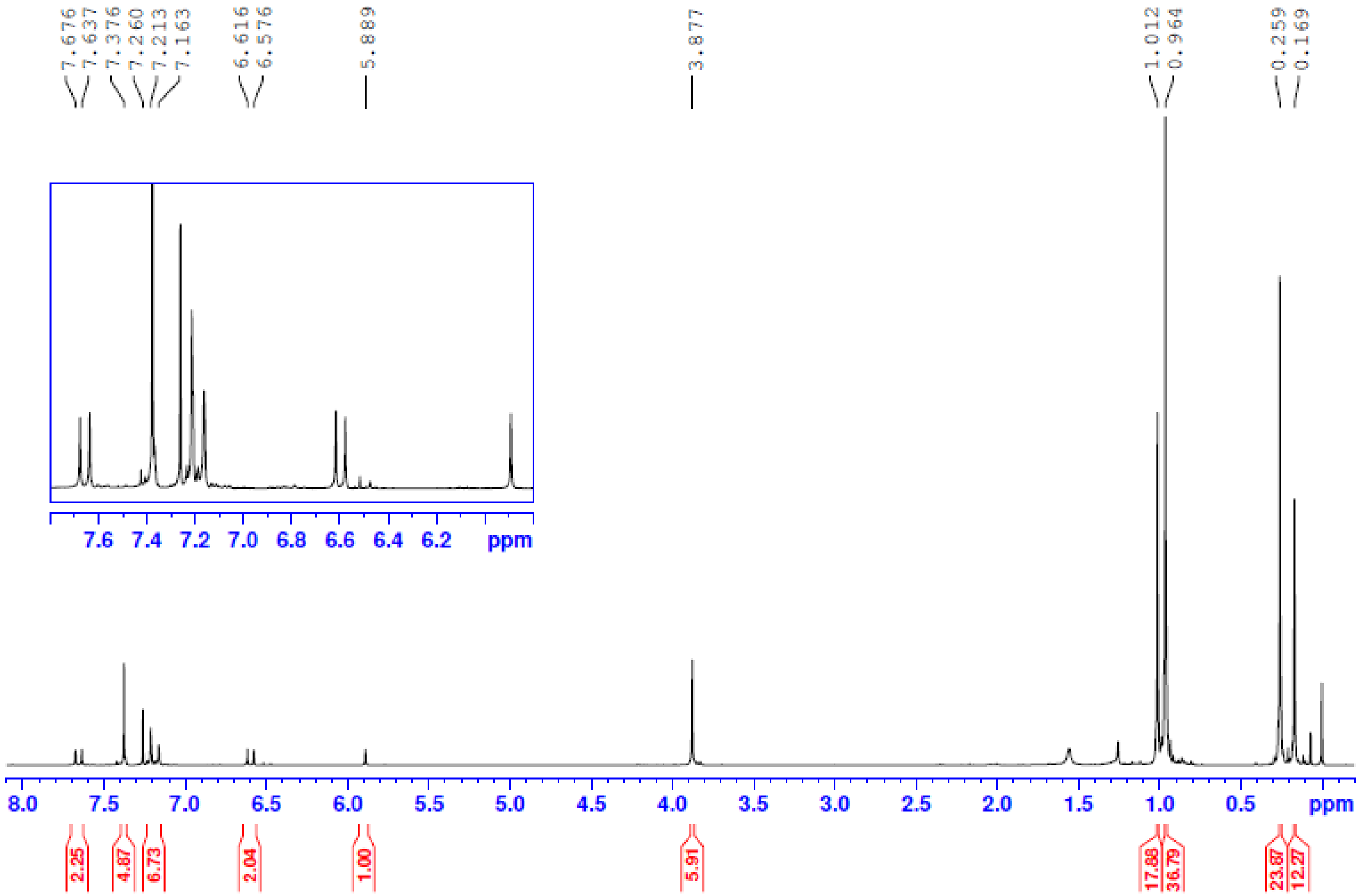

^{13}C-NMR (100 MHz, $CDCl_3$), δ (ppm)

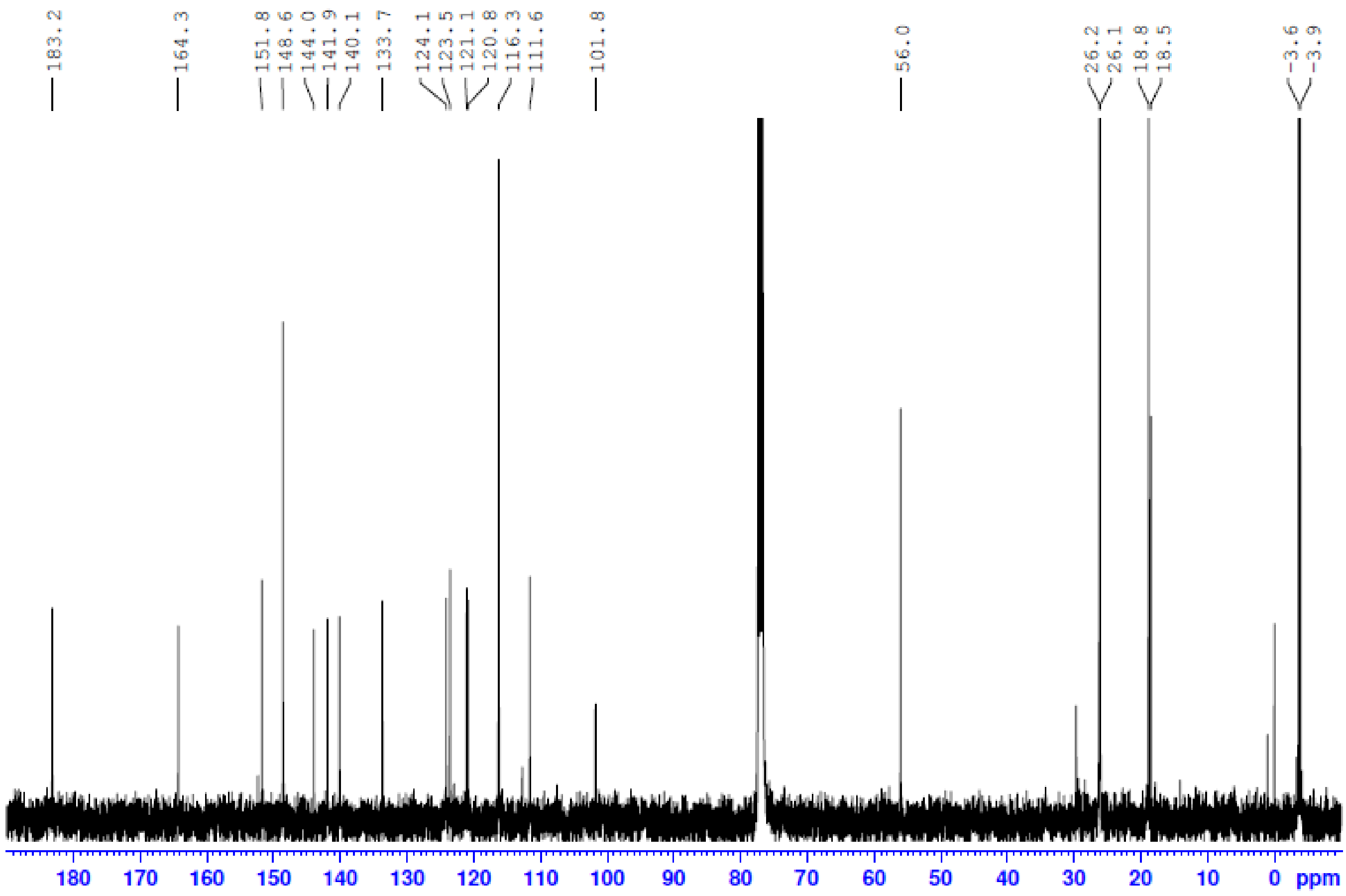

FT-IR (neat), ν (cm^{-1})

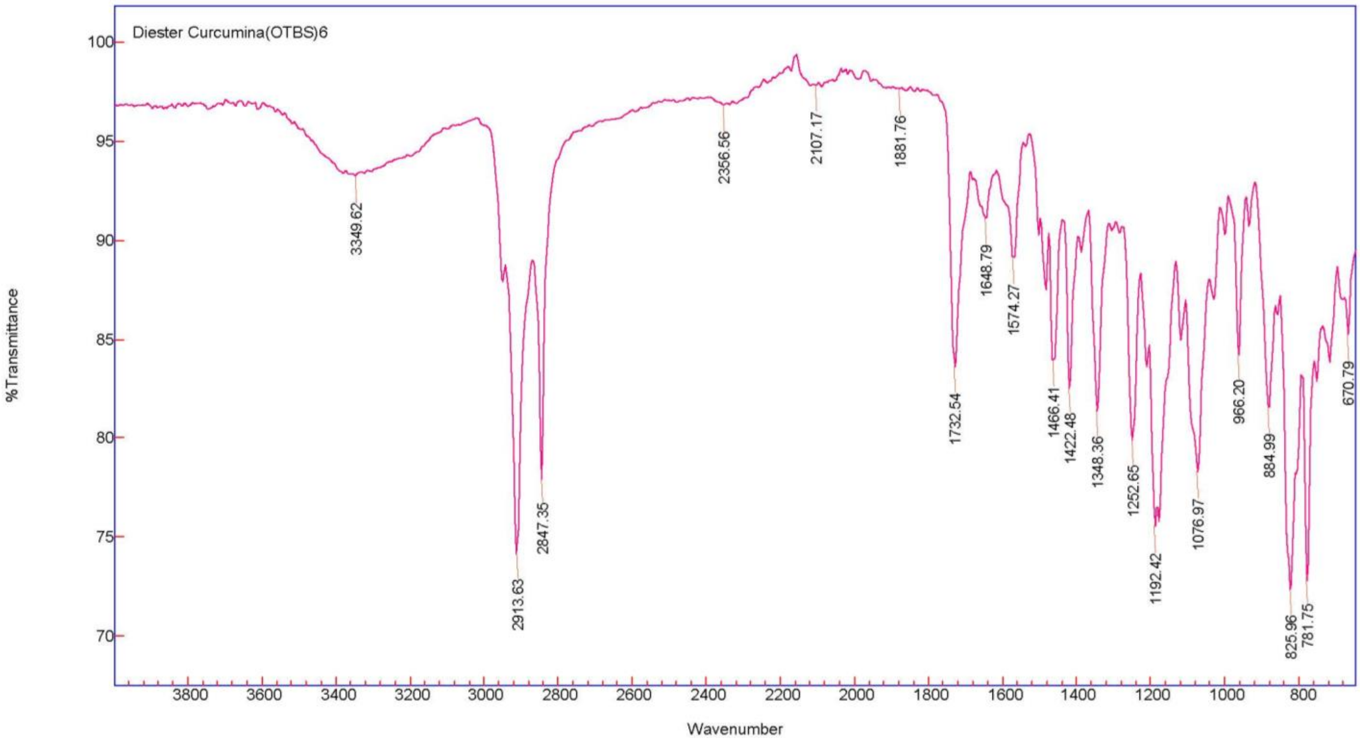

HRMS (*m/z*)

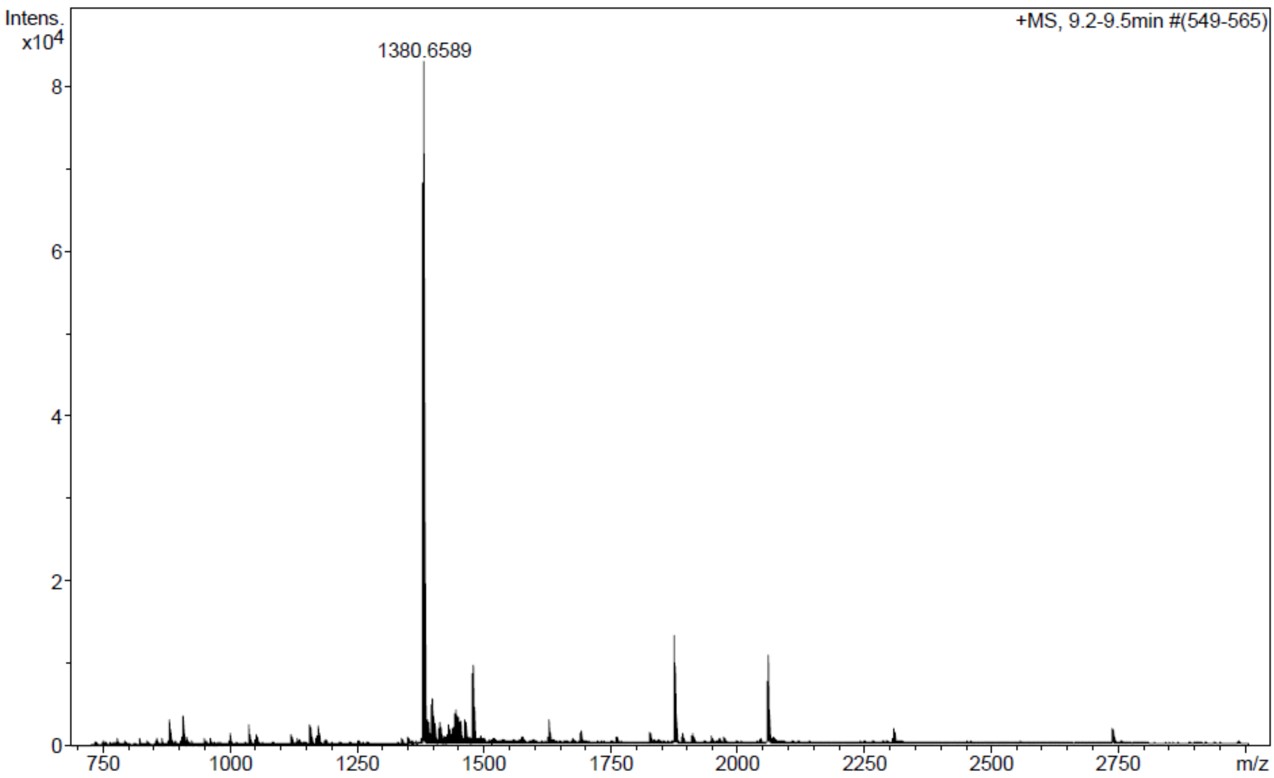

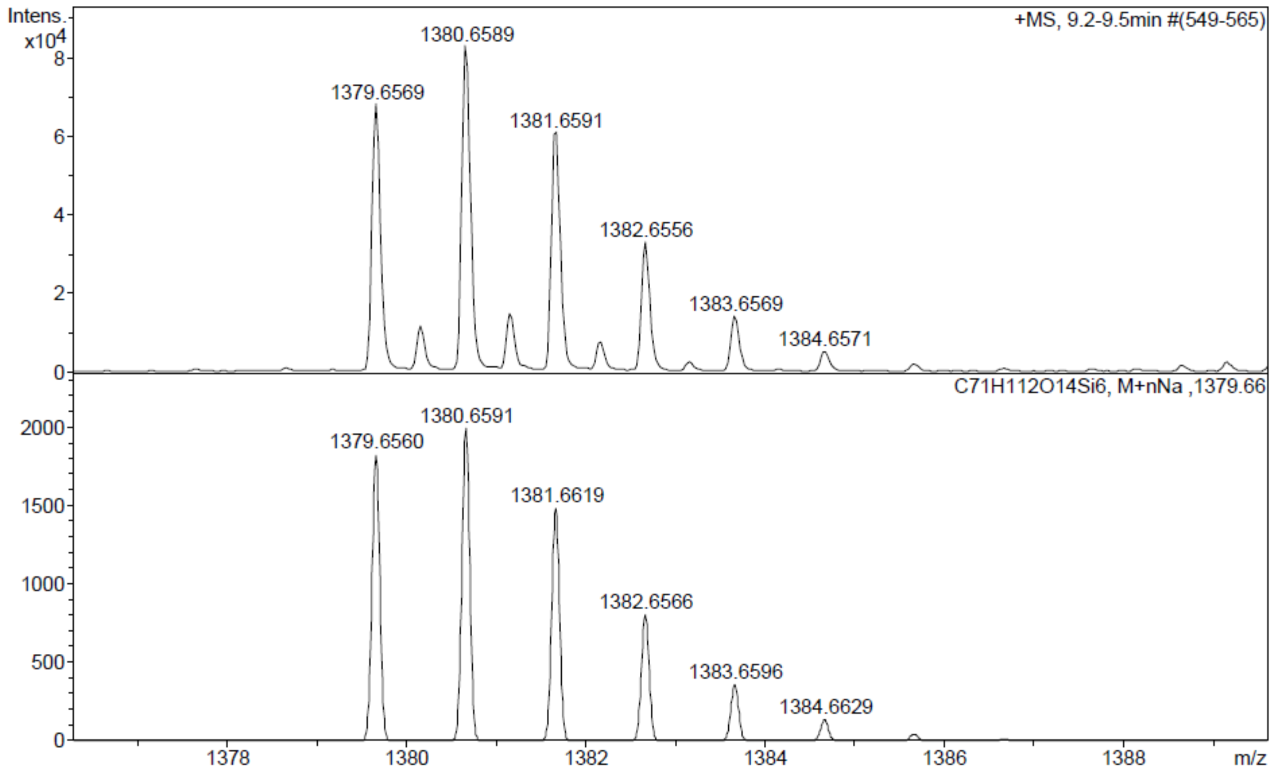

	Molecular formula	Calculated	Found
**$[M+Na]^+$**	$C_{71}H_{112}O_{14}Si_6Na$	1379.6560	1379.6569

2.9. 1,4-Phenylenebis(methylene) bis(3,4,5-trihydroxybenzoate) (TL1)

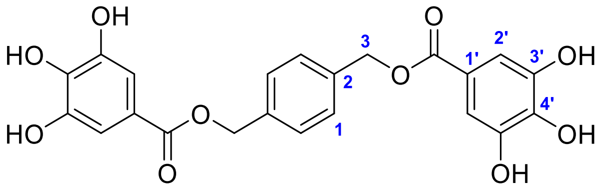

^{1}H-NMR (400 MHz, CD_3OD), δ (ppm)

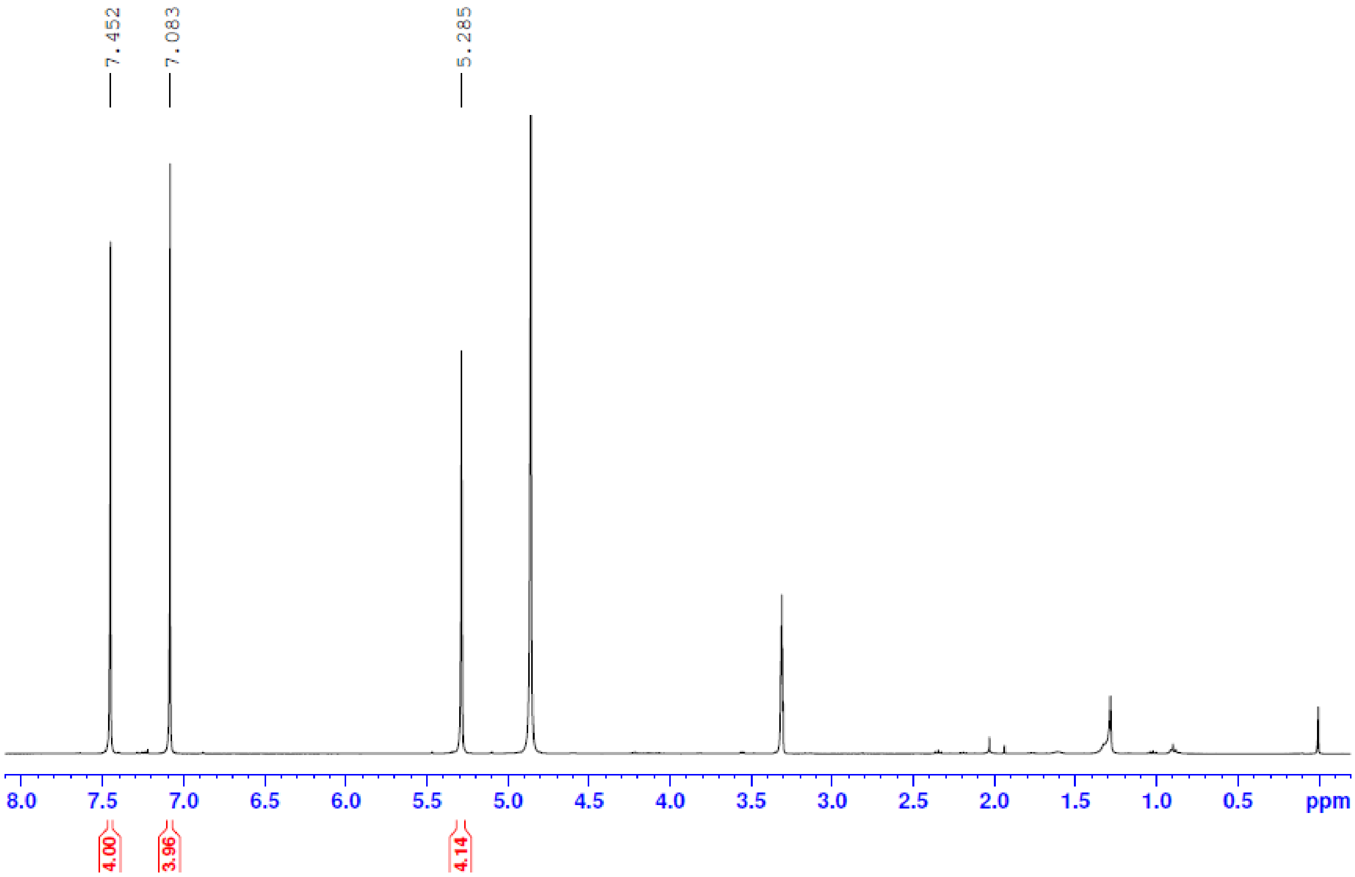

^{13}C-NMR (100 MHz, CD_3OD), δ (ppm)

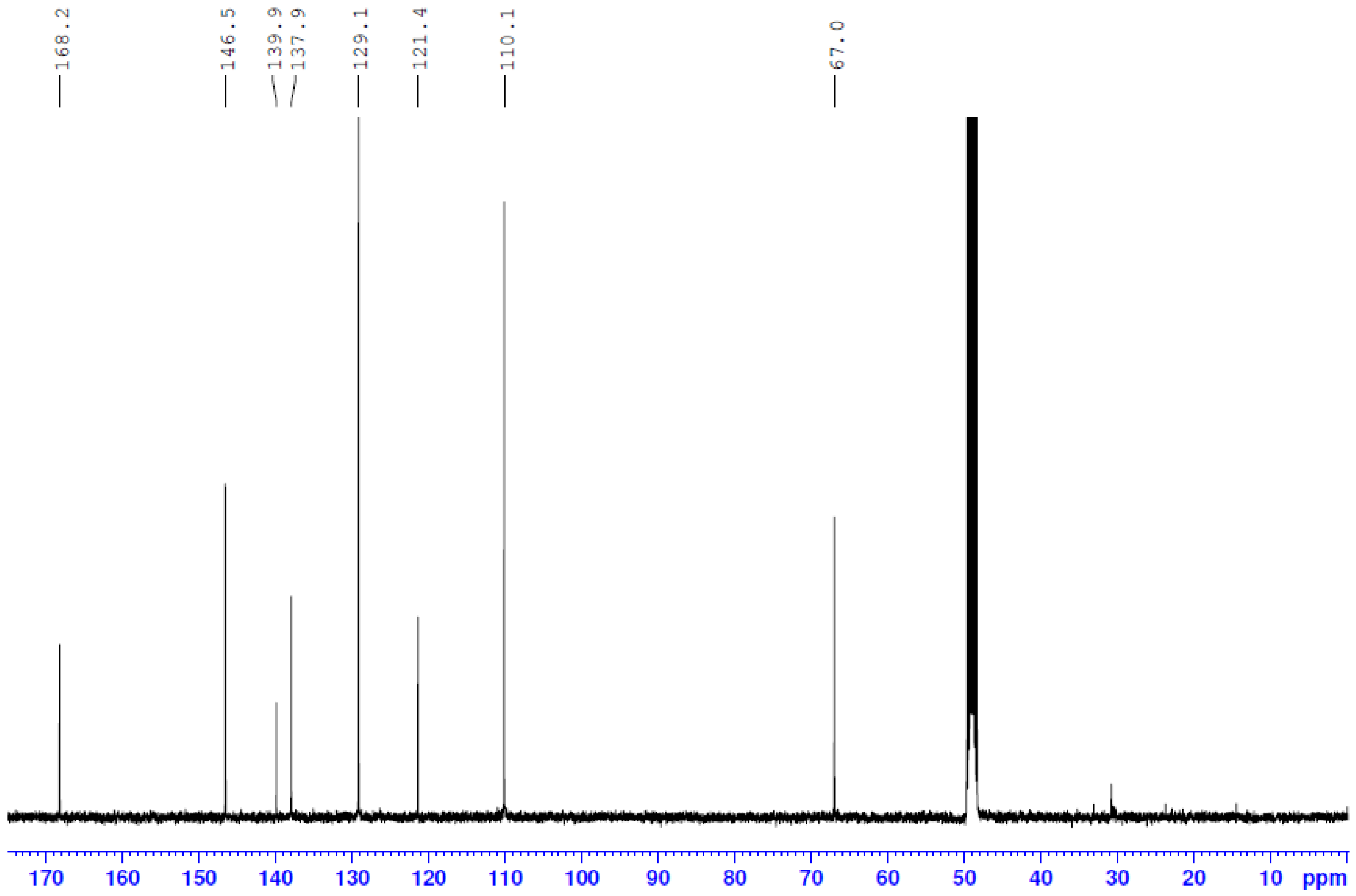

HSQC ^{1}H-^{13}C ($CDCl_3$), δ (ppm)

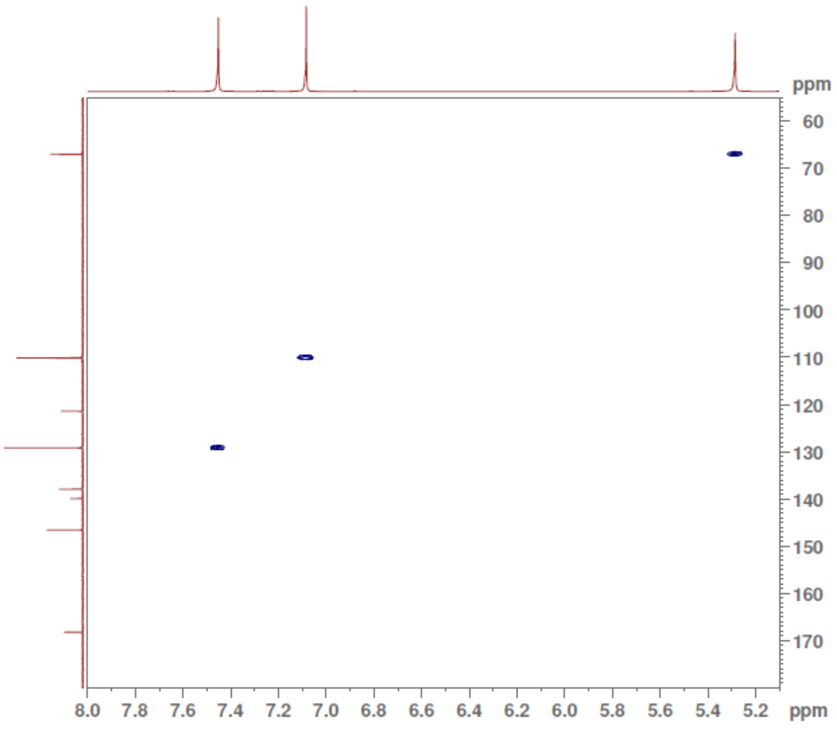

HMBC ^{1}H-^{13}C ($CDCl_3$), δ (ppm)

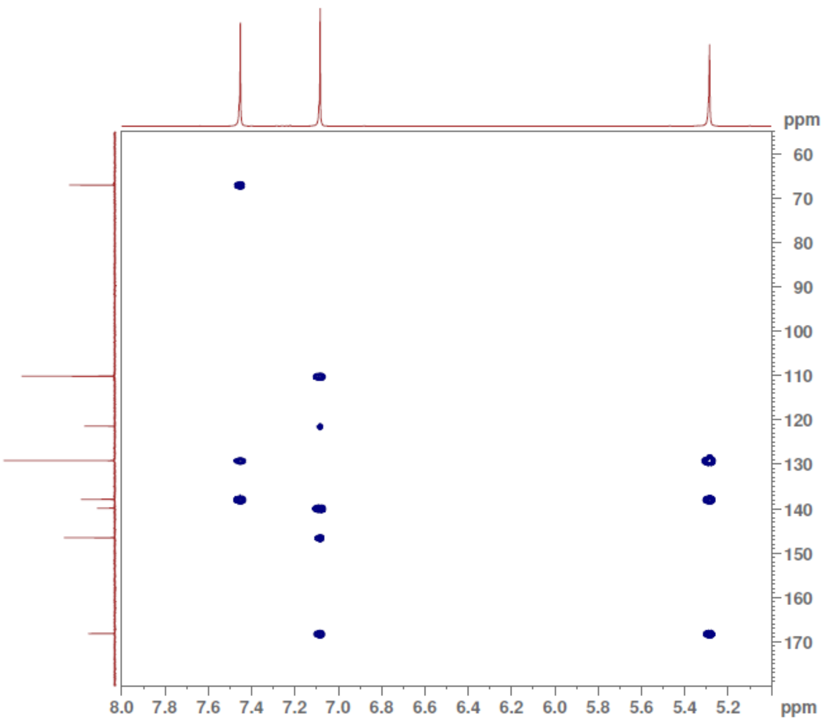

FT-IR (neat), ν (cm^{-1})

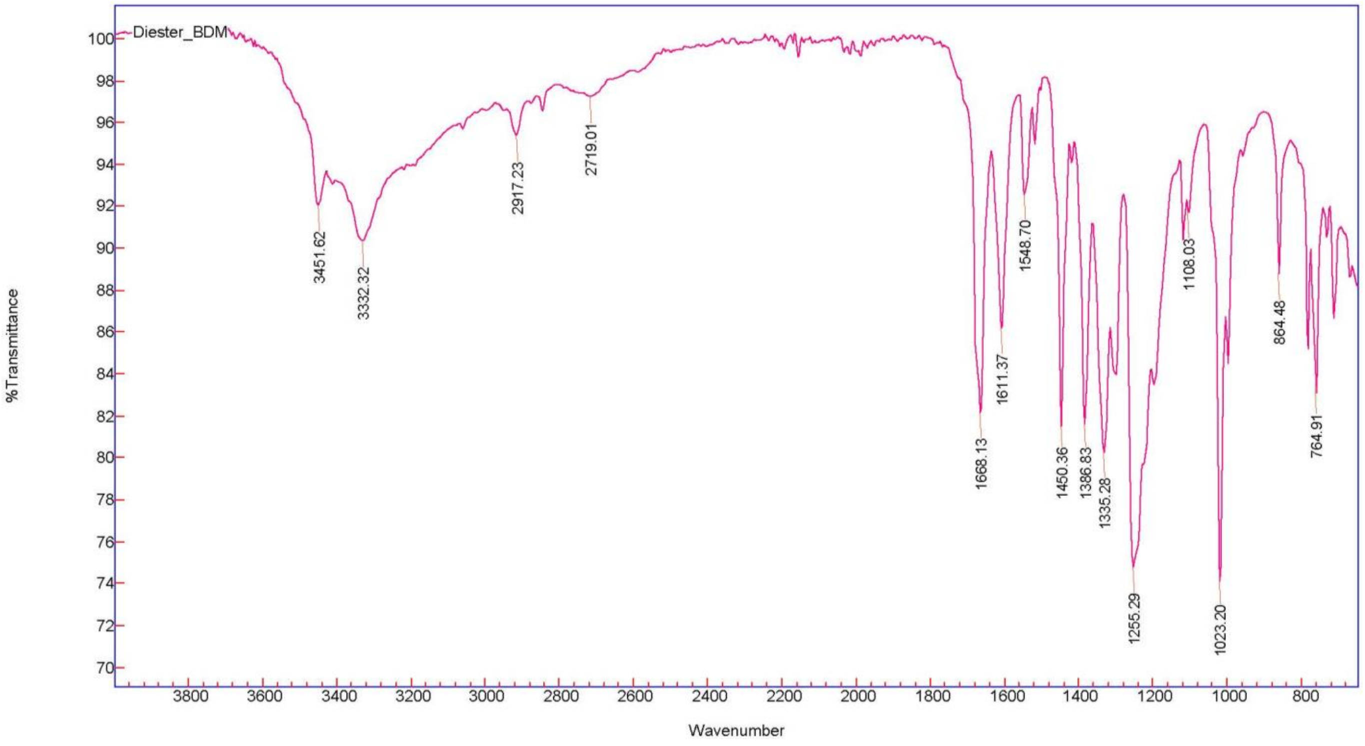

UV-Vis (CH_3CN), λ (nm)

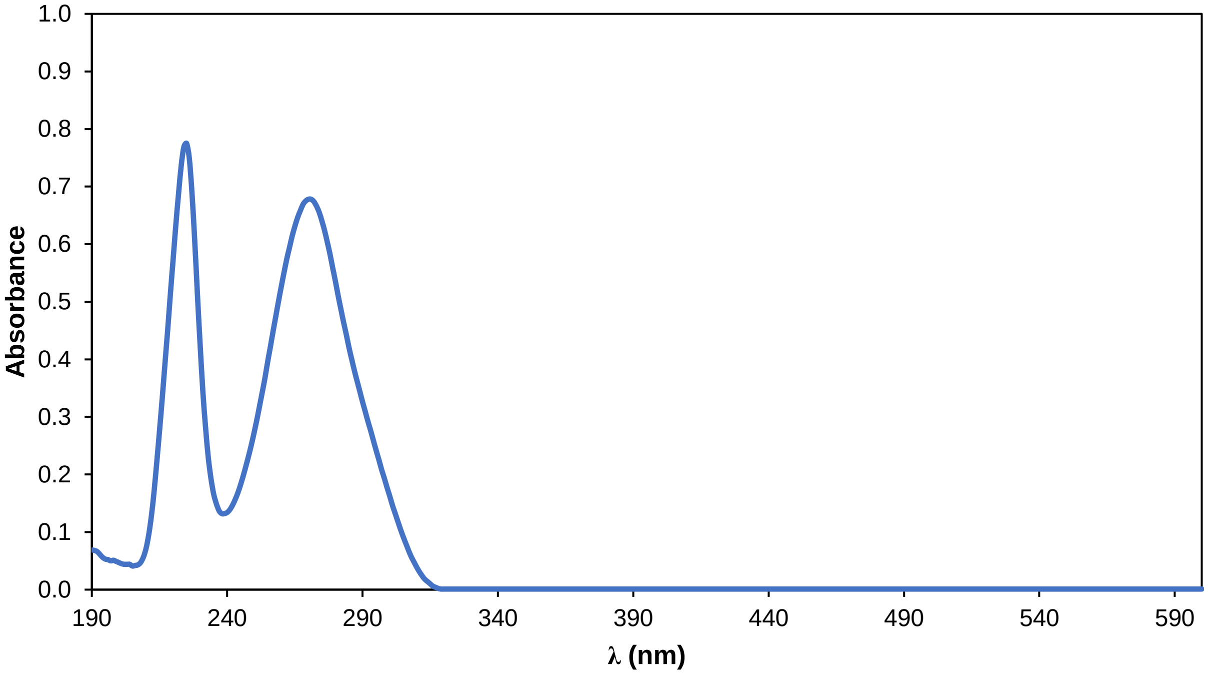

HPLC (λ = 220 nm)

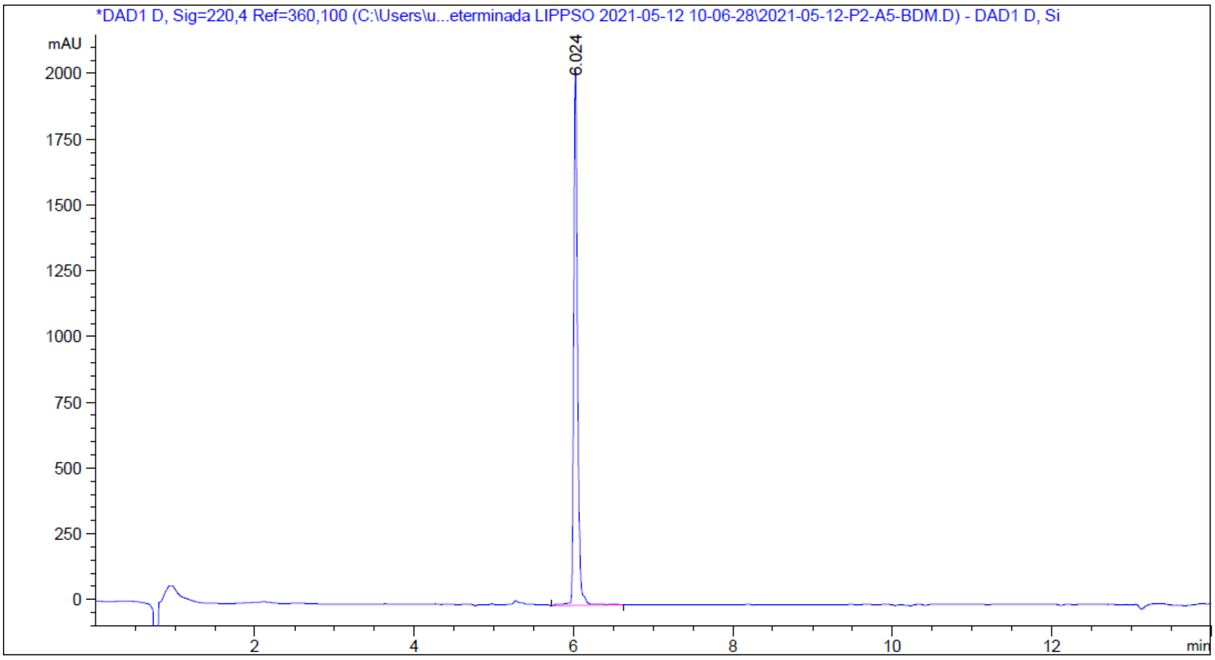

Peak #	RetTime [min]	Type	Width [min]	Area [mAU*s]	Height [mAU]	Area %
1	6.024	BV R	0.0510	6825.30957	2041.29993	100.0000
Totals :				6825.30957	2041.29993	

ESI-MS (*m/z*)

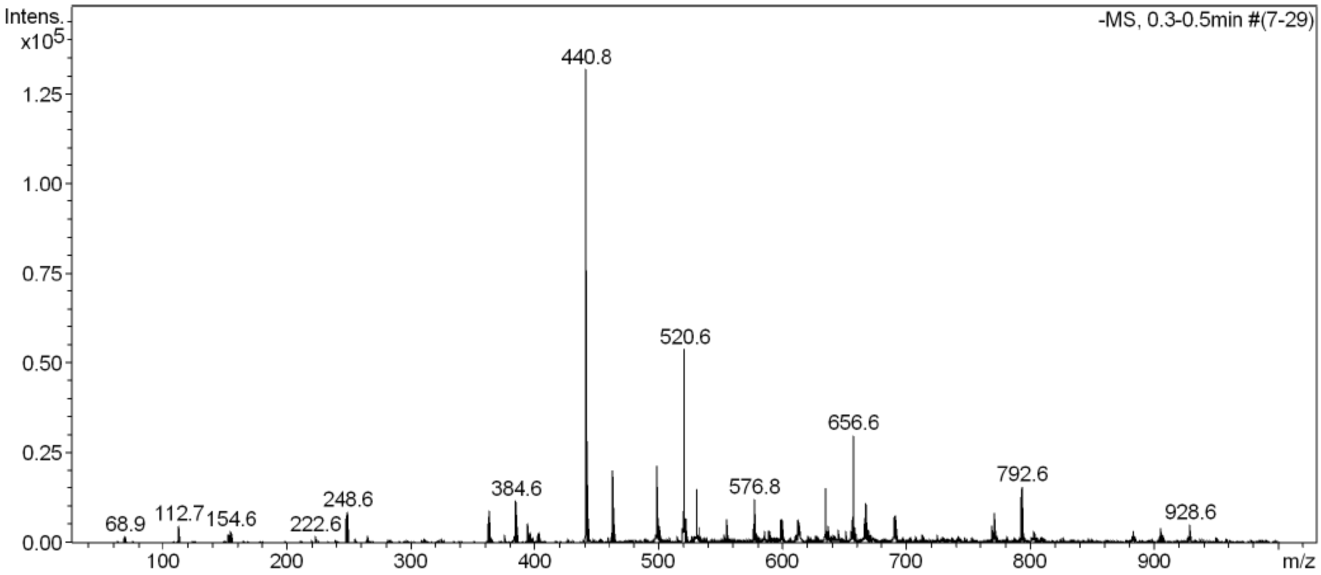

Intens.
x10^5
-MS, 0.3-0.5min #(7-29)
1.25
1.00
0.75
0.50
0.25
0.00
440.8
520.6
656.6
792.6
576.8
384.6
928.6
248.6
68.9
112.7
154.6
222.6
100
200
300
400
500
600
700
800
900
m/z

HRMS (*m/z*)

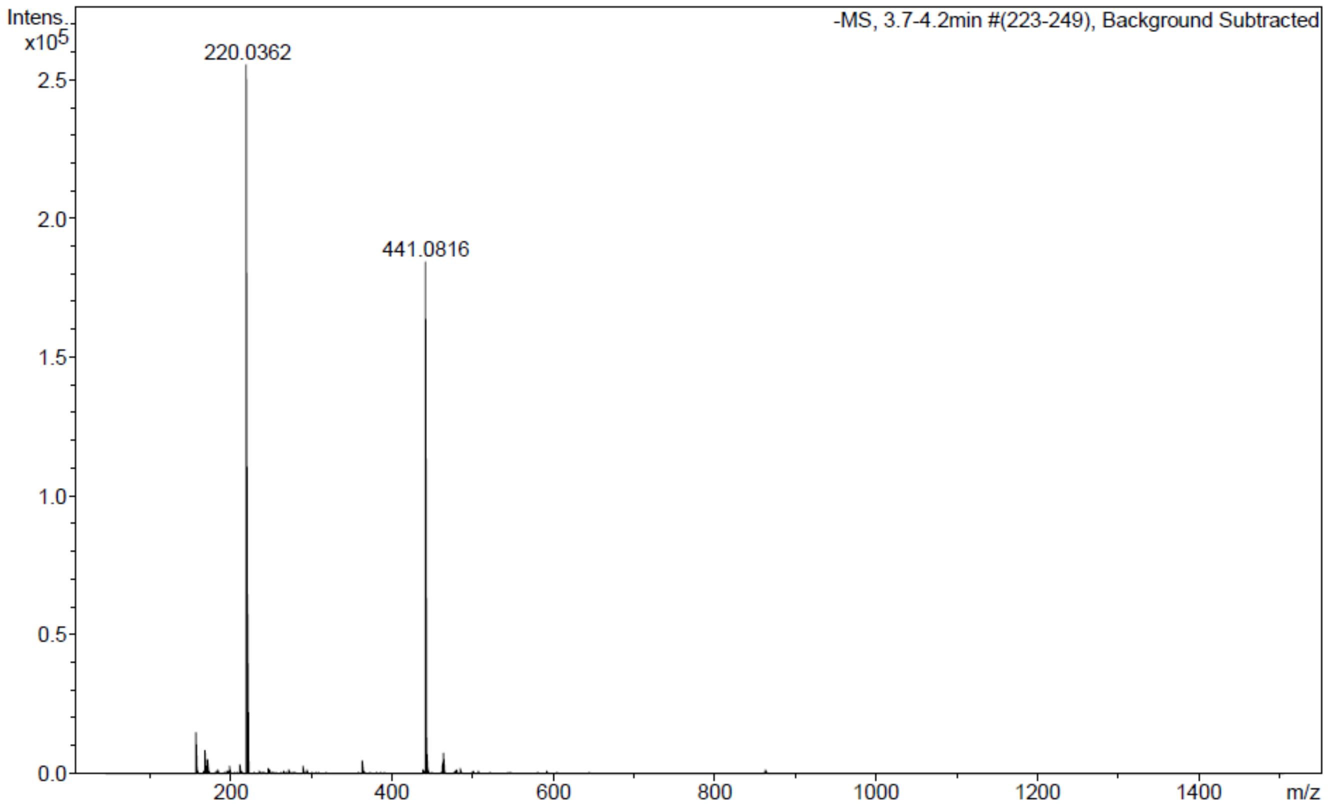
Intens.
x10^5
-MS, 3.7-4.2min #(223-249), Background Subtracted
220.0362
441.0816
m/z

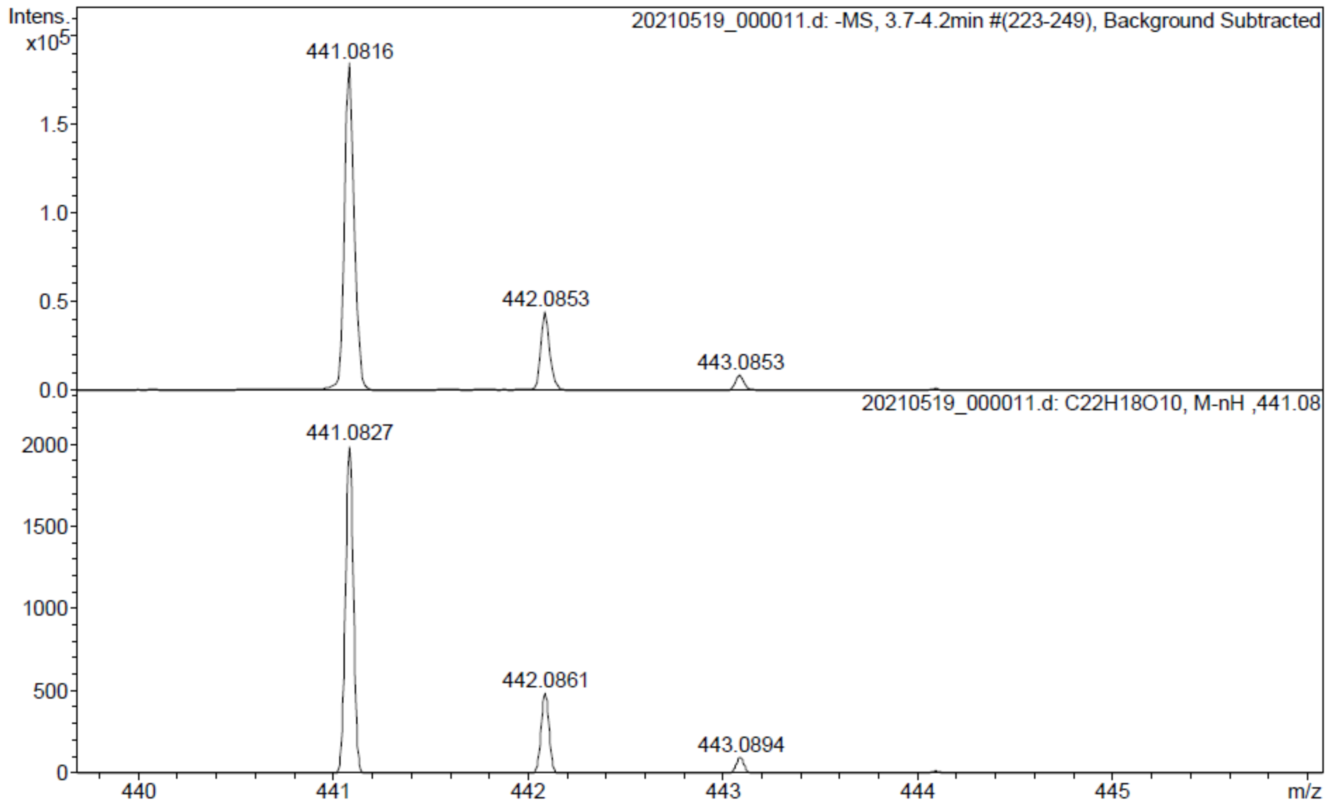
Intens.
x10^5
20210519_000011.d: -MS, 3.7-4.2min #(223-249), Background Subtracted
441.0816
442.0853
443.0853
20210519_000011.d: C22H18O10, M-nH ,441.08
441.0827
442.0861
443.0894
m/z

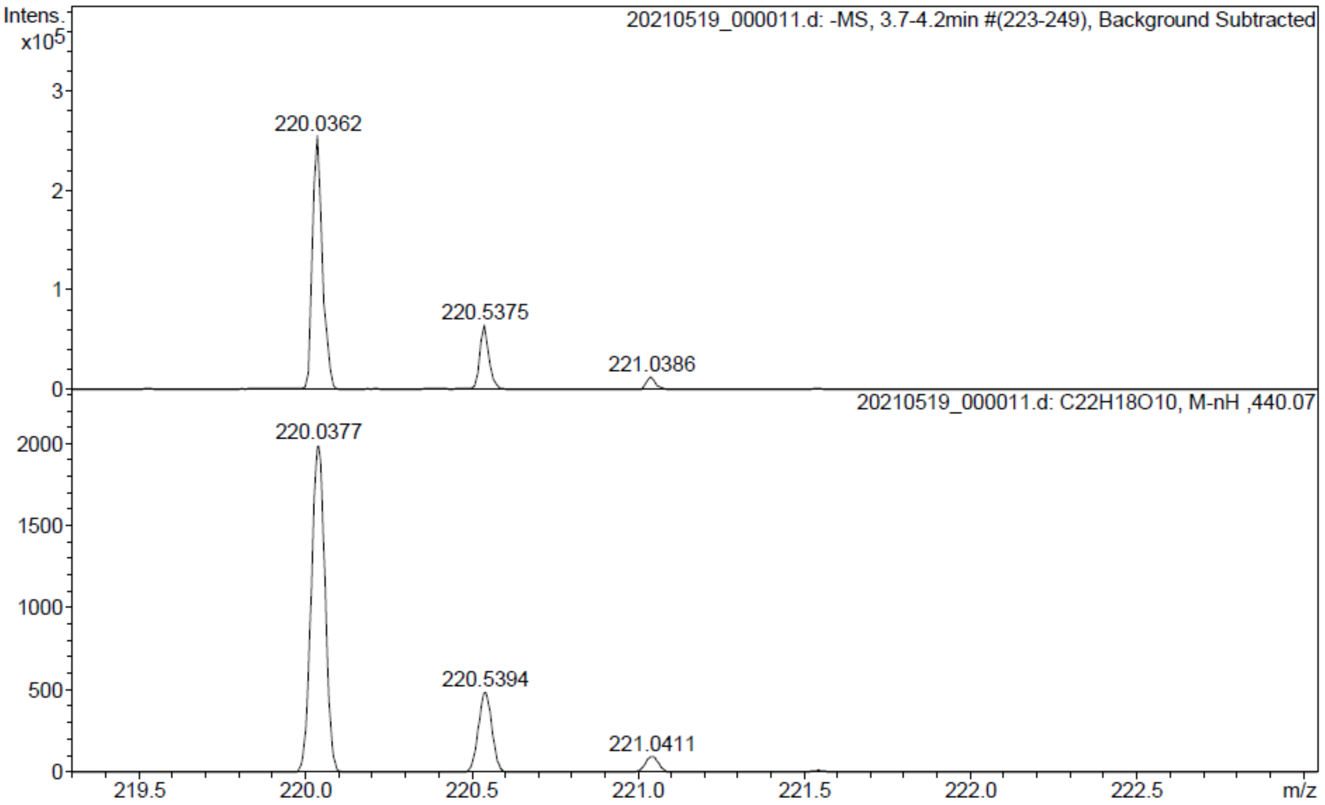

	Molecular formula	Calculated	Found
**[M–H]⁻**	$C_{22}H_{19}O_{10}$	441.0827	441.0816
**[M–2H]$^{2-}$**	$C_{22}H_{18}O_{10}$	220.0377	220.0362

2.10. Benzene-1,3,5-triyl tris(3,4,5-trihydroxybenzoate) (TL2)

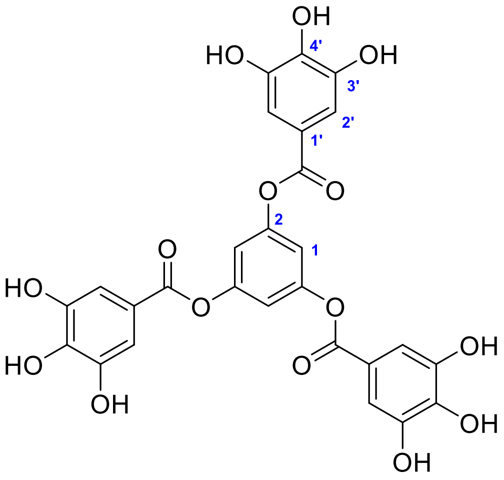

^{1}H-NMR (400 MHz, CD_3OD), δ (ppm)

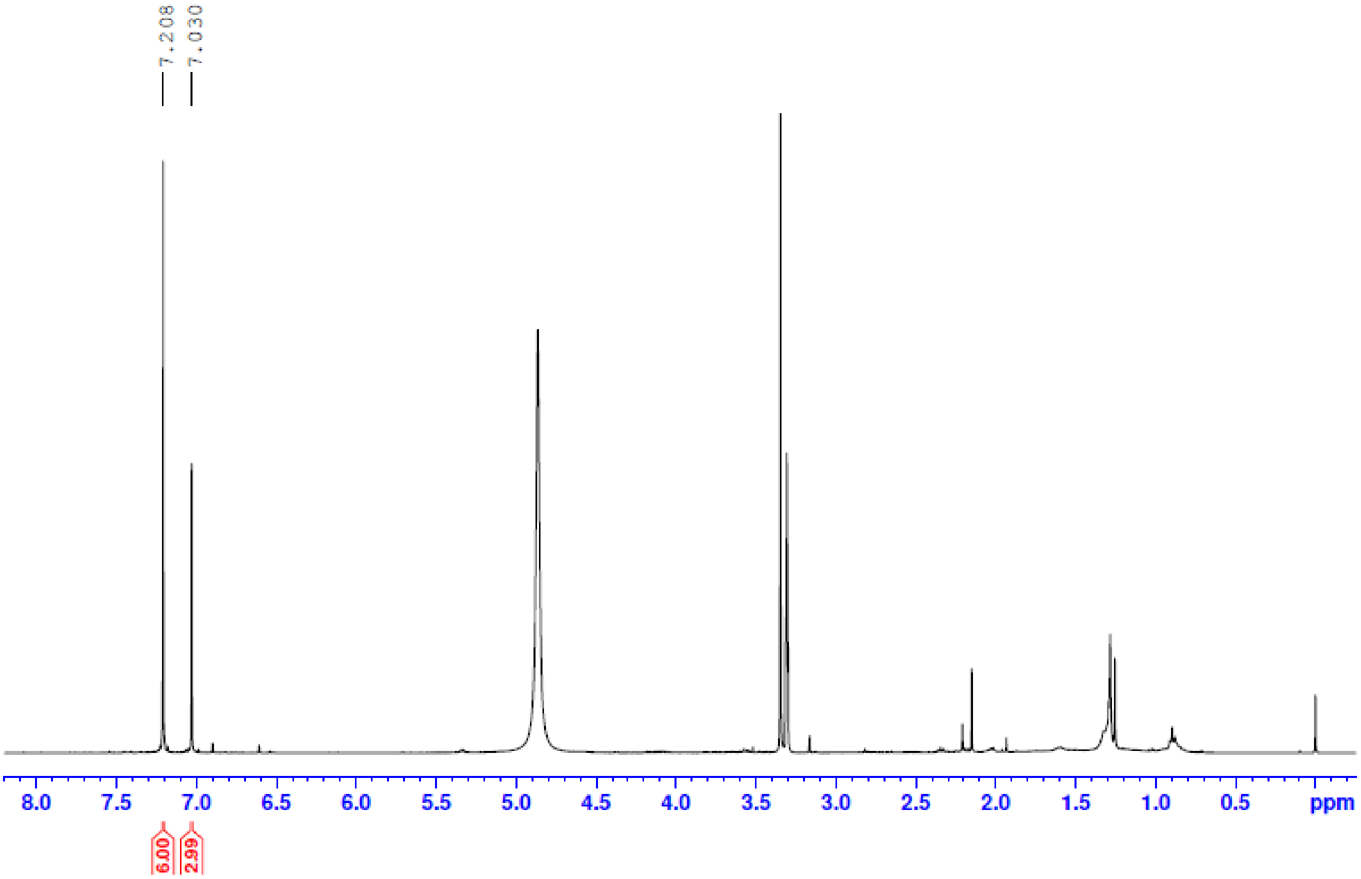

^{13}C-NMR (100 MHz, CD_3OD), δ (ppm)

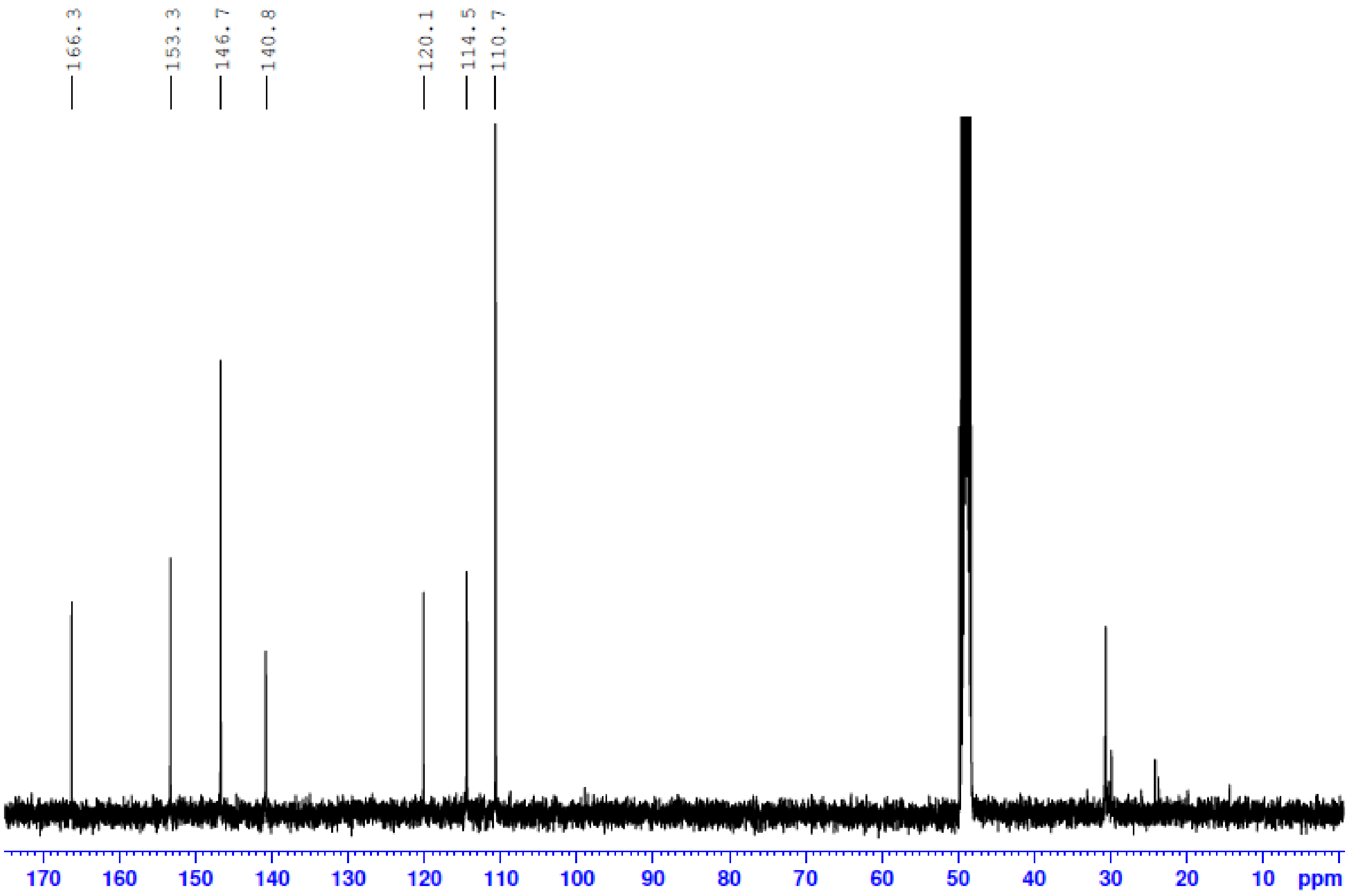

HSQC ^{1}H-^{13}C (CD_3OD), δ (ppm)

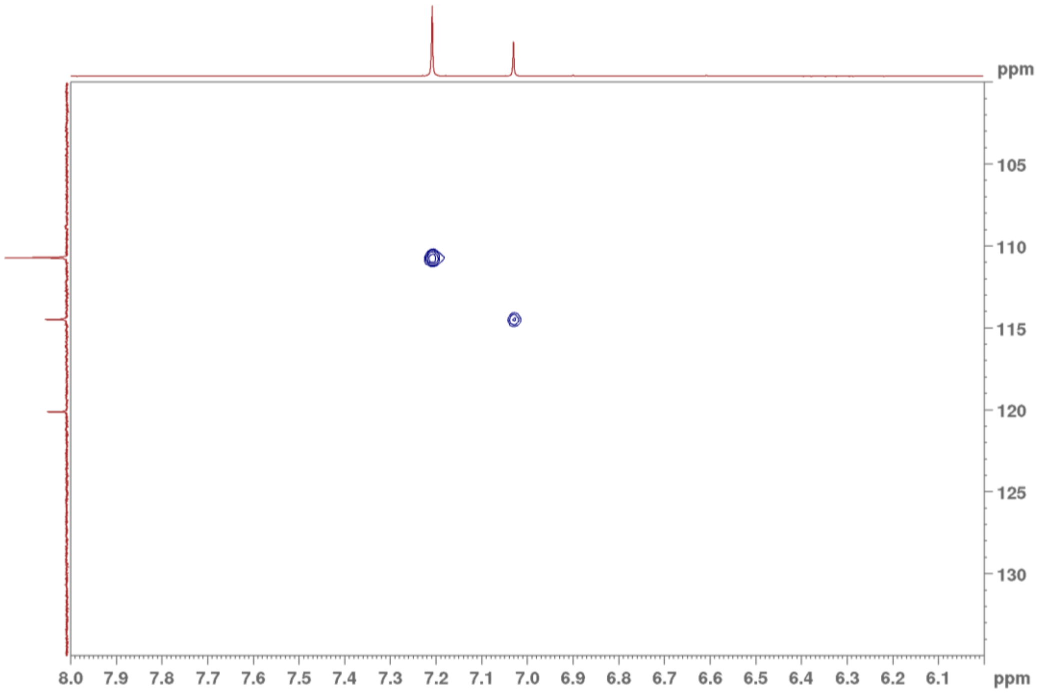

HMBC ^{1}H-^{13}C (CD_3OD), δ (ppm)

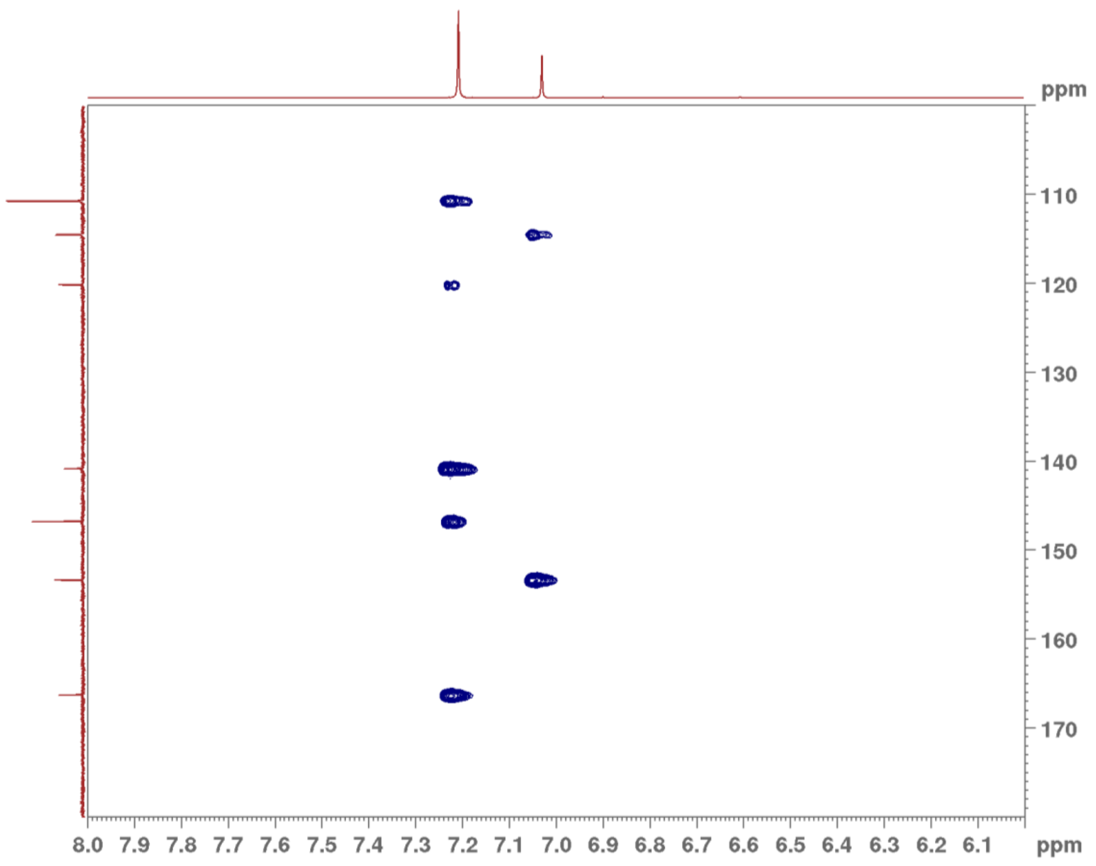

FT-IR (neat), ν (cm^{-1})

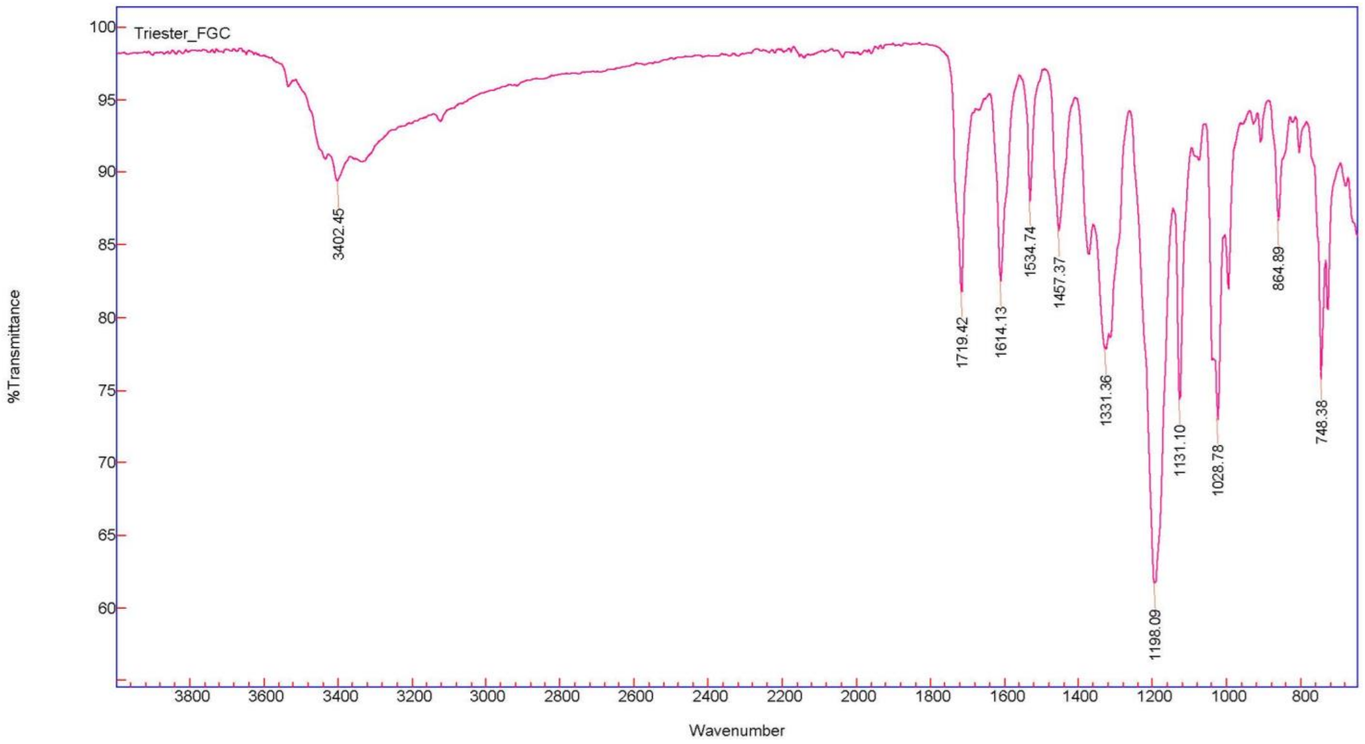

HPLC (λ = 220 nm)

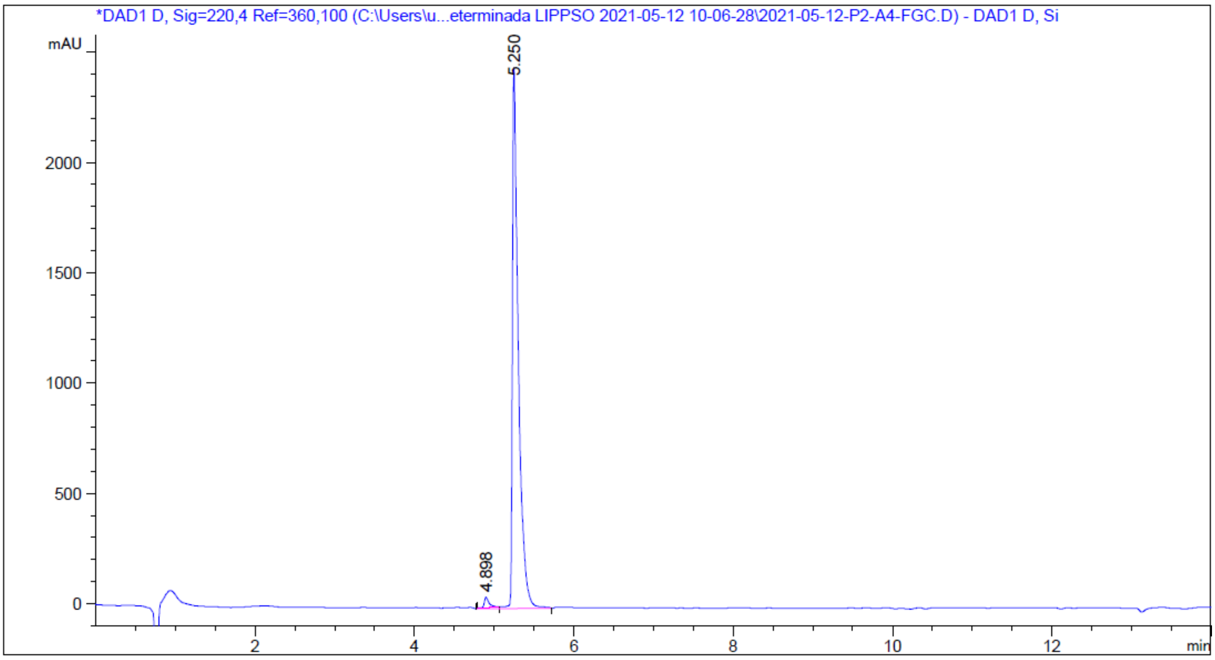

Peak #	RetTime [min]	Type	Width [min]	Area [mAU*s]	Height [mAU]	Area %
1	4.898	BV E	0.0589	195.00165	47.47273	1.4974
2	5.250	VB R	0.0721	1.28280e4	2451.05640	98.5026

Totals : 1.30230e4 2498.52913

ESI-MS (*m/z*)

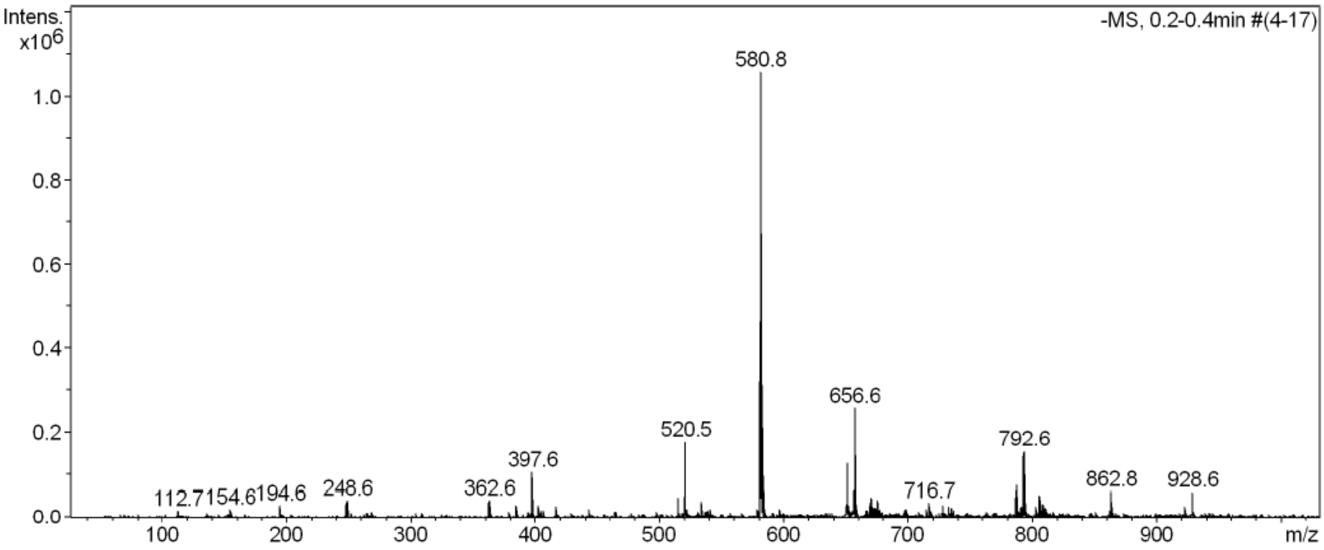

HRMS (*m/z*)

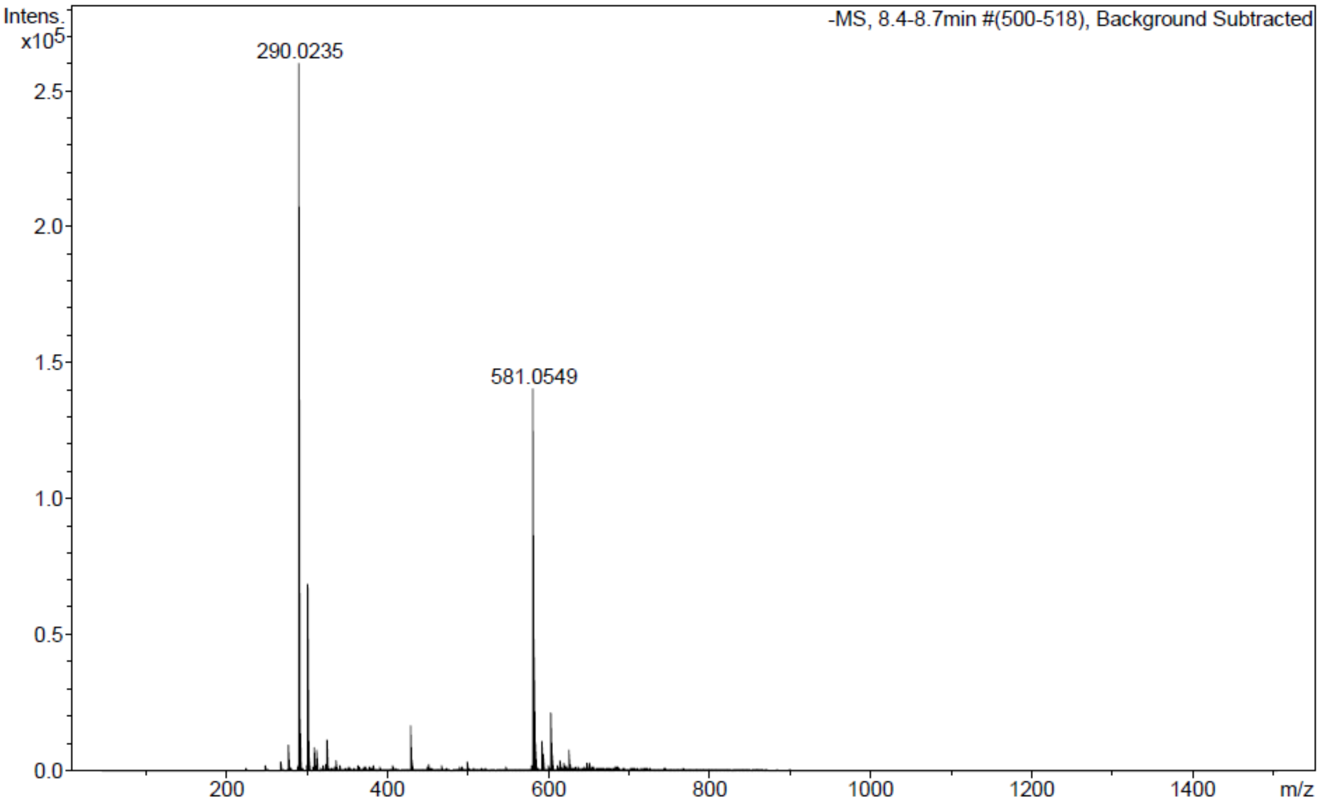

Intens.
x10^5
-MS, 8.4-8.7min #(500-518), Background Subtracted
290.0235
581.0549
2.5
2.0
1.5
1.0
0.5
0.0
200
400
600
800
1000
1200
1400
m/z

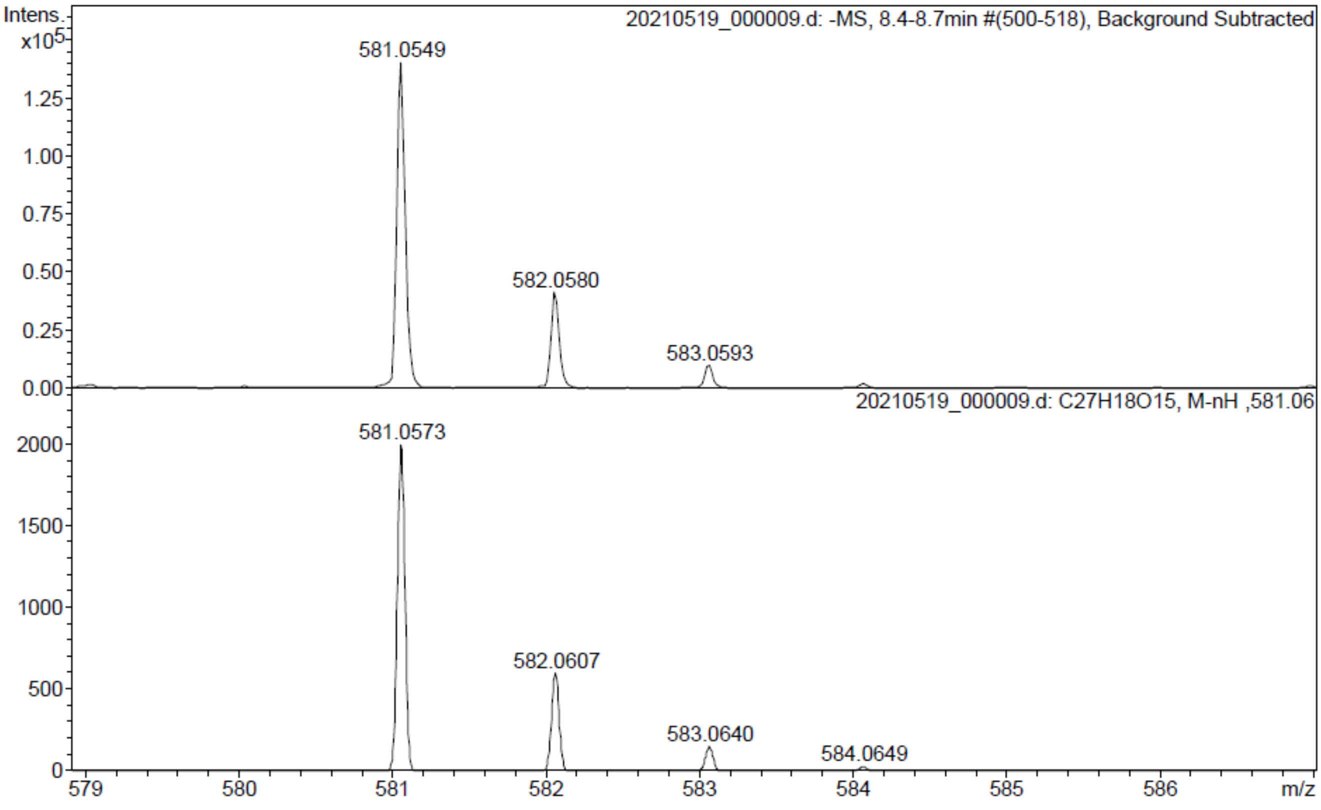

Intens.
x10^5
20210519_000009.d: -MS, 8.4-8.7min #(500-518), Background Subtracted
581.0549
582.0580
583.0593
1.25
1.00
0.75
0.50
0.25
0.00
20210519_000009.d: C27H18O15, M-nH ,581.06
581.0573
582.0607
583.0640
584.0649
2000
1500
1000
500
0
579
580
581
582
583
584
585
586
m/z

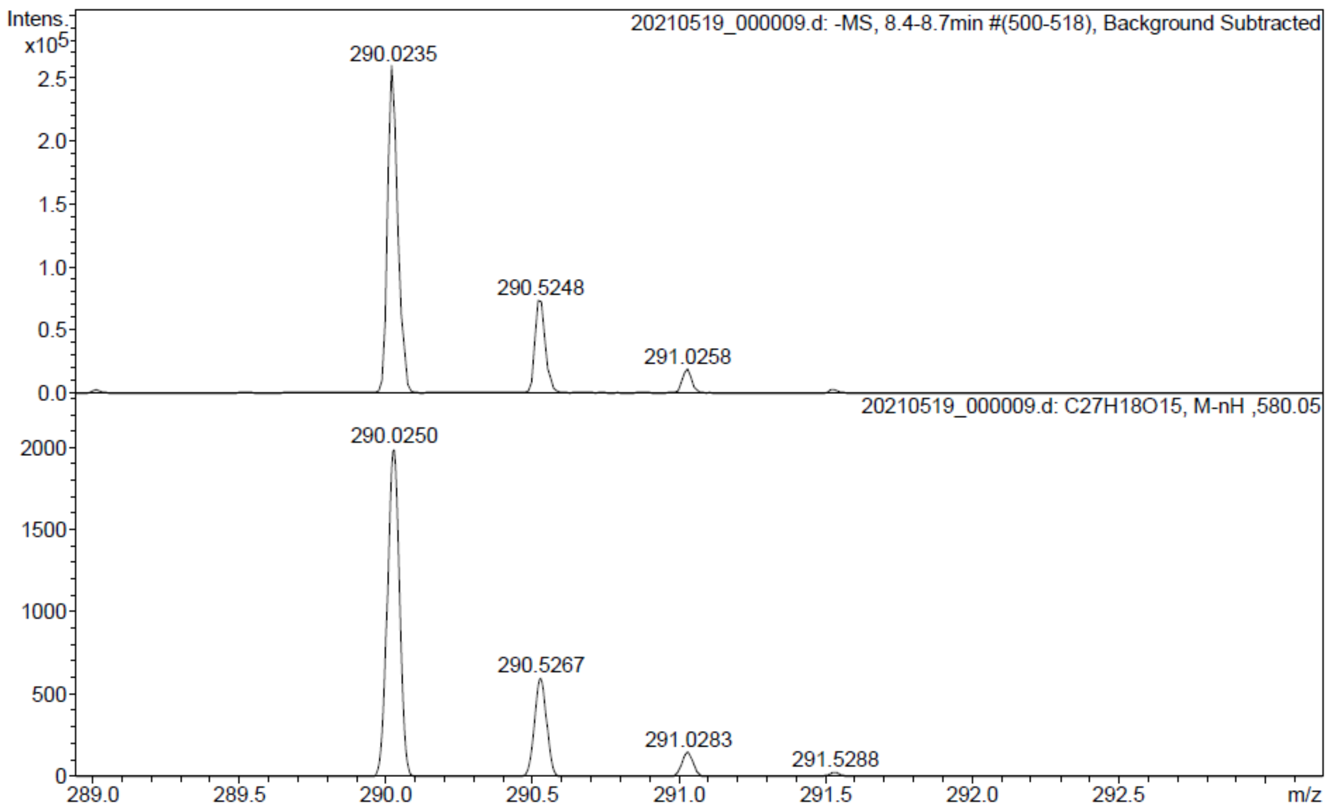

	Molecular formula	Calculated	Found
**[M–H]⁻**	$C_{27}H_{17}O_{15}$	518.0573	581.0549
**[M–2H]$^{2–}$**	$C_{27}H_{16}O_{10}$	290.0250	290.0239

2.11. 1,4-Phenylenebis(methylene) bis(3,4,5-trihydroxybenzoate) (TL3)

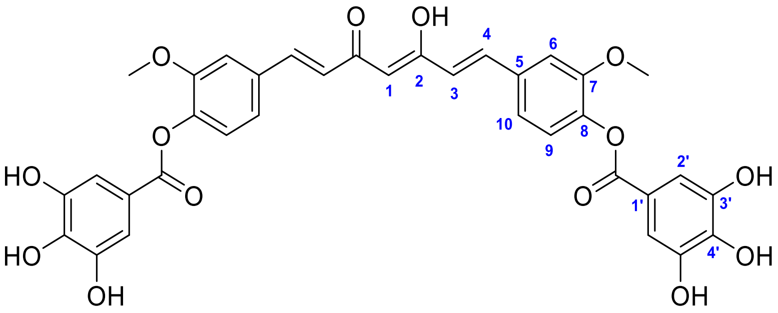

^{1}H-NMR (400 MHz, $(CD_3)_2CO$), δ (ppm)

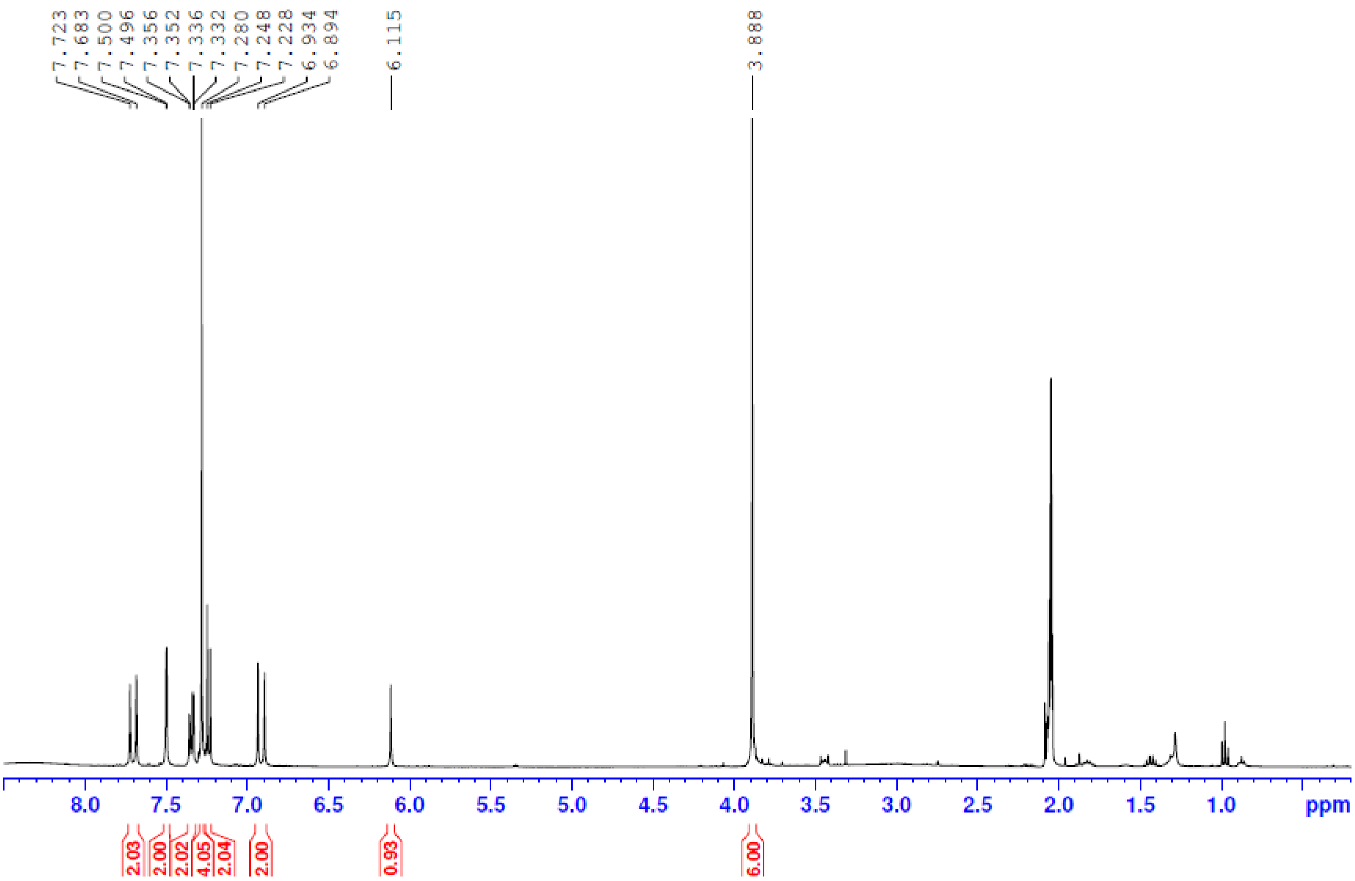

^{1}H-NMR (400 MHz, $(CD_3)_2CO$), δ (ppm) – Aromatic zoom

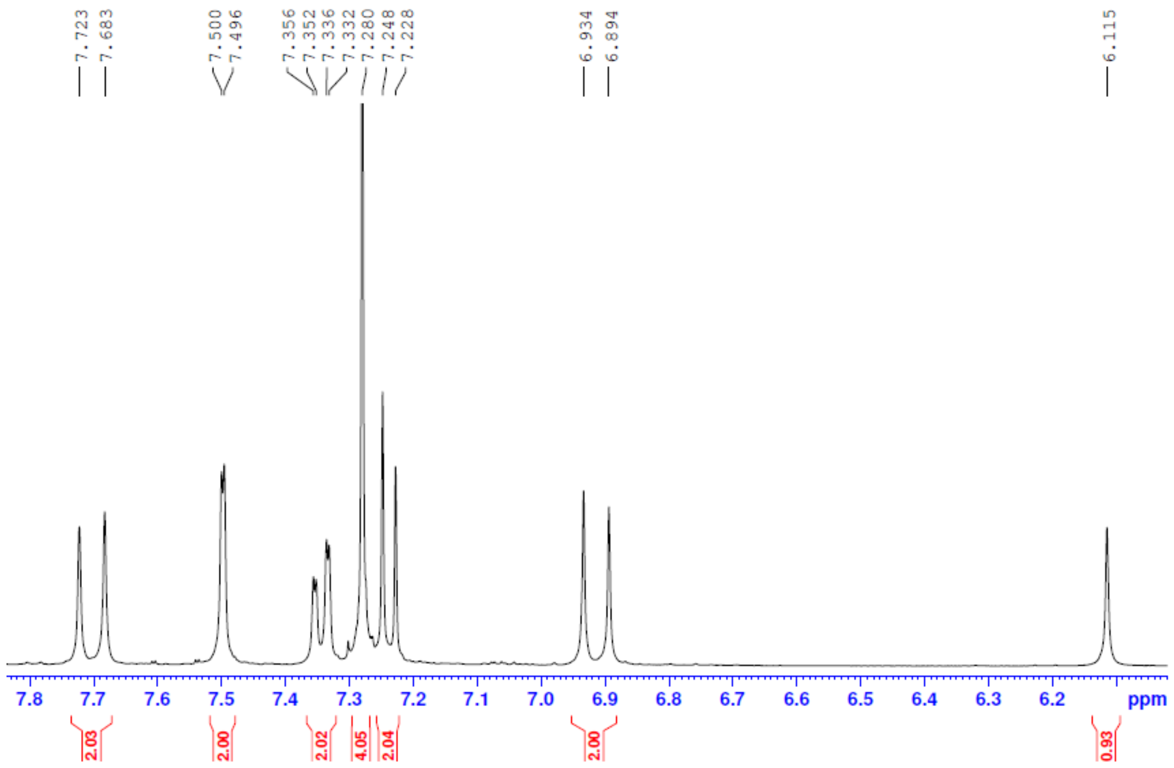

^{13}C-NMR (100 MHz, $(CD_3)_2CO$), δ (ppm)

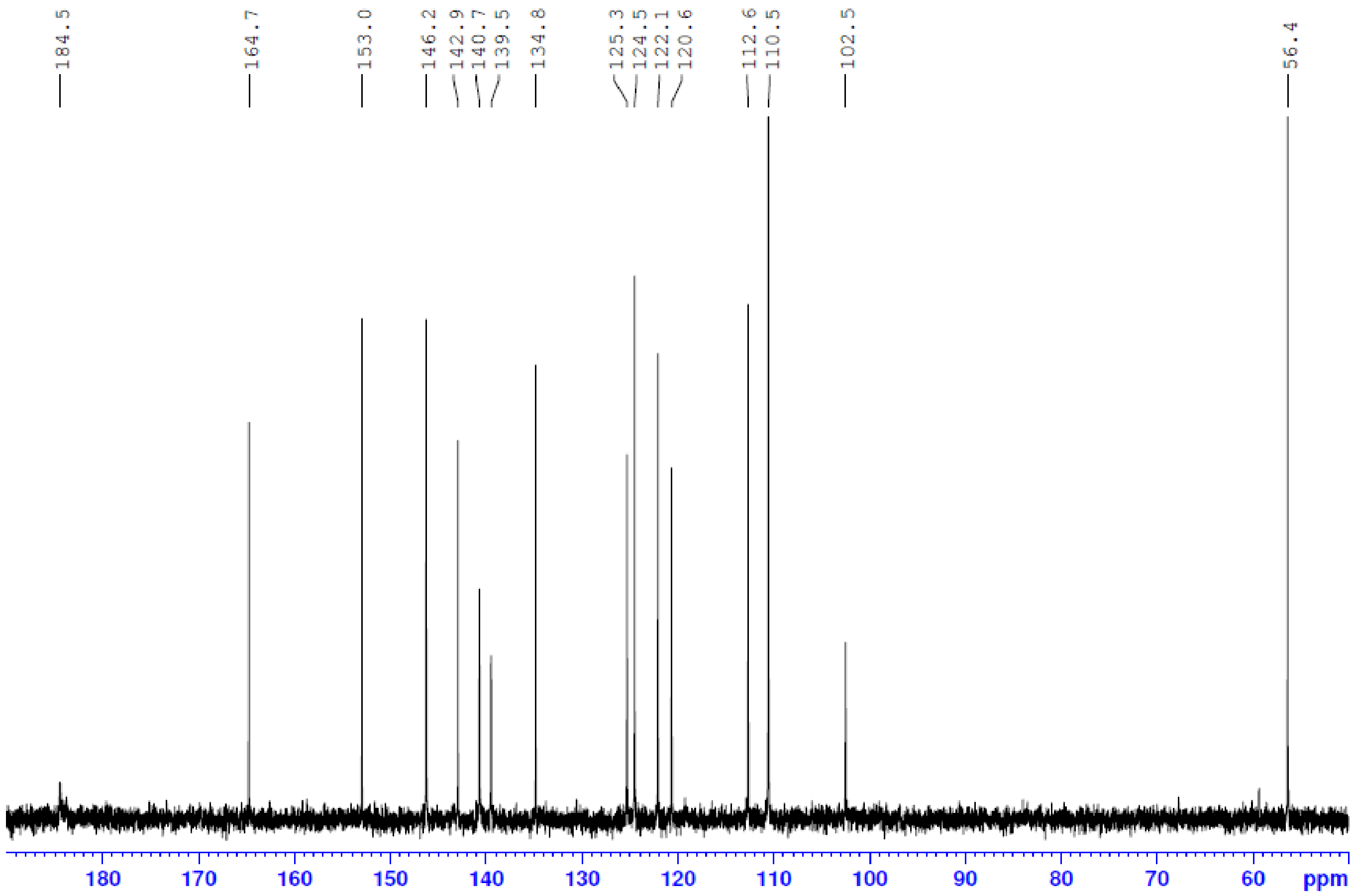

COSY ^1H-^1H ($(CD_3)_2CO$), δ (ppm)

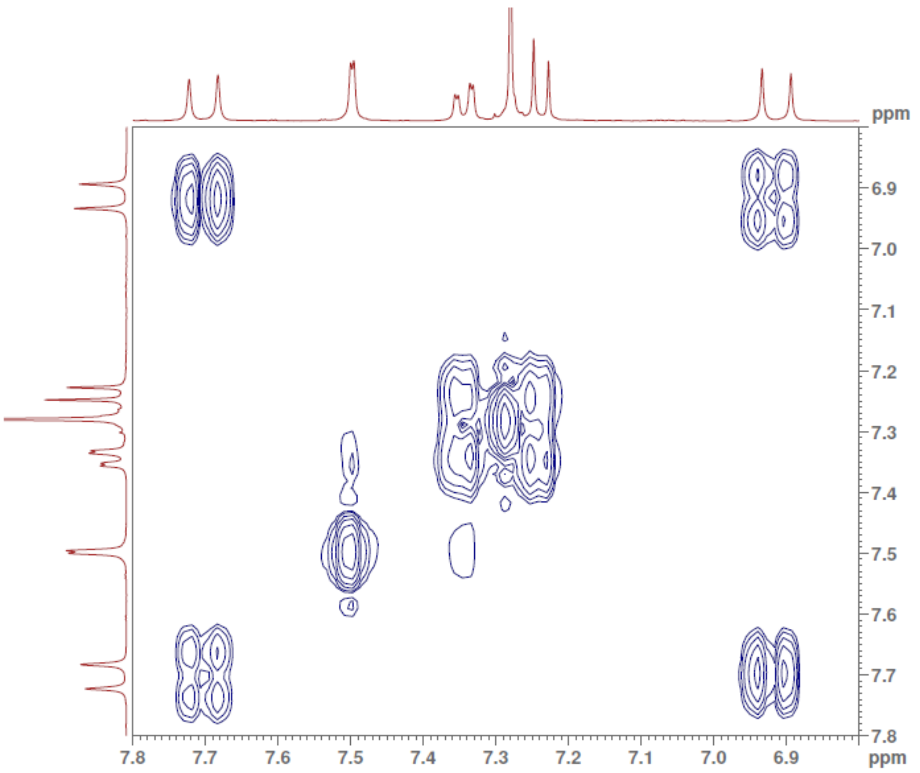

HSQC ^1H-^{13}C ($(CD_3)_2CO$), δ (ppm)

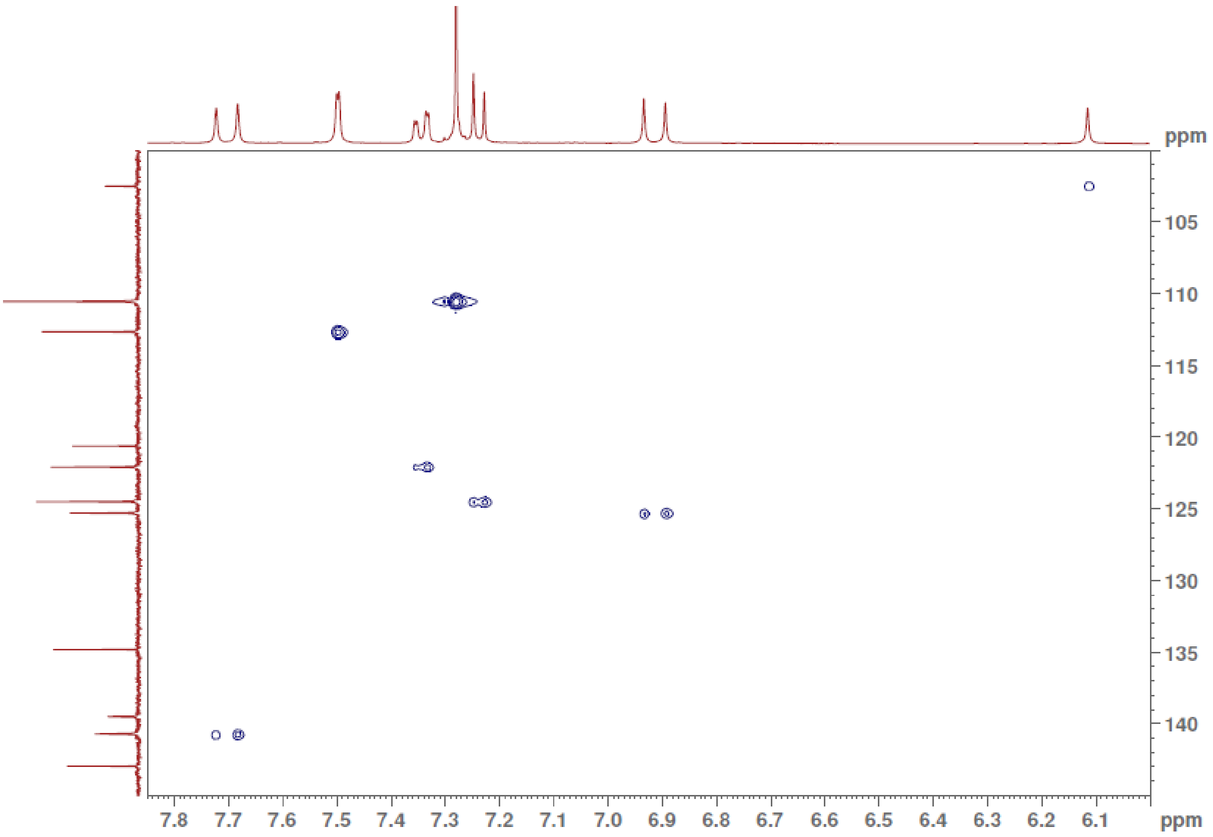

HMBC ^{1}H-^{13}C ($(CD_3)_2CO$), δ (ppm)

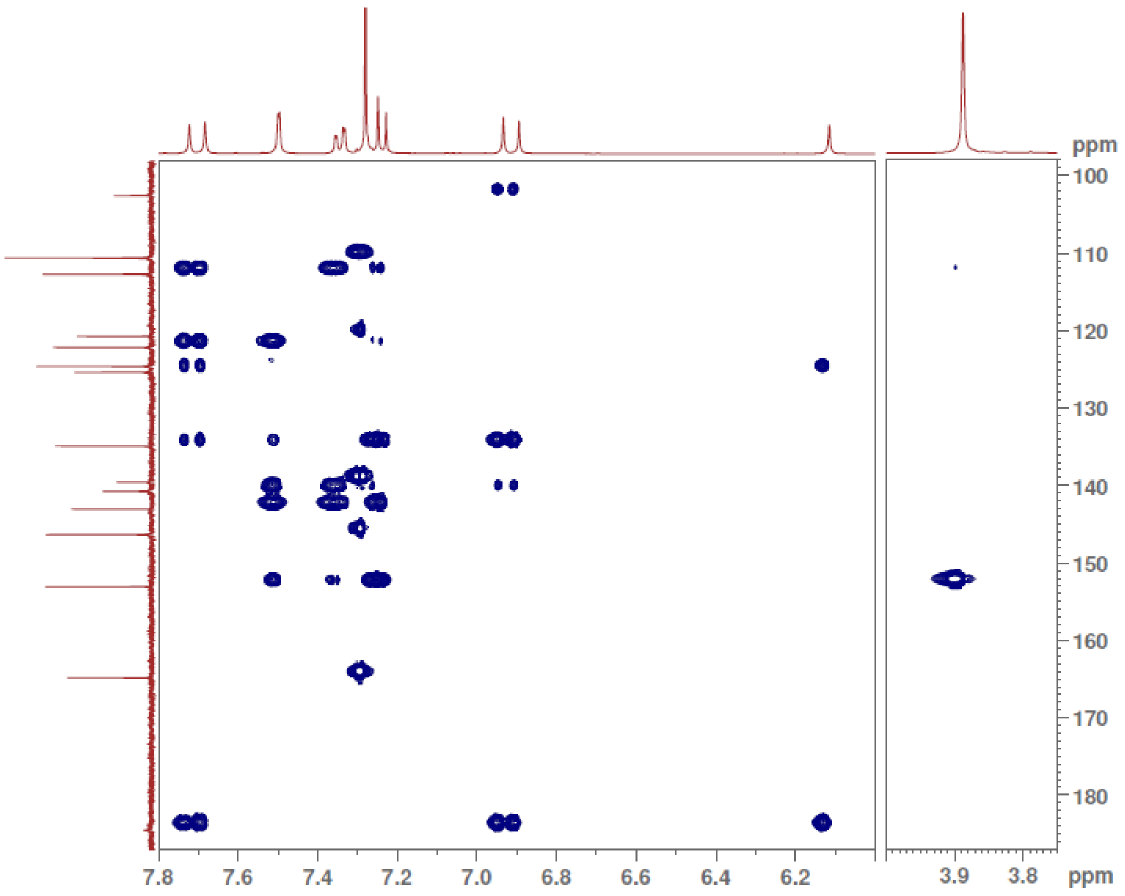

NOESY ^{1}H-^{1}H ($(CD_3)_2CO$), δ (ppm)

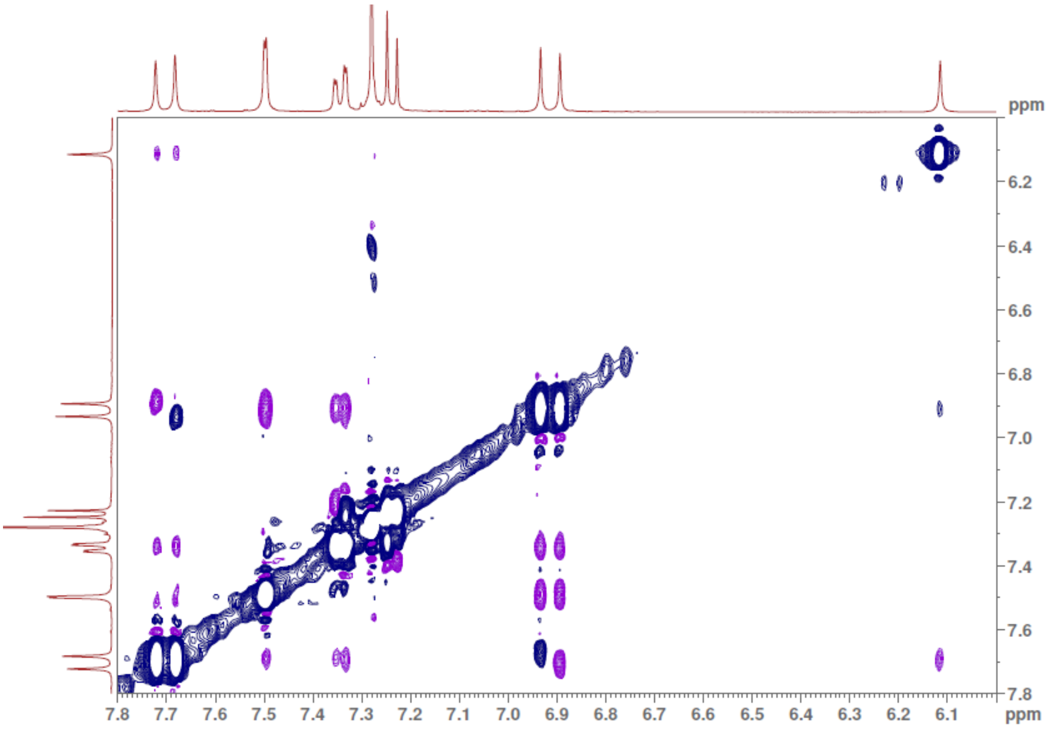

FT-IR (neat), ν (cm^{-1})

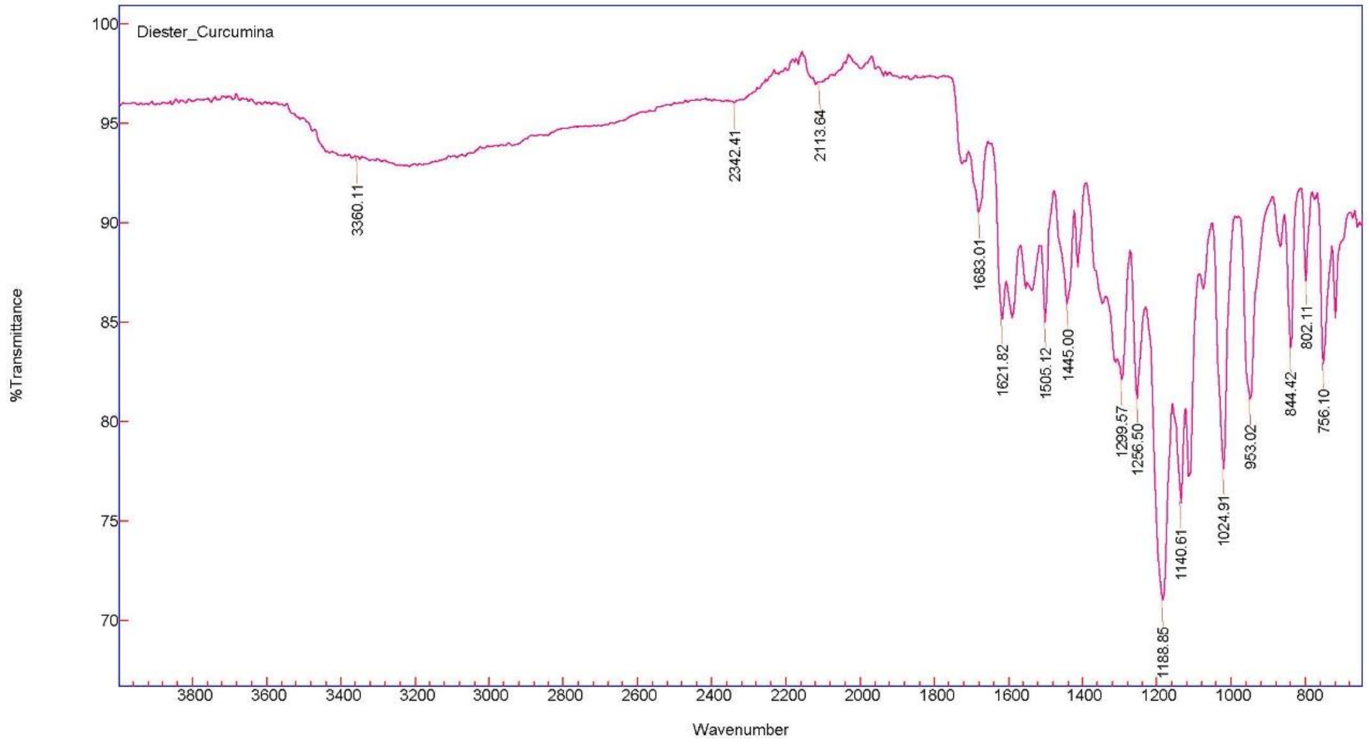

HPLC (λ = 220 nm)

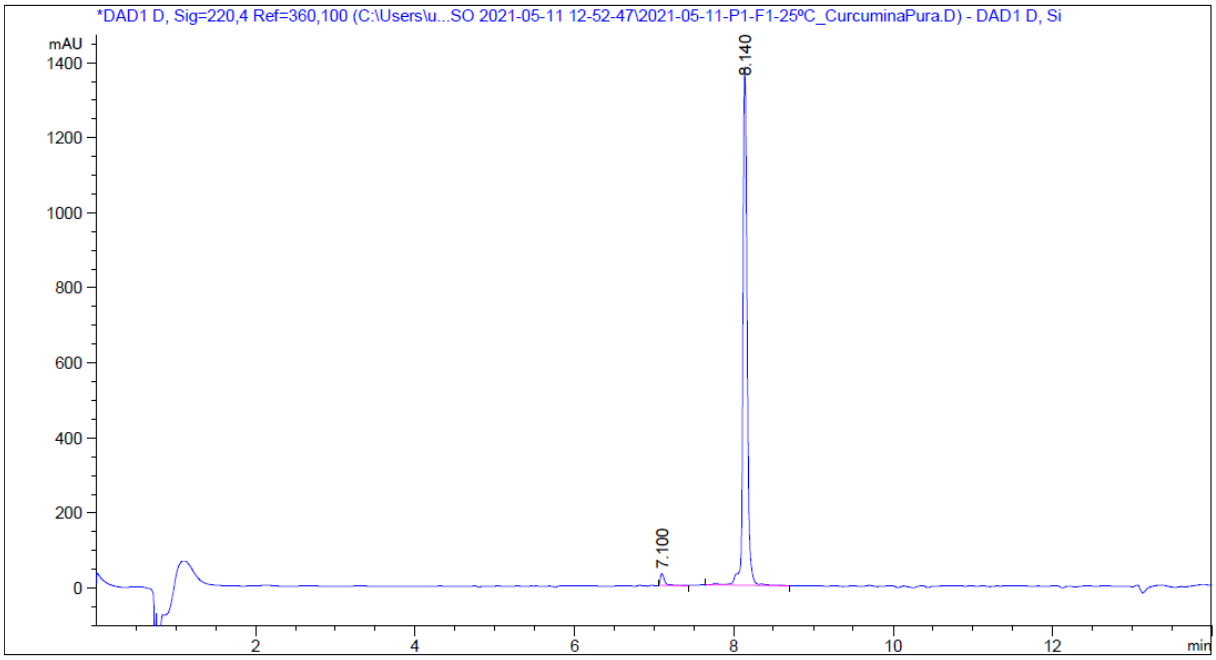

Peak #	RetTime [min]	Type	Width [min]	Area [mAU*s]	Height [mAU]	Area %
1	7.100	BV R	0.0556	121.17000	32.40459	2.3111
2	8.140	VV R	0.0572	5121.74561	1382.56921	97.6889

Totals : 5242.91560 1414.97380

(–)-ESI-MS (*m/z*)

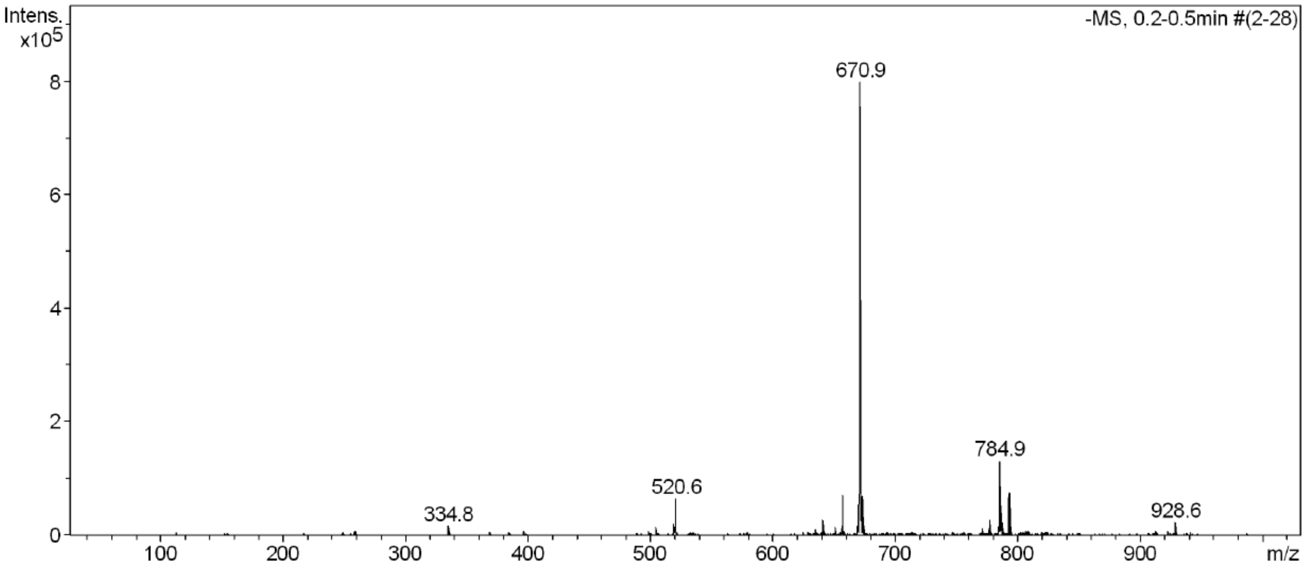

(–)-ESI-MS^2 (*m/z* = 670.9)

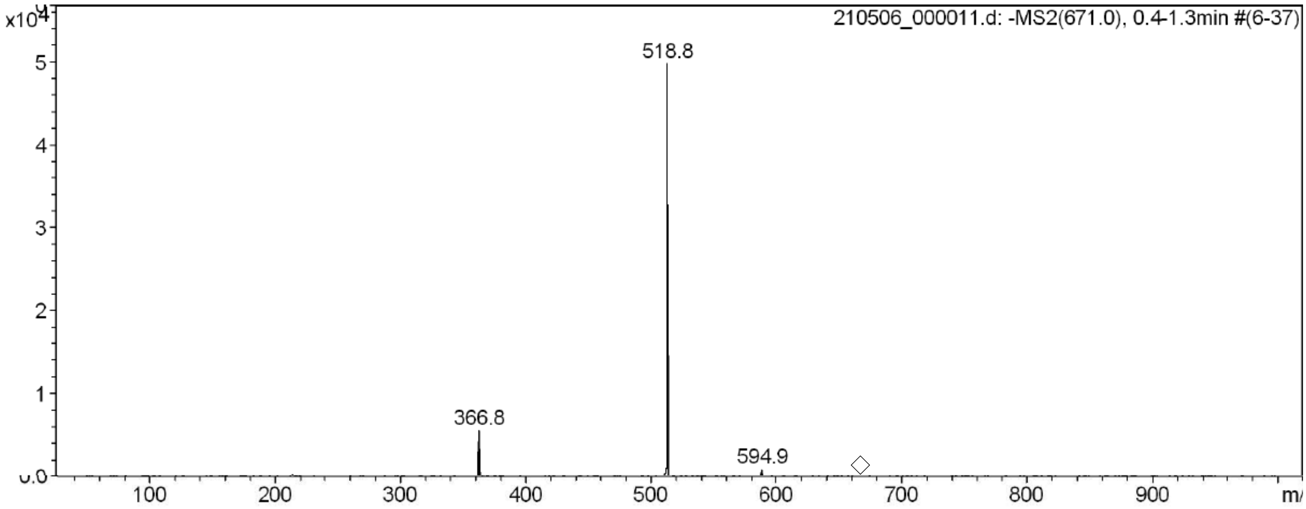

(–)-ESI-MS^3 (*m/z* = 518.8)

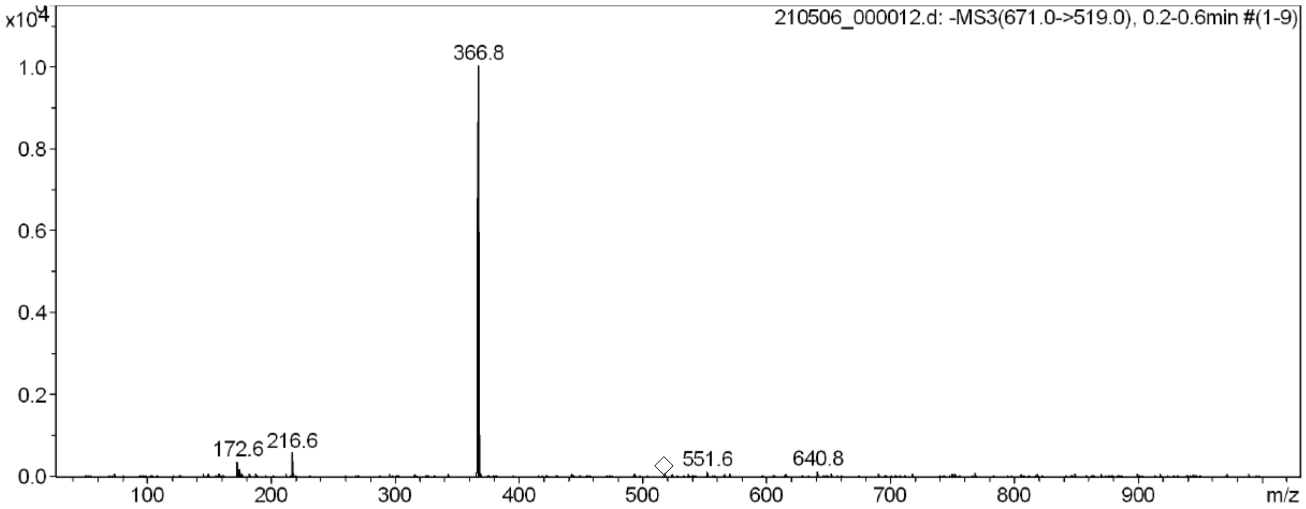

HRMS (*m/z*)

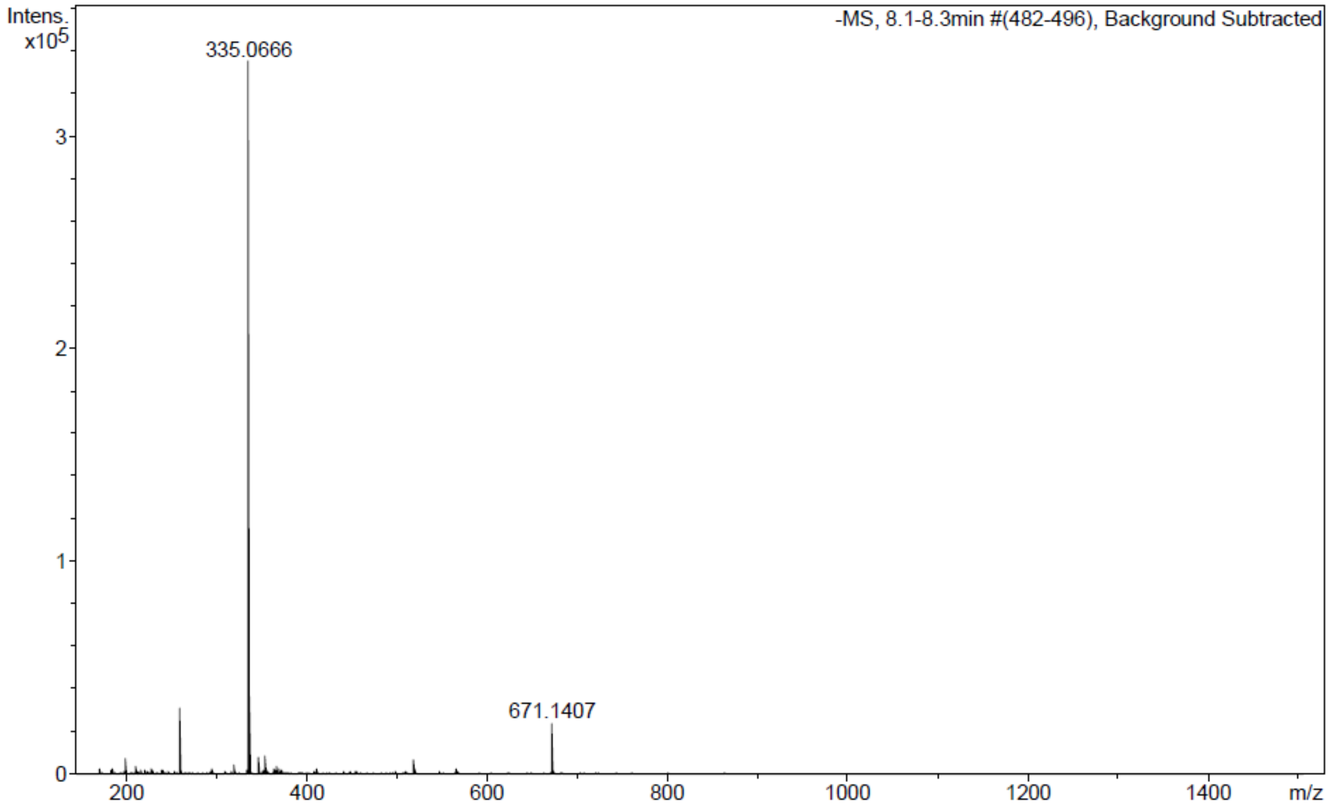
Intens.
x10^5
-MS, 8.1-8.3min #(482-496), Background Subtracted
335.0666
671.1407
3
2
1
0
200
400
600
800
1000
1200
1400
m/z

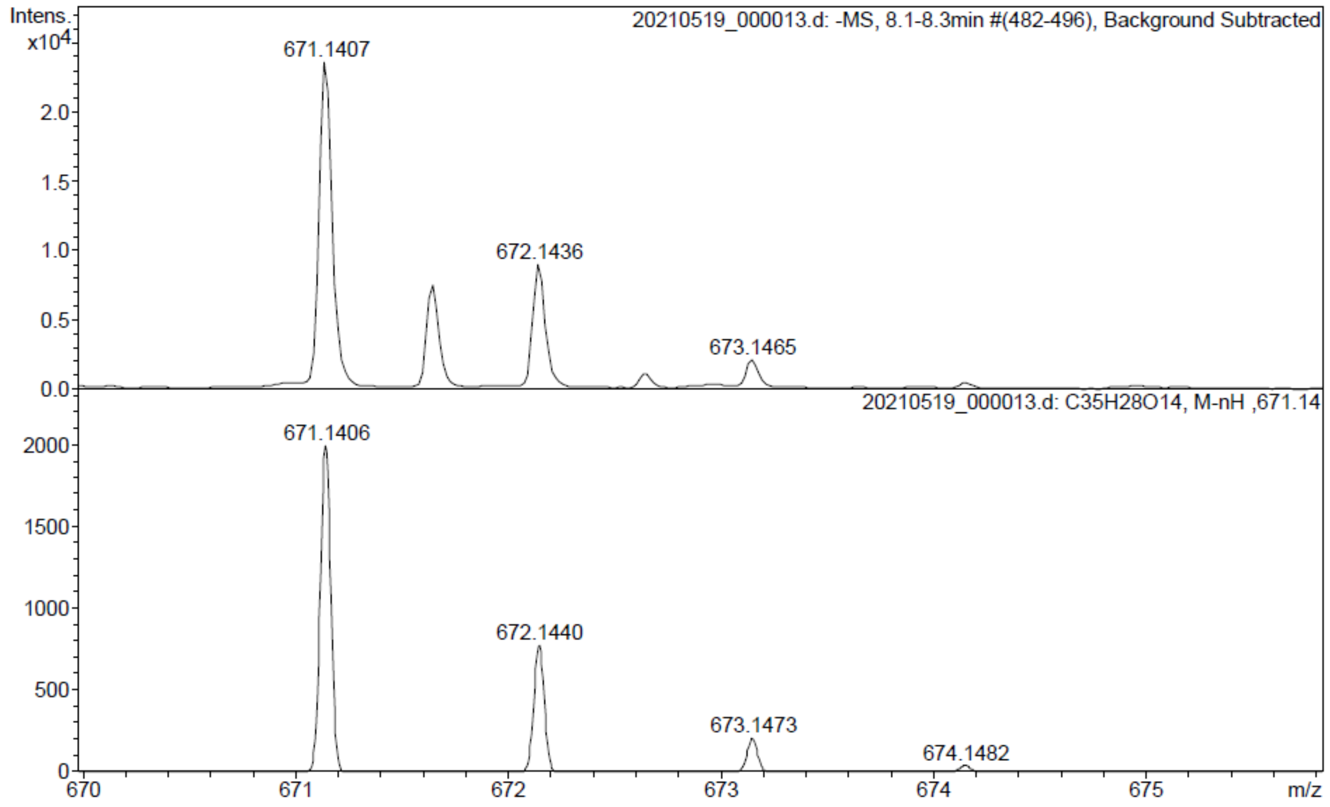
Intens.
x10^4
20210519_000013.d: -MS, 8.1-8.3min #(482-496), Background Subtracted
671.1407
672.1436
673.1465
2.0
1.5
1.0
0.5
0.0
20210519_000013.d: C35H28O14, M-nH ,671.14
671.1406
672.1440
673.1473
674.1482
2000
1500
1000
500
0
670
671
672
673
674
675
m/z

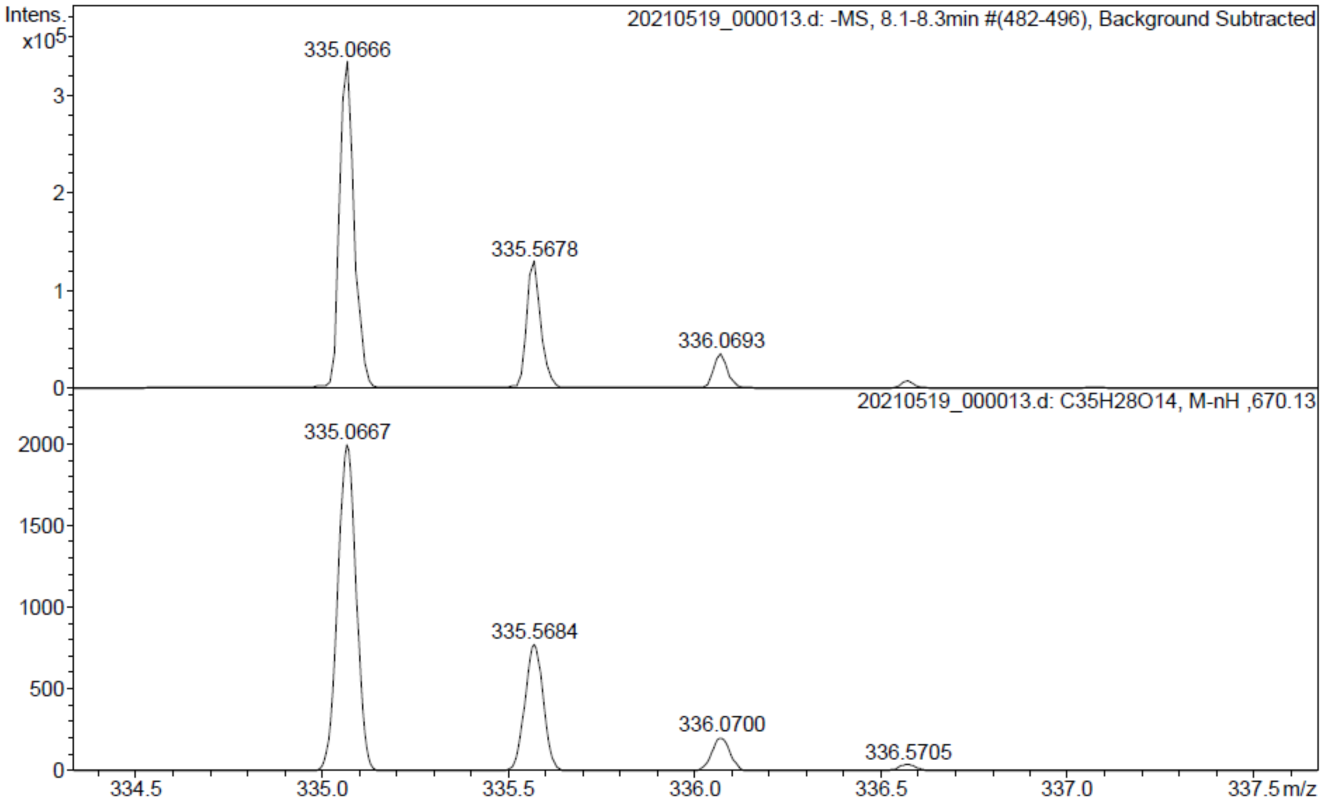

	Molecular formula	Calculated	Found
**[M–H]⁻**	$C_{35}H_{27}O_{14}$	671.1406	671.1407
**[M–2H]$^{2-}$**	$C_{35}H_{26}O_{14}$	335.0667	335.0666

2.12. ((1*E*,1'*E*)-(2-Oxocyclohexane-1,3-diylidene)bis(methanylylidene))bis(2-methoxy-4,1-phenylene) bis[3,4,5-tris((*tert*-butyldimethylsilyl)oxy)benzoate] (TL4)

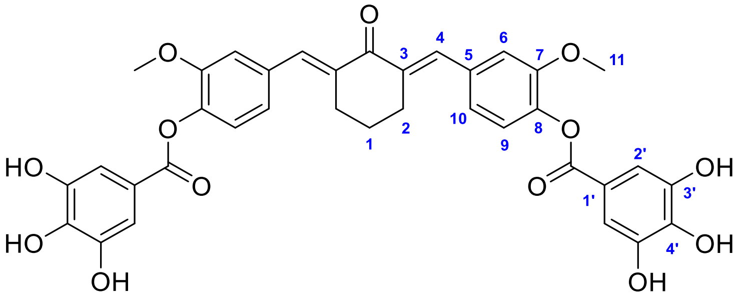

^{1}H-NMR (400 MHz, $(CD_3)_2CO$), δ (ppm)

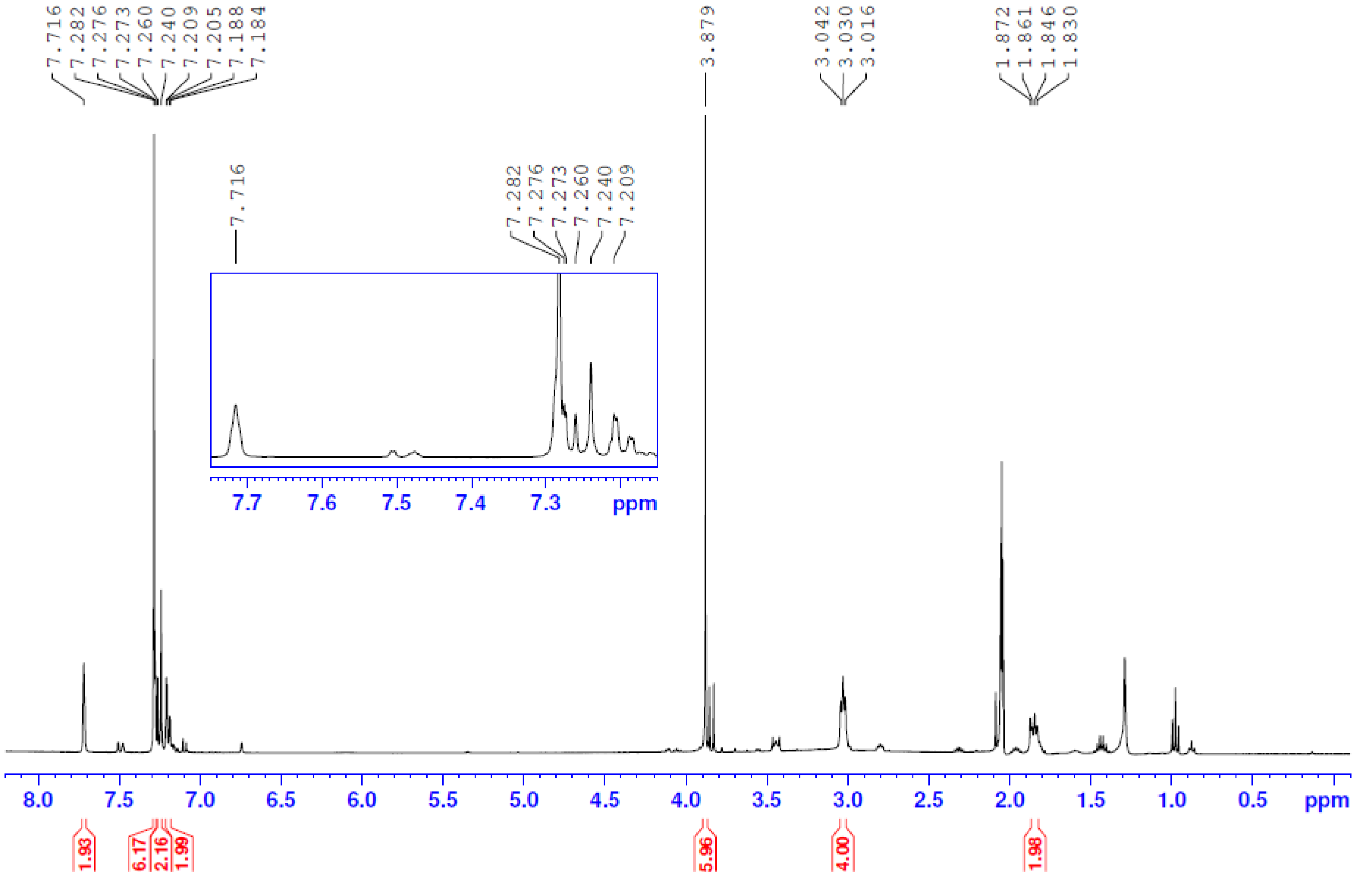

^{13}C-NMR (100 MHz, $(CD_3)_2CO$), δ (ppm)

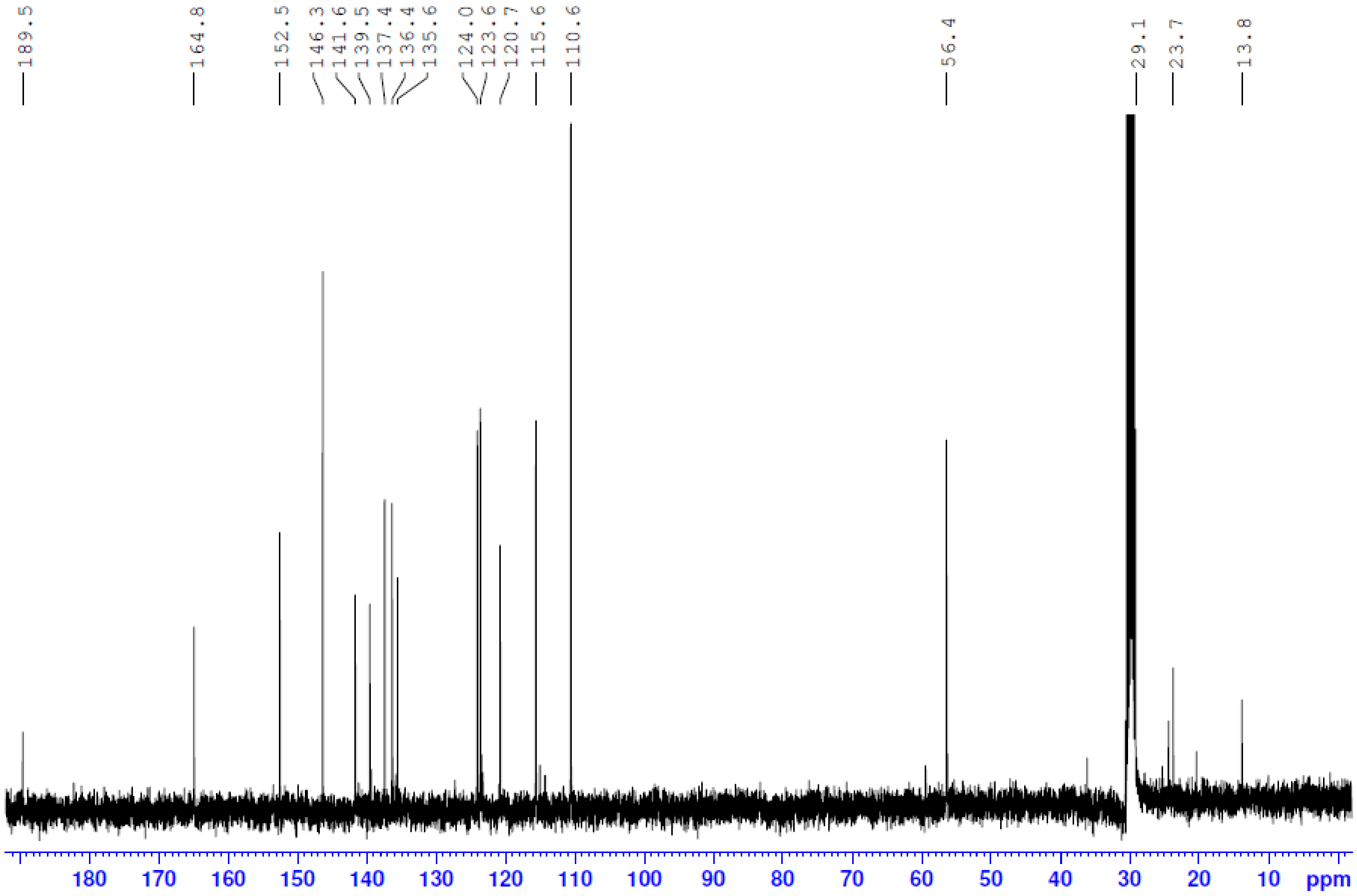

COSY ^{1}H-^{1}H ($(CD_3)_2CO$), δ (ppm)

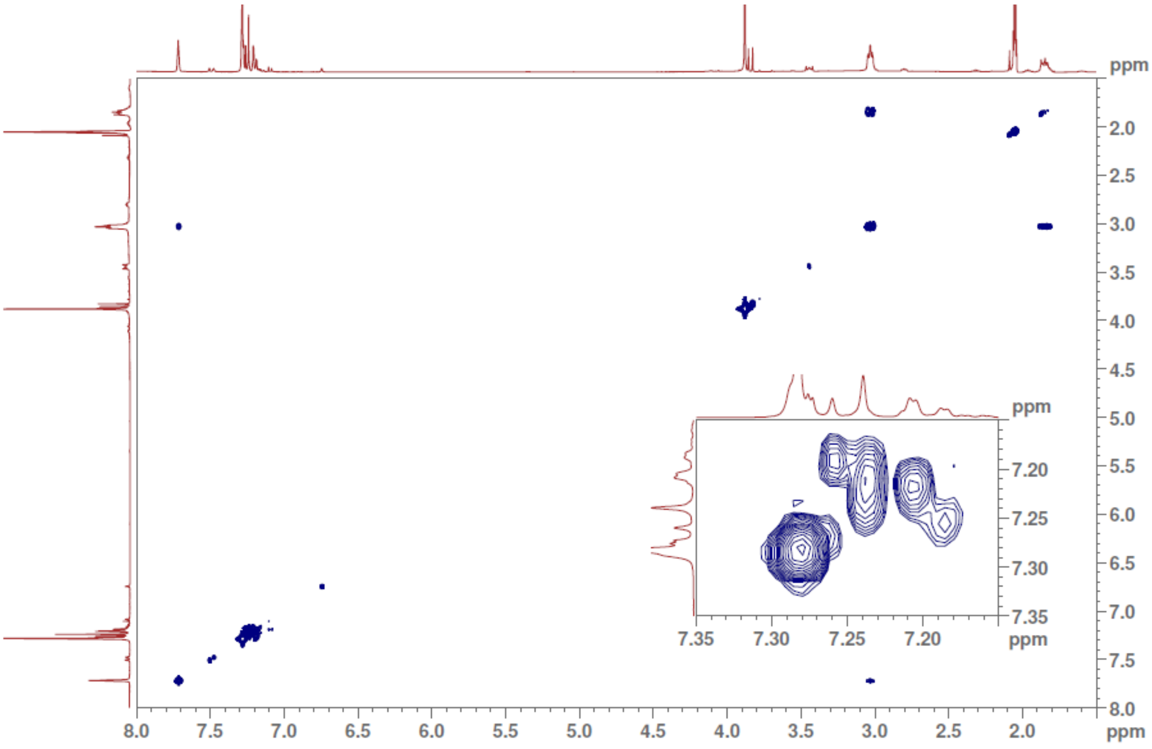

HSQC ^{1}H-^{13}C ($(CD_3)_2CO$), δ (ppm)

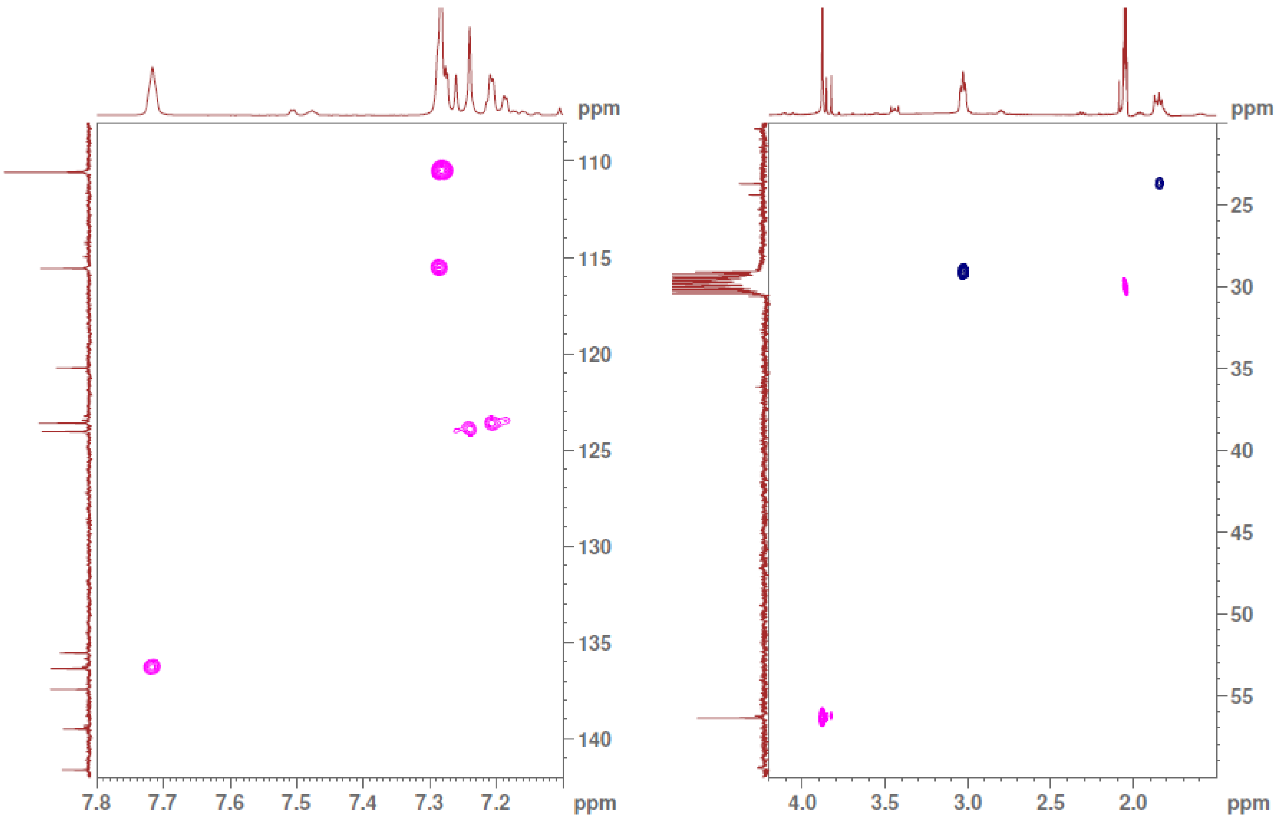

HMBC ^{1}H-^{13}C ($(CD_3)_2CO$), δ (ppm)

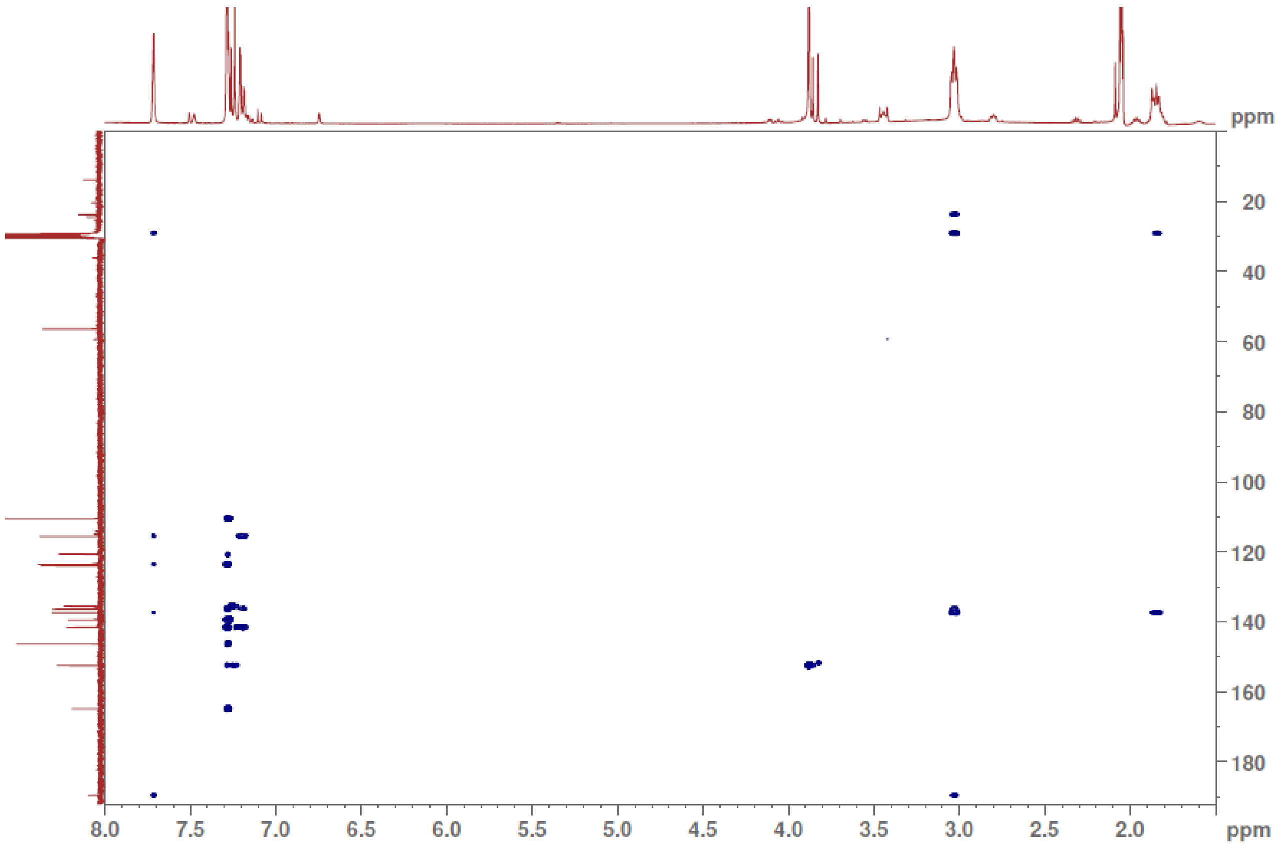

FT-IR (neat), ν (cm^{-1})

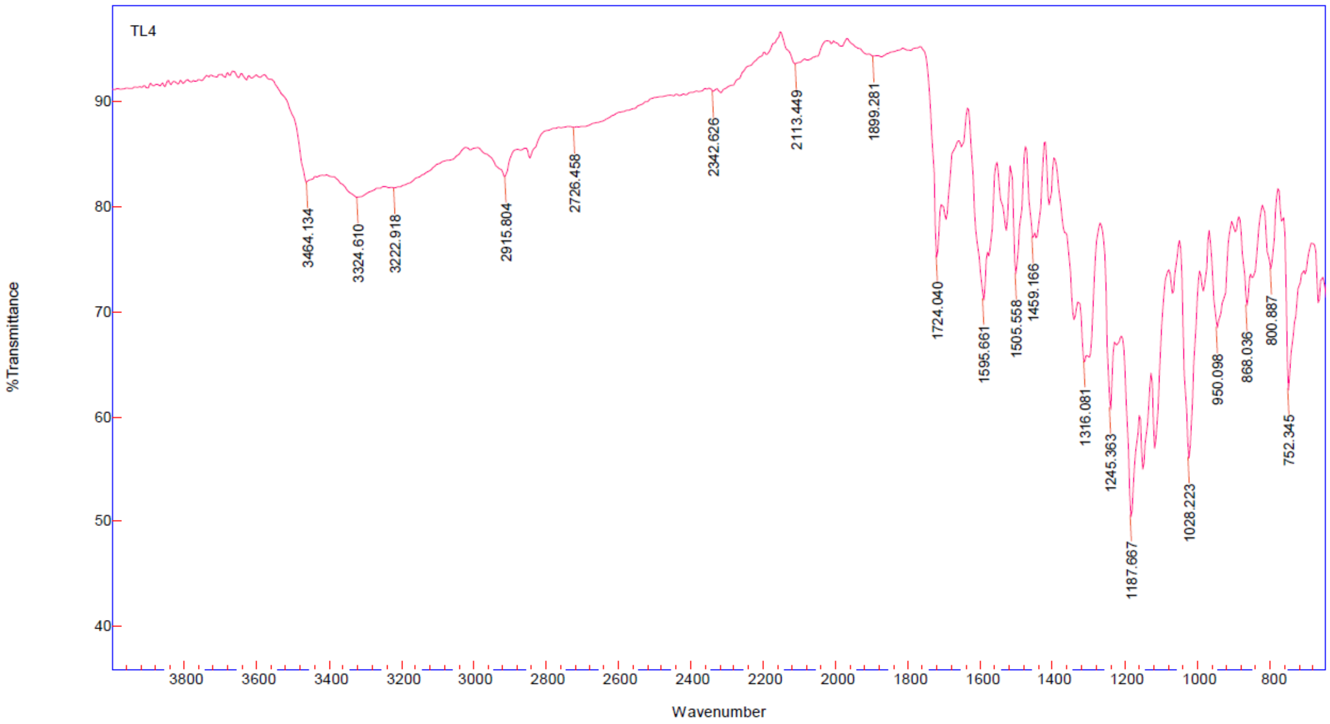

UV-Vis (CH_3CN), λ (nm)

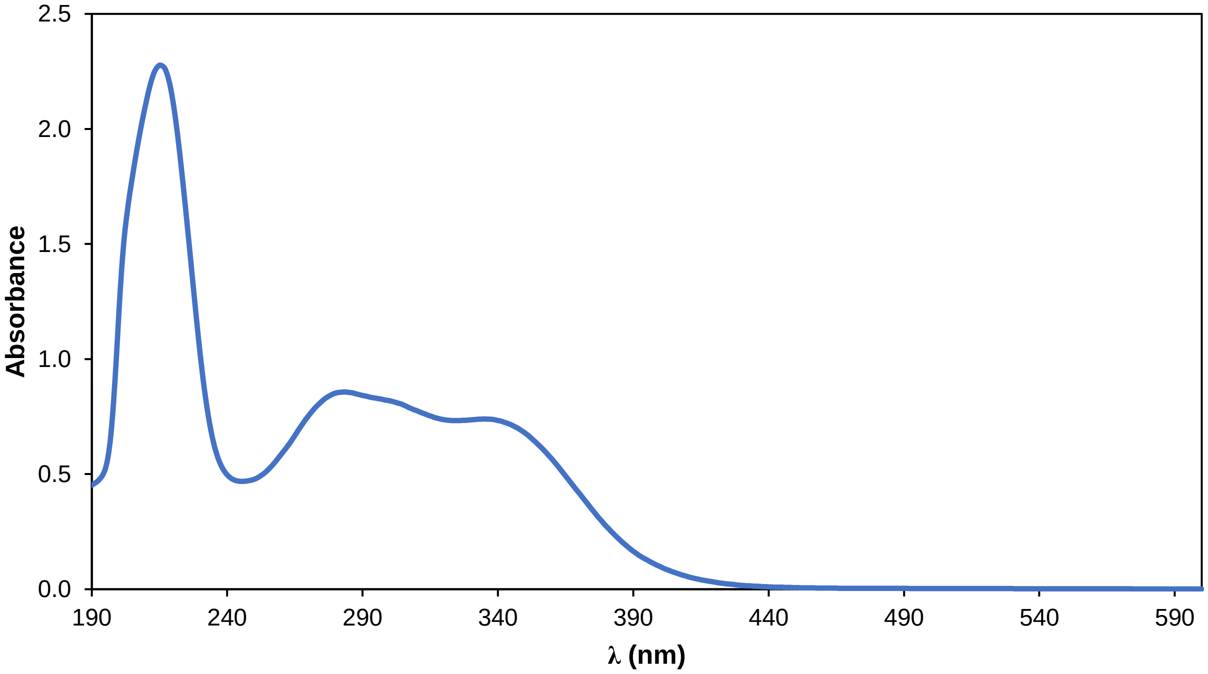

HPLC (λ = 220 nm)

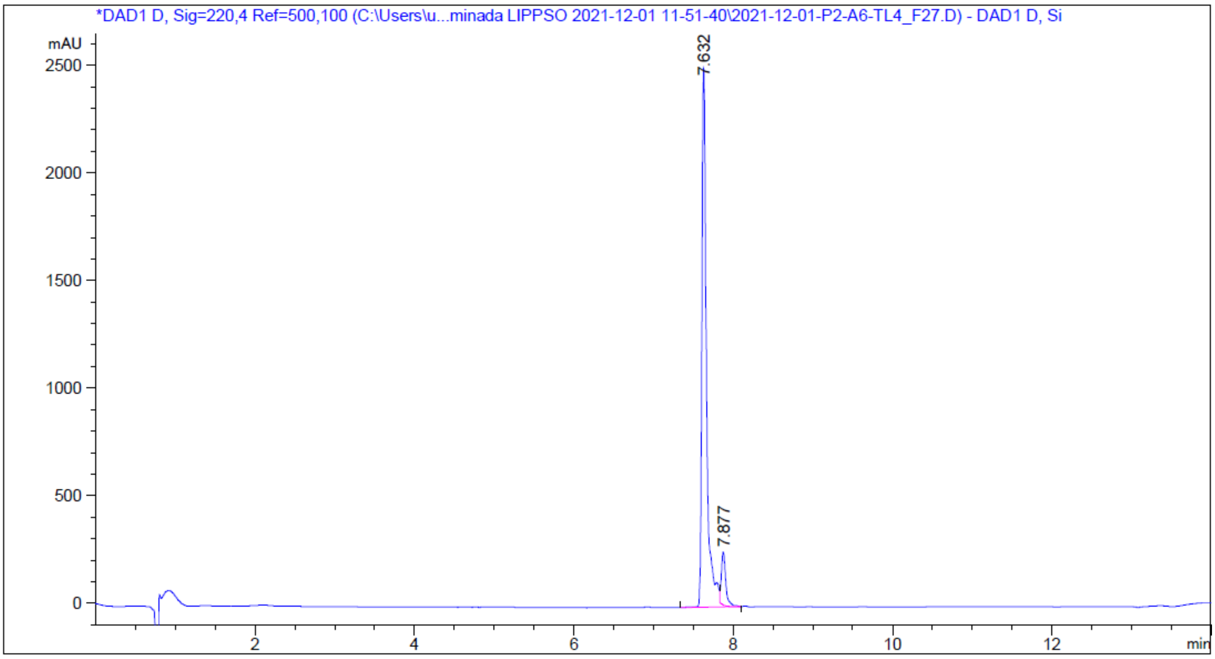

Peak #	RetTime [min]	Type	Width [min]	Area [mAU*s]	Height [mAU]	Area %
1	7.632	BV R	0.0618	1.02783e4	2507.92700	91.5710
2	7.877	VB E	0.0577	946.10846	246.68805	8.4290
Totals :				1.12244e4	2754.61505	

(–)-ESI-MS (*m/z*)

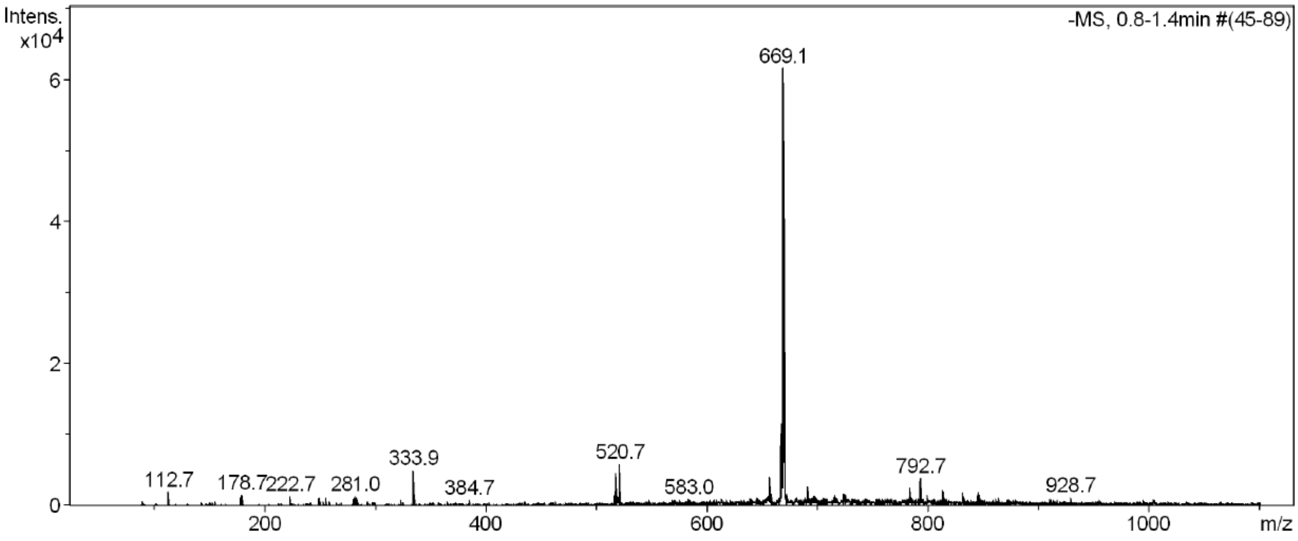

(–)-ESI-MS^2 (*m/z* = 669.1)

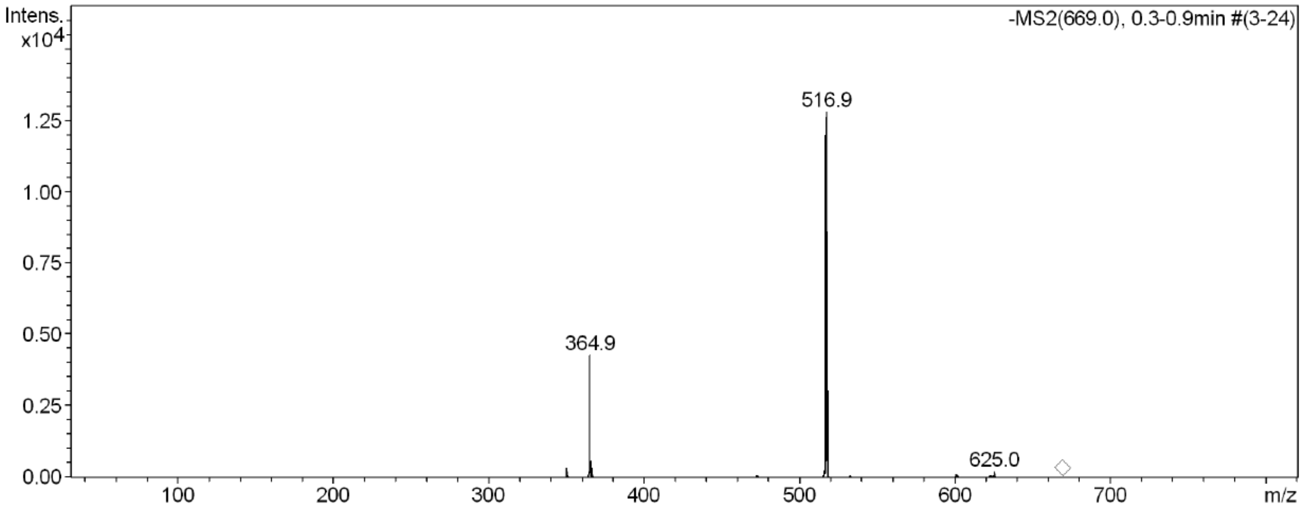

(–)-ESI-MS^3 (*m/z* = 516.9)

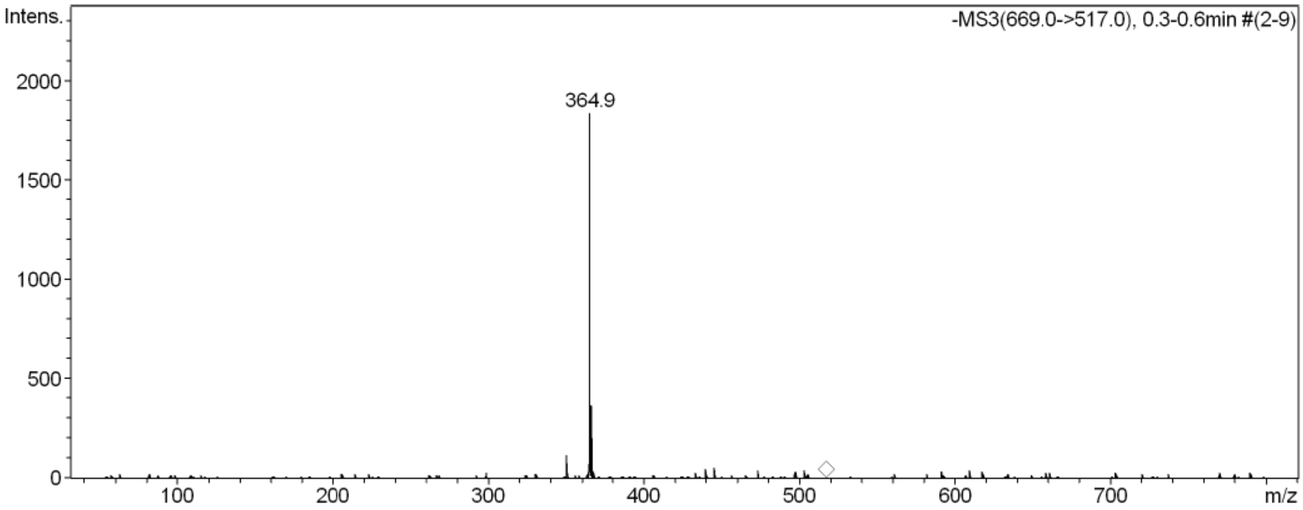

HRMS (*m/z*)

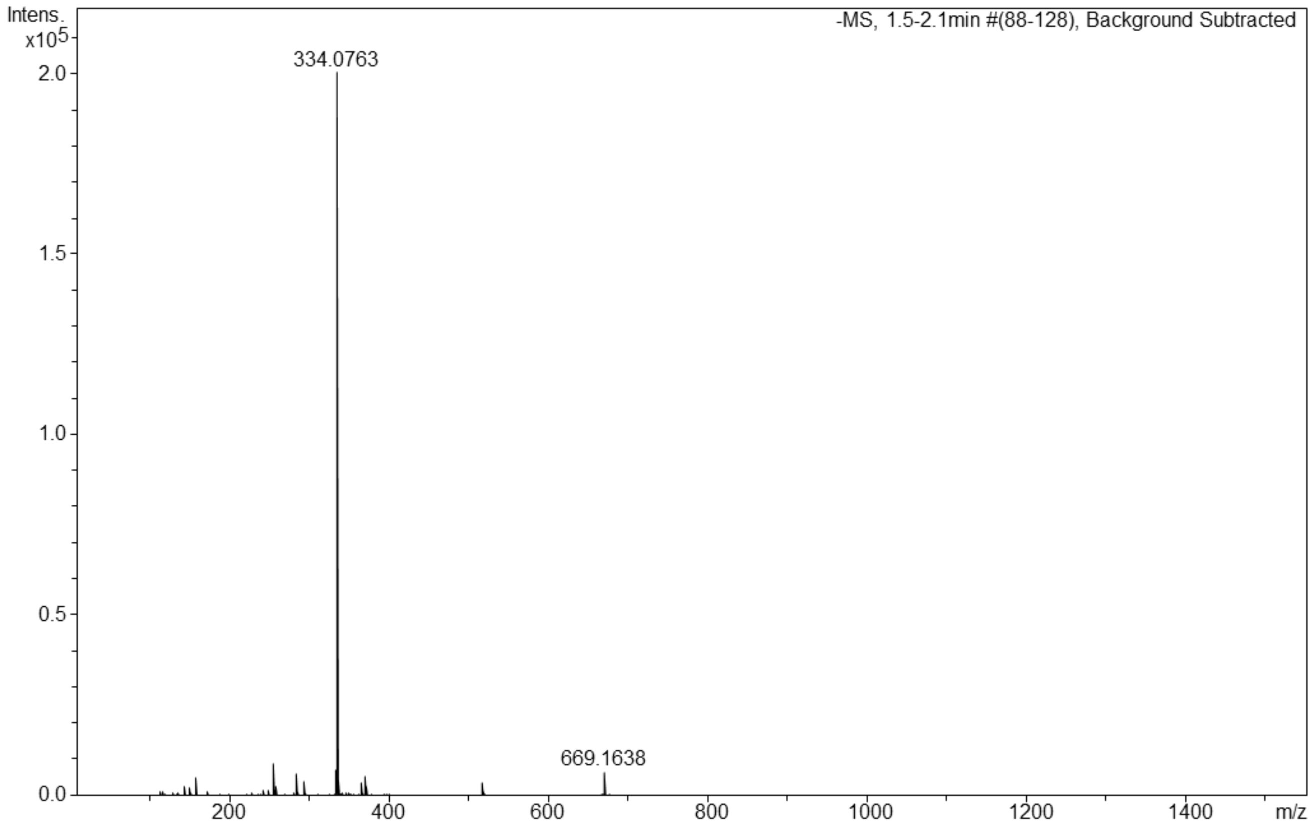
Intens.
x10^5
-MS, 1.5-2.1min #(88-128), Background Subtracted
334.0763
669.1638
2.0
1.5
1.0
0.5
0.0
200
400
600
800
1000
1200
1400
m/z

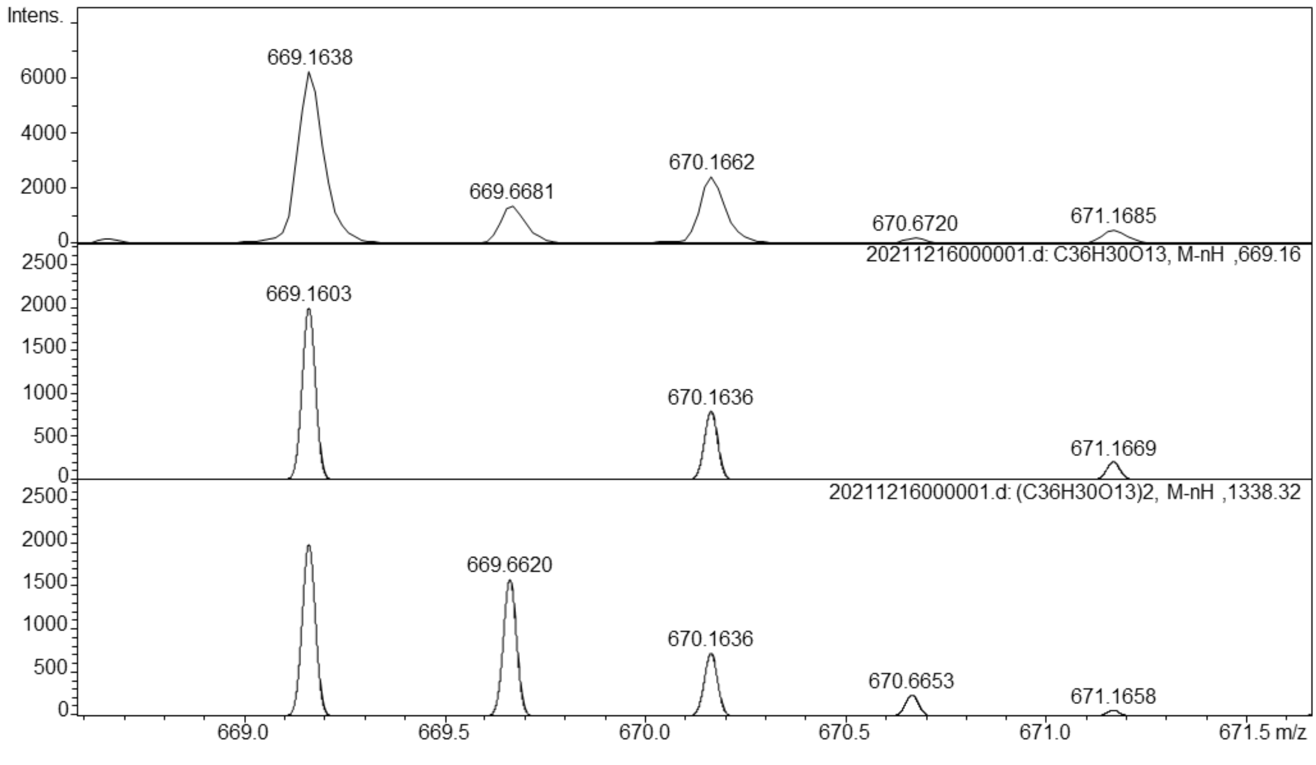
Intens.
669.1638
669.6681
670.1662
670.6720
671.1685
20211216000001.d: C36H30O13, M-nH ,669.16
669.1603
670.1636
671.1669
20211216000001.d: (C36H30O13)2, M-nH ,1338.32
669.6620
670.1636
670.6653
671.1658
669.0
669.5
670.0
670.5
671.0
671.5 m/z

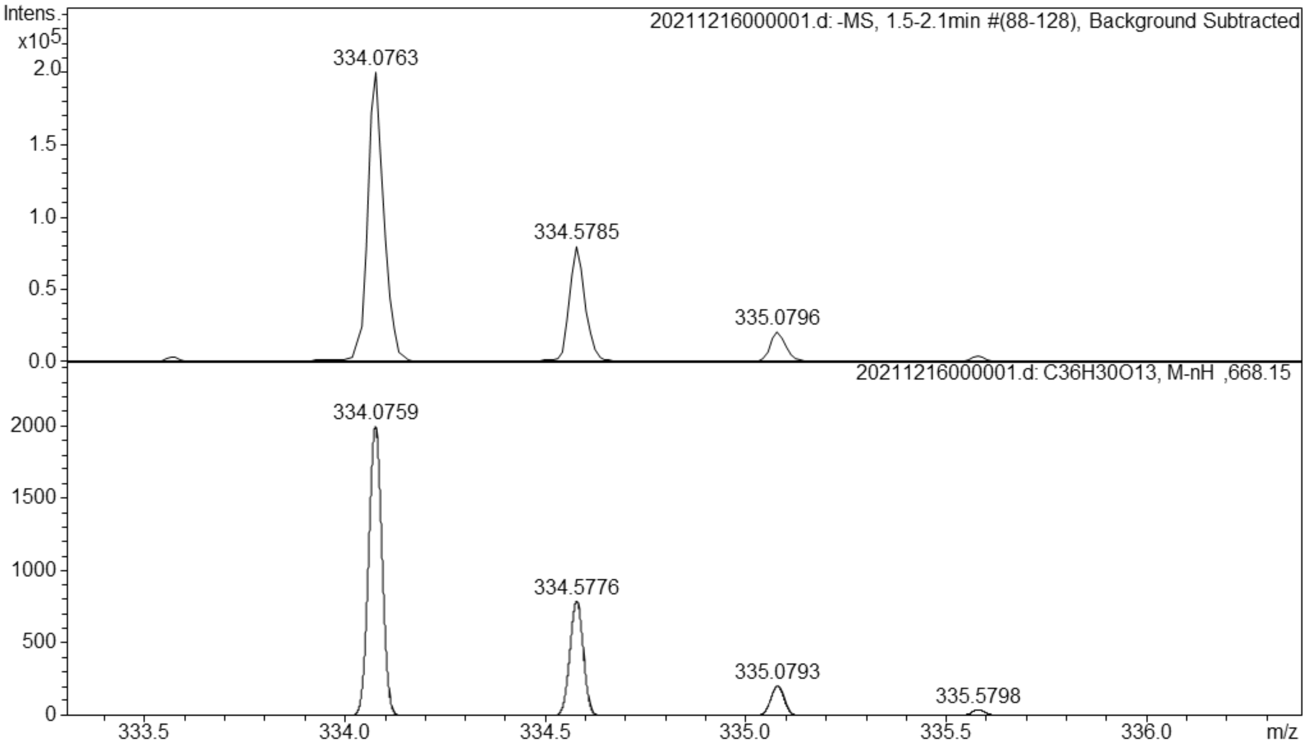

	Molecular formula	Calculated	Found
**[M–H]⁻**	$C_{36}H_{29}O_{13}$	669.1603	669.1638
**[2M–2H]⁻**	$(C_{36}H_{29}O_{13})_2$	669.1603	669.1638
**[M–2H]²⁻**	$C_{36}H_{28}O_{13}$	334.0759	334.0763

2.13. ((1*E*,1′*Z*)-(2-Oxocyclohexane-1,3-diylidene)bis(methanylylidene))bis(2-methoxy-4,1-phenylene) bis[3,4,5-tris((*tert*-butyldimethylsilyl)oxy)benzoate] (TL5)

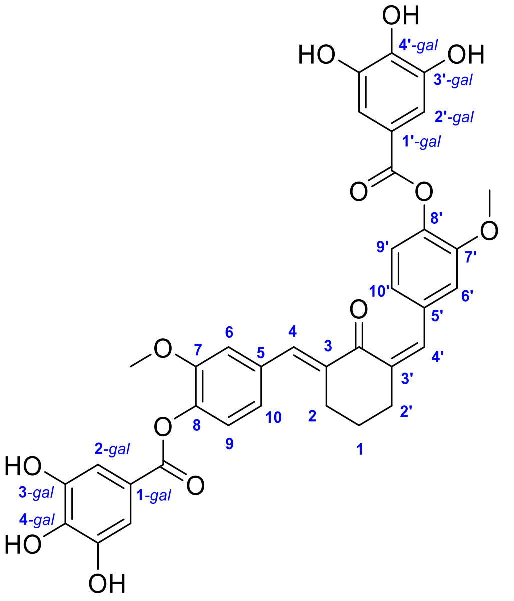

^{1}H-NMR (400 MHz, $(CD_3)_2CO$), *δ* (ppm)

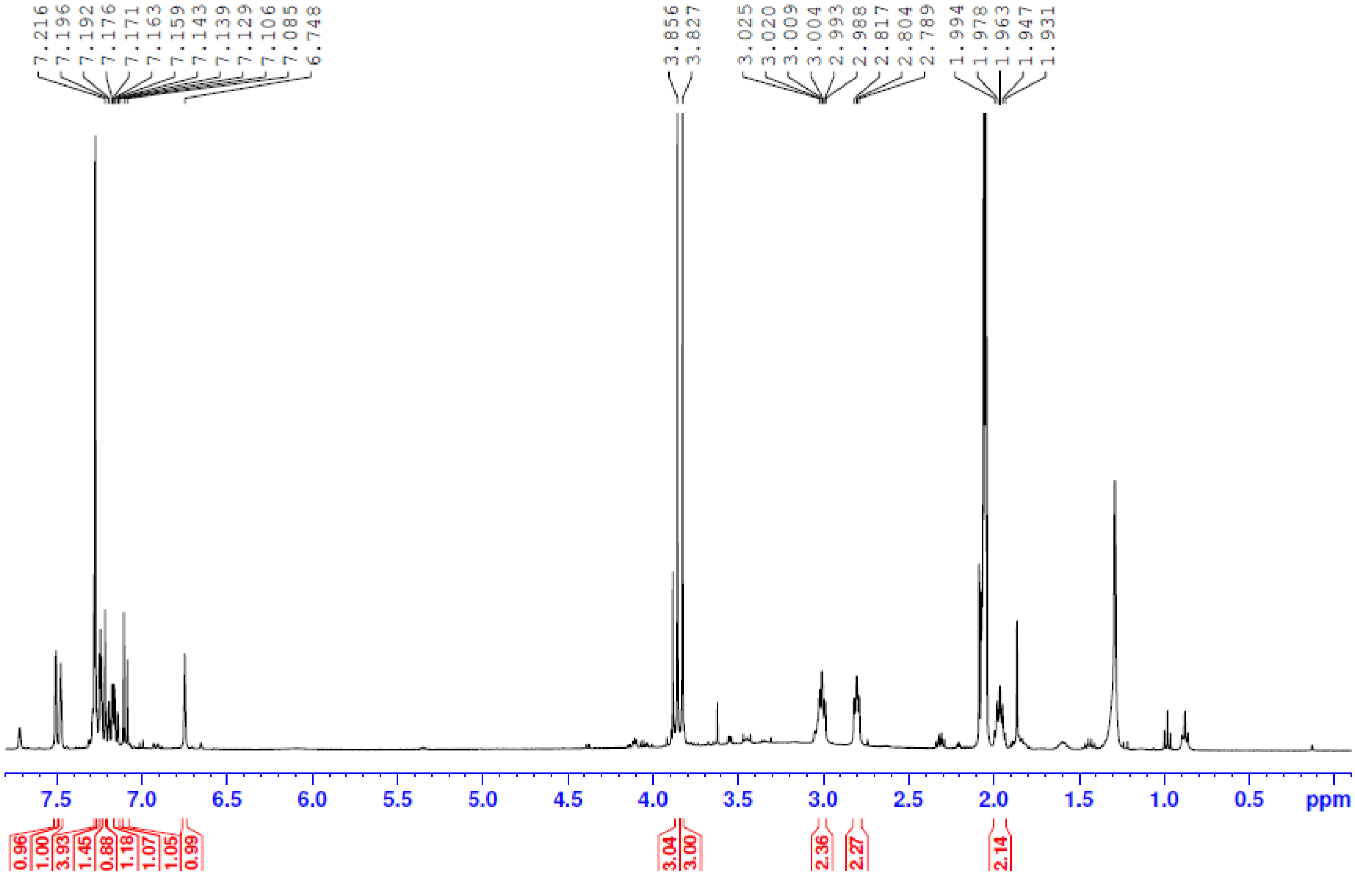

^{1}H-NMR (400 MHz, $(CD_3)_2CO$), δ (ppm) – Aromatic zoom

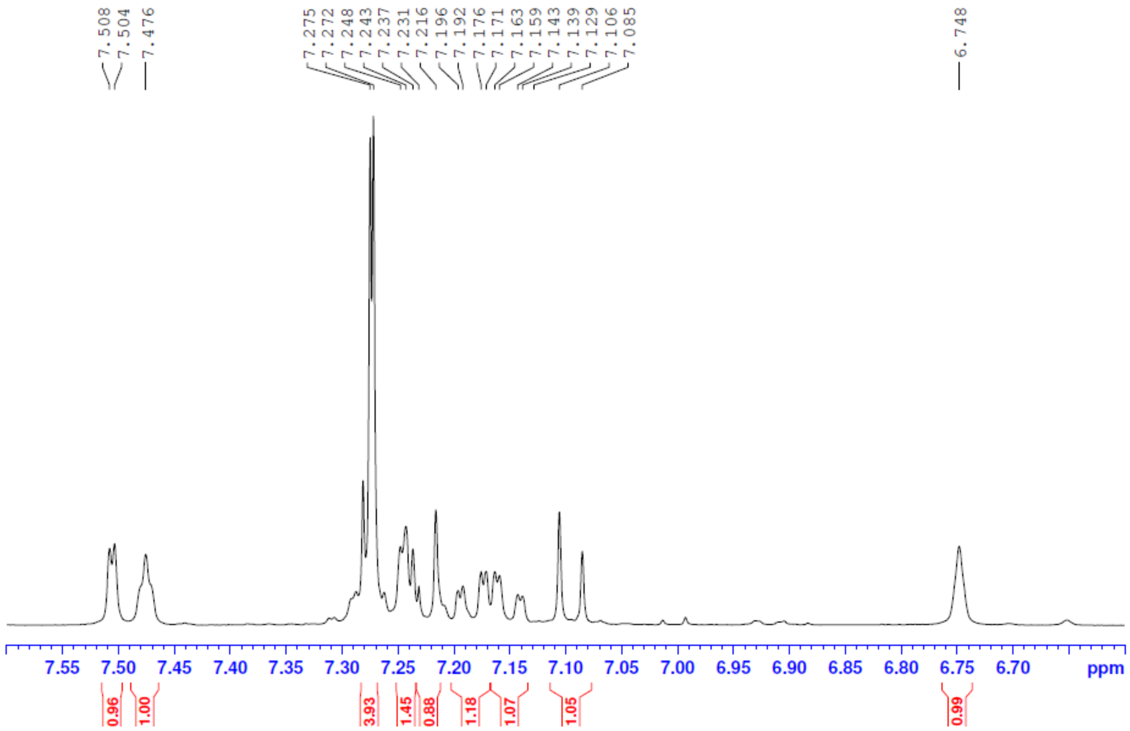

COSY ^{1}H-^{1}H ($(CD_3)_2CO$), δ (ppm)

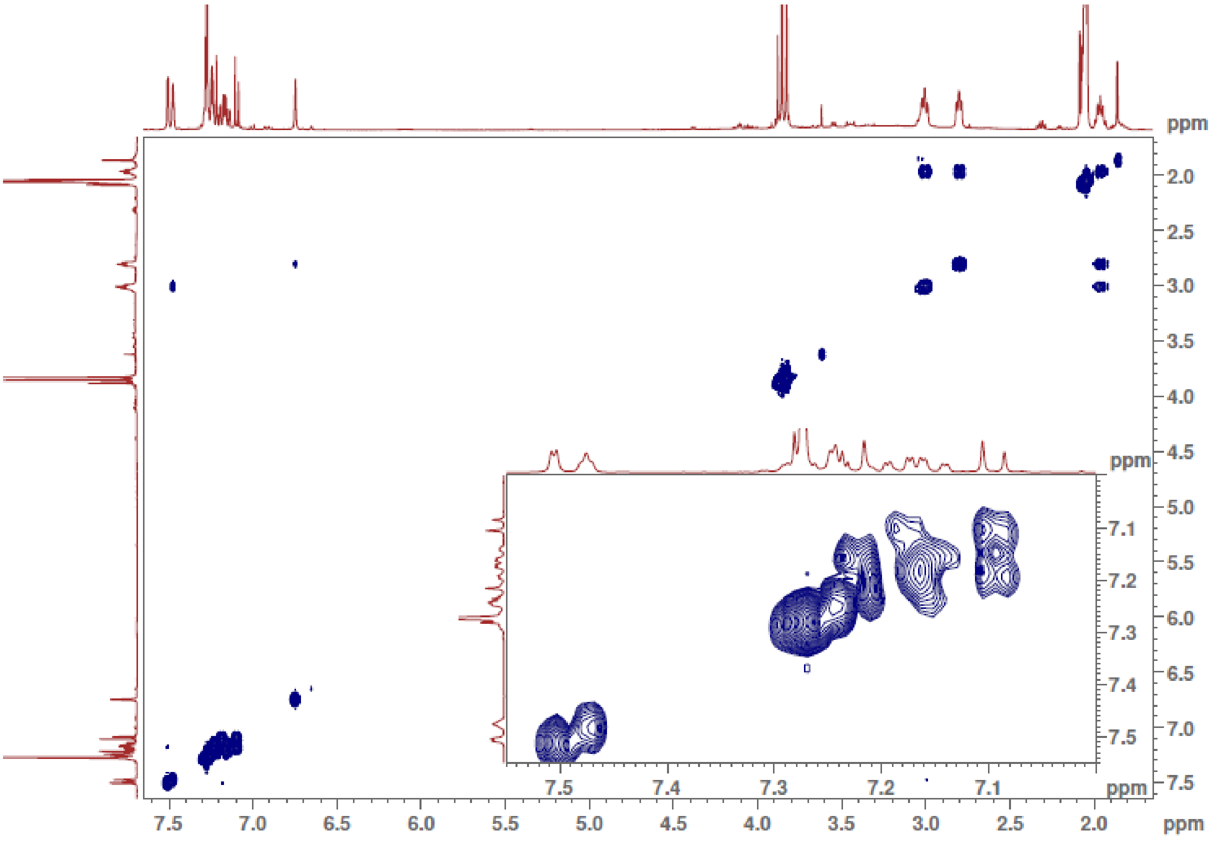

^{13}C-NMR (100 MHz, $(CD_3)_2CO$), δ (ppm)

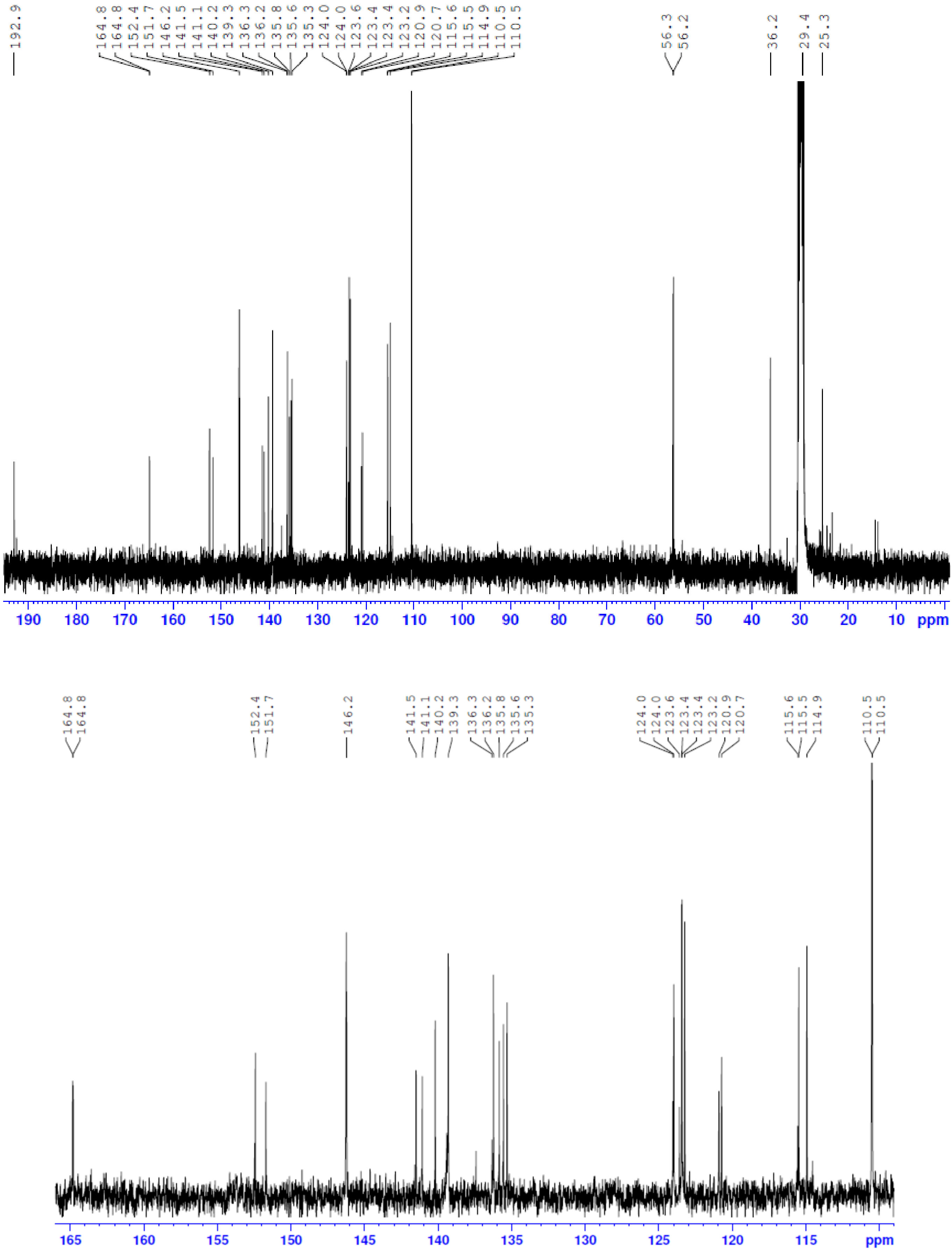

HSQC ^{1}H-^{13}C ($(CD_3)_2CO$), δ (ppm)

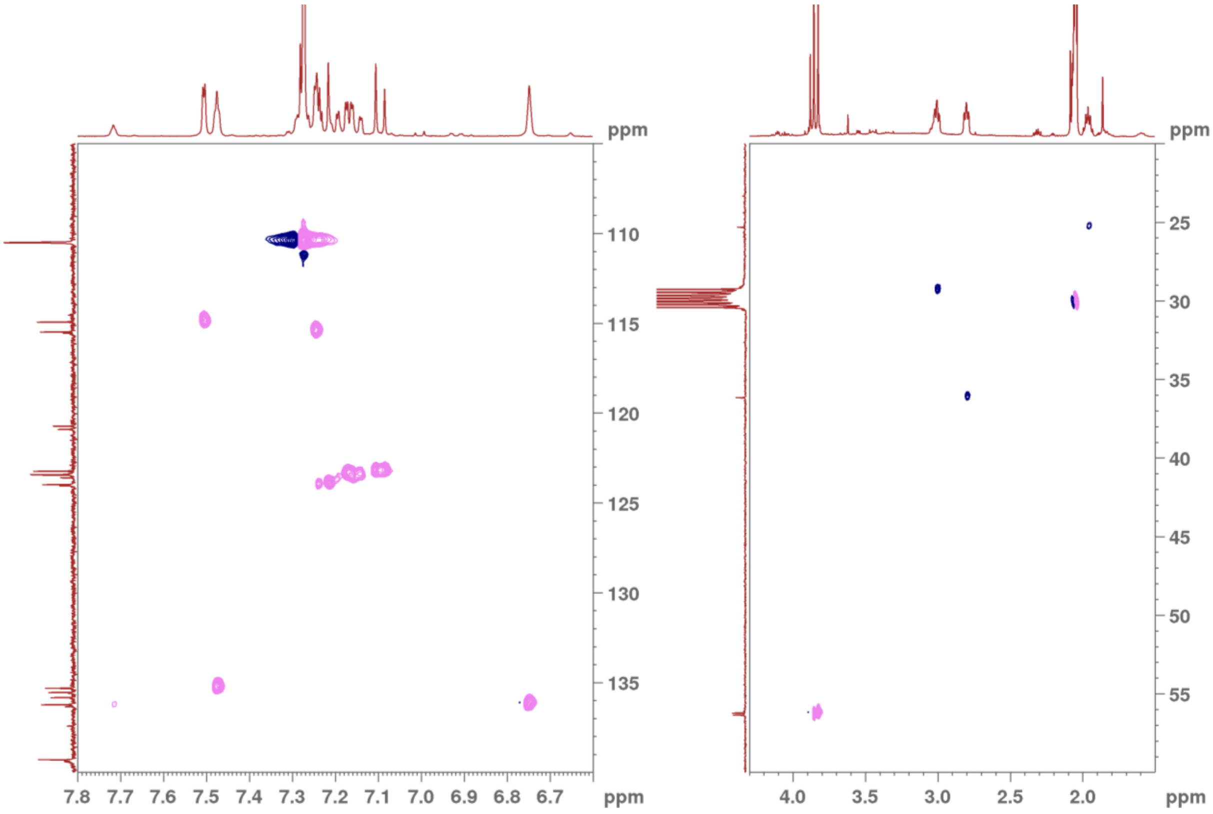

HMBC ^{1}H-^{13}C ($(CD_3)_2CO$), δ (ppm)

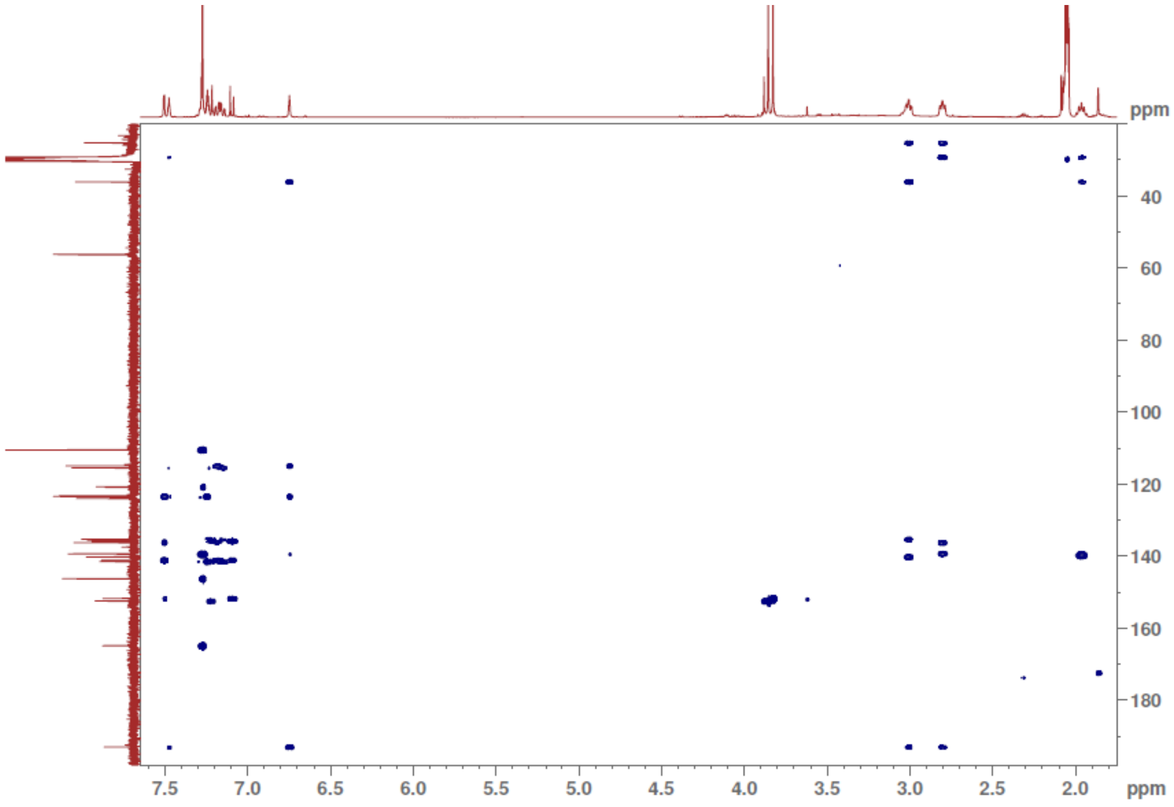

NOESY ^{1}H-^{1}H ($(CD_3)_2CO$), δ (ppm)

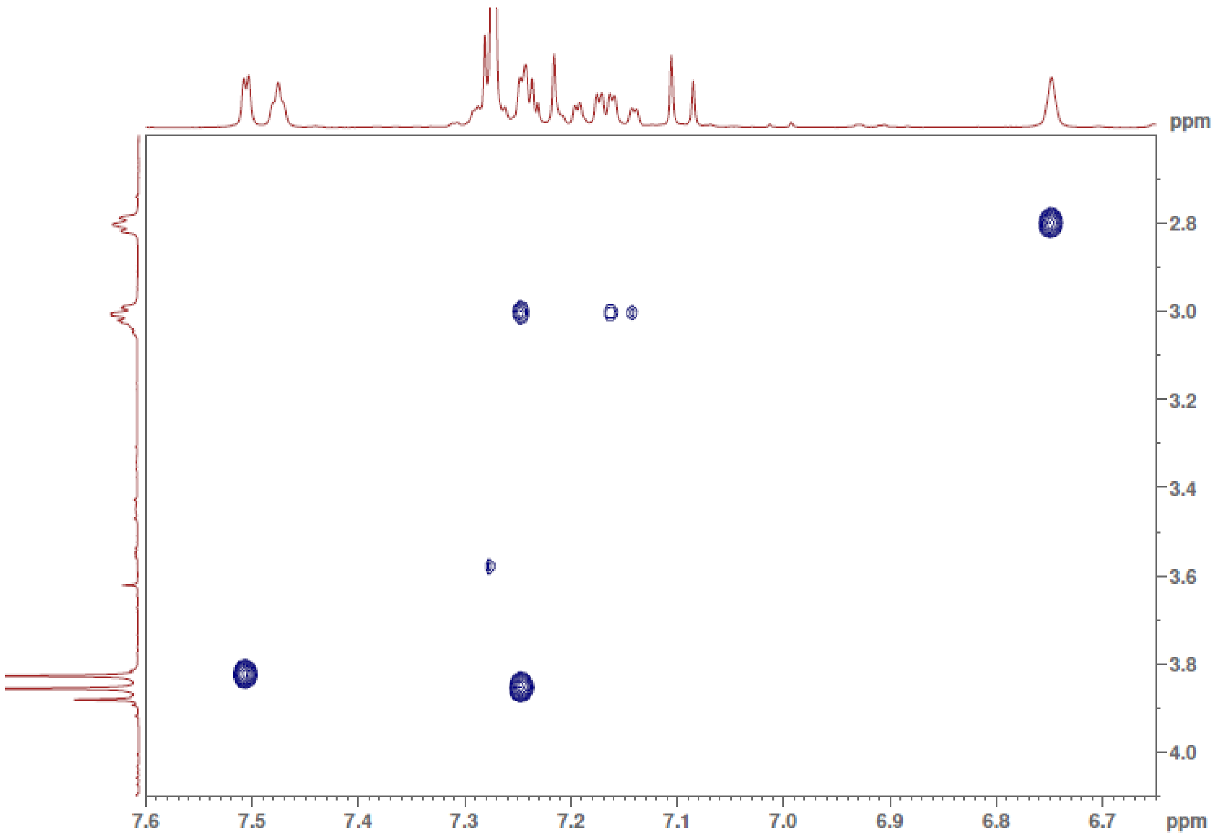

FT-IR (neat), ν (cm^{-1})

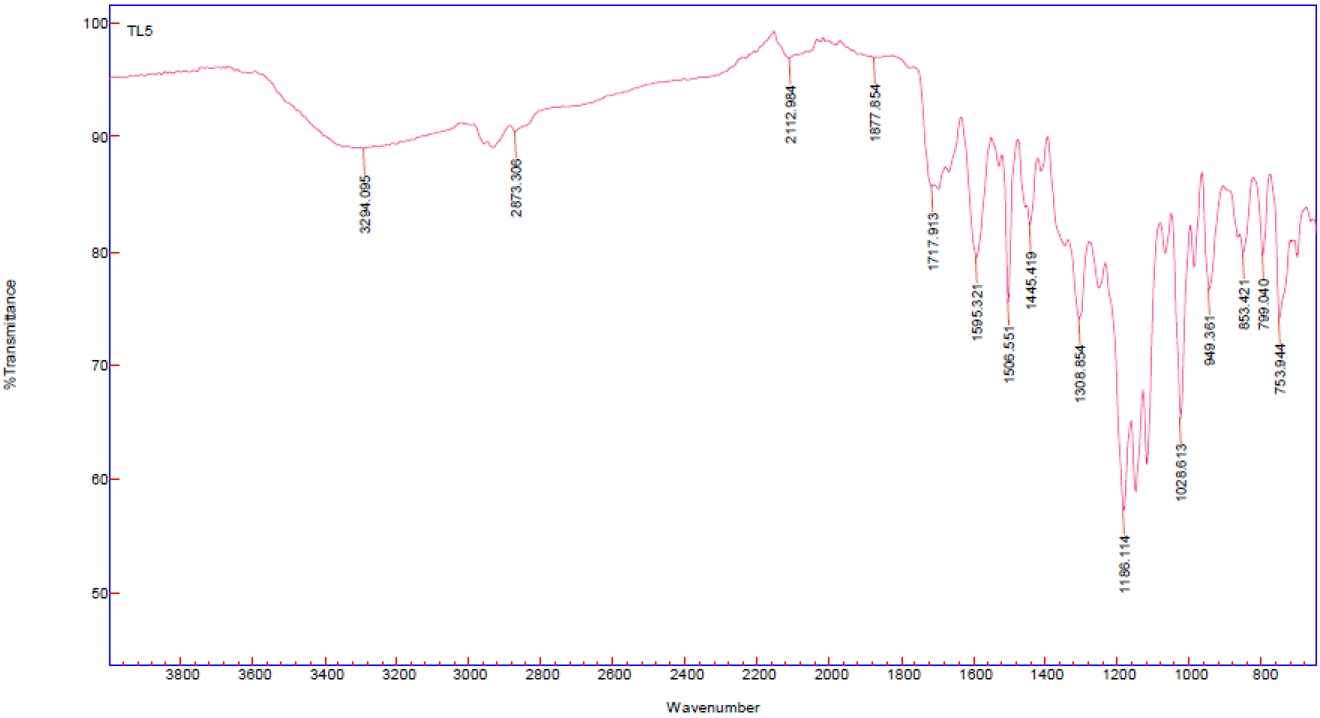

UV-Vis (CH_3CN), λ (nm)

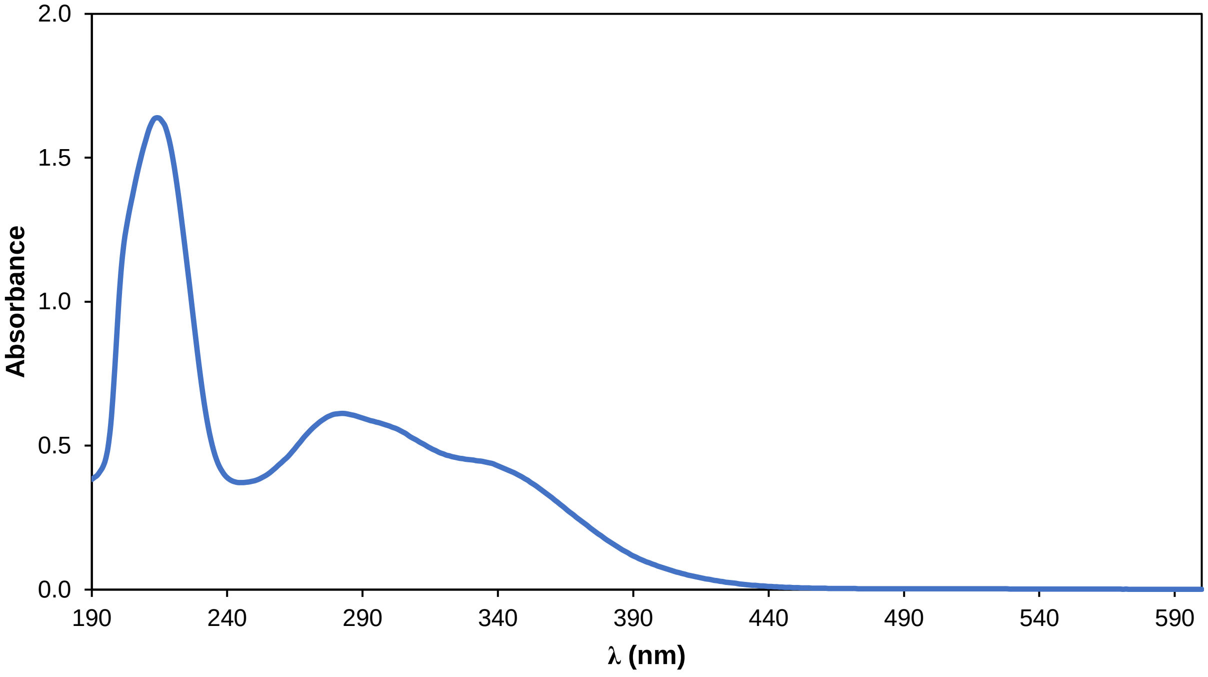

HPLC (λ = 220 nm)

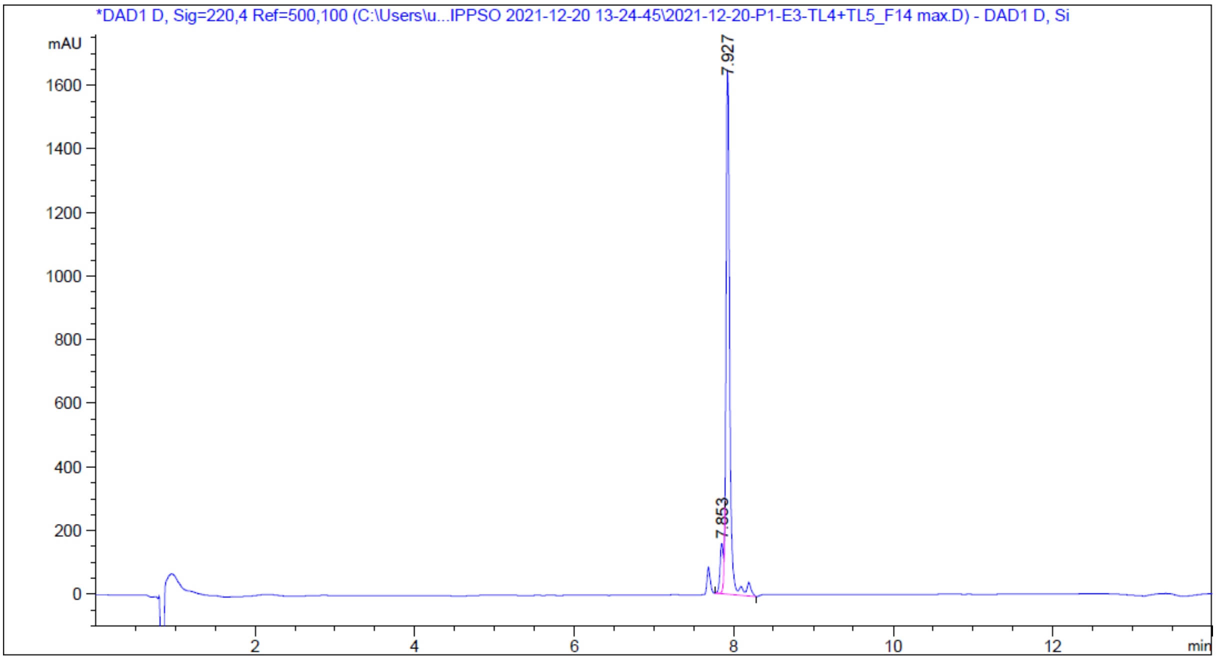

Peak #	RetTime [min]	Type	Width [min]	Area [mAU*s]	Height [mAU]	Area %
1	7.853	BV E	0.0401	399.56467	149.81126	6.9752
2	7.927	VV R	0.0487	5328.81641	1647.83044	93.0248
Totals :				5728.38107	1797.64171	

(–)-ESI-MS (*m/z*)

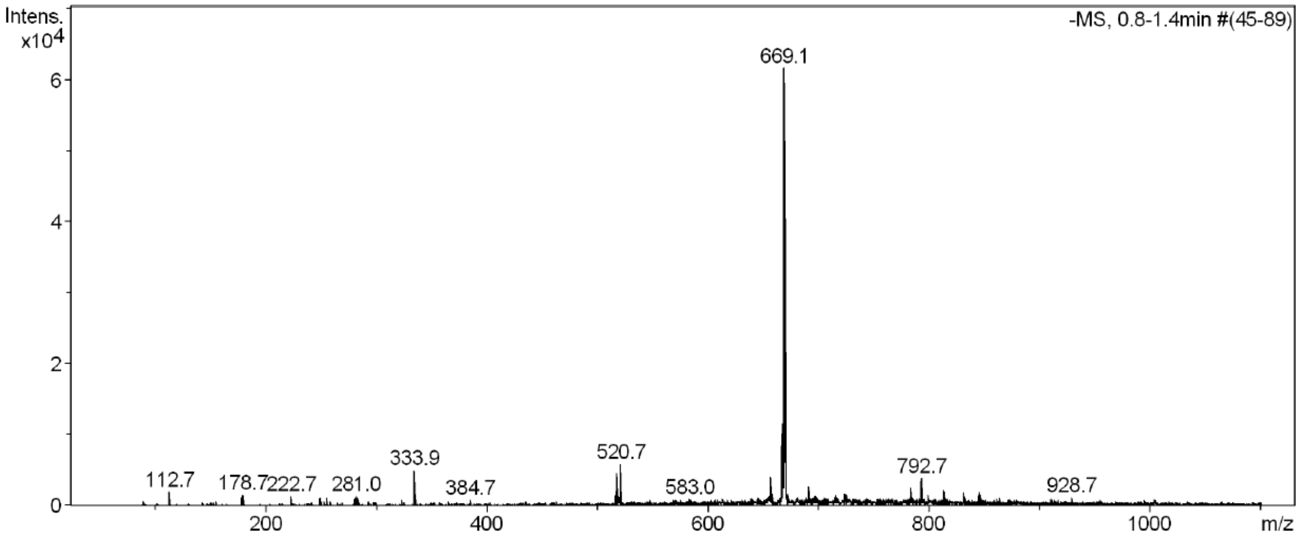

Intens.
x10^4
-MS, 0.8-1.4min #(45-89)
669.1
112.7
178.7
222.7
281.0
333.9
384.7
520.7
583.0
792.7
928.7
0
2
4
6
200
400
600
800
1000
m/z

HRMS (*m/z*)

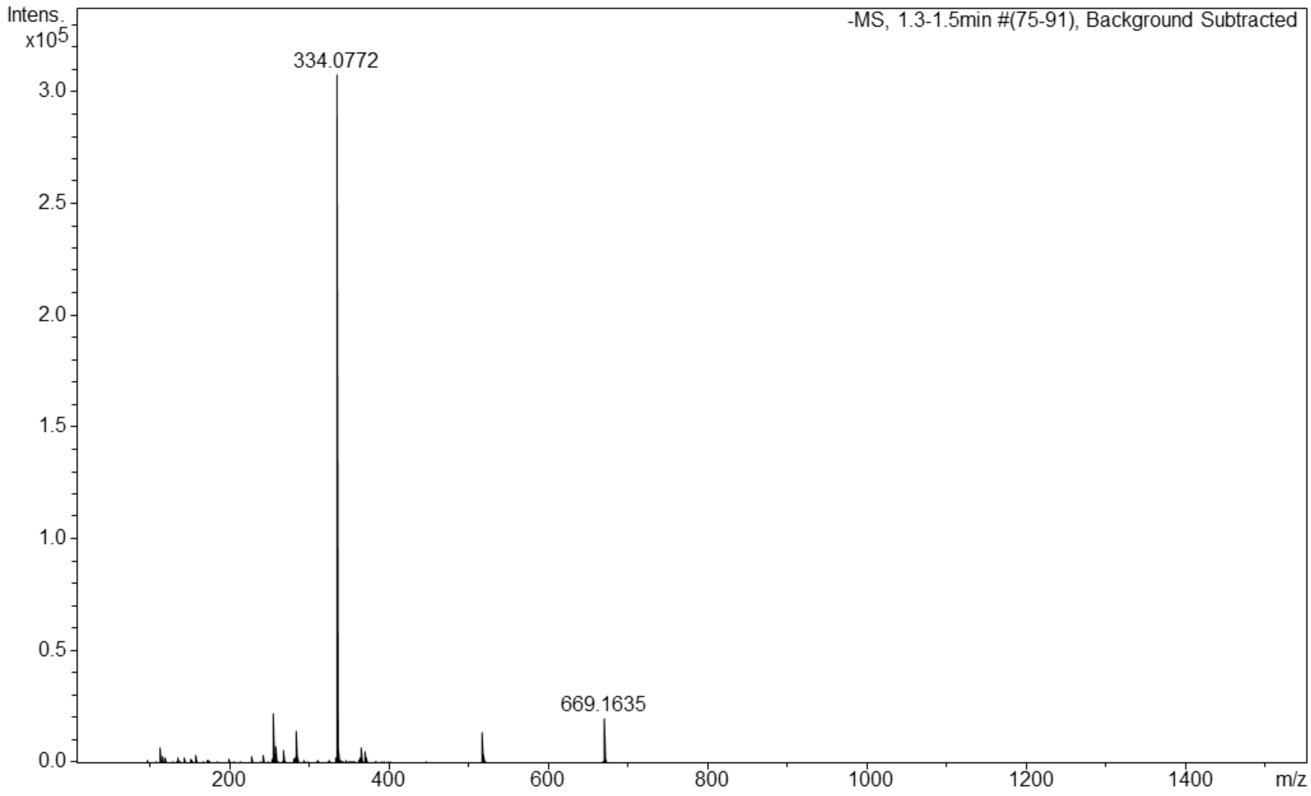
Intens.
x10^5
-MS, 1.3-1.5min #(75-91), Background Subtracted
334.0772
669.1635
3.0
2.5
2.0
1.5
1.0
0.5
0.0
200
400
600
800
1000
1200
1400
m/z

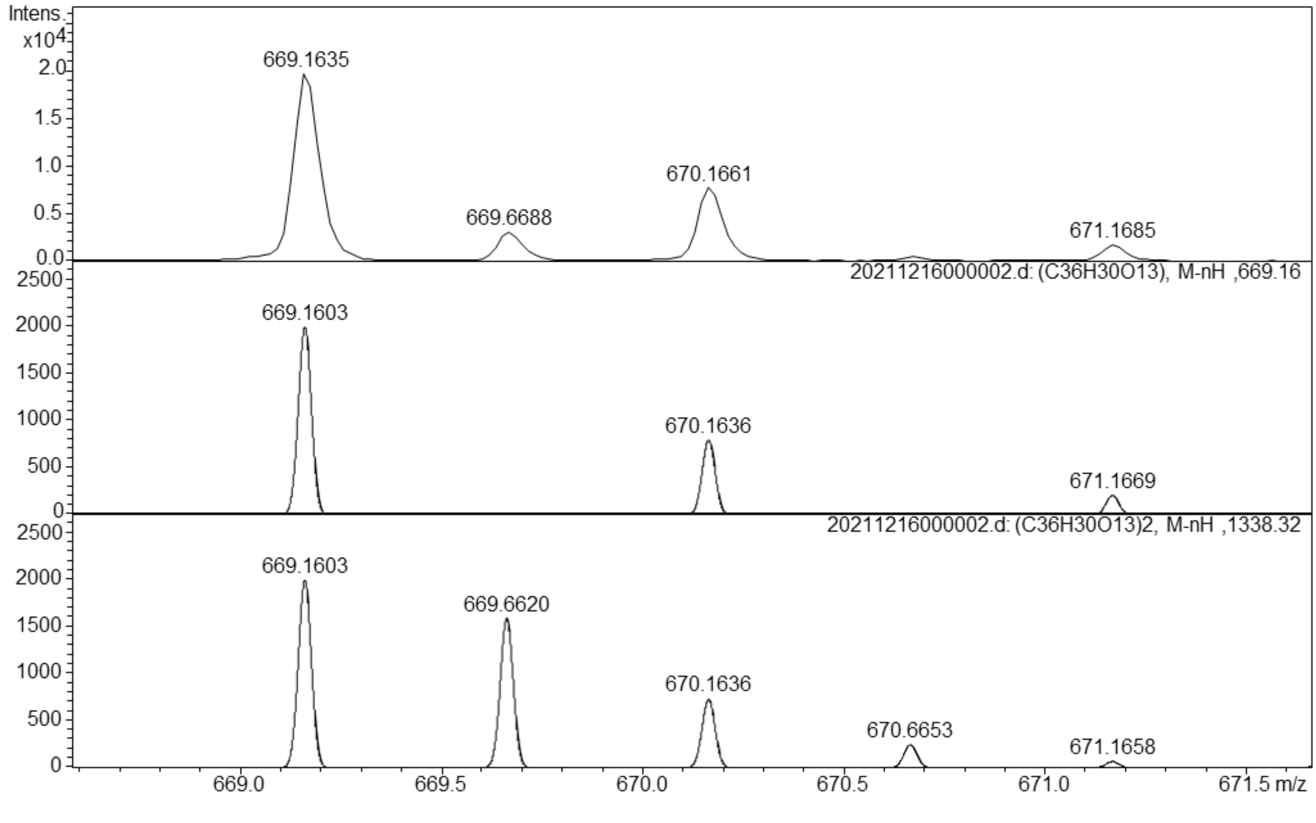
Intens.
x10^4
669.1635
669.6688
670.1661
671.1685
20211216000002.d: (C36H30O13), M-nH ,669.16
669.1603
670.1636
671.1669
20211216000002.d: (C36H30O13)2, M-nH ,1338.32
669.1603
669.6620
670.1636
670.6653
671.1658
669.0
669.5
670.0
670.5
671.0
671.5 m/z

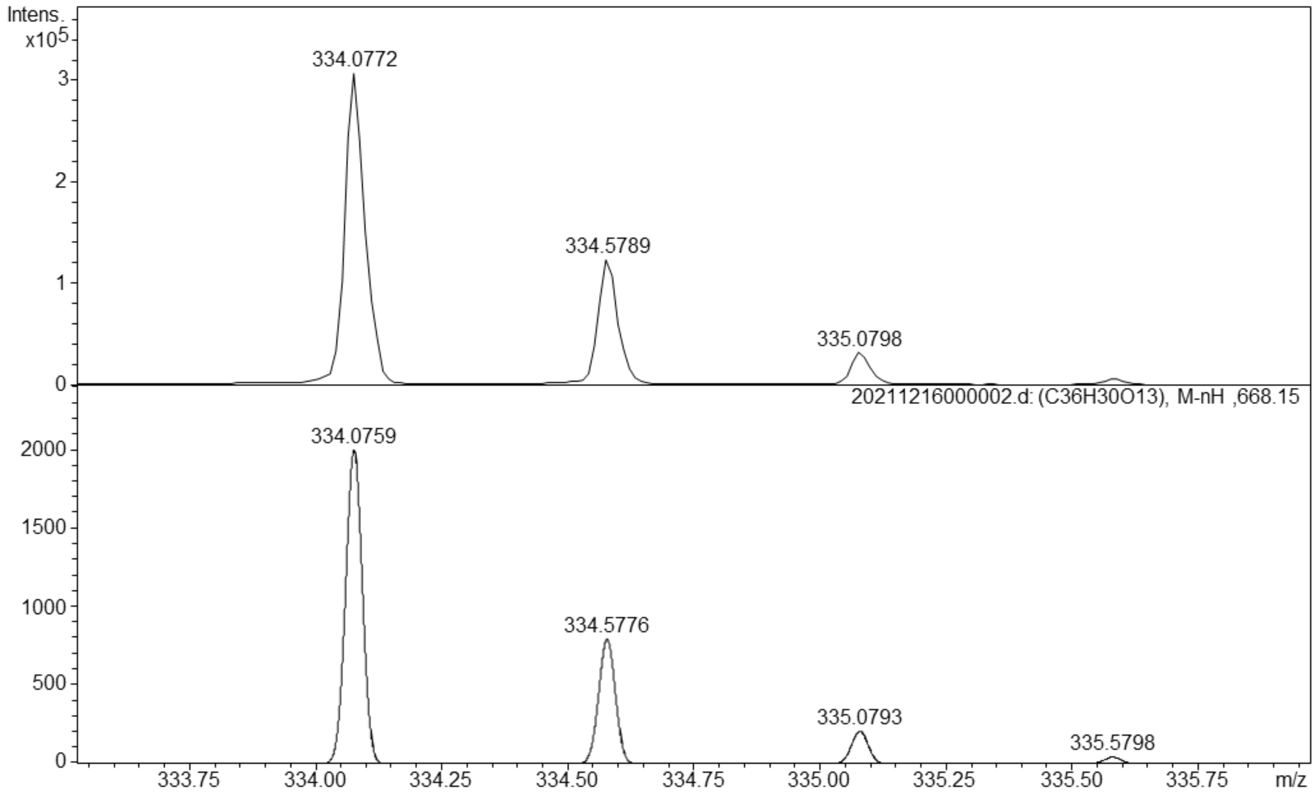

	Molecular formula	Calculated	Found
**[M–H]⁻**	$C_{36}H_{29}O_{13}$	669.1603	669.1635
**[2M–2H]⁻**	$(C_{36}H_{29}O_{13})_2$	669.1603	669.1635
**[M–2H]$^{2-}$**	$C_{36}H_{28}O_{13}$	334.0759	334.0772

2.14. ((1*E*,4*E*)-3-Oxopenta-1,4-diene-1,5-diyl)bis(2-methoxy-4,1-phenylene) bis[3,4,5-tris((*tert*-butyldimethylsilyl)oxy)benzoate] (TL6)

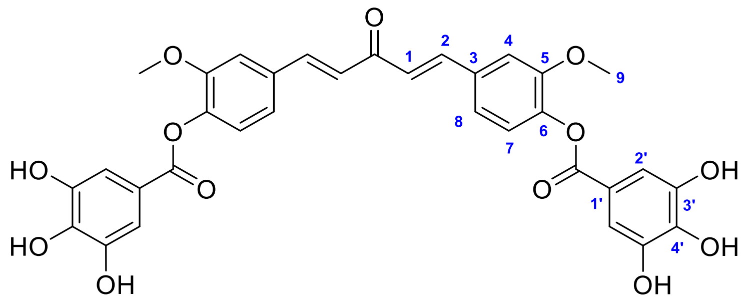

^{1}H-NMR (400 MHz, CD_3OD), δ (ppm)

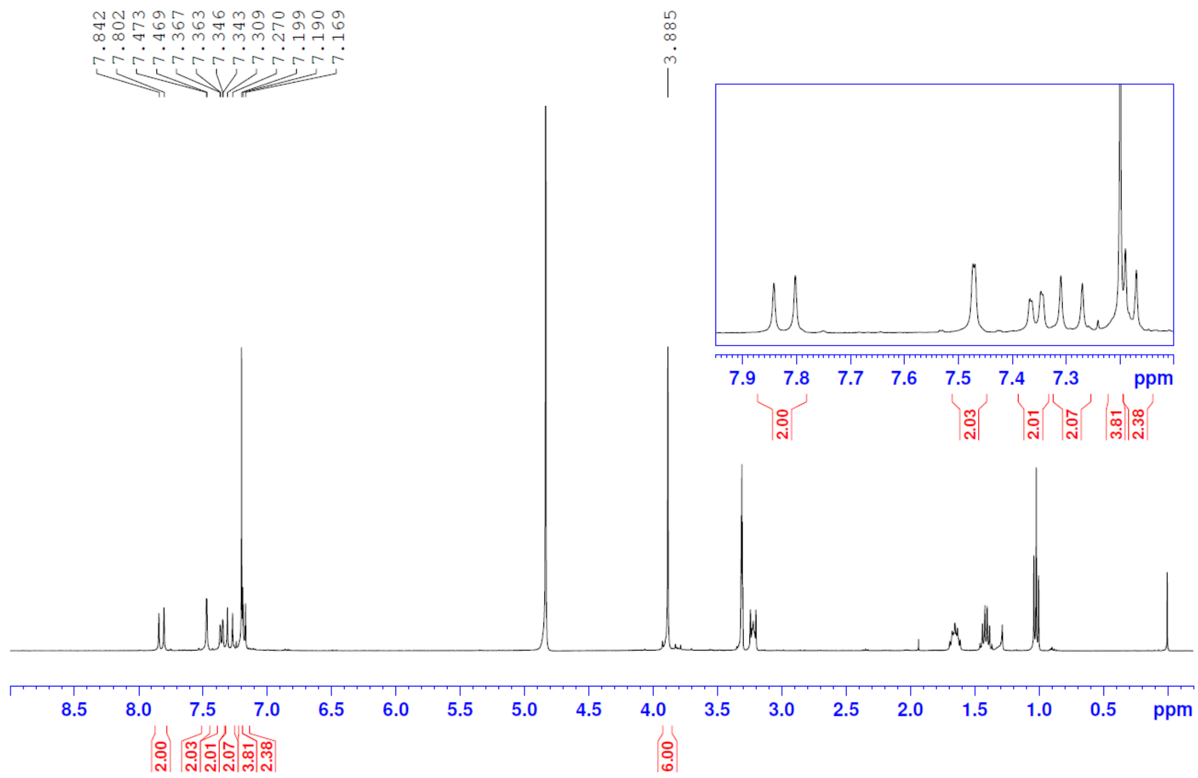

^{13}C-NMR (100 MHz, CD_3OD), δ (ppm)

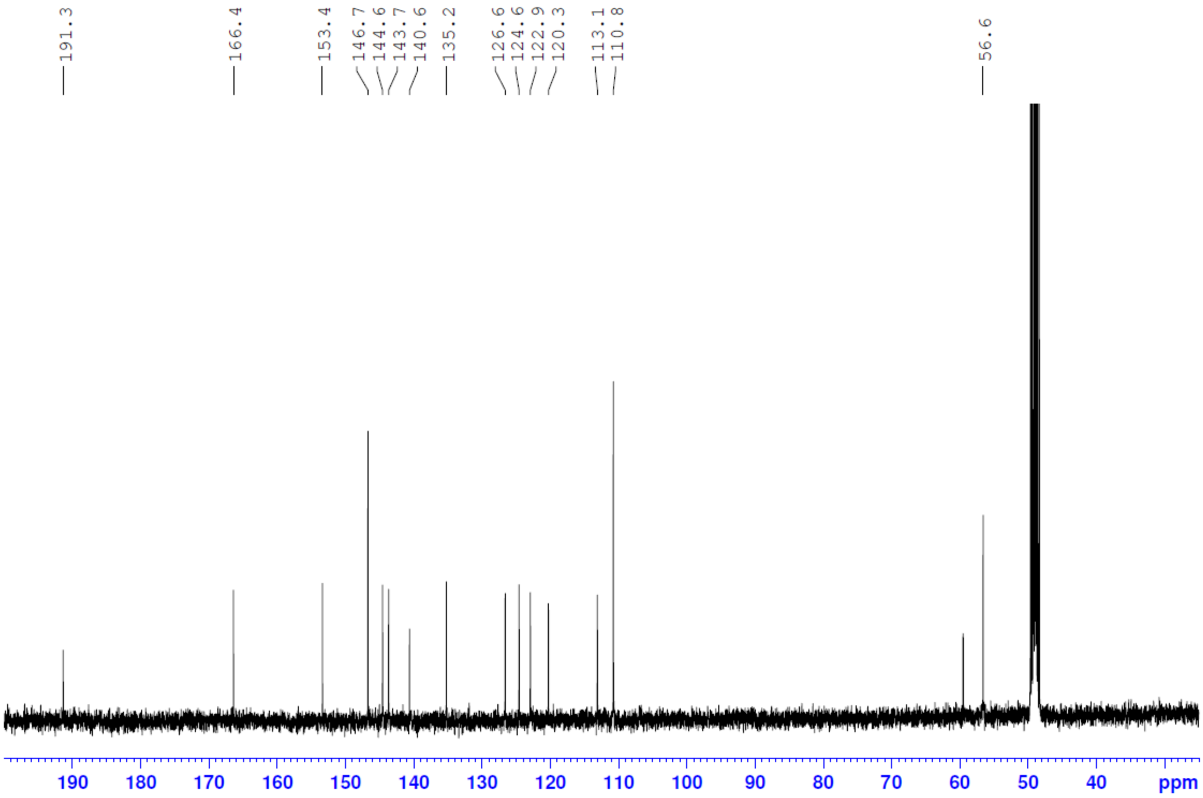

COSY ^{1}H-^{1}H (CD_3OD), δ (ppm)

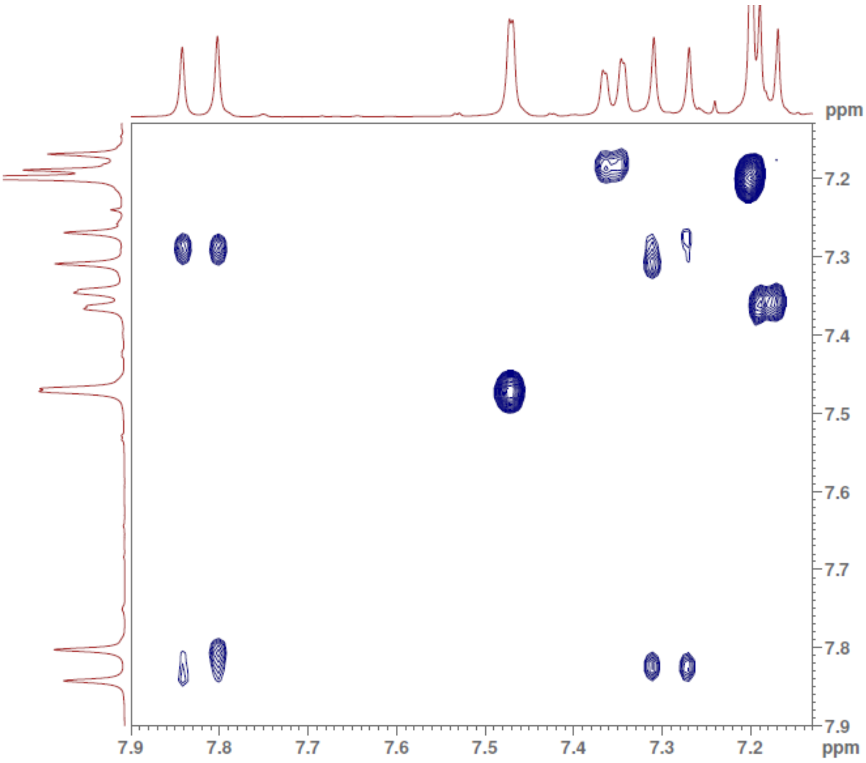

HSQC ^{1}H-^{13}C (CD_3OD), δ (ppm)

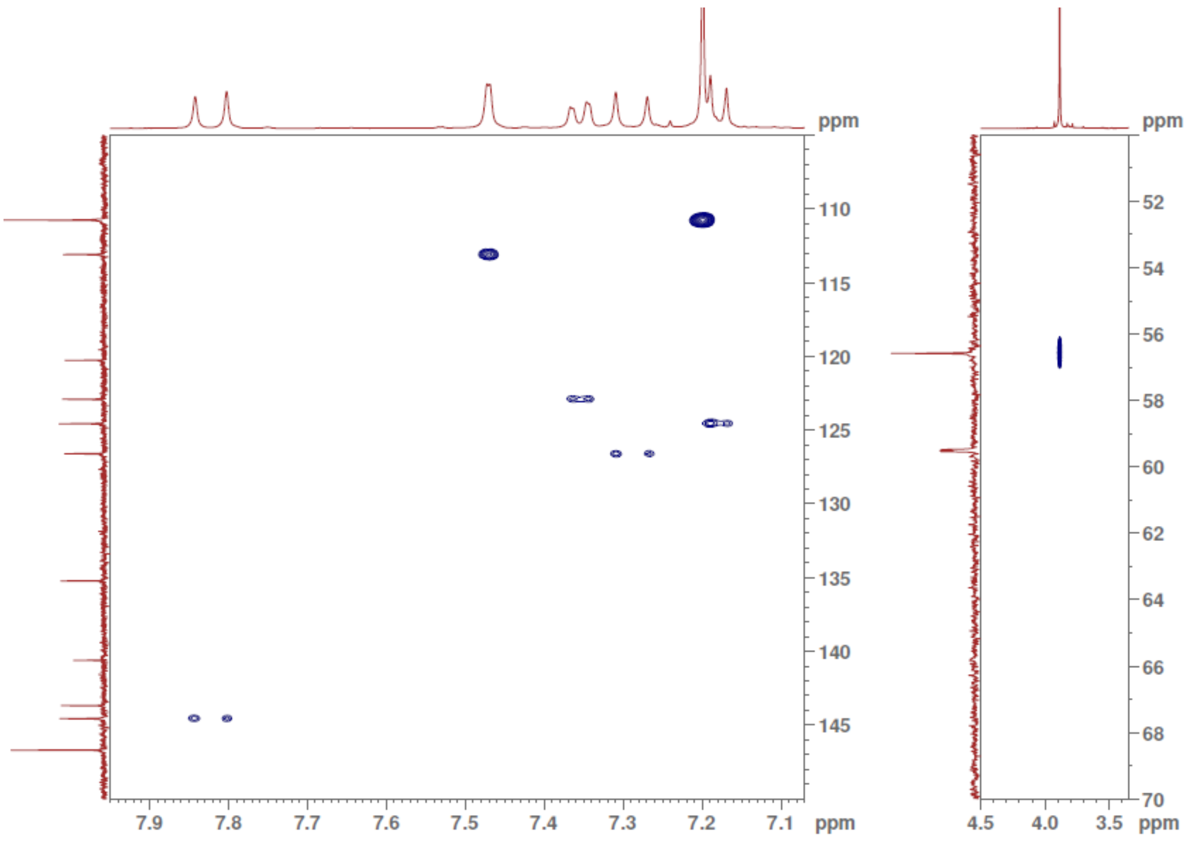

HMBC ^1H-^{13}C (CD_3OD), δ (ppm)

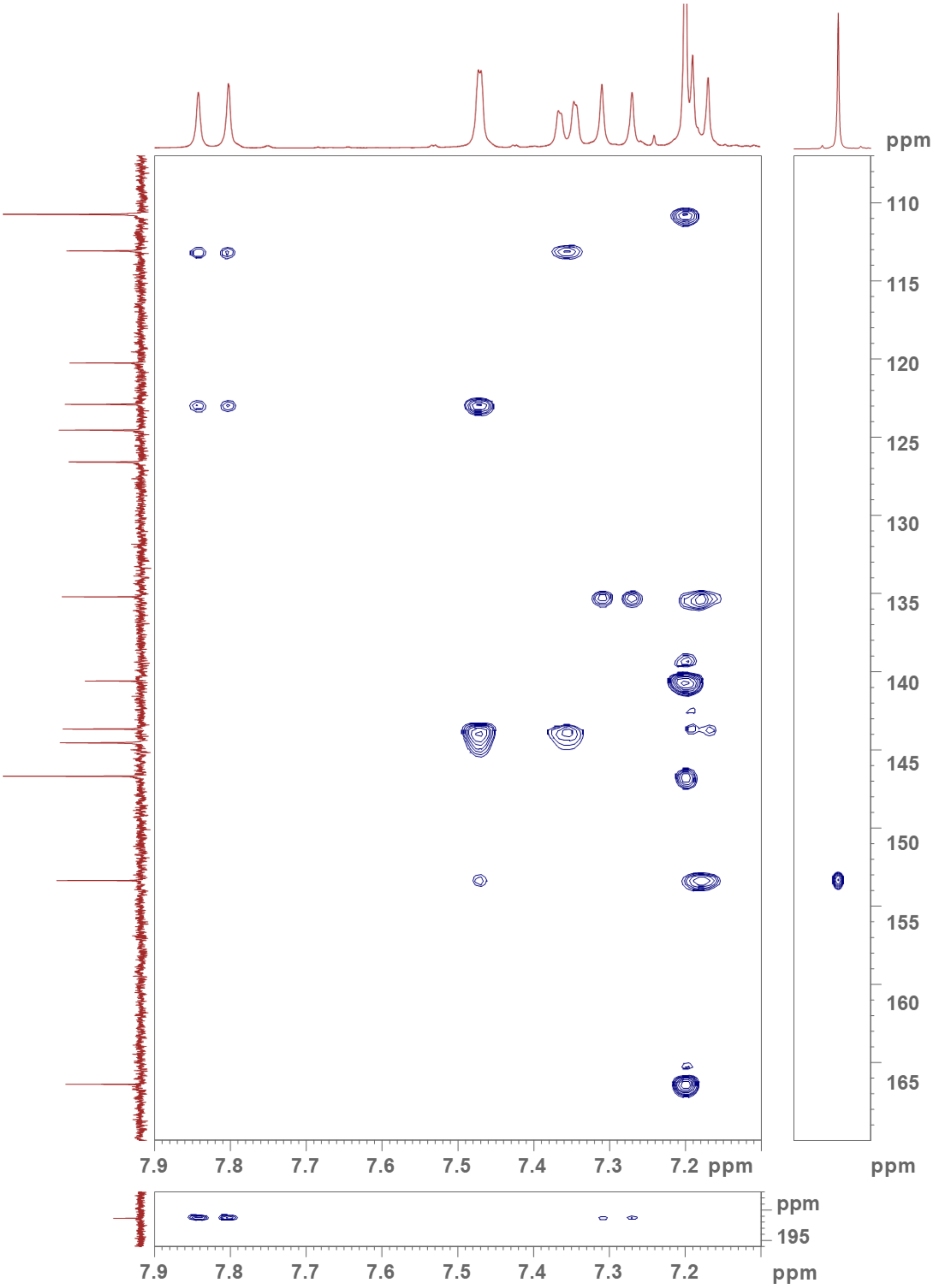

FT-IR (neat), ν (cm^{-1})

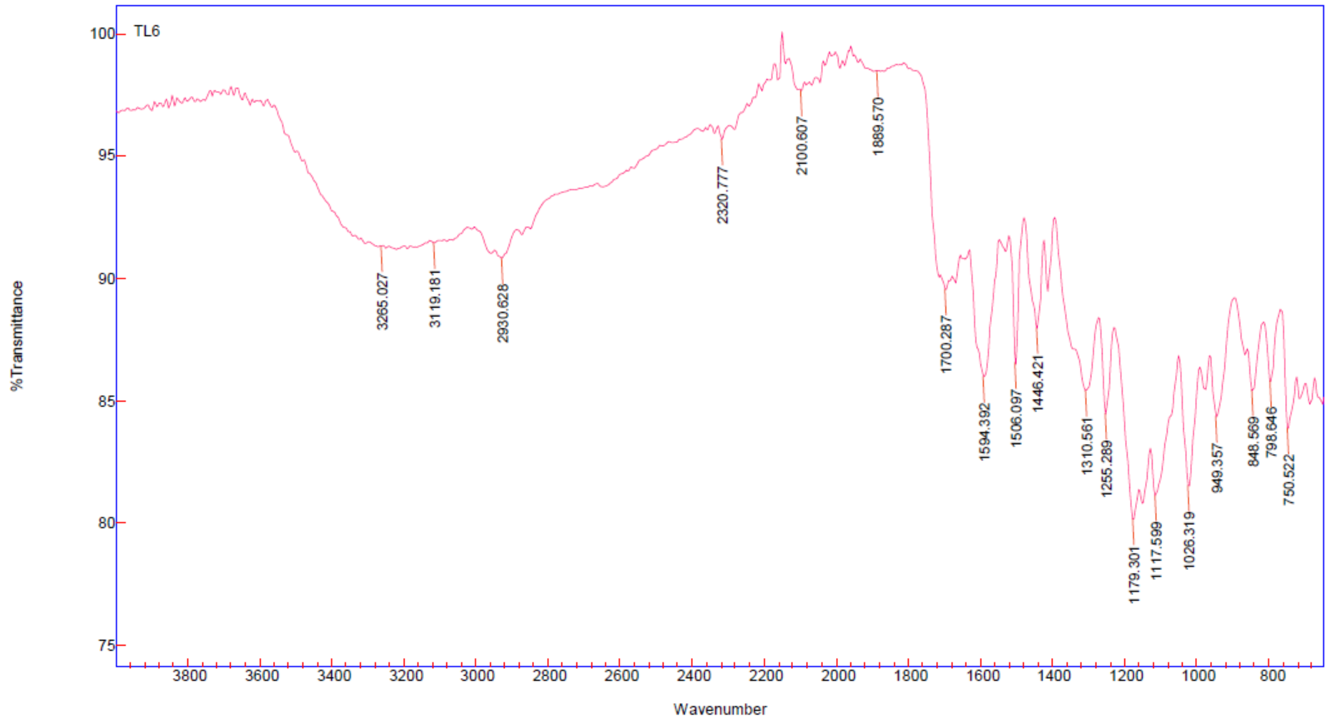

UV-Vis (CH_3CN), λ (nm)

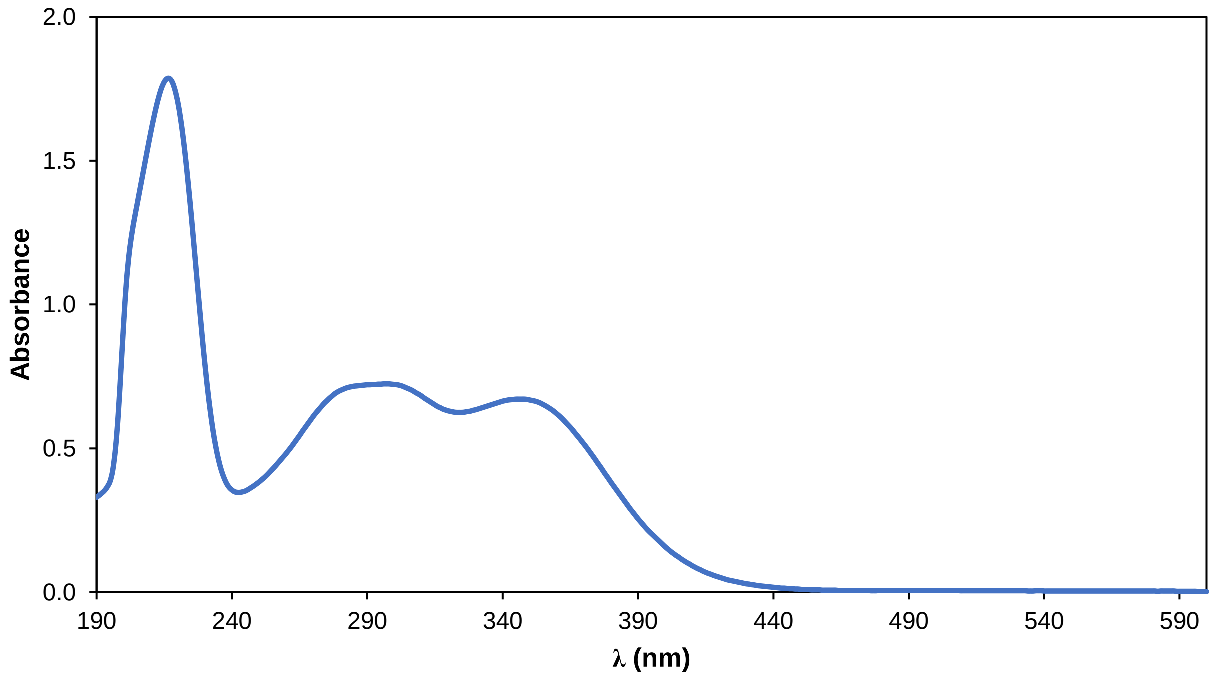

HPLC (λ = 220 nm)

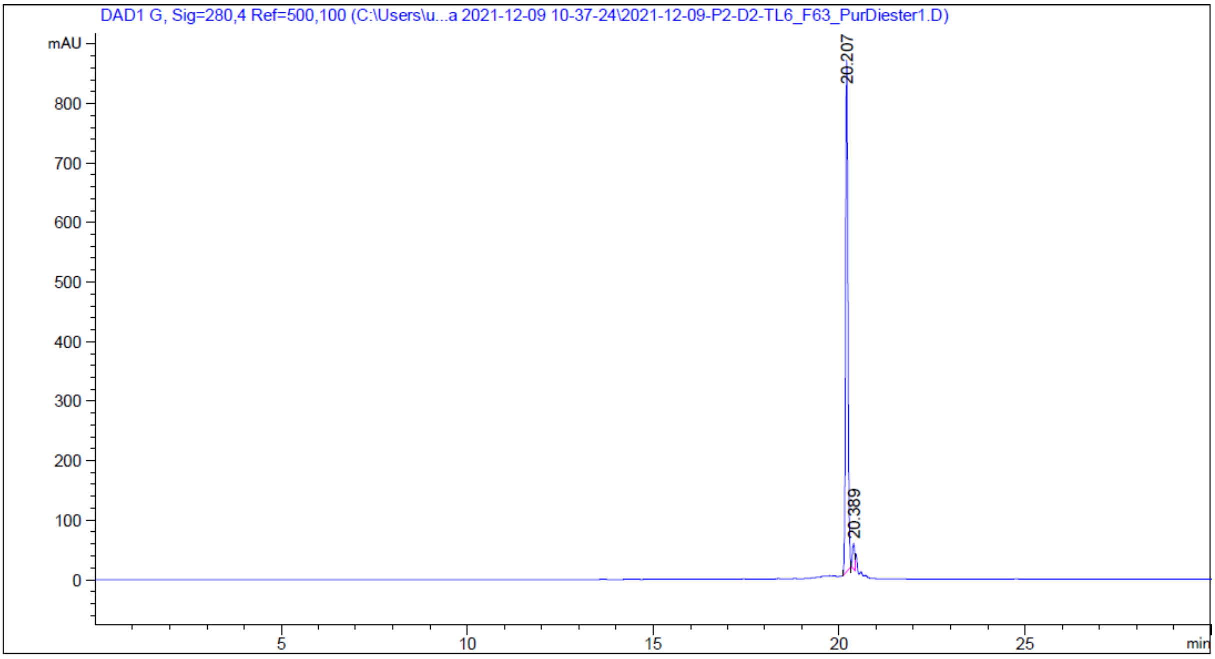

Peak #	RetTime [min]	Type	Width [min]	Area [mAU*s]	Height [mAU]	Area %
1	20.207	BB	0.0681	3769.46753	861.06512	95.6510
2	20.389	BV	0.0615	171.38843	42.06042	4.3490
Totals :				3940.85596	903.12555	

(–)-ESI-MS (*m/z*)

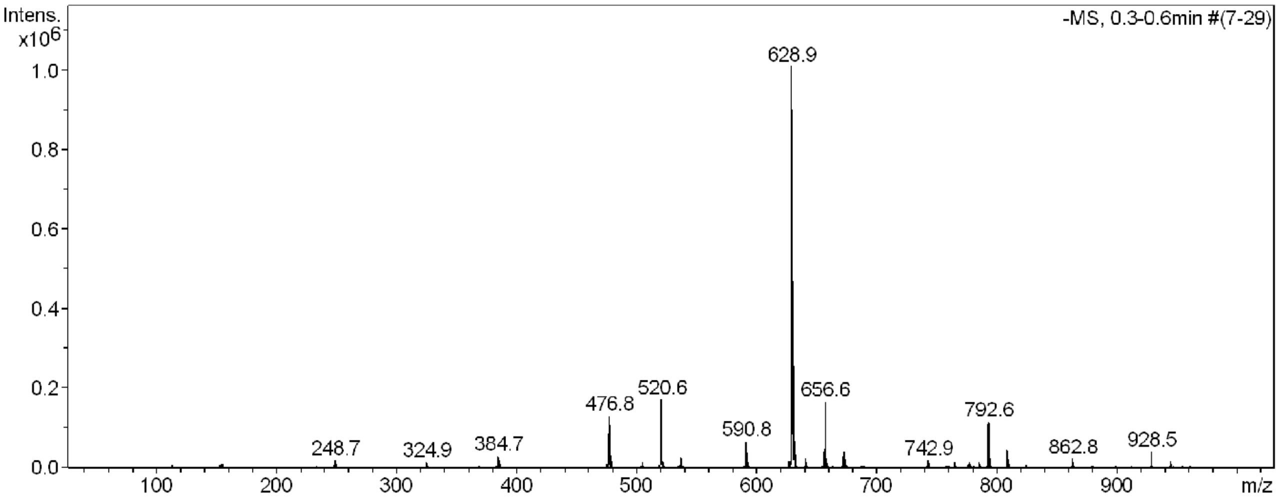

(–)-ESI-MS^2 (*m/z* = 628.9)

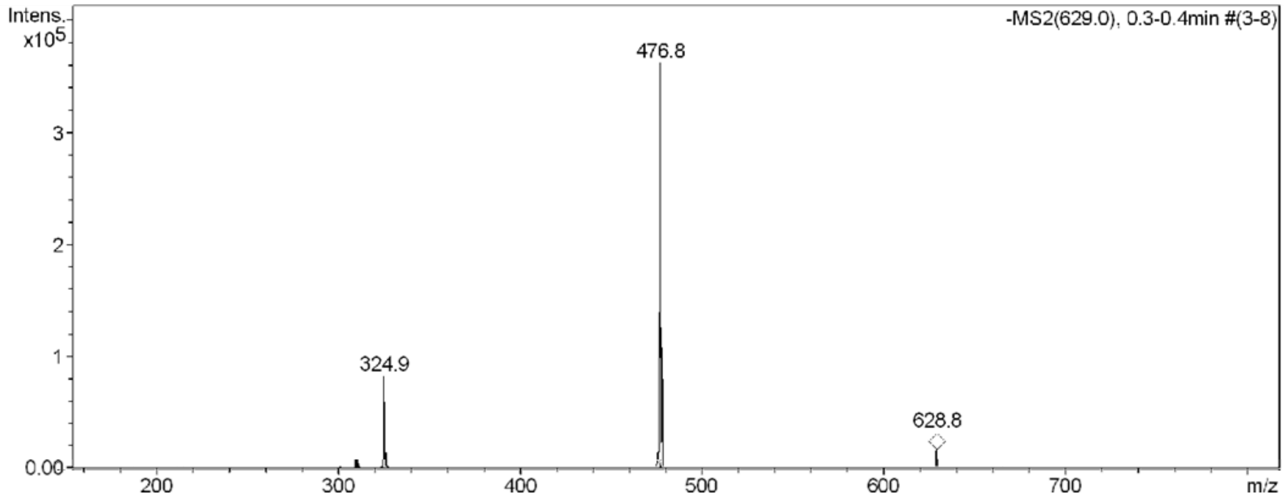

(–)-ESI-MS^3 (*m/z* = 476.8)

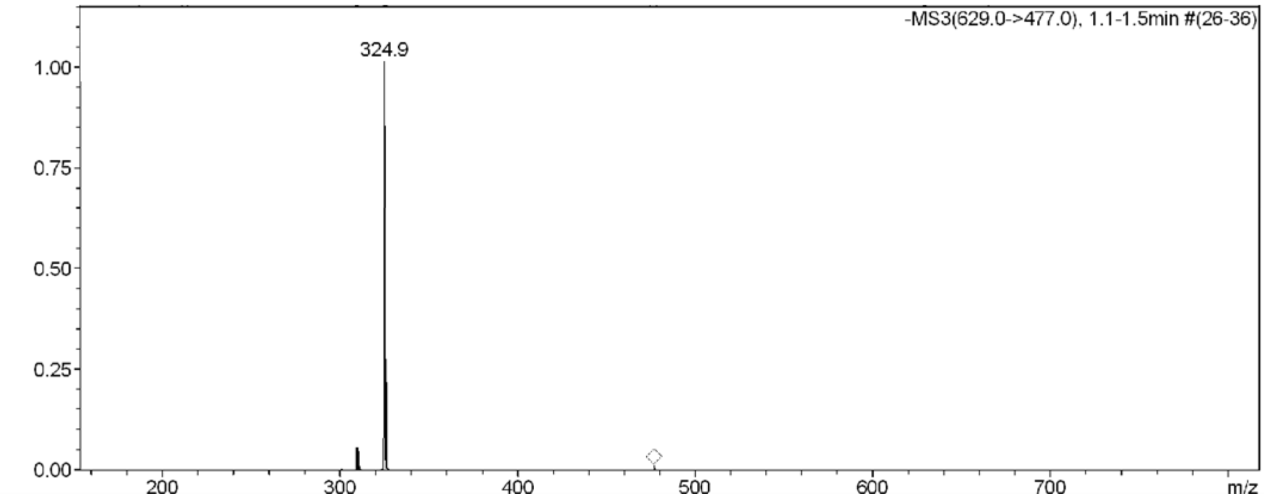

HRMS (*m/z*)

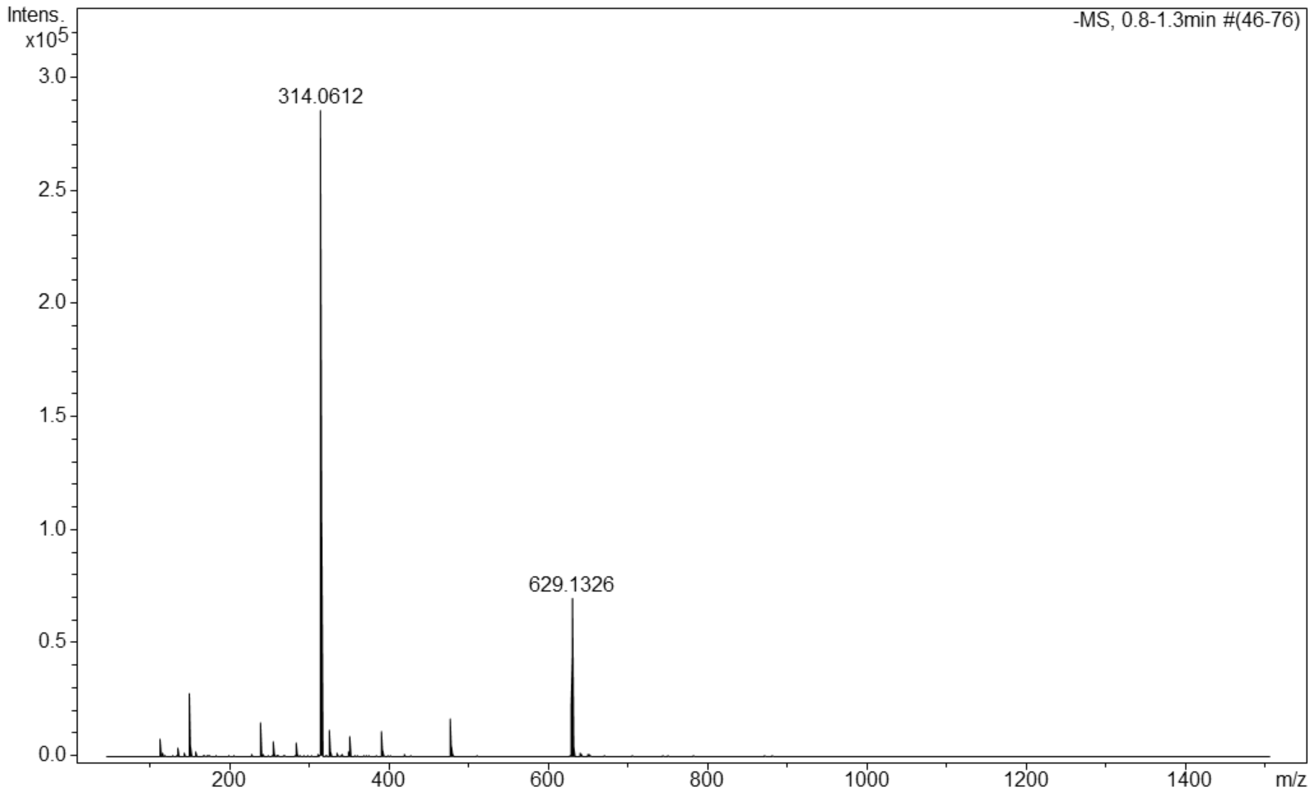
Intens.
x10^5
-MS, 0.8-1.3min #(46-76)
314.0612
629.1326
3.0
2.5
2.0
1.5
1.0
0.5
0.0
200
400
600
800
1000
1200
1400
m/z

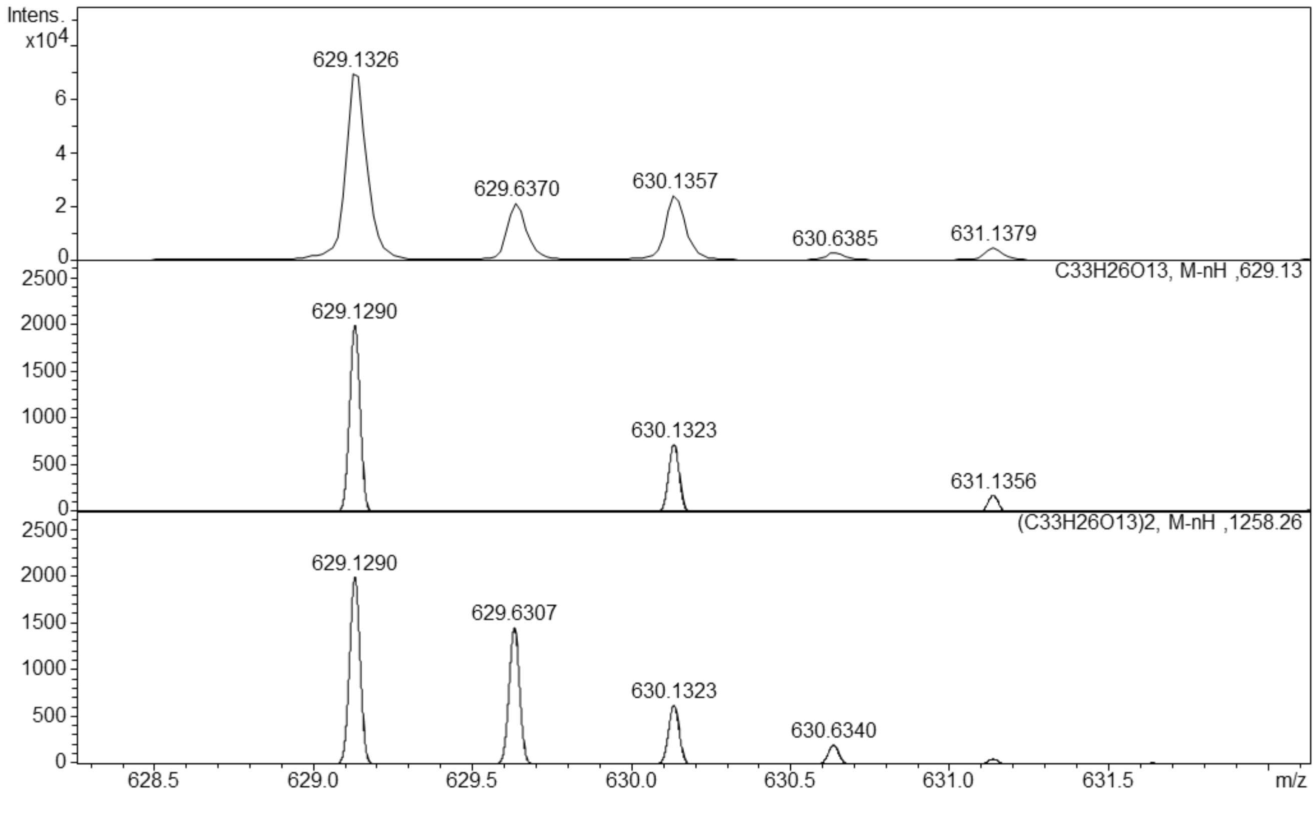
Intens.
x10^4
629.1326
629.6370
630.1357
630.6385
631.1379
C33H26O13, M-nH ,629.13
629.1290
630.1323
631.1356
(C33H26O13)2, M-nH ,1258.26
629.1290
629.6307
630.1323
630.6340
628.5
629.0
629.5
630.0
630.5
631.0
631.5
m/z

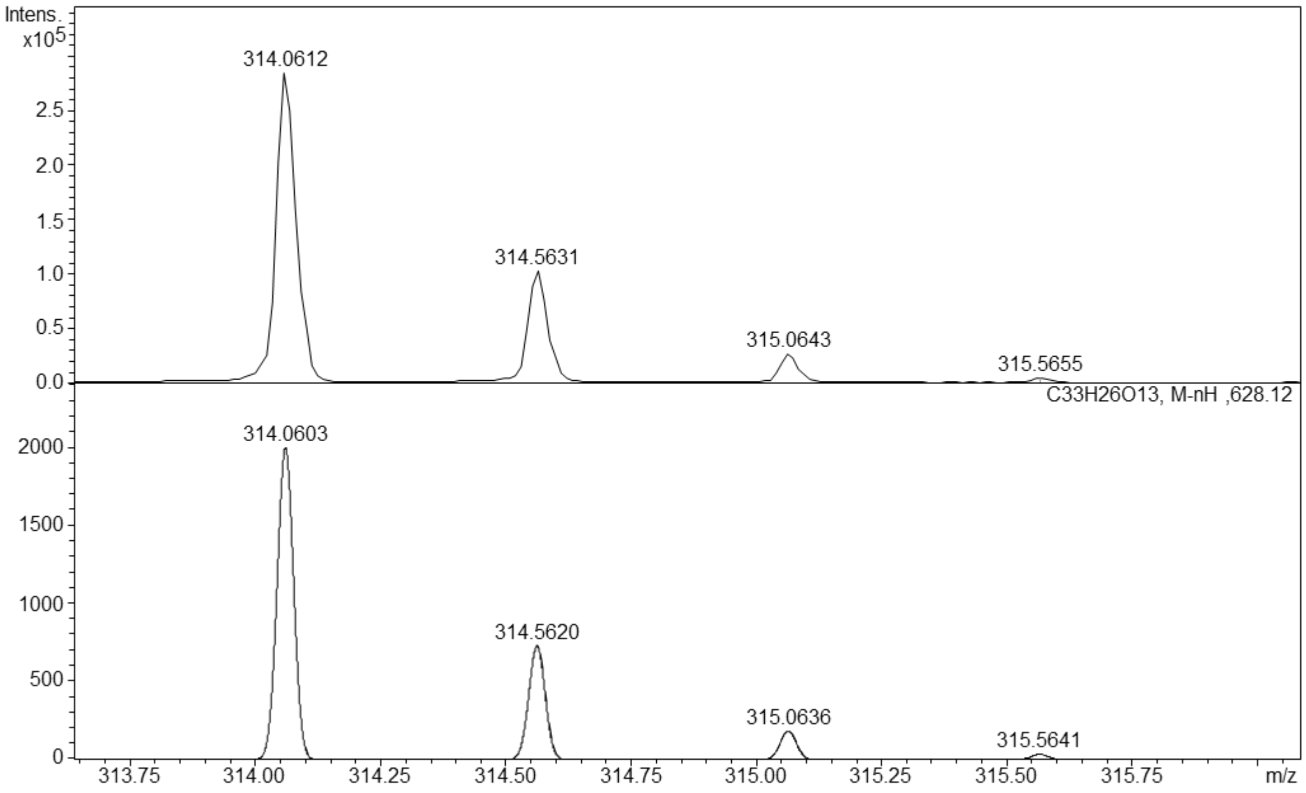

	Molecular formula	Calculated	Found
**[M–H]⁻**	$C_{33}H_{25}O_{13}$	629.1290	629.1326
**[2M–2H]⁻**	$(C_{33}H_{25}O_{13})_2$	629.1290	629.1326
**[M–2H]²⁻**	$C_{33}H_{24}O_{13}$	314.0612	314.0603
